# Fragment‐Based Drug Discovery of Novel High‐affinity, Selective, and Anti‐inflammatory Inhibitors of the Keap1‐Nrf2 Protein‐Protein Interaction

**DOI:** 10.1002/anie.202508121

**Published:** 2025-08-21

**Authors:** Chunyu Lin, Dilip Narayanan, Marilia Barreca, Cecilie Poulsen, Leandro Silva da Costa, Xiangrong Chen, Kendall G. Wichman, Cherisse A. Charley, Jaslin L. Lindsay, Mahya Dezfouli, Dimitra Vlissari, Thomas S. Mortensen, Camilla B. Chan, Jingyi Wang, William Richardson, Charlotte E. Manning, Zhuoyao Chen, Jie Zang, Helena Käck, Michael Gajhede, Alex N. Bullock, David J. Blake, David Olagnier, Anders Bach

**Affiliations:** ^1^ Department of Drug Design and Pharmacology, Faculty of Health and Medical Sciences University of Copenhagen Copenhagen 2100 Denmark; ^2^ Department of Biological, Chemical and Pharmaceutical Sciences and Technologies (STEBICEF) University of Palermo Palermo 90123 Italy; ^3^ Department of Biomedicine Faculty of Health Aarhus University Aarhus 8000 Denmark; ^4^ Centre for Medicines Discovery, Nuffield Department of Medicine University of Oxford Oxford OX3 7FZ UK; ^5^ Department of Biology Fort Lewis College Durango Colorado 81301 USA; ^6^ Assays Profiling and Cell Sciences, Discovery Sciences Biopharmaceuticals R&D AstraZeneca Gothenburg Sweden; ^7^ Novo Nordisk Foundation Center for Protein Research, Faculty of Health and Medical Sciences University of Copenhagen Copenhagen 2200 Denmark; ^8^ Protein Sciences, Structure and Biophysics, Discovery Sciences Biopharmaceuticals R&D AstraZeneca Gothenburg Sweden

**Keywords:** Fragment‐based drug discovery, Inflammation, Keap1, Oxidative stress, Protein‐protein interactions

## Abstract

Activating the cytoprotective response of nuclear factor erythroid 2‐related factor 2 (Nrf2) can reduce oxidative stress and inflammation. A promising strategy is to inhibit the protein‐protein interaction between Kelch‐like ECH‐associated protein 1 (Keap1) and Nrf2 using noncovalent compounds that target the Keap1 Kelch domain. These compounds may be more specific than covalent Keap1‐reacting Nrf2 activators. However, the development of drug‐like noncovalent Keap1‐Nrf2 inhibitors faces challenges due to the size and polarity of the Kelch binding pocket. Here, we present a new series of noncovalent Keap1‐Nrf2 inhibitors developed from a weak fragment hit identified by crystallographic screening. A two‐step growing strategy and optimization guided by several X‐ray cocrystal structures led to compounds with low nanomolar affinities and complete selectivity for Keap1 in a panel of homologous Kelch domains. In cells, compounds **24** and **28** potently activated the expression of Nrf2‐controlled genes and showed anti‐inflammatory effects by downregulating NLRP3 inflammasome and STING signalling activation. RNA sequencing revealed activation of cytoprotective pathways and a different profile from typical covalent Nrf2 activators. This work highlights the potential of fragment‐based drug discovery for challenging targets like Keap1 and introduces novel Keap1‐Nrf2 inhibitors as chemical probes and drug leads.

## Graphical abstract

We developed potent and selective noncovalent Keap1‐Nrf2 inhibitors through a structure‐guided fragment‐based approach. These nanomolar inhibitors activate Nrf2‐controlled genes, suppress inflammation via NLRP3 and STING pathways, and exhibit distinct transcriptional profiles compared to covalent Nrf2 activators, offering promising chemical probes and drug leads for oxidative stress‐related diseases.

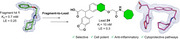

## Introduction

Protein‐protein interactions (PPIs) play essential roles in a vast range of cellular functions and are attractive drug targets in many diseases.^[^ ^1^, ^2^ ^]^ Initially considered “undruggable” by small molecules due to their large, shallow, and dispersed interfaces, advancements in drug discovery, e.g., the introduction of fragment‐based drug discovery (FBDD), have offered promise for discovering small‐molecule PPI modulators.^[^ ^3^, ^4^, ^5^ ^]^ FBDD operates by screening low‐molecular‐weight molecules (≤300 Da) with little chemical complexity, which tend to bind the “hot spots” of the protein target,^[^ ^6^ ^]^ followed by fragment‐to‐lead (F2L) optimization often guided by structural data. As of today, seven FBDD‐derived drugs have been approved, of which two are against PPIs.^[^ ^4^, ^5^ ^]^

The interaction between Kelch‐like ECH‐associated protein 1 (Keap1) and nuclear factor erythroid 2‐related factor 2 (Nrf2) constitutes an attractive drug target for diseases involving oxidative stress and inflammation.^[^ ^7^, ^8^, ^9^ ^]^ Keap1 functions as a molecular sensor for reactive oxygen species (ROS) and a negative regulator of Nrf2. Under physiological conditions, Keap1 targets Nrf2 for ubiquitin‐dependent degradation and keeps Nrf2 at a low cellular concentration. When cells are under electrophilic or oxidative stress, specific cysteine residues on Keap1 are affected. This leads to a conformational change in the Keap1‐Nrf2 complex, whereby Nrf2 escapes Keap1‐induced degradation, translocates to the nucleus, and activates the expression of antioxidant and cytoprotective genes.^[^ ^10^, ^11^, ^12^ ^]^ Covalent Nrf2 activators‒such as the marketed drugs dimethyl fumarate (DMF) and omaveloxolone for multiple sclerosis and Friedreich's ataxia‒directly react with the Keap1 cysteines. However, the reactivity of such compounds often leads to off‐target binding and hence potential toxicity issues and complicated modes of action.^[^ ^7^, ^13^, ^14^ ^]^ A promising alternative is to inhibit the Keap1‐Nrf2 PPI using noncovalent compounds that bind the Kelch domain of Keap1 that otherwise interacts with peptide motifs of the Nrf2 Neh2 domain.^[^ ^13^, ^14^, ^15^ ^]^ Such compounds are likely more specific than covalent Nrf2 activators and therefore also useful as chemical probes.^[^ ^16^ ^]^

A main challenge in developing effective noncovalent Keap1‐Nrf2 inhibitors is the Keap1 Kelch binding pocket itself. The buried surface area of the Kelch‐Nrf2 interaction is 550–780 Å^2^,^[^ ^17^ ^]^ which is smaller than many PPIs, but larger than typical small molecule‐protein interactions.^[^ ^18^ ^]^ Additionally, the Kelch binding pocket contains three centrally placed arginines (Arg380, Arg415, Arg483). Combined, these features often result in molecules that are relatively large (>500 Da) and contain carboxylic acids, and therefore show low cell permeability, reduced metabolic stability, and poor oral absorption.^[^ ^13^, ^19^ ^]^ Hence, to obtain biologically active Keap1‐Nrf2 inhibitors, it is essential to combine high affinity with physicochemical properties that counteract the polarity of the acid.^[^ ^20^, ^21^, ^22^, ^23^ ^]^ Recent efforts, including screening, structure‐based design, FBDD, prodrug development, and macrocycle design, have led to a rise in noncovalent Keap1‐Nrf2 inhibitors that combine high target affinity, cellular activity, and pharmacokinetic (PK) properties suitable for studies in animal disease models.^[^ ^13^, ^14^, ^15^ ^]^ These studies demonstrate that noncovalent inhibition of the Keap1‐Nrf2 PPI is a promising strategy for various diseases associated with oxidative stress, inflammation, and fibrosis, such as chronic obstructive pulmonary disease (COPD), metabolic dysfunction‐associated steatohepatitis (MASH), and chronic kidney disease (CKD).^[^ ^13^ ^]^ However, to our knowledge, no noncovalent Keap1‐Nrf2 inhibitors have reached clinical trials, likely due to the challenge of combining necessary properties into a single drug candidate, as well as the complexity of the mentioned diseases and general drug development hurdles.

To further advance the field, we here use FBDD to identify a new series of chemical probes and drug leads. We present a clear FBDD case, in which a fragment hit with millimolar affinity is optimized to nanomolar Keap1‐Nrf2 inhibitors while maintaining the core structure and binding mode of the original fragment. We demonstrate that the compounds are selective, potent in cells, and exhibit anti‐inflammatory effects. Additionally, we utilize RNA sequencing to compare the cytoprotective pathways they activate with those of typical covalent Nrf2 activators.

## Results and Discussion

**Figure 1:**
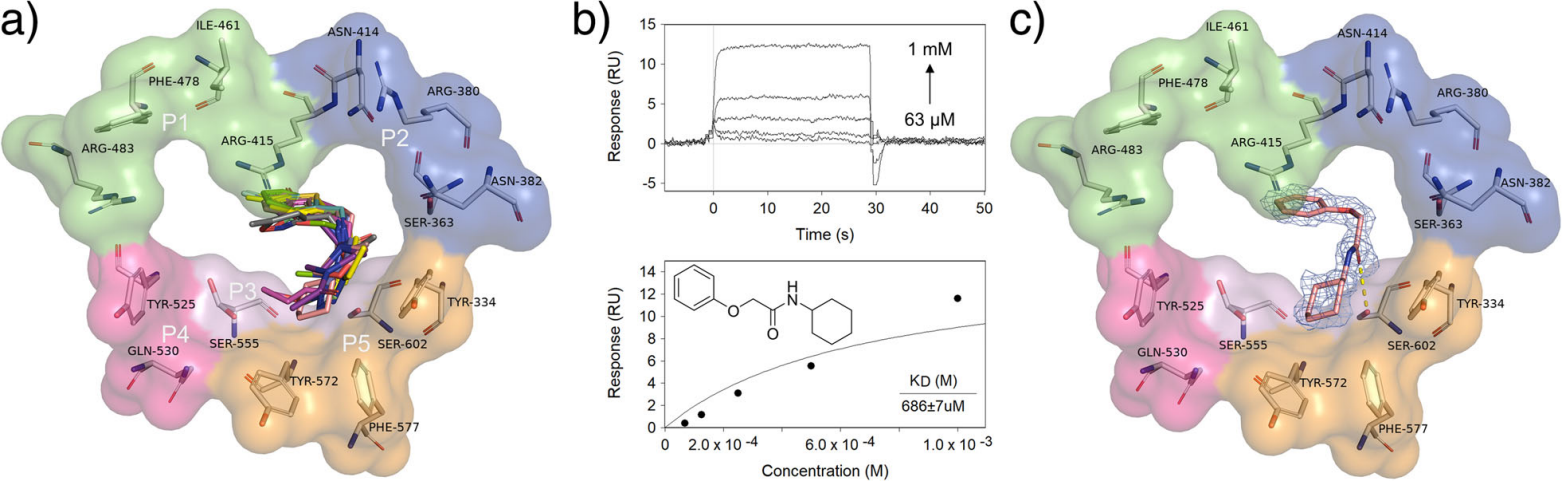
Fragment screening and data leading to selection of fragment **1** for F2L optimization. a) X‐ray crystal structures of the 13 fragment hits binding in the P3/P5 subpockets of the Keap1 Kelch domain. b) SPR sensorgram of **1** injected in 2‐fold serial dilutions over immobilized Keap1 Kelch and plot of equilibrium binding responses against the injected concentrations below. The *K*_d_ value was estimated by fixing R_max_ to the theoretical maximum value (15 RU) of **1** based on the immobilization level (2145 RU). c) X‐ray crystal structure of fragment **1** in complex with the Keap1 Kelch domain (PDB ID: 9HWQ). Hydrogen bond is shown as yellow dashed lines. Standard 2*F*o−*F*c electron density map around the fragment at 1.14Å (blue) contoured at 1*σ* and carved at 1.6Å is shown.

A total of 768 fragments from the DSI‐poised library^[^ ^24^ ^]^ were screened against the Keap1 Kelch domain using X‐ray crystallography at the XChem platform of Diamond Light Source. Data processing resulted in 80 high‐resolution (1.0–2.1Å) crystal structures of bound fragments, which together covered the entire Keap1 Kelch pocket, including the five subpockets P1‒5.^[^ ^25^ ^]^ Thirteen of the fragment hits were anchored in the central P3 region extending to the hydrophobic P5 subpocket (Figure 1a). P3 and P5 are known to accommodate several high‐affinity Keap1‐Nrf2 inhibitors; thus, we focused on these 13 fragments in our further analysis. Five of the hits displayed the same binding mode in molecular docking as observed in X‐ray crystallography, offering an advantage for structure‐based optimization. Re‐synthesis of these five fragments and testing by surface plasmon resonance (SPR)‒which we have previously used to measure weak Keap1 binders with millimolar affinities^[^ ^20^, ^22^ ^]^‒revealed clear, robust binding for fragment **1** (Figure 1b). This fragment consists of a central phenyl core and a cyclohexylacetamide substituent and binds the Keap1 Kelch domain with a *K*_d_ of approximately 0.7 mM and hence a ligand efficiency (LE) of 0.25. The four other fragment hits showed very weak binding with *K*_d_ values >3 mM (Figure S1). Detailed analysis of the X‐ray crystallographic structure of fragment **1** with the Keap1 Kelch domain, solved at a resolution of 1.14Å (Table S1), revealed three major interactions with the binding pocket (Figure 1c): a cation‐π interaction between Arg415 and the benzene moiety of **1**, a hydrogen bond interaction between the amide carbonyl with Ser602, and the cyclohexyl group of **1** occupying the hydrophobic subpocket P5. Based on these data and its synthetic tractability, fragment **1** was selected as a starting point for F2L elaboration.

**Figure 2:**
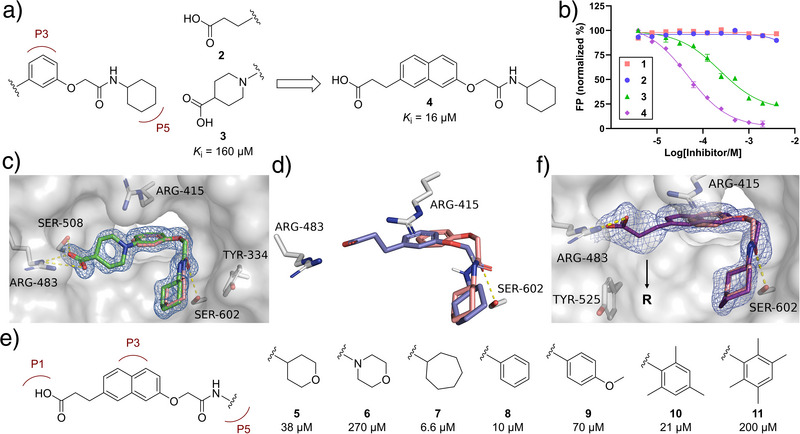
Initial F2L optimization. a) Investigating acid linkers with compounds **2**–**4**. b) Representative FP data for fragment hit **1** and acid analogues, compounds **2**−**4**. c) X‐ray crystal structure of compound **3** (green) in complex with the Keap1 Kelch domain (PDB ID: 9HWR), superimposed with **1** (salmon). d) Molecular docking of **2** (deep blue) shows misalignment with fragment **1** and loss of key interactions with Keap1. e) Structures and *K*_i_ values of compounds **5**–**11**. Mean *K*_i_ values determined by FP based on ≥3 individual measurements are shown (SEM values are seen in Table S2). f) X‐ray crystal structure of **7** (deep purple) in complex with the Keap1 Kelch domain (PDB ID: 9HWS), superimposed with **1**. Standard 2*F*o−*F*c electron density maps (blue) around compounds **3** and **7** contoured at 1*σ* and carved at 1.6Å are shown. Hydrogen bonds and salt bridges are shown as yellow dashed lines. See Supporting Information for the synthesis of **1**‒**11**.

In the first stage of the F2L process, we used a structure‐guided growing strategy to improve binding affinity. Our initial approach involved extending fragment **1** with an acidic aliphatic chain to form a salt bridge with Arg483 at the P1 hotspot. Analogues **2** and **3** were designed by attaching a flexible linear propanoic acid chain and a rigid piperidine carboxylic acid chain to the *meta*‐position of fragment **1′**s benzene ring, respectively (Figure 2a). A fluorescence polarization (FP) competition assay was used to determine the binding affinities between Keap1 and compounds.^[^ ^22^ ^]^ While compound **2** and the original fragment **1** were inactive in this assay, compound **3** showed notable inhibition, with a *K*_i_ value of 160 µM (Figure 2b and Table S2). In addition, an X‐ray cocrystal structure of compound **3** with the Keap1 Kelch domain revealed a perfect overlap in binding mode with fragment **1** (Figure 2c and Table S1). However, the affinity of 160 µM and the resulting low LE of 0.20 led us to reconsider the design. Molecular docking suggested that compound **2** shifted away from fragment **1′**s original position to form a salt bridge with Arg483 (Figure 2d), thereby losing key interactions to the Kelch domain. To address this, we replaced the benzene ring in **2** with a naphthalene ring, thereby extending the linker to P1, anticipating that this would preserve the pose and key interactions of the fragment moiety. Encouragingly, the resulting compound **4** demonstrated a marked improvement in affinity and LE (*K*_i_ = 16 µM; LE = 0.25) (Figure 2a,b). Given the favorable affinity and LE of compound **4**, we synthesized seven analogues (compounds **5**–**11**) to investigate the structure‐activity relationship (SAR) of the P5‐binding region (Figure 2e). The cycloheptyl analogue **7** showed 2‐fold improved binding affinity compared to compound **4** (Figures 2e and S2), while maintaining LE (0.26). Further, X‐ray crystallography confirmed the expected binding mode showing that **7** engages with the previously unoccupied subpocket P1, interacts with Arg483, and overlaps with the original hit, fragment **1** (Figure 2f and Table S1).

**Figure 3:**
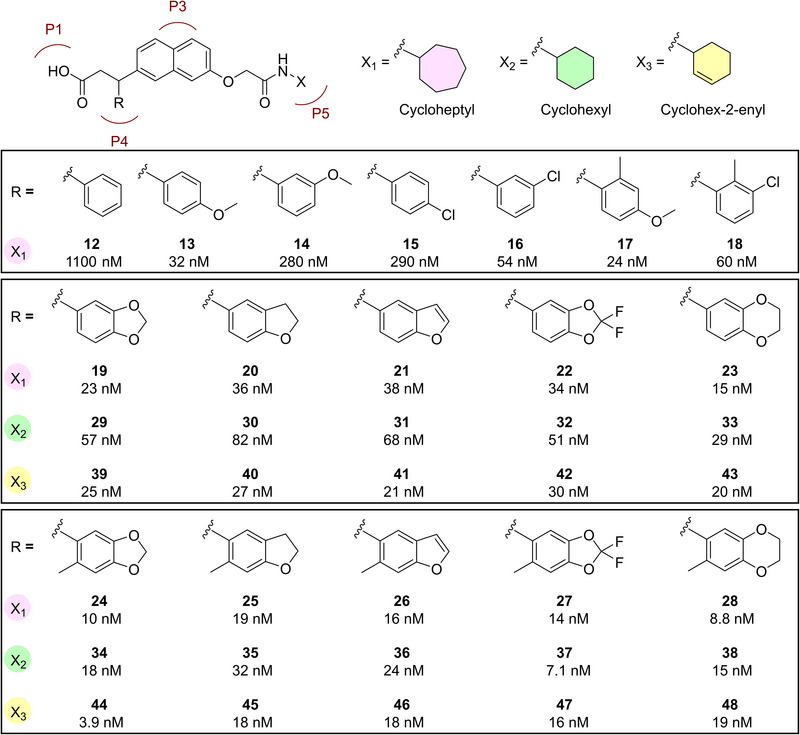
Systematic F2L optimization combining three P5 binding substituents with a range of P4 binding groups resulting in a diverse range of novel high‐affinity (low nanomolar) Keap1‐Nrf2 inhibitors. The mean *K*_i_ values determined by FP based on ≥3 individual measurements are shown, while SEM values are found in Table S2. See Supporting Information for the synthesis of **12**‒**48**.

The X‐ray costructure of **7** binding to Keap1 Kelch showed that the hydrophobic subpocket P4 is unoccupied (Figure 2f). Our strategy for the second stage of the F2L optimization process, therefore involved introducing an aromatic substituent to the acid linker, anticipating an additional π‐π stacking interaction with the side chain of Tyr525. Encouragingly, the phenyl‐substituted analogue of **7**, compound **12**, displayed a 6‐fold increase in binding affinity in the FP assay (*K*_i_  =  1.1 µM) and similar LE of 0.25 (Figures 3, S2, and Table S2). Next, a methoxy group was added at the benzene *para*‐position of **12**, resulting in compound **13**. The rationale was to further boost the affinity by introducing a hydrogen bond acceptor for interaction with the deeper parts of P4. This led to a remarkable 34‐fold improvement in affinity and thus the first molecule exhibiting nanomolar binding affinity (*K*_i_ = 32 nM) (Figure 3) together with a much improved LE value (LE = 0.29). We then explored other simple mono‐ and di‐substituent patterns, as in **14**–**18**, showing that an extra *ortho*‐methyl group slightly increased the affinity (**17** vs. **13**) and that meta‐methoxy, as in **14**, increased the affinity about four‐fold relative to **12**. Combining the two favorable meta‐ and para‐methoxy substituents into the benzodioxole analogue **19** led to a new high‐affinity Keap1‐Nrf2 inhibitor with *K*_i_ = 23 nM and preserved LE of 0.29 (Figure 3). This incited the synthesis of a subseries of benzodioxole analogues either without (**20**–**23**) or with (**24**–**28**) an *ortho*‐methyl on the P4‐binding substituent to gain affinity. The benzodioxine analogue **23** showed higher affinity (*K*_i_ = 15 nM) than benzodioxole **19**, and *ortho*‐methylation improved affinities 1.7‒2.4 fold across the entire subseries, leading to high‐affinity and ligand‐efficient compounds, such as **24** and **28** (*K*_i_  =  10 and 8.8 nM; LE  =  0.30 and 0.29, respectively) (Figure 3).

Further, building on previous studies highlighting the critical role of physicochemical properties like tPSA and cLogD for drug‐like characteristics–such as solubility, metabolic stability, membrane permeability, and hence cell activity^[^ ^20^, ^21^, ^22^, ^23^ ^]^–we aimed to cover a wide range of these properties to enhance the likelihood of identifying compounds with optimal profiles. We therefore expanded the SAR study by replacing the cycloheptyl group with cyclohexyl (compounds **29**‒**38**) and cyclohex‐2‐enyl (compounds **39**–**48**); substitutions that reduce cLogD with about 0.44 and 0.65, respectively. At the same time, tPSA and MW were within a reasonably low range (76‒94 Å^2^ and 446‒540 g mol^−1^) for compounds **12**‒**48** (Table S2 and Figure S3). Like the affinity difference between **7** and **4**, introducing the cyclohexyl instead of cycloheptyl generally led to a two‐fold reduction in affinity, except for **37** that showed a two‐fold improvement in affinity (*K*_i_ = 7.1 nM) relative to **27** (*K*_i_ = 14 nM) (Figure 3). The cyclohex‐2‐enyl analogues showed similar or slightly improved affinities compared to their cycloheptyl counterparts, with compound **44** having an especially high inhibitory activity (*K*_i_ = 3.9 nM) (Figure 3). Overall, this structure‐guided and systematic F2L optimization process resulted in a novel series of Keap1‐Nrf2 inhibitors with favorable physicochemical properties and high affinities, several showing very low and even single‐digit nanomolar *K*_i_ values (e.g., **24**, **28**, **37**, and **44**).

**Figure 4:**
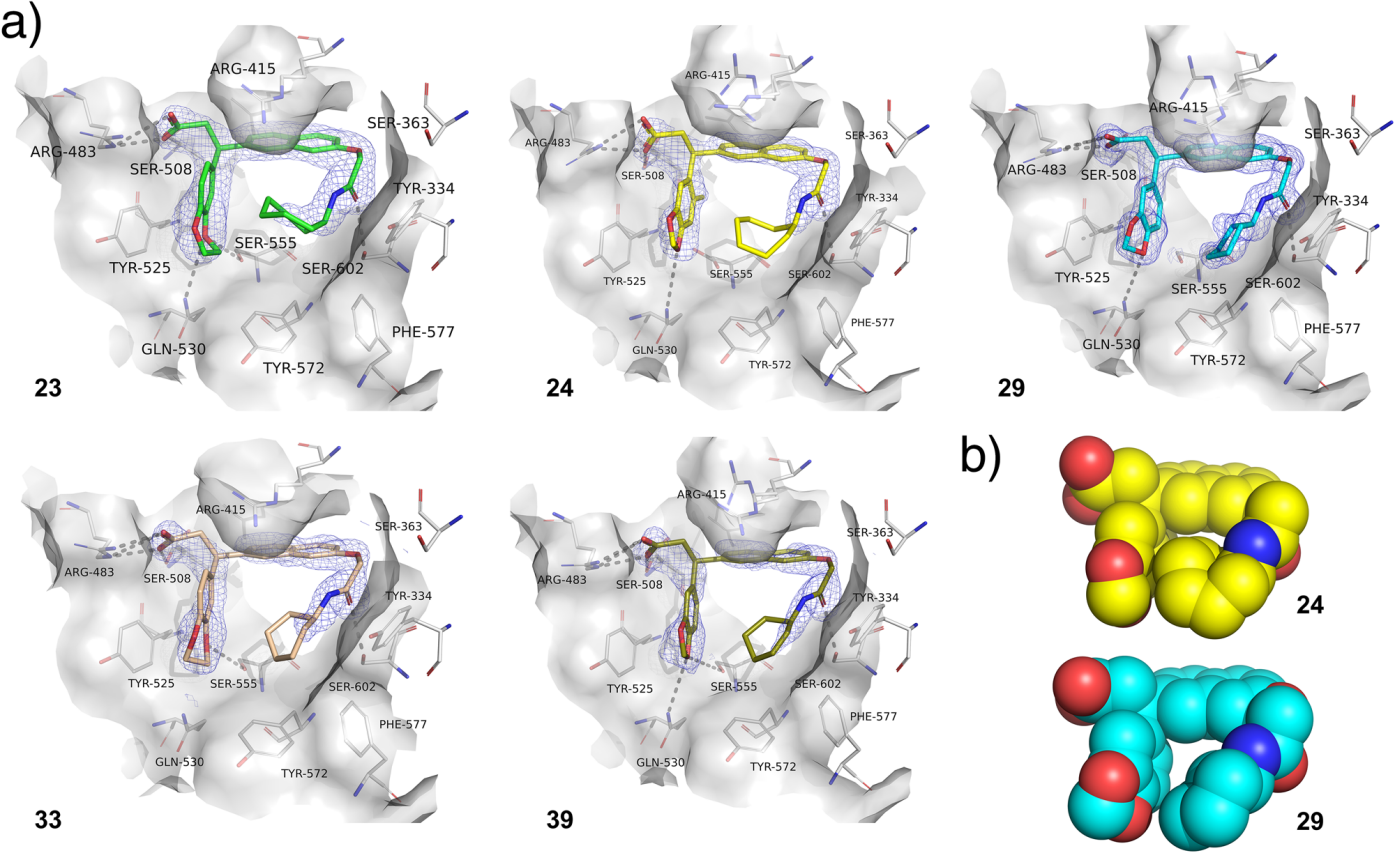
X‐ray structures of the Keap1 Kelch domain in complex with novel high‐affinity Keap1‐Nrf2 inhibitors. a) **23** (PDB ID: 9HWX), **24** (PDB ID: 9HWT), **29** (PDB ID: 9HWU), **33** (PDB ID: 9HWW), and **39** (PDB ID: 9HWV). Standard 2*F*o−*F*c electron density maps (blue) around the compounds contoured at 1*σ* and carved at 1.6Å are shown. Hydrogen bonds and salt bridges are shown as black dashed lines. b) Intramolecular contacts and hydrophobic interactions may facilitate the binding conformation of **24** and **29**.

The binding modes were determined by X‐ray crystallography for five of the lead‐like high‐affinity Keap1‐Nrf2 inhibitors (**23**, **24**, **29**, **33**, and **39**) and found to be similar to each other and with a preservation of the original fragment moiety (Figure 4a). In all cases, the *S*‐enantiomer fitted better into the electron density maps than the *R*‐enantiomer, indicating a binding preference for this enantiomer. This was confirmed by chiral HPLC separation, providing the pure enantiomers of compound **24** (≥99% ee)‒which showed a 14‐fold difference in affinity by FP‒and docking analysis (Figure S4). The core naphthalene occupies the central P3 pocket stabilized by interactions with Arg415, and the amide carbonyl serves as a hydrogen bond acceptor to Ser602, while the cycloheptyl (**23**, **24**), cyclohexyl (**29**, **33**), and cyclohexenyl (**39**) groups bind the hydrophobic P5 subpocket. The carboxylic acid group of the compounds forms charge‐assisted hydrogen bonds to Arg483 and Ser508, and the P4 binding compound moieties engage in π‐π stacking with the Tyr525 side chain. Also, the heterocyclic part of the benzodioxole/dioxine ring systems interacts with Gln530 and/or Ser555 via hydrogen bonds and generally extends the interactions with Tyr525, explaining the boost in affinity when introducing this group. We also noticed a very close proximity of the cyclic aliphatic groups and the heterocyclic P4 binding groups, which is most prevalent for the high‐affinity compounds, as seen when comparing **24** (*K*_i_ = 10 nM) with **29** (*K*_i_ = 57 nM) (Figure 4b). This might indicate the formation of a pre‐organized conformation mediated by a “hydrophobic” collapse, as suggested for other Keap1‐Nrf2 inhibitors,^[^ ^26^ ^]^ which could contribute to the high affinity and may be further enhanced by the extra methylene groups in cycloheptyl and benzodioxine compared to cyclohexyl and benzodioxole, respectively.

**Figure 5:**
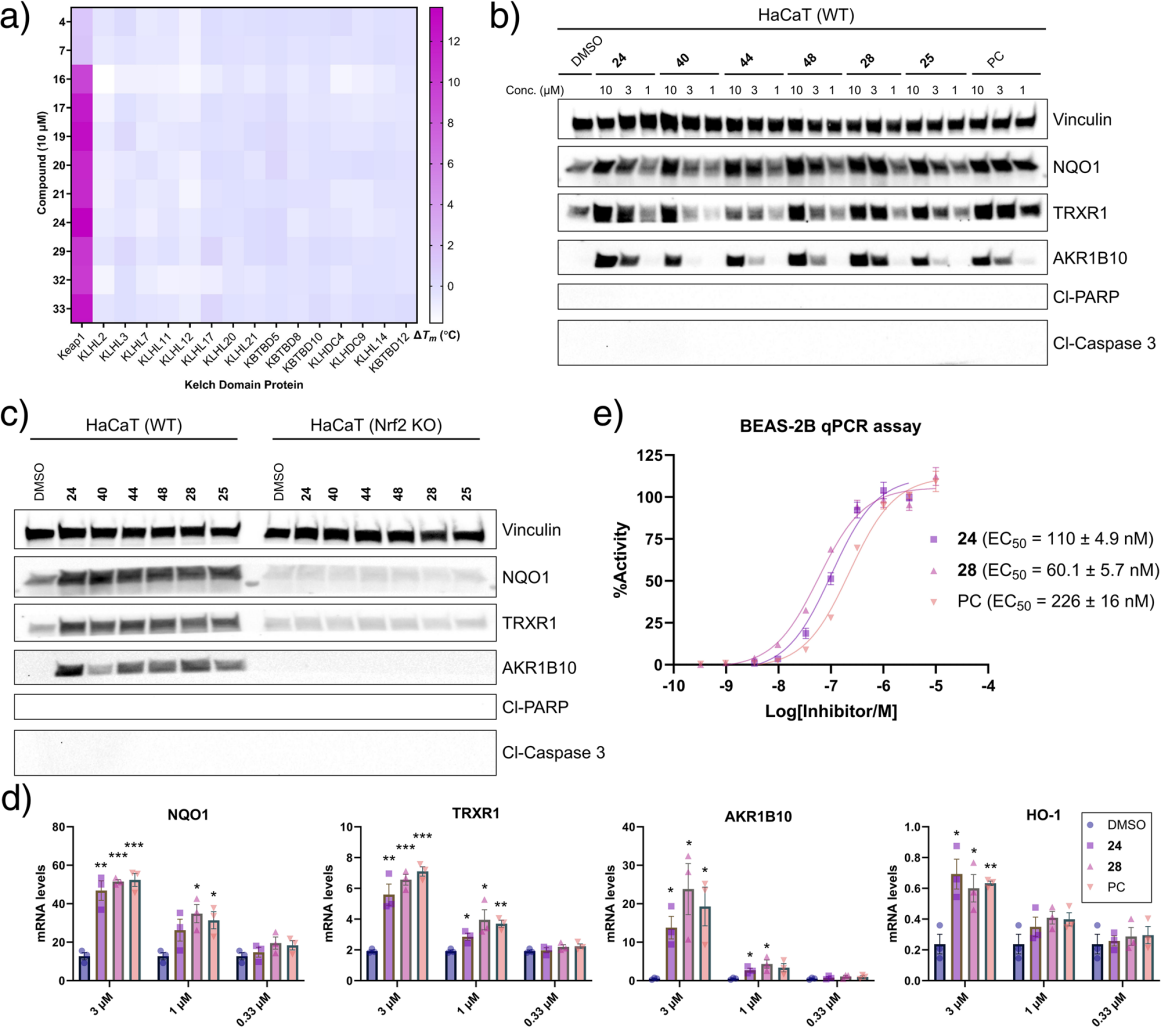
Cell activity. a) Kelch domain selectivity profile. Heat map depicting Δ*T*_m_ values (°C) of the Kelch domain proteins treated with compounds at 10 µM (*n* = 3). b) Protein levels from whole cell lysates assessed by WB after treating HaCaT cells with six of the most cell‐potent compounds and the PC at 10, 3, and 1 µM for 24 h. c) WB analysis after treating wild‐type (WT) and Nrf2 KO HaCaT cells with the six compounds at 10 µM for 24 h. “Cl” = cleaved. d) Expression of Nrf2‐regulated genes at the mRNA level was determined by RT‐qPCR after treating WT HaCaT cells with compounds at 3, 1, and 0.33 µM for 5 h. Data are shown as the mean of three independent experiments performed in duplicates, where error bars represent the SEM. TATA‐box binding protein (TBP) is used for normalization. Statistical analyses were performed using a Student's t‐test (*p< 0.05, **p< 0.01, ***p<0.001). e) qPCR‐based BEAS‐2B cell assay data of **24**, **28**, and the PC. Mean EC_50_ values ± SEM are shown based on 2‒4 independent experiments.

We next examined the pharmacological properties of our compounds in a range of experiments. First, selectivity within the family of Kelch domain proteins was assessed employing a thermal shift assay (TSA).^[^ ^23^, ^27^ ^]^ Sixteen human Kelch domains were expressed and purified (Figure S5) and the potential binding of several analogues was tested. Keap1 Kelch exhibited clear observable Tm shifts (Δ*T*_m_) for all compounds (Figure S5), while no or negligible shifts were measured for the other Kelch proteins at 10 µM compound concentration (Figure 5a) thereby demonstrating high selectivity for Keap1.

We then evaluated the ability of the compounds to activate Nrf2‐regulated genes in HaCaT human keratinocytes. The target genes included aldo‐keto reductase family 1 member B10 (AKR1B10), NAD(P)H quinone dehydrogenase 1 (NQO1), thioredoxin reductase 1 (TRXR1), and heme oxygenase‐1 (HO‐1).^[^ ^28^ ^]^ Activation was assessed at the protein level using Western Blotting (WB) and at the mRNA level using quantitative reverse transcription PCR (RT‐qPCR). A noncovalent, membrane‐permeable, high‐affinity 1‐phenylpyrazole‐based Keap1‐Nrf2 inhibitor from literature^[^ ^21^ ^]^ was used as the positive control (PC) (Figure S6), consistent with its use in our previous studies.^[^ ^22^, ^23^, ^29^ ^]^ An initial screening of 19 analogues at 30 µM was unable to differentiate the cell potencies, as intense bands were seen in WB for all the compounds and proteins (data not shown). Instead, testing at 10 µM identified compounds **24**, **25**, **28**, **40**, **44**, and **48** as the most cell‐active, producing strong bands in WB for all tested proteins (Figure S6a). These six compounds were then tested at three concentrations, revealing clear activity at 3 and 10 µM, while at 1 µM, activity was minor (Figure 5b). Overall, **24** and **28** emerged as the most potent. Furthermore, the apoptosis markers‒cleaved poly(ADP‐ribose) polymerase (PARP) and cleaved caspase‐3‒were not detected by WB (Figure 5b), and the dependency on Nrf2 was confirmed by the absence of protein upregulation in Nrf2 knockout (KO) cells (Figure 5c). At the transcription level, **24** and **28** induced several‐fold increases in target gene mRNA expression and demonstrated dose‐dependent activity down to 1 µM, comparable to the PC (Figure 5d).

To further explore the cellular activity, we tested **24** and **28** in BEAS‐2B cells, derived from normal human bronchial epithelium and commonly used to evaluate Keap1‐Nrf2 inhibitors.^[^ ^13^ ^]^ For example, the 1‐phenylpyrazole‐based PC used herein has been reported to increase NQO1 activity with an EC_50_ of 220 nM.^[^ ^21^ ^]^ Here, we used a qPCR‐based assay to quantify compound‐induced expression of NQO1 mRNA,^[^ ^30^ ^]^ and found that the PC exhibited an EC_50_ of 226 nM (Figure 5e). Notably, compounds **24** and **28** demonstrated significantly higher cellular potency, with EC_50_ values of 110 and 60 nM, respectively (Figure 5e). These data underscore the effectiveness of our Keap1‐Nrf2 inhibitors in cells and also highlight that cellular activity can vary depending on the assay. This variability may arise from cell‐type‐specific effects or assay conditions, emphasizing the importance of using control compounds and validating activity in advanced, disease‐relevant cell models.

**Figure 6:**
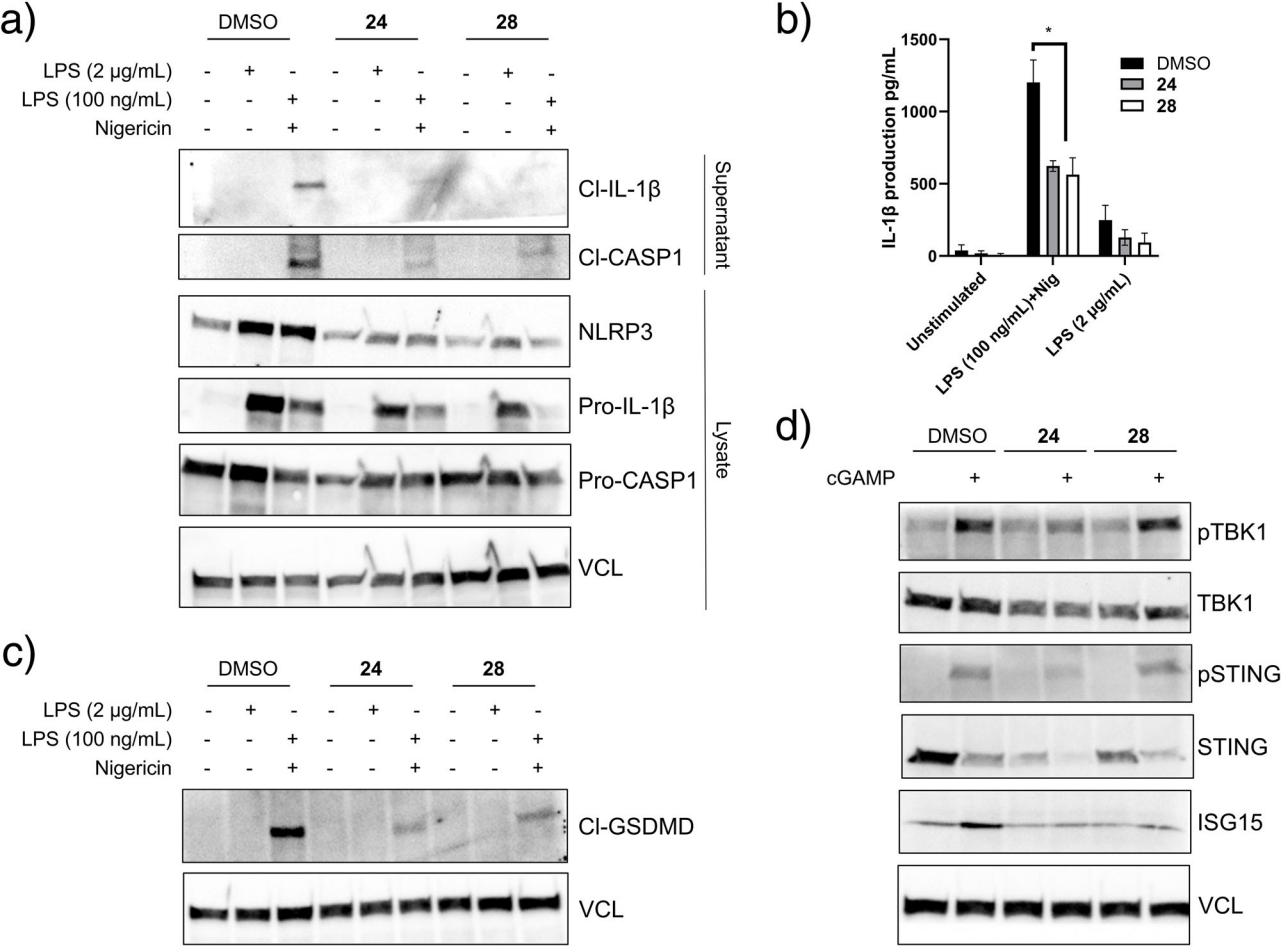
Keap1 targeting molecules, **24** and **28**, inhibit the NLRP3 inflammasome and STING pathways. Macrophage‐derived THP‐1 cells were treated with **24** and **28** (100 µM) or DMSO for 24 h. Then, a)–c) Inflammasome activation was evaluated in primed (100 ng mL^−1^ LPS, 3 h) cells treated with nigericin (10 µM, 1 h) or only stimulated with LPS (2 µg mL^−1^, 6 h). a, c) Supernatants and cell lysates were collected to quantify target proteins involved in inflammasome signalling (NLRP3, cleaved caspase‐1, cleaved IL‐1β, and cleaved gasdermin) by WB. b) IL‐1β release was quantified by ELISA in the supernatants of LPS+nigericin or LPS‐treated cells. d) The cGAS‐STING pathway was induced with cGAMP (4 µg mL^−1^, 6 h) after the 24 h compound treatment period, and cell lysates were analyzed by WB for the indicated proteins. Abbreviations: Cl (cleaved), p (phosphor); CASP1 (caspase‐1), VCL (vinculin).

Having demonstrated cellular target engagement, we proceeded to investigate the potential anti‐inflammatory effects of our compounds, as this may be relevant in addressing a range of inflammatory diseases. THP‐1 monocytes were differentiated into macrophages using phorbol 12‐myristate 13‐acetate (PMA). Then, Toll‐Like receptor 4 (TLR4) and the NLR family pyrin domain containing 3 (NLRP3) inflammasome were activated using high concentrations of lipopolysaccharide (LPS) (2 µg mL^−1^) or a combination of LPS (100 ng mL^−1^) and nigericin, respectively. Both **24** and **28** markedly reduced the expression of NLRP3, a key pattern‐recognition receptor (PRR) domain of the inflammasome,^[^ ^31^ ^]^ upon LPS or LPS+nigericin stimulation (Figure 6a). Additionally, the total protein levels of pro‐ILβ were also reduced (Figure 6a). Both **24** and **28** treatment markedly decreased NLRP3 inflammasome activation as seen by the reduction in the release of cleaved caspase‐1 and cleaved interleukin‐1 beta (IL‐1β) in the supernatants of treated cells (Figure 6a). ELISA of cell supernatants confirmed a decrease in IL‐1β release following NLRP3 activation and treatment with **24** and **28** (Figure 6b). The two compounds also showed a similar trend in reducing IL‐1β release following stimulation with high concentrations of LPS, although no statistical difference was observed (Figure 6b). Interestingly, **24** and **28** prevented the cleavage of gasdermin D (GSDMD), a process otherwise mediated by active caspase‐1 and leading to the formation of pores in the plasma membrane and pro‐inflammatory cell death (Figure 6c).

Since **24** and **28** impacted IL‐1β release following TLR4 and NLRP3 activation, we also investigated the impact of the two compounds on the cyclic GMP–AMP synthase (cGAS)–stimulator of interferon genes (STING) pathway and type‐I interferon (IFN) signalling.^[^ ^32^ ^]^ First, **24** and **28** reduced total STING levels and STING activation as apparent from a reduction in phosphorylated STING and TANK‐binding kinase 1 (TBK1) following cGAMP stimulation (Figure 6d). A dampening of STING signalling by **24** and **28** was also clear from the reduced levels of interferon‐stimulated gene 15 (ISG15) (Figure 6d). However, while STING signalling is being altered, **24** and **28** did not affect IFN signalling following IFNβ stimulation as no change in phosphorylated STAT1 or IFIT1 protein levels were observed (Figure S7). Overall, these data demonstrate that compounds **24** and **28** have anti‐inflammatory effects by downregulating NLRP3 inflammasome and STING signalling.

**Figure 7:**
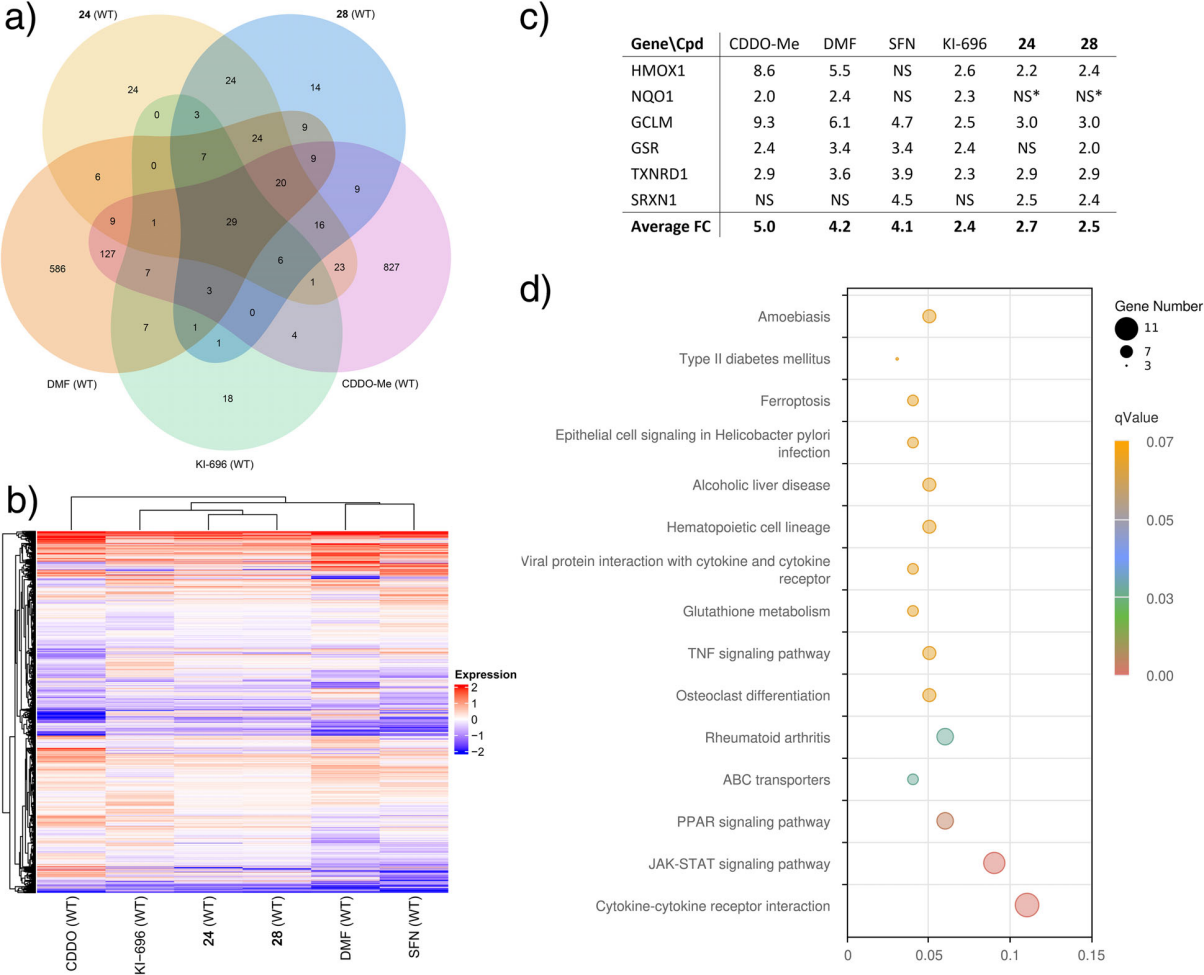
Transcriptomic analysis of differentiated THP1 macrophages treated with covalent Nrf2 activators (CDDO‐Me at 0.1 µM; SFN and DMF at 10 µM) and noncovalent Keap1‐Nrf2 inhibitors (KI‐696 at 1 µM; **24** and **28** at 10 µM) for 12 h (*n* = 3 technical replicates). a) Venn diagram showing the overall number of upregulated genes as defined by fold‐change (FC) >2.0 and *p*‐adj <0.05 in WT cells. b) Transcriptome profile for compound‐treated groups and control group shown as a Pearson correlation heatmap of fluctuating genes (FC>2 or <2; *p*‐adj< 0.05). c) Fold changes of upregulated Nrf2‐dependent genes compared to vehicle controls. NS=not significant. *At 24 h, 1.5‐fold upregulation was observed. d) KEGG analysis of differentially expressed up‐regulated genes in the presence of **24**. KEGG pathways with *q*‐values <0.05 were considered significantly enriched by differentially expressed genes.

We next conducted an expression profile analysis of our compounds. Covalent electrophilic compounds that react with the Keap1 cysteines and thereby activate Nrf2 are known for their promiscuity, which may contribute to the clinical effectiveness as well as side effects.^[^ ^13^ ^]^ Well‐designed noncovalent Keap1‐Nrf2 inhibitors, on the other hand, are expected to have much narrower off‐target profiles. Still, the plethora of >200 genes under Nrf2 control, downstream effects, and the fact that the Keap1 Kelch domain has other protein partners than Nrf2 make the pharmacological effects of this compound class complicated too. To obtain insight into which pathways are affected by our compounds and to compare their profiles with that of typical covalent Nrf2 activators we conducted a quantitative transcriptome analysis using RNA sequencing. THP‐1 cells were differentiated into macrophages with PMA and treated with noncovalent Keap1‐Nrf2 inhibitors‒our own **24** and **28**, and literature compound KI‐696^[^ ^33^ ^]^‒or covalent activators, the well‐studied sulforaphane (SFN), the multiple sclerosis drug DMF, and bardoxolone methyl (CDDO‐Me), which has been tested in several clinical trials,^[^ ^34^ ^]^ for 12 h. Principal components analysis (PCA) revealed that compounds **24**, **28**, and KI‐696 exhibit similar RNA expression profiles, consistent with their shared mechanism of action. Interestingly, these profiles differed from those of covalent inhibitors, with SFN and DMF showing the most distinct RNA signatures (Figure S8a). DMF and CDDO‐Me upregulated 845 and 1091 genes, respectively (Figure 7a), while SFN upregulated 676 genes (Figure S8b). In comparison, **24**, **28**, and KI‐696 upregulated much fewer genes–193, 175, 88, respectively (Figure 7a). As expected, several genes were overlapping, with 29 genes shared among all five treatment groups and 129 genes upregulated by both compounds **24** and **28**, of which 45 genes are shared with KI‐696. However, all compounds upregulated a set of unique genes too, especially DMF and CDDO‐Me, each with >500 genes not shared by the other groups (Figure 7a). The experiment was also conducted with a 24 h incubation, yielding similar results but with a weaker RNA expression response (data not shown).

Transcriptome analysis was also conducted on Nrf2 KO cells after 12 h of incubation to directly assess off‐target effects‒here referring to activities not related to Nrf2. This revealed a substantial number of up‐ *or* downregulated genes for the covalent Nrf2 activators (DMF: 3709; CDDO‐Me: 2149), while compounds **24** and **28** induced fewer expression changes (347 and 266, respectively), and, remarkably, KI‐696 showed none (Figure S8c), illustrating the greater specificity of noncovalent Keap1‐Nrf2 inhibitors over covalent Nrf2 activators. Here, it is important to note that CDDO‐Me and KI‐696 were tested at lower concentrations (0.1 and 1 µM, respectively) than the other compounds (10 µM) and that the observed non‐Nrf2 effects could be dose‐dependent. Also, expression changes observed in KO cells after 24 h with **24** and **28** were markedly reduced (data not shown). Importantly, exposure to 10 and 100 µM **24** or **28** did not reduce cell viability in THP‐1 WT cells even after 48 h, which contrasted with SFN and DMF at 100 µM (Figure S8d).

Heat map profiling of up‐ and downregulated genes confirmed similar pharmacological effects among the noncovalent Keap1‐Nrf2 inhibitors, whereas greater variability was observed among the covalent Nrf2 activators (Figure 7b). Several genes known to be regulated by Nrf2 and involved in maintaining the reducing environment of the cell were upregulated across the different compound groups, with compounds **24** and **28** showing fold changes comparable to KI‐696 (Figure 7c).

Furthermore, pathway analysis revealed that compound **24** activated other important gene clusters involved in cytokine receptor interaction and activation of immune cells, as well as JAK‐STAT and PPAR signalling pathways (Figure 7d). Specifically, upregulated genes involved with immune functions activating T‐cell (IL‐15), B cells (CXCL3), and neutrophils (IL‐8) were highly expressed (not shown). Finally, specific analysis revealed that several genes involved in fibrosis, inflammation, and ROS metabolism were favorably regulated (up‐ or downregulated) in THP‐1 cells following a 12 h incubation with compounds **24** and **28** (Table S3).

Overall, compounds **24** and **28** exhibited RNA expression profiles similar to KI‐696, consistent with their shared mechanism as noncovalent Keap1‐Nrf2 inhibitors, while the three covalent activators (SFN, DMF, CDDO‐Me) showed distinct and more variable signatures. Transcriptome analysis revealed fewer non‐Nrf2 effects‒used as a surrogate marker for direct off‐target effects‒for noncovalent inhibitors compared to covalent Nrf2 activators. Also, **24** and **28** activated specific Nrf2‐controlled genes and key immune signalling pathways. Their ability to robustly activate essential cellular defence mechanisms while maintaining low off‐target activity underscores their significant pharmacological potential.

## Conclusion

In this study, we developed a novel series of noncovalent Keap1‐Nrf2 inhibitors using a structure‐guided FBDD approach. Fragment hit **1** was identified via crystallographic screening and found to occupy the P3/5 subpocket of the Keap1 Kelch domain, while forming specific interactions with key amino acid residues. SPR characterization revealed a weak affinity of **1** (*K*_d_ ∼0.7 mM), but reasonable LE (0.25). We optimized the scaffold by a stepwise growing strategy to occupy the P1 and P4 subpockets and a F2L process that systematically explored a broad physicochemical space, focusing on tPSA and cLogD. This led to several high‐affinity and ligand‐efficient compounds, such as **24** and **28** (*K*_i_ = 9‒10 nM; LE = 0.29‒0.30). X‐ray crystallography of **24** and four other lead‐like compounds gave detailed insight into the binding modes. The fragment core maintained a conserved position and orientation in the binding pocket, while several new interactions were created, such as π‐π stacking with Tyr525 and hydrogen bonds with P4's Gln530 and Ser555. In addition, we observed a compact conformation of the compounds in the binding pocket, likely driven by intramolecular interactions and contributing to the high affinity.

The compounds demonstrated excellent selectivity for Keap1 over 15 homologous Kelch domains, and the most potent compounds, **24** and **28**, exhibited strong cellular activity, upregulating key Nrf2‐controlled cytoprotective genes, consistent with their intended mechanism of action. Cellular potency varied depending on the assay, with EC_50_ values of 110 and 60 nM as measured in the qPCR‐based BEAS‐2B cell assay for **24** and **28**, respectively, being particularly promising. Importantly, our inhibitors also exhibited robust anti‐inflammatory effects by downregulating NLRP3 inflammasome activation, IL‐1β release, and STING signalling. Transcriptomic analysis via RNA sequencing confirmed that compounds **24** and **28** activated Nrf2‐dependent genes while displaying distinct transcriptional signatures compared to the covalent activators SFN, DMF, and CDDO‐Me known to suffer from off‐target effects due to their intrinsic electrophilicity. Compounds **24** and **28** also favorably regulated important genes involved in fibrosis, inflammation, and ROS production. Hence, with reduced off‐target activity and activation of protective cellular pathways, we believe these novel noncovalent Keap1‐Nrf2 inhibitors hold therapeutic potential.

Overall, our findings underscore the value of FBDD in tackling challenging PPIs and provide a strong foundation for the continued development of selective Keap1‐Nrf2 inhibitors as both chemical probes and potential drug candidates. Future optimization and studies will address the compounds’ efficacy in animal disease models associated with oxidative stress and inflammation.

## Supporting Information

Supporting Information includes tables (including X‐ray data collection and refinement statistics of the eight deposited PDB structures), figures, experimental methods, chemical synthesis and compound characterization data, including LC‐MS and NMR spectra. The authors have cited additional references within the Supporting Information.^[^ ^35^, ^36^, ^37^, ^38^, ^39^, ^40^, ^41^, ^42^, ^43^, ^44^, ^45^, ^46^ ^]^

## Conflict of Interests

A patent application protecting the class of compounds disclosed in this paper has been filed by C.L., D.N., and A.B. M.D. and H.K. are AstraZeneca employees and may own stocks or stock options. The remaining authors have no competing interests to declare.

## Supporting information



Supporting information

Supporting information

## Data Availability

Data supporting the findings of this study are available in the supporting information of this article. Structure factors and coordinate files of the eight X‐ray structures are deposited in the Protein Data Bank database (https://www.rcsb.org/).
