# Supplementary material for: Fragment‐Based Drug Discovery of Novel High‐affinity, Selective, and Anti‐inflammatory Inhibitors of the Keap1‐Nrf2 Protein‐Protein Interaction
Source: Angew Chem Int Ed Engl. 2025 Aug 21;64(39):e202508121. doi: 10.1002/anie.202508121 (PMC12455397; doi:10.1002/anie.202508121)
Supplement: Supplementary file 1 — Supporting information [file ANIE-64-e202508121-s002.pdf]

SUPPORTING INFORMATION

Contents

Supporting Tables 2

Table S1. PDB structures–X-ray crystallographic data and refinement statistics. 2
Table S2. Structures, Calculated Physicochemical Properties, and K_i values (FP assay) 4
Table S3. Transcriptome Data of Genes Related to Fibrosis, Inflammation, and ROS 11

Supporting Figures 12

Figure S1. SPR Sensorgrams of Fragment Hits 12
Figure S2. FP Data (Concentration−Response Curves) 13
Figure S3. cLogD and tPSA Distribution 14
Figure S4. Chiral HPLC of **24** and characterization of enantiomers 15
Figure S5. Selectivity Profiling 16
Figure S6. Cell Data (HaCaT, 10 µM) and Positive Control 17
Figure S7. IFN Signalling Following IFNβ Stimulation 18
Figure S8. Transcriptome Analysis 19

Experimental Methods 20

Expression and Purification of Human and Mouse Keap1 Kelch Domains 20
Crystallization, X-ray data collection, and Structure Determination 20
Molecular Docking 20
Surface Plasmon Resonance 20
Fluorescence Polarization Assay 21
Selectivity Panel 21
Cell Assay (HaCaT) 21
Cell culture and compound stimulation 21
Western Blotting 22
RT-qPCR 22
Cell Assay (BEAS-2B) 22
Anti-inflammatory Effects in THP-1 Macrophages 23
Cell culture and differentiation of THP-1 monocytes to macrophages 23
Inflammasome activation 23
ELISA of IL-1β release 23
Immunoblotting and antibodies 23
RNA Sequencing and Analysis 23
RNA isolation 23
Illumina RNA sequencing, quantification of differential gene expression, and pathway analysis 24
Cytotoxicity Assessment 24

Chemical Synthesis 25

Material and Methods 25
General Procedures 25
Synthesis of Novel Keap1-Nrf2 Inhibitors 27
Synthesis of fragment hit and analogues (**1**–**4**) 27
Synthesis of F2L analogues (**5**–**11**) 31
Synthesis of the cycloheptyl series (**12**–**28**) 37
Synthesis of the cyclohexyl series (**29**–**38**) 50
Synthesis of the cyclohex-2-enyl series (**39**–**48**) 57

NMR and LC-MS Spectra (UV250) of Final Compounds (1−48) 64

Supporting References 112

Supporting Tables

Table S1. PDB structures–X-ray crystallographic data and refinement statistics.[a]

Crystal data								
Compound	**1**	**3**	**7**	**23**	**24**	**29**	**33**	**39**
PDB ID	**9HWQ**	**9HWR**	**9HWS**	**9HWX**	**9HWT**	**9HWU**	**9HWW**	**9HWV**
Space group	*P 6₁*	*P 6₁*	*P 6₁*	*P 6₁*	*P 6₁*	*P 6₁*	*P 6₁*	*P 6₁*
Unit cell axes a, b, c (Å)	103.15, 103.15, 55.23	103.18, 103.18, 54.99	103.28, 103.28, 55.04	103.57, 103.57, 54.62	103.47, 103.47, 54.54	102.99, 102.99, 54.64	103.34, 103.34, 55.03	103.13, 103.13, 54.92
Unit cell axes α, β, γ (°)	90, 90, 120	90, 90, 120	90, 90, 120	90, 90, 120	90, 90, 120	90, 90, 120	90, 90, 120	90, 90, 120
Molecules in a.u.[a]	1	1	1	1	1	1	1	1
**Data collection**								
Beamline	DLS, I04-1	MAXIV, BioMAX	MAXIV, BioMAX	MAXIV, BioMAX	MAXIV, BioMAX	MAXIV, BioMAX	MAXIV, BioMAX	MAXIV, BioMAX
Wavelength (Å)	0.9159	0.9762	0.9762	0.9763	0.9763	0.9762	0.9763	0.9763
Resolution range (Å)	89.33-1.14 (1.18-1.14)[b]	44.68-1.20 (1.24-1.20)[b]	37.66-1.61 (1.67-1.61)[b]	34.66-1.74 (1.80-1.74)[b]	46.59-1.65 (1.71-1.65)[b]	44.6-1.40 (1.45-1.40)[b]	44.8-1.61 (1.67-1.61)[b]	44.66-1.50 (1.55-1.50)[b]
Total reflections	1,162,045 (96,255)[b]	1,168,927 (53,472)[b]	812,875 (43,765)[b]	710,763 (71,073)[b]	813,690 (81,275)[b]	1,338,296 (130,768)[b]	899,547 (88,990)[b]	1,094,669 (108,617)[b]
Unique reflections	121,734 (12,109)[b]	65,252 (6,287)[b]	43,209 (4096)[b]	34,446 (3431)[b]	40,112 (3935)[b]	64,632 (6365)[b]	43,298 (4278)[b]	53,387 (5303)[b]
Multiplicity	9.5 (7.9)[b]	17.9 (8.5)[b]	18.8 (10.6)[b]	20.6 (20.6)[b]	20.3 (20.7)[b]	20.7 (20.5)[b]	20.8 (20.8)[b]	20.5 (20.5)[b]
Completeness (%)	99.94 (99.67)[b]	99.53 (95.69)[b]	99.45 (95.01)[b]	99.93 (99.62)[b]	99.80 (98.50)[b]	99.18 (98.27)[b]	99.71 (98.98)[b]	99.92 (99.81)[b]
Mean I/σ(I)	12.23 (1.27)[b]	24.44 (2.86)[b]	22.28 (3.52)[b]	21.34 (1.24)[b]	18.86 (1.24)[b]	12.53 (1.18)[b]	14.81 (1.32)[b]	15.22 (1.35)[b]
Wilson B-factor (Å^2)	11.22	11.72	18.92	37.16	26.45	16.15	20.45	17.77
R_{pim} (%)	3.8 (85.4)[b]	2.5 (37.8)[b]	1.9 (19.3)[b]	1.7 (64.3)[b]	2.9 (81.9)[b]	3.2 (37.6)[b]	5.5 (51.3)[b]	3.8 (65.9)[b]
$CC_{1/2}$	0.99 (0.49)[b]	1 (0.8)[b]	0.99 (0.92)[b]	1.0 (0.67)[b]	0.99 (0.59)[b]	0.99 (0.75)[b]	0.99 (0.61)[b]	0.99 (0.55)[b]
**Refinement**								
Amino-acid residues built (chain A)	289	293	291	290	291	291	290	291
Compound	1	1	1	1	1	1	1	1
Sulfate	5	6	5	4	2	4	4	3
DMSO	9	16	8	5	3	6	5	5
Water	381	271	189	42	99	203	152	163
Cl	0	1	1	0	1	2	0	2
R_{work} (%)[c]	14.5	13.2	15.6	18.6	17.2	16.3	16.5	16.5
R_{free} (%)[c]	16.4	15.8	18.1	21.5	20.4	17.5	19.7	18.2
Average B values (Å^2) for:								
Amino acid residues (chain A)	16.3	15.9	23.8	52.5	39.1	22.5	26.5	24.8
Compound	19.0	13.8	34.9	68.6	62.3	26.8	47.8	48.9
Sulfate	19.6	22.4	28.4	57.4	43.2	27.8	35.3	27.9

DMSO	37.1	51.5	50.5	72.6	67.9	45.2	50.4	52.4
Water	30.3	27.5	31.9	45.5	39.9	30.6	31.9	31.1
Cl	-	46.2	62.6	-	58.0	40.7	-	45.3
RMSD bond length (Å)	0.007	0.008	0.009	0.011	0.010	0.009	0.009	0.009
RMSD bond angles (°)	1.02	1.08	1.09	1.14	1.08	1.12	1.07	1.10
Ramachandran favored (%)[d]	97.6	96.9	96.9	97.2	96.5	97.9	98.3	96.9
Ramachandran allowed (%)[d]	2.4	3.1	3.1	2.8	3.5	2.1	1.7	3.1
Ramachandran outliers (%)[d]	0	0	0	0	0	0	0	0
Rotamer outliers (%)	0	0	0	0	0.7	0	0	0
Clash score	4.0	4.0	3.0	3.0	4.0	4.0	4.0	4.0
ligand ID	B0A	A1IX2	A1IX3	A1IX1	A1IX4	A1IXY	A1IX0	A1IXZ

[a] a.u: asymmetric unit of the crystal

[b] Outershell values are shown in parentheses

[c] R_{free} is equivalent to R_{work}, but calculated with 5% of reflections omitted from the refinement process

[d] MolProbity statistics from phenix

Table S2. Structures, Calculated Physicochemical Properties, and K_i values (FP assay)

Cmpd	Structures	MW	tPSA (Å^2)[a]	cLogD[b]	FP (K_i/nM)[c]
**1**	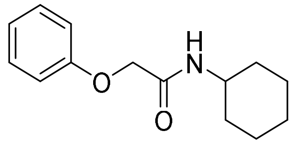	233.31	38.3	2.51	NA
**2**	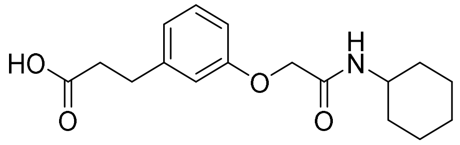	305.37	75.6	-0.58	NA
**3**	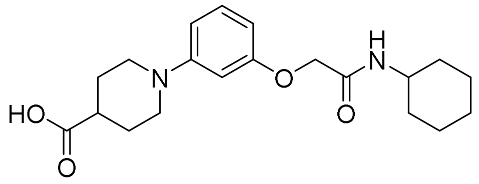	360.45	78.9	-0.32	162000 ± 22000
**4**	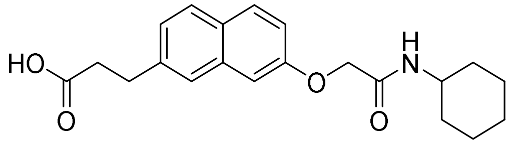	355.43	75.6	0.63	15500 ± 1300
**5**	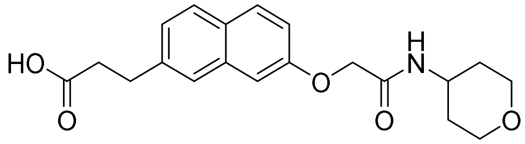	357.41	84.9	-1.21	38300 ± 2900
**6**	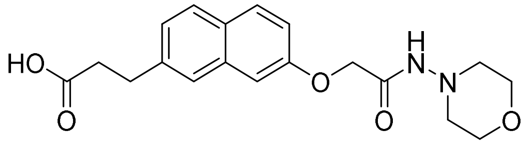	358.39	88.1	-1.75	268000 ± 29000
**7**	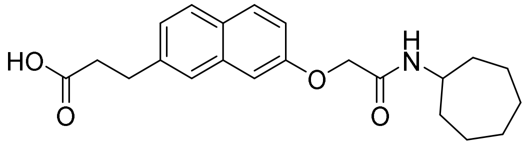	369.46	75.6	1.07	6560 ± 580
**8**	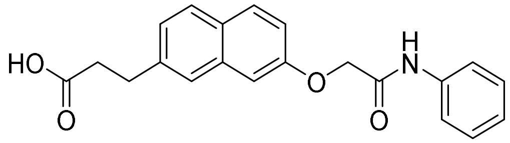	349.39	75.6	0.84	10200 ± 2400
**9**	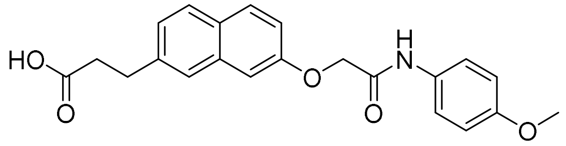	379.41	84.9	0.69	69600 ± 9400
**10**	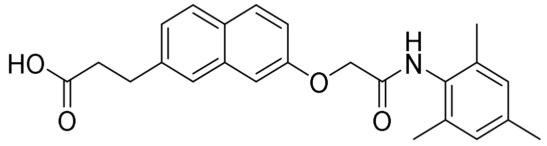	391.47	75.6	2.39	21300 ± 710
**11**	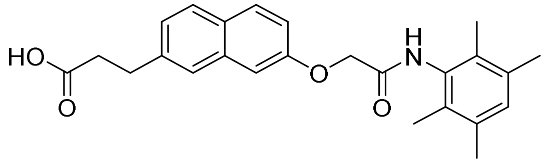	405.49	75.6	2.90	196000 ± 24000

**12**	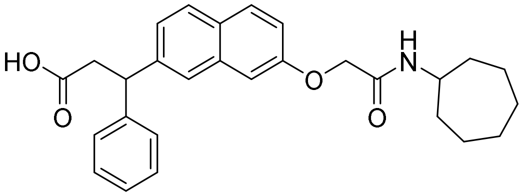	445.56	75.6	2.56	1060 ± 99
**13**	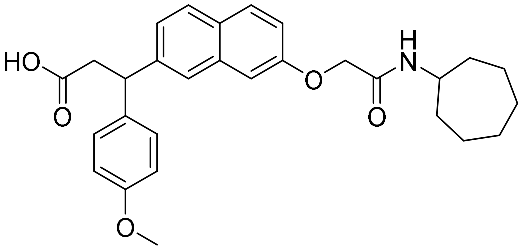	475.58	84.9	2.26	31.6 ± 2.6
**14**	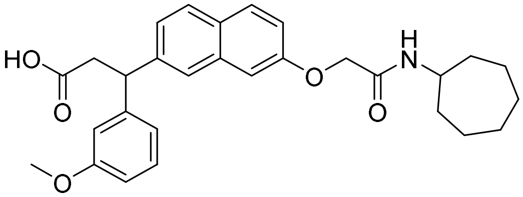	475.58	84.9	2.26	284 ± 31
**15**	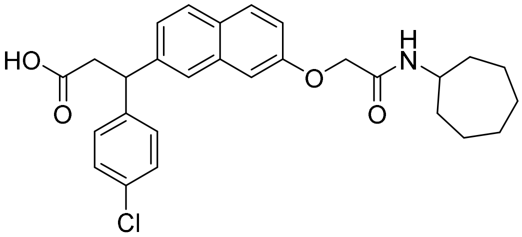	480.00	75.6	2.81	294 ± 25
**16**	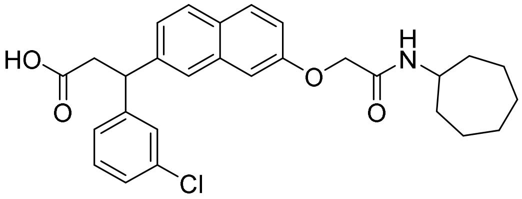	480.00	75.6	2.81	53.9 ± 6.1
**17**	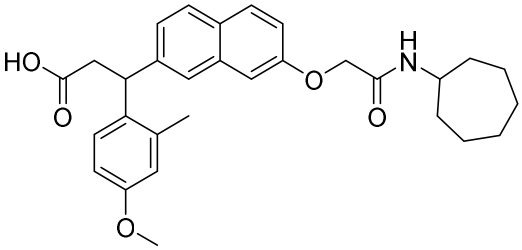	489.61	84.9	2.83	23.6 ± 2.2
**18**	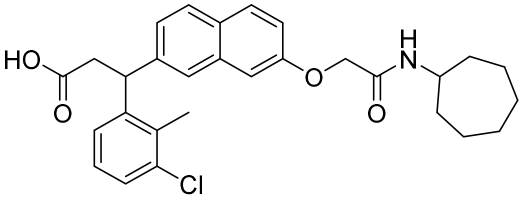	494.03	75.6	3.38	59.5 ± 8.4
**19**	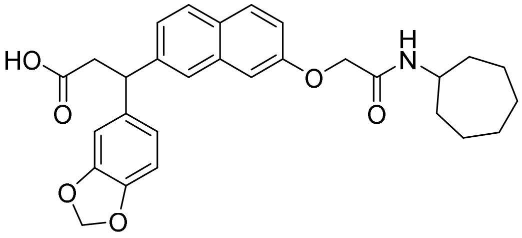	489.57	94.1	1.86	23.2 ± 1.3

**20**	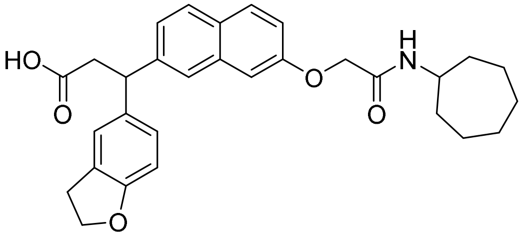	487.60	84.9	2.28	35.5 ± 3.4
**21**	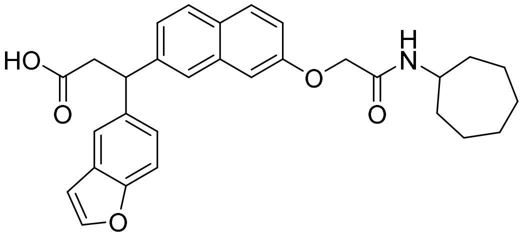	485.58	84.9	3.03	38.3 ± 1.6
**22**	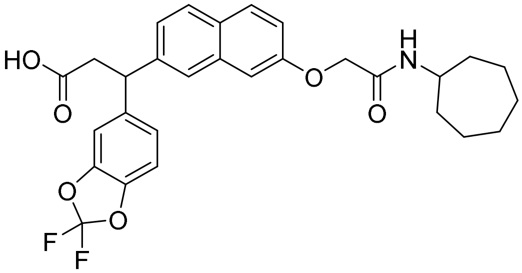	525.55	94.1	3.06	33.8 ± 4.7
**23**	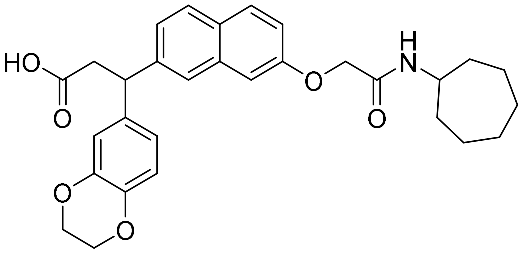	503.60	94.1	1.79	15.0 ± 2.5
**24**	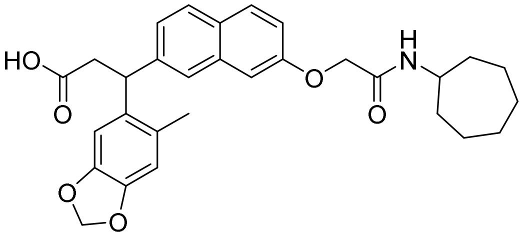	503.60	94.1	2.42	10.1 ± 1.4
**25**	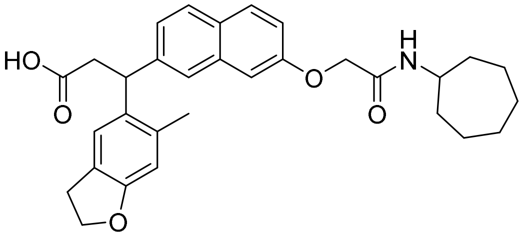	501.62	84.9	2.85	18.8 ± 1.7
**26**	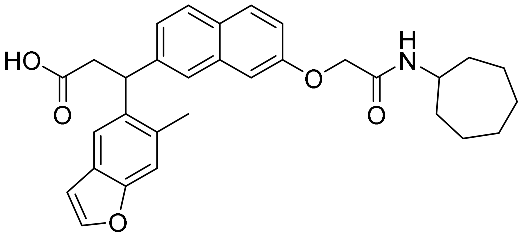	499.61	84.9	3.59	15.6 ± 2.6

**27**	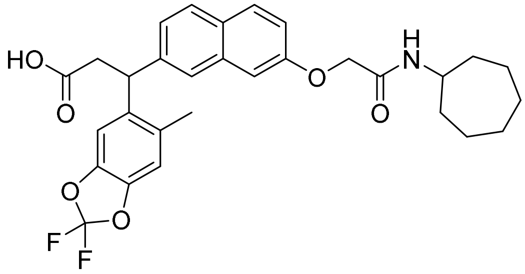	539.58	94.1	3.60	14.1 ± 2.4
**28**	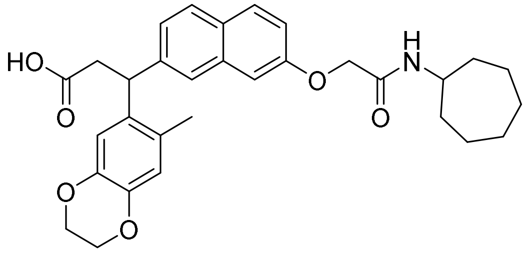	517.62	94.1	2.35	8.8 ± 0.8
**29**	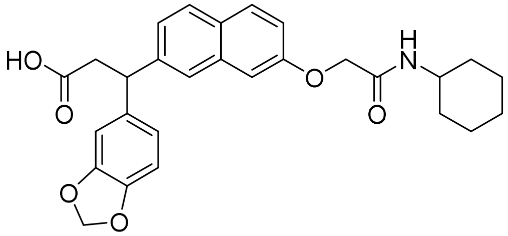	475.54	94.1	1.42	57.1 ± 4.8
**30**	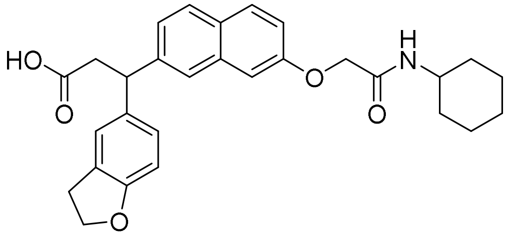	473.57	84.9	1.84	82.1 ± 7.9
**31**	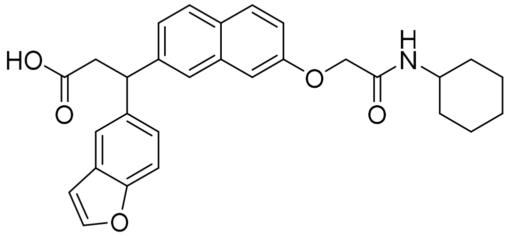	471.55	84.9	2.58	68.2 ± 12
**32**	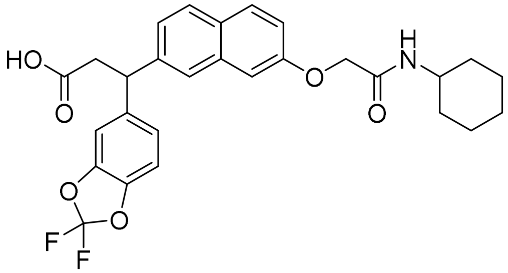	511.52	94.1	2.62	51.4 ± 4.8
**33**	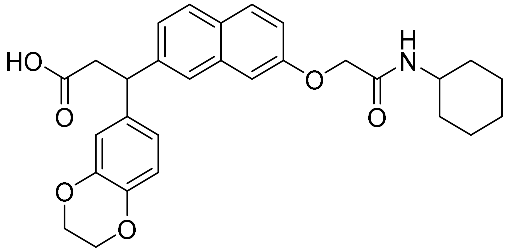	489.57	94.1	1.35	29.3 ± 1.7

**34**	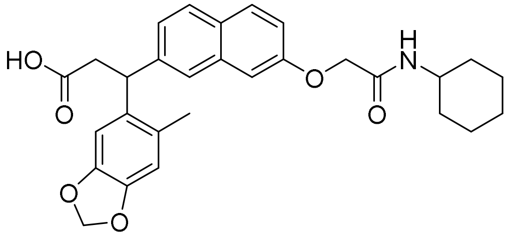	489.57	94.1	1.97	18.1 ± 5.9
**35**	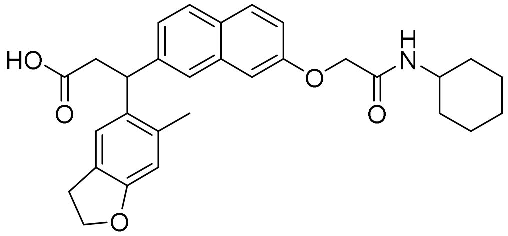	487.60	84.9	2.40	32.4 ± 1.3
**36**	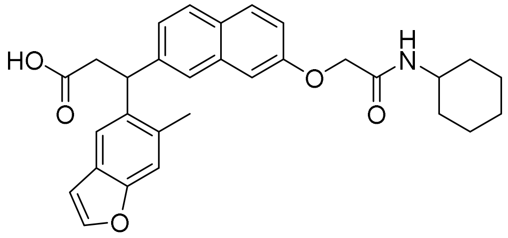	485.58	84.9	3.15	24.4 ± 0.4
**37**	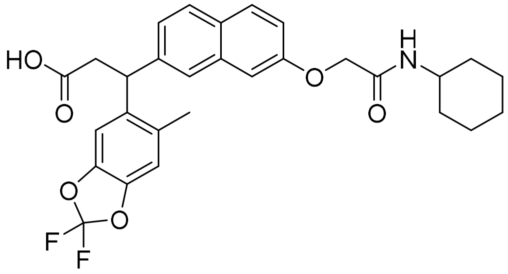	525.55	94.1	3.15	7.1 ± 1.0
**38**	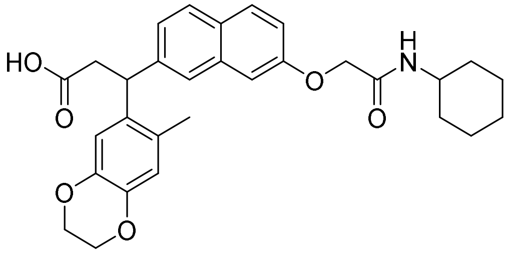	503.60	94.1	1.91	14.9 ± 0.8
**39**	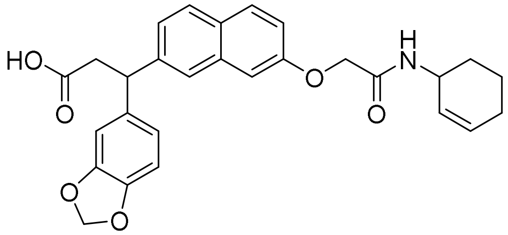	473.53	94.1	1.21	24.5 ± 1.7
**40**	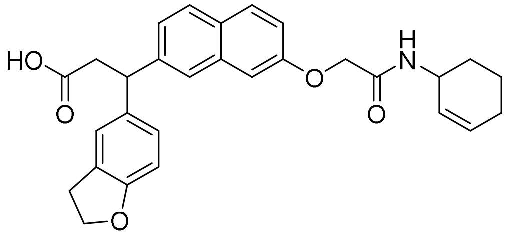	471.55	84.9	1.63	26.7 ± 4.2

**41**	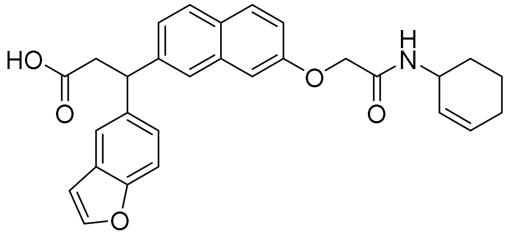	469.54	84.9	2.37	21.1 ± 1.5
**42**	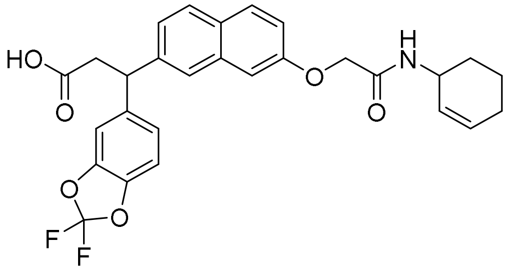	509.51	94.1	2.41	29.9 ± 2.2
**43**	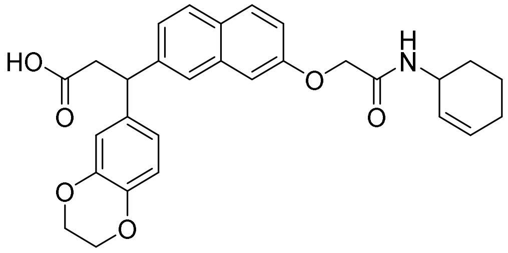	487.55	94.1	1.14	20.3 ± 2.1
**44**	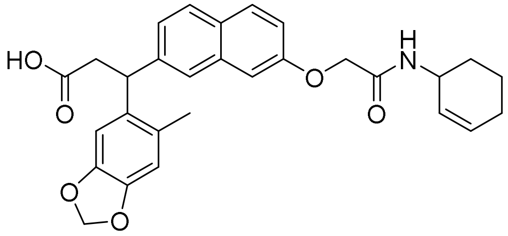	487.55	94.1	1.77	3.9 ± 0.8
**45**	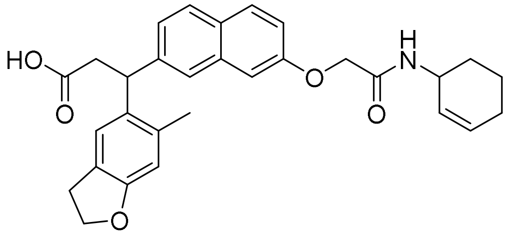	485.58	84.9	2.20	18.3 ± 2.4
**46**	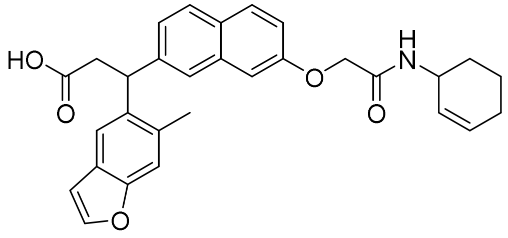	483.56	84.9	2.94	17.8 ± 3.4
**47**	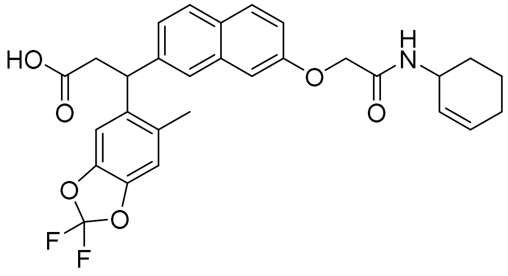	523.53	94.1	2.95	16.1 ± 3.8

**48**	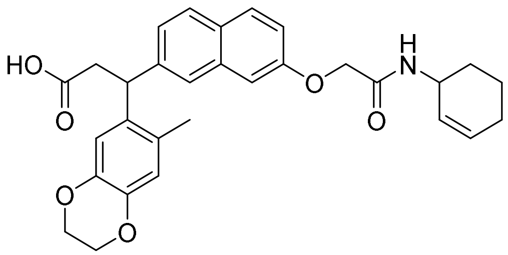	501.58	94.1	1.70	19.2 ± 2.4

[a] ChemDraw version 21.0.0.28 was used for determining tPSA.
[b] cLogD values at pH 7.4 using ChemAxon's MarvinSketch version 23.2.0.
[c] K_i values determined by FP are shown as mean ± SEM based on ≥ 3 individual measurements. NA: Not active (measured up to 4 mM)

Table S3. Transcriptome Data of Genes Related to Fibrosis, Inflammation, and ROS[a]

		24		28		KI-696		CDDO-Me		DMF		SFN	
**Pathway**	Gene Name	**FC**	**p-adj**	**FC**	**p-adj**	**FC**	**p-adj**	**FC**	**p-adj**	**FC**	**p-adj**	**FC**	**p-adj**
**Fibrosis**	COL1A1	1,40	NS	1,40	NS	1,59	NS	**3,46**	***	**0,44**	***	0,98	NS
	COL4A1	1,16	NS	**1,15**	**	0,61	NS	**0,30**	***	**1,46**	***	**0,55**	***
	COL4A2	1,10	NS	1,11	NS	0,77	NS	**0,49**	***	**1,16**	**	**1,38**	***
	**MMP9**	**0,91**	**	1,03	NS	1,21	NS	**0,51**	***	**0,85**	***	**0,70**	***
	**MMP12**	**0,51**	***	**0,53**	***	0,60	NS	**0,25**	***	**1,88**	***	**0,29**	***
	MMP8	0,79	NS	0,94	NS	0,82	NS	**0,21**	***	**1,70**	***	**0,32**	***
	LAMB1	**5,65**	***	**4,94**	***	2,64	NS	**3,83**	***	**4,44**	***	**7,81**	***
	LAMB4	**4,43**	*	3,01	NS	3,38	NS	**5,61**	**	**7,56**	***	**17,37**	***
**Inflammation**	**IFNGR1**	**0,63**	***	**0,64**	***	0,70	NS	**0,81**	***	0,93	NS	**0,63**	***
	**IFNGR2**	**0,73**	***	**0,76**	***	0,91	NS	**0,41**	***	**0,78**	***	**0,58**	***
	**IFNAR1**	0,93	NS	**0,92**	**NS**	0,90	NS	**0,82**	***	0,95	NS	**0,88**	**
	IFNAR2	1,00	NS	0,99	NS	1,02	NS	**0,52**	***	0,80	NS	0,85	NS
	**IL1B**	**0,85**	***	**0,88**	***	0,85	NS	**0,13**	***	**2,70**	***	**0,36**	***
	**TRAP1**	**0,82**	***	**0,86**	**	1,02	NS	**0,64**	***	**0,85**	**	**0,74**	***
	TNFRSF1B	1,08	NS	**1,14**	***	1,13	NS	**0,27**	***	**1,26**	***	**1,19**	***
	cGAS	**1,36**	**	**1,29**	*	1,08	NS	1,19	NS	1,04	NS	**1,42**	***
	**STAT1**	**0,82**	***	**0,77**	***	0,69	NS	1,02	NS	**0,62**	***	**0,66**	***
	**TBK1**	**0,86**	*	1,01	NS	1,56	NS	1,06	NS	**1,19**	**	0,95	NS
	IRF3	0,96	NS	1,03	NS	1,43	NS	0,95	NS	**1,15**	**	**1,48**	***
	**NLRP3**	**0,79**	***	**0,79**	***	0,78	NS	**0,43**	***	**1,19**	***	**0,44**	***
	**CASP1**	**0,65**	*	**0,59**	**	0,94	NS	1,31	NS	**0,18**	***	**0,45**	***
	NOD2	0,35	NS	0,38	NS	0,57	NS	0,78	NS	**0,31**	*	**0,16**	***
	TLR4	1,17	NS	1,04	NS	**0,32**	***	**0,26**	***	**0,84**	*	**0,43**	***
	**SOCS1**	**2,11**	***	**1,95**	***	**2,43**	**	**0,37**	***	**5,21**	***	**2,19**	***
	**SOCS3**	**2,26**	***	**2,13**	**	1,66	NS	1,14	NS	**18,01**	***	0,99	NS
**ROS production**	CYP1B1	**1,14**	**	1,04	NS	0,76	NS	**1,72**	***	**1,37**	***	**1,15**	**
	GPX1	**0,60**	***	**0,62**	***	1,38	NS	**0,44**	***	**0,59**	***	**0,53**	***
	**GPX4**	**1,13**	**	**1,20**	***	1,74	NS	0,91	NS	1,09	NS	0,94	NS
	**SOD1**	**0,41**	***	**1,35**	***	1,75	NS	**2,71**	***	**1,73**	***	**2,16**	***
	SOD2	**0,59**	***	**0,68**	***	0,87	NS	0,36	NS	0,19	NS	**0,40**	***
	**NOXA1**	**0,41**	***	**0,50**	***	0,92	NS	**0,67**	**	**0,30**	***	**0,45**	***
	AKR1B10	6,62	NS	4,27	NS	26,39	NS	**61,69**	**	1,00	NS	**34,78**	**

[a]FC = Fold change; NS = Not significant. P-values: *$p < 0.05$, **$p < 0.01$, ***$p < 0.001$. Statistical significant data is highlighted in bold. Gene names in bold if favourably regulated by compound **24**/**28**.

Supporting Figures

Figure S1. SPR Sensorgrams of Fragment Hits

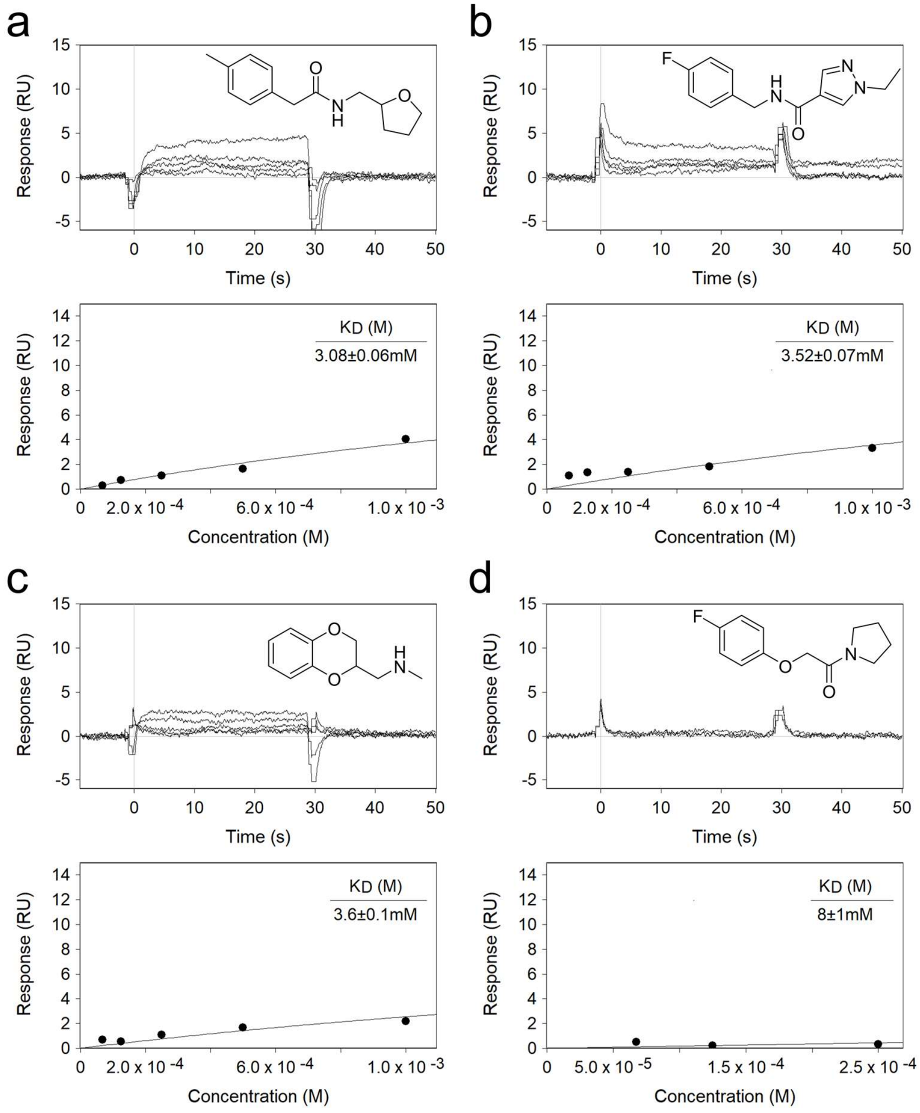

Figure S1. SPR sensorgrams of the four other P3/P5 subpocket-binding fragment hits (besides **1**), which displayed the same binding mode in molecular docking as in X-ray crystallography, and plots of equilibrium binding responses against the injected concentrations below. The fragments were injected in 2-fold serial dilutions (63 µM – 1 mM) over immobilized Keap1 Kelch. Signals from the two highest concentrations for the fragment in figure d were removed because of extremely high responses (>1000 RU), likely due to unspecific binding to the chip. The K_d values were produced by the program after fixing R_{max} to the theoretical maximum values (12–16 RU) of the four fragments based on the immobilization level (2145 RU).

Figure S2. FP Data (Concentration−Response Curves)

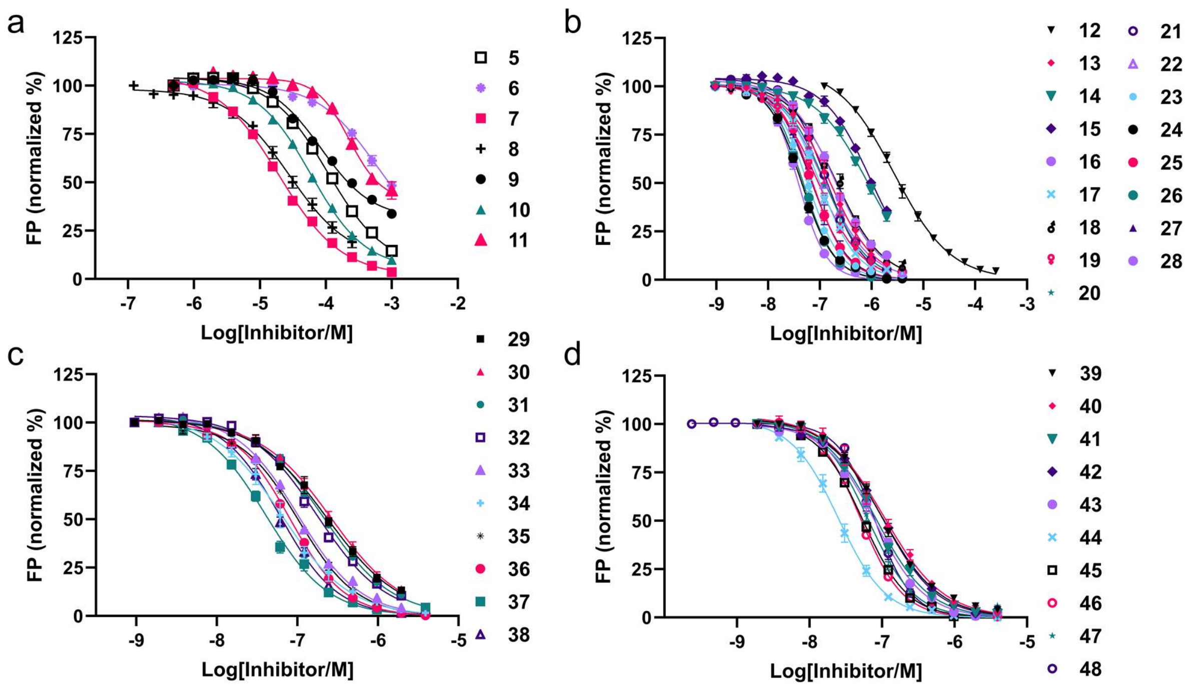

Figure S2. Representative FP data for compounds **5**−**11** (a), **12**−**28** (b, cycloheptyl subseries), **29**−**38** (c, cyclohexyl subseries), and **39**−**48** (d, cyclohex-2-enyl subseries) illustrating the affinity-improvements during the structure-guided F2L process.

Figure S3. cLogD and tPSA Distribution

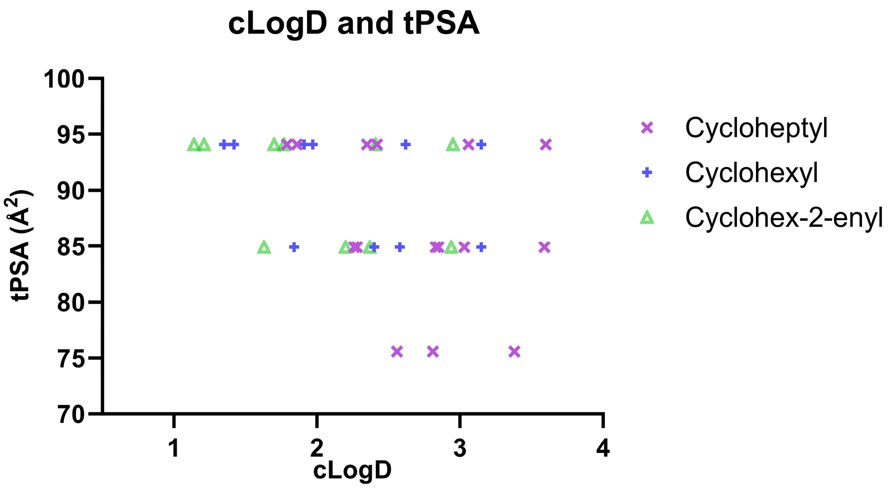

Figure S3. Mapping of cLogD and tPSA properties of compounds **12–48** demonstrating a broad coverage across the three subseries. Values are also listed in **Table S2**.

Figure S4. Chiral HPLC of 24 and characterization of enantiomers

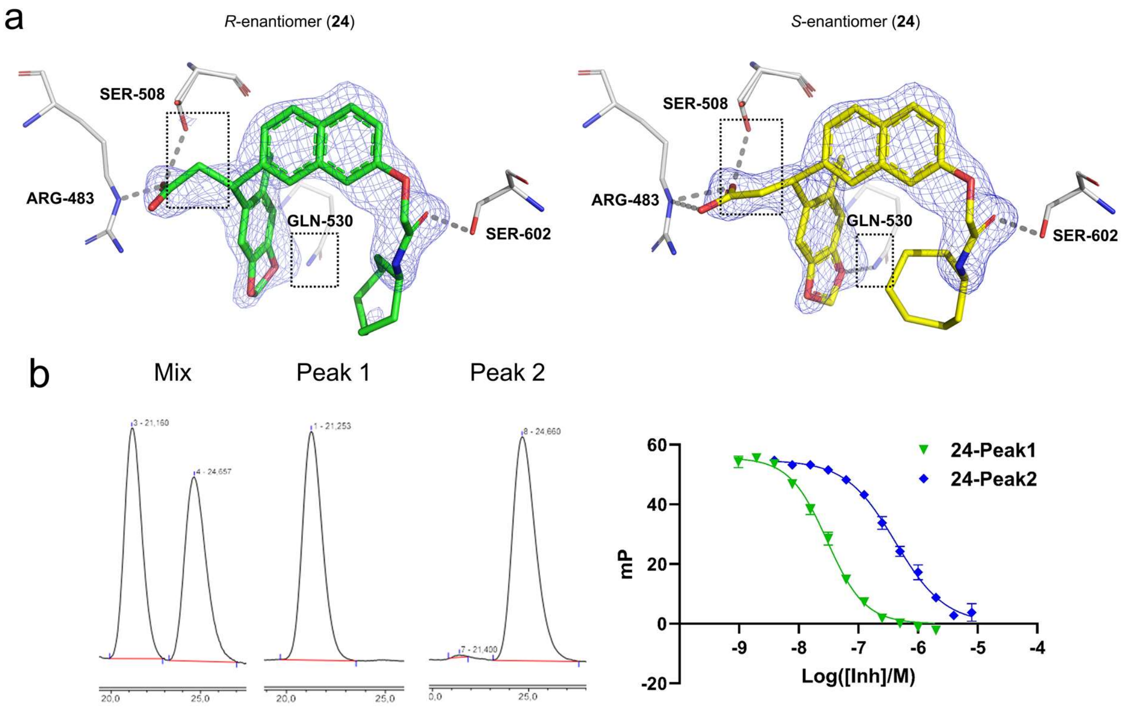

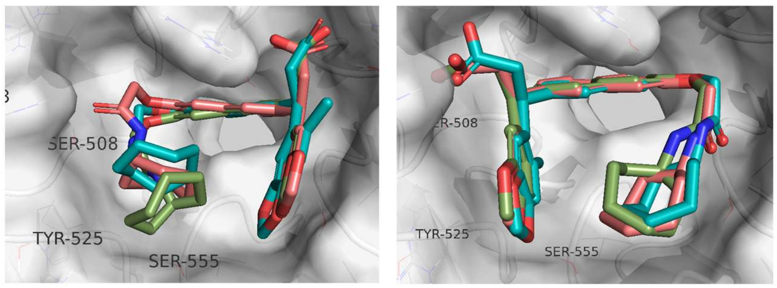

Glide Gscore (kcal/mol)	
R-**24**	Pose 1: -6.963
	Pose 2: -6.689
	Pose 3: -6.668
S-**24**	Pose 1: -8.235
	Pose 2: -8.179
	Pose 3: -7.916

Figure S4. a) Investigations of chirality of compound **24** in the Keap1/**24** costructure (PDB ID: 9HWT). Based on visual evaluation, the *S*-enantiomer is concluded to fit better to the electron density than the *R*-enantiomer. For *R*-**24**, the methylene next to the carboxylic acid group moves away from the electron density, and it does not form the hydrogen bond interaction to Gln-530 as seen for *S*-**24** (stipulated boxes). b) Chiral separation of compound **24** into enantiomers was performed using a preparative Chiralcel OD column. 10 mg of **24** was solubilized in EtOH:heptane 50:50 v/v% to a concentration of 1 mg/mL and loaded on the column over four runs. The two enantiomers were separated by isocratic elution using EtOH:heptane:TFA 10:90:0.1 v/v% resulting in two peaks with enantiomeric excess of 99% (Peak 1) and 98.8% (Peak 2; here, the other enantiomer ended up being present at a level of 0.6%). The fractions of the enantiomers were evaporated, solubilized in MeCN:H_2O 1:1 v/v% and freeze-dried yielding 2.5 mg Peak/enantiomer 1 and 1.6 mg Peak/enantiomer 2. The powders were solubilized in DMSO for characterization by LC-MS and FP and in MeCN:H_2O for characterization by chiral HPLC using an analytical Chiralcel OD column (chromatograms shown in figure). Testing the two enantiomers by FP revealed a 14-fold higher affinity of Peak 1 (K_i = 7.9 ± 1 nM) compared to Peak 2 (K_i = 114 ± 13 nM) (n ≥ 3). c) Molecular docking of the *R* (left) and *S* (right) enantiomers of **24** into the Keap1/**24** costructure (PDB ID: 9HWT) indicates that the high-affinity enantiomer is *S*. Its top 3 highest-scoring docking poses show less variability and have better scores compared to the *R*-enantiomer. Also, *R*-**24** show a reverted binding mode relative to the Keap1/**24** costructure. Hence, based on X-ray crystallography data, FP, and docking it is concluded that Peak 1 represents the high-affinity *S*-enantiomer and Peak 2 the *R*-enantiomer of **24**.

Figure S5. Selectivity Profiling

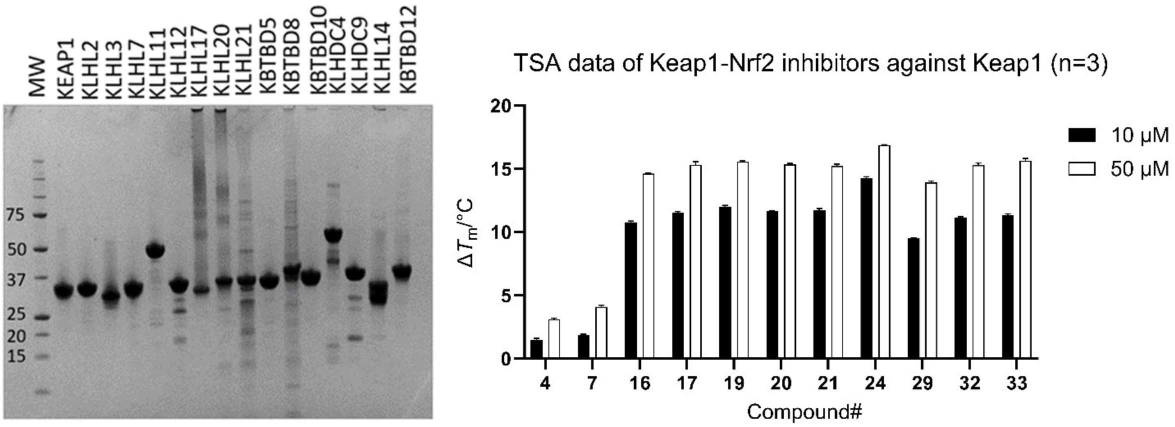

Figure S5. SDS-PAGE of the 16 Kelch domains and Keap1 TSA data. The 16 human Kelch domains used in the selectivity profiling of the Keap1-Nrf2 inhibitors were expressed in *E. coli* or Sf9 insect cells and purified by standard protein chromatographic methods. Their purity is shown by SDS-PAGE (left). ΔT_m (mean ± SEM) of Keap1 Kelch treated with the different compounds at 10 or 50 μM.

Figure S6. Cell Data (HaCaT, 10 μM) and Positive Control

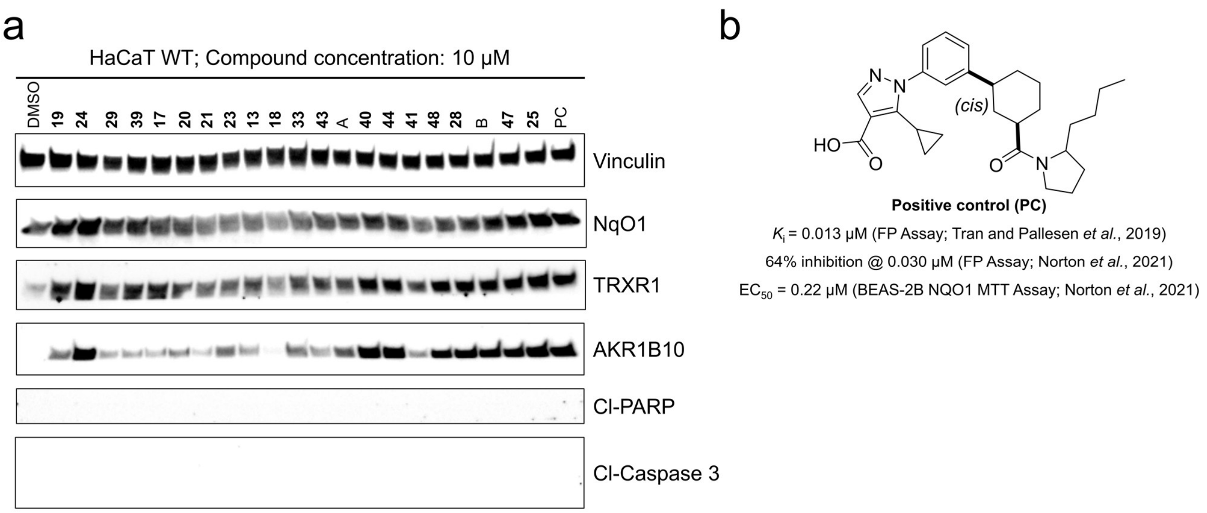

Figure S6. a) Protein levels from whole cell lysates assessed by WB after treating HaCaT cells with 19 high-affinity noncovalent Keap1-Nrf2 inhibitors and the positive control (PC) at 10 μM for 24 h. A representative blot from two independent experiments is shown. Compounds A and B are not disclosed in this study, as they are not relevant to its scope. b) The structure of the positive control, along with affinity (FP assay) and cell potency (BEAS-2B assay) data from literature.[1-2]

Figure S7. IFN Signalling Following IFNβ Stimulation

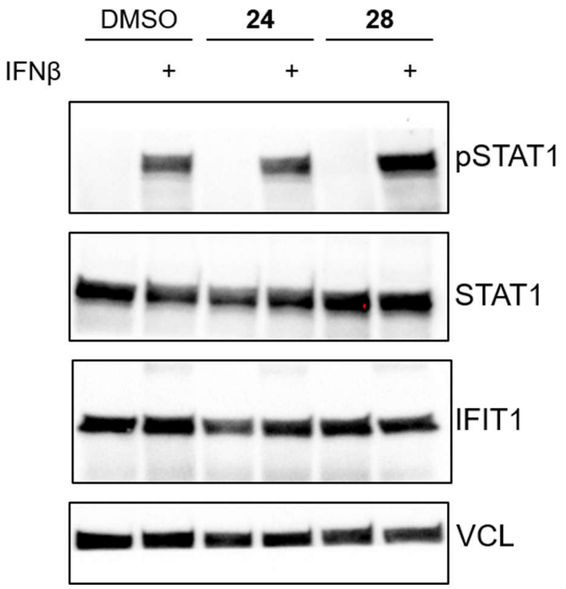

Figure S7. Compounds **24** and **28** did not affect IFN signalling following IFNβ stimulation. Macrophage-derived THP-1 cells were treated with **24** and **28** (100 μM) or DMSO for 24 h. Type I IFN pathway was induced with IFNβ (500 U/mL, 1 h), and cell lysates were analysed by WB for the indicated proteins.

Figure S8. Transcriptome Analysis

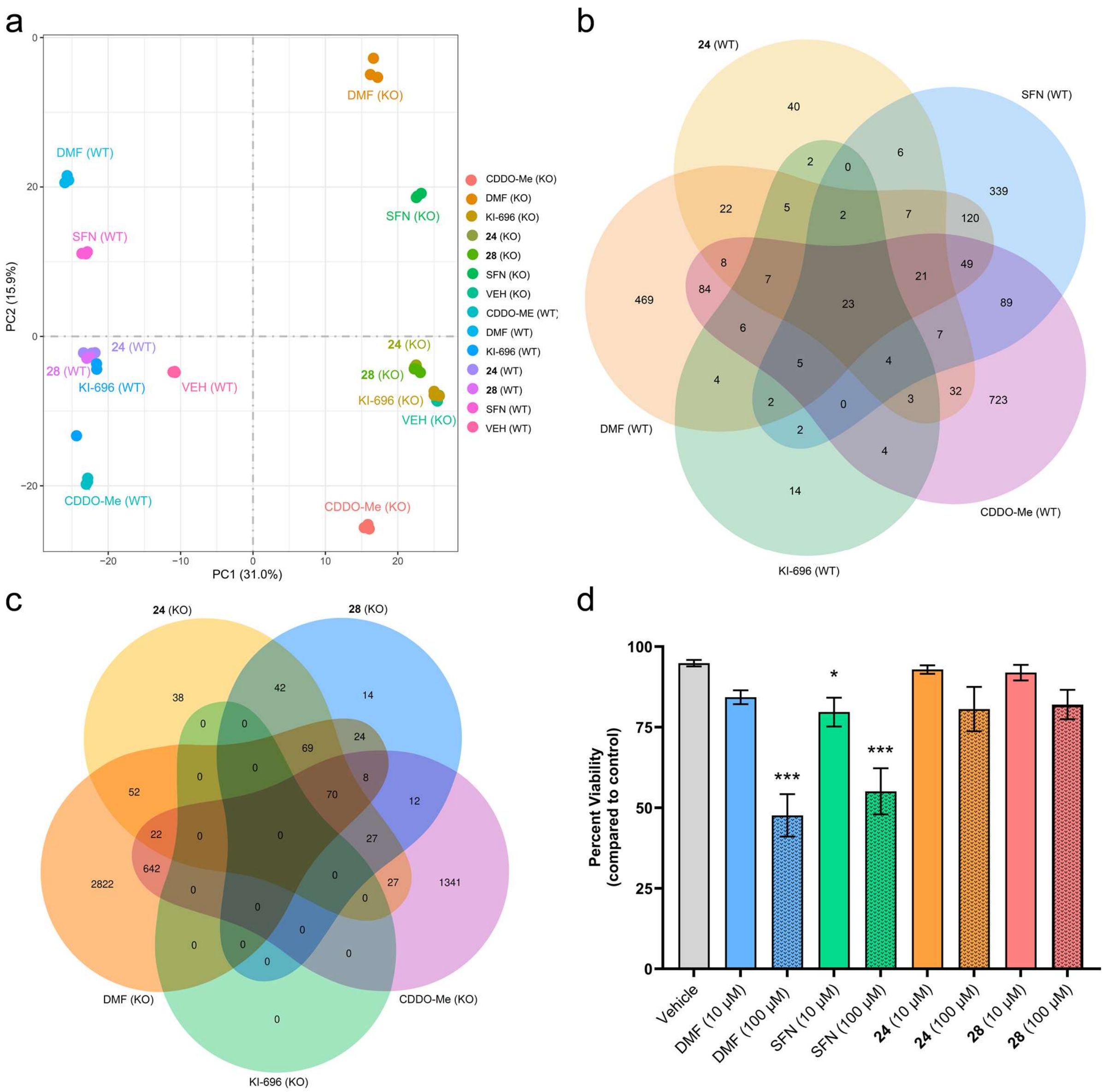

Figure S8. Transcriptome analysis of THP1 cells (WT and Nrf2 KO) differentiated into macrophages with PMA and treated with covalent Nrf2 activators (CDDO-Me at 0.1 µM; SFN and DMF at 10 µM) and noncovalent Keap1-Nrf2 inhibitors (KI-696 at 1 µM; **24** and **28** at 10 µM) for 12 hours (n = 3 technical replicates). a) PCA plot illustrating the RNA expression profiles of the various treatment groups in WT cells. b) Venn diagram of upregulated genes (FC > 2.0; p-adj < 0.05) in WT cells. The data are the same as shown in figure 7a, but with the SFN treated group replacing that of compound **28**. c) Differentially expressed genes (up- and downregulated; FC > 2 or < 2; p-adj < 0.05) in Nrf2 KO cells. d) Cytotoxicity measured by exposing THP1 cells to 10 or 100 µM compound for 48 hours and measuring the viability by quantifying propidium iodide exclusion through flow cytometry. Data presented as mean ± SEM (n > 3; asterisks indicate significance values of the difference to control cells: *p < 0.05, ***p < 0.001; One-way ANOVA).

Experimental Methods

Expression and Purification of Human and Mouse Keap1 Kelch Domains

The Keap1 Kelch domains were expressed and purified as previously described.[1, 3-5] In short, the his-tagged human Kelch domain (residue 321–609, UniProt Q14145) was cloned into a pRSET A vector and expressed in *Escherichia coli* BL21 (DE3) pLysS. Protein was purified by column chromatography, following established procedures.[1] The His-tagged mouse Kelch domain (residue 322–624, UniProt Q9Z2X8) was also cloned into a pRSET A vector and expressed in *Escherichia coli* BL21 (DE3) pLysS. Purification was performed by column chromatography, as detailed previously.[3]

Crystallization, X-ray data collection, and Structure Determination

Crystals of the mouse Keap1 Kelch domain protein were obtained as described previously.[3-5] For obtaining protein−fragment/compound complexes, crystals of mouse Keap1 Kelch domain were soaked for 2.5 hours (50 mM; 10% DMSO) with fragments or 2–16 hours (0.5–10 mM; 10% DMSO) with compounds, in a solution of 0.1 M Na citrate pH 5.6, 0.5–0.6 M ammonium sulphate, 0.9–1.1 M lithium sulphate, and then harvested in liquid nitrogen for X-ray diffraction. Data for mouse Keap1 Kelch domain in complex with fragments and larger compounds were collected from the synchrotron beamline facilities Diamond Light Source[6], Oxfordshire and BioMAX at MAXIV[7], Lund, respectively. In total, 768 fragments from the DSI-poised library[8] were tested by crystallographic fragment screening against the mouse Keap1 Kelch domain at the XChem platform of Diamond Light Source. Diffraction images were integrated, scaled, and merged using autoprocessed beamline tools, while in some cases the data were reprocessed and scaled using XDS.[9] The structures were solved using PHASER[10] using PDB ID 6ZF4[3] as the search model for molecular replacement. Restraints for the fragments/compounds were prepared using the AceDRG[11] and Phenix.elbow[12] followed by Model building and refinement using COOT[13] and Phenix.refine.[14] Figures were prepared using PyMOL (The PyMOL Molecular Graphics System, Version 2.5.2 Schrödinger, LLC).[15]

Molecular Docking

In silico experiments were performed using Schrödinger's Maestro software (version 13.2).[16] The protein from the PDB ID 5FNU[17] X-ray structure was used as receptor grid while retaining the five conserved waters at the opening of the central Kelch domain channel, as previously described.[3-4] Ligands were prepared by LigPrep with default settings. Ligand docking and scoring were performed using Glide with default settings and with the XP (extra precision) scoring function. PyMOL was applied for analyses and visualization of docking poses.[15]

Surface Plasmon Resonance

SPR measurements were performed at 25 °C using a Pioneer FE FE instrument (Sartorius), as described previosuly.[1, 3-4] The Keap1 Kelch domain was covalently immobilized on the biosensor chip surface by amine coupling to a level of 2150 RU, using a 10 mM NaOAc pH 5 immobilization buffer. The 1×HBSTET buffer (10 mM Hepes, 150 mM NaCl, 0.005% Tween20, 3 mM EDTA, 1 mM TCEP, pH = 7.4) supplemented with 4% DMSO was used as running buffer for the experiments. Microcalibration was performed for all SPR experiments to adjust for DMSO bulk effects (low limit of 3% and a high limit of 5%). The compound was injected in a concentration series of twofold serial dilutions (67.5–1000 µM) using standard injections at a 30 µL/min flow rate. The data were analyzed using Qdat Data Analysis Tool version 2.6.3.0 (Sartorius). The sensorgrams were corrected for buffer effects and unspecific binding to the chip matrix by subtraction of blank and reference surfaces (a blank flow cell channel activated by the injection of EDC/NHS and inactivated by the injection of ethanolamine). The

dissociation constant (K_d) was estimated by plotting MW normalized response levels (RU_{norm}) at equilibrium (R_{eq}) against the injected concentration and curve-fitted to a Langmuir (1:1) binding isotherm or by kinetic global fit of the SPR sensorgram to a simple 1:1 interaction model, while $R_{norm,max}$ was fixed to the theoretical possible maximum (12–20 RU dependent on fragment) normalized R_{max}.

Fluorescence Polarization Assay

The assay was conducted with Cy5-Nrf2 (Cy5-LDEETGEFL-NH_2) as the fluorescently labelled peptide probe (3 nM) and human Keap1 Kelch at 14 nM in the 1×HBSTET assay buffer, as previously.[1, 3-5] In short, the assay was performed in black flat-bottom 384-well plates (Corning Life Sciences, NY) allowing an assay volume of 30 µL/well. Proper mixing and elimination of air bubbles was ascertained by spinning down the plates and incubation for 10–15 minutes at RT before measuring FP levels on a Safire2 plate-reader (Tecan, Männedorf, Switzerland). The K_i values of the compounds were determined by dose-response experiments (12-points, 4% DMSO in all wells), where FP values were fitted to the equation $Y = Bottom + (Top - Bottom)/[1 + (10^{HillSlope*(LogIC50-X)})]$; X representing the logarithmic value of compound concentration. The resulting IC_{50} value, together with the K_d value (the dissociation constant between probe and Keap1 Kelch, determined in a prior experiment) and Keap1 Kelch concentrations, was used to calculate the theoretical competitive inhibition constant, the K_i value.[18]

Selectivity Panel

Melting curves of Kelch domains (Keap1, KLHL2, KLHL3, KLHL7, KLHL11, KLHL12, KLHL17, KLHL20, KLHL21, KBTBD5, KBTBD8, KBTBD10, KLHDC4, KLHDC9, KLHL14, KBTBD12) with and without the presence of compounds were determined by thermal shift assay (TSA) using the Sypro Orange dye (Life Technologies), a Stratagene Mx3005P RT-PCR apparatus (Agilent Technologies, Waldbronn, Germany), and white non-skirted 96-well PCR-plates, as previously described.[5, 19] The compounds were tested at 10 and 50 µM (4% DMSO) in the presence of protein (final concentration: 2 µM) and Sypro Orange (final concentration: 5×) using TSA assay buffer (10 mM HEPES, pH = 7.4, 150 mM NaCl, 1 mM TCEP) and final sample volume of 20 µL/well. The plates were sealed and spun-down for 1 minutes at 500 *g*, incubated for 10 min at RT, and measured from 25–95 °C in 70 cycles with a 1 °C temperature increase per 21 seconds and fluorescence intensities measured at each cycle (excitation and emission set to 465 and 590 nm, respectively). The sigmoidal plot of the normalized fluorescence intensity values versus temperature was fitted to the Boltzmann equation $Y = Bottom + (Top-Bottom)/(1+exp((T_m-X)/Slope))$, where X is temperature in °C, whereby the melting temperature (T_m), where 50% of protein is denatured, was determined. The difference in T_m (ΔT_m) for protein with and without (DMSO blank) presence of compounds was used as output parameter for the compounds.

Cell Assay (HaCaT)

The HaCaT cell assay was done as previously described,[5] with a few minor variations as detailed below.

Cell culture and compound stimulation

WT, pLenti control and Nrf2-KO HaCaT human keratinocytes were maintained at 37°C with 5% CO_2. All cell lines were cultured in Complete DMEM (Gibco) (supplemented with 10% heat inactivated fetal calf serum (Sigma-Aldrich), 1000 U/mL penicillin, 1000 µg/mL streptomycin, and 2 mM L-glutamine (Gibco)) and split in regular intervals using trypsin. Prior to compound stimulation, cells were counted and seeded in 24-well plates for subsequent RT-qPCR tests or 12-well plates for a subsequent immunoblotting setup. For RT-qPCR, approximately 100.000–150.000 HaCaT WT/pLenti cells and 200.000–250.000 Nrf2-KO HaCaT cells were added per well, whereas for immunoblotting approximately 250.000–300.000 HaCaT WT/pLenti cells and 400.000–450.000 Nrf2-KO HaCaT cells were added per well. Cells used in both Western Blotting and

RT-qPCR were stimulated with compounds or DMSO diluted in DMEM and incubated for 24 h. DMSO was added accordingly to highest percentage compound volume added.

Western Blotting

Upon 24 hour compound stimulation, the cells were trypsinized, washed, spun down and resuspended in 70 µL of ice-cold Pierce RIPA lysis buffer (Thermo Scientific) supplemented with 10 mM NaF, 1× complete protease cocktail inhibitor (Roche), and 5 IU mL-1 benzonase (Sigma), respectively. The protein concentrations were determined using the BCA protein assay kit (Thermo Scientific) and samples was equalized accordingly to the sample of lowest protein concentration. 1×XT sample buffer (Bio-Rad) and 1×XT reducing agent (Bio-Rad) were added to the samples, and lysates were denatured by boiling for 5 min at 95 °C. Denatured samples was separated by SDS-PAGE on a 4–20% Criterion TGX precast gradient gel (Bio-Rad). The gel ran initially for 20 min at 70 V and 45 min at 120 V and was then transferred onto a PVDF membrane (Bio-Rad) using a Trans-Blot Turbo transfer system. Membranes were blocked in 5% skim milk (Sigma-Aldrich) in PBS supplemented with 0.05% Tween-20 (PBST) for 1 h at room temperature on a shaker. Membranes were fractionated accordingly to the sizes of investigated proteins and probed with antibodies overnight at 4 °C on a shaker. Following primary antibodies were used in PBS Tween 0.05%: anti-NqO1 (Cell Signaling, 1/1000), anti-TRXR1 (Cell Signaling, 1/1000), anti-Cl-Caspase-3 (Cell Signaling, 1/1000), anti-Cl-PARP (Cell Signaling, 1/1000), anti-AKR1B10 (Santa Cruz, 1/1000), and anti-Vinculin (VCL; Sigma-Aldrich 1/10 000) used as loading control. The membranes were washed three times 10 min in PBST and then incubated for 1 h at room temperature in secondary antibodies, peroxidase-conjugated F(ab)2 donkey anti-mouse IgG (H + L) (1:10 000) or peroxidase-conjugated F(ab)2 donkey anti-rabbit IgG (H + L) (1:10 000) (Jackson ImmunoResearch), in PBST 1% milk. Membranes were washed three times 10 min and exposed using the SuperSignal West PicoPLUS chemiluminescent substrate and the iBrightTM Imaging System Model No. CL1500.

RT-qPCR

Upon 5 hour compound stimulation, RNA was extracted from the cells using the High Pure RNA Isolation Kit (Roche, 11828665001) according to the manufacturer's instructions. RNA was eluted in 60 µL of sterile RNase-free water. The quality and purity of the RNAs were assessed by Nanodrop spectrometry (DeNovix DS-11). Using the TaqMan detection systems, the gene expression of HO-1 (HMOX-1), NQO1, AKR1B10, and TRXR1 was determined by real-time PCR. In accordance with the manufacturer's recommendations (Applied Biosciences) the premade TaqMan assays and RNA-to-Ct-1-Step kit was used to analyze the RNA levels. The commercially available TaqMan assay (Thermo Fischer Scientific, cat. no. 4392938) was used, and samples were analyzed in a final volume of 10 µL reaction mix containing 5 µL of master mix, 0.2 µL of RT enzyme, 2.3 µL of nuclease-free water, 0.2 µL of primers (TATA-Box Binding Protein/TBP; Hs00427620_m1, AKR1B10; Hs00252524_m1, HO-1; Hs01110250, NqO1; Hs01045993_g1, and TRXR1; Hs00917067_m1, Thermo Fisher), and 2 µL of pure RNA. The samples were analyzed using a QuantStudioTM 3 real-time PCR instrument with the following program: 1 × (10 min, 48.0 °C; 10 min, 95.0 °C); 40 × (30 s, 95.0 °C; 1 min, 60.0 °C). The Ct values were extracted by use of the ThermoFisher Cloud Software.

Cell Assay (BEAS-2B)

The upregulation of NQO1 mRNA in BEAS-2B cells was measured by qPCR, as previously described.[20] The potencies of the tested compounds were determined as EC_{50} values from dose-response experiments starting at 10 µM compound concentration, using 3.162-fold dilutions in 10 points. The total DMSO concentration is 0.1%.

Anti-inflammatory Effects in THP-1 Macrophages

Cell culture and differentiation of THP-1 monocytes to macrophages

THP1 cells (WT and Nrf2-KO) were cultured in RPMI (ThermoFisher) supplemented with 10% heat inactivated fetal calf serum (Sigma-Aldrich), 1000 U/mL penicillin, 1000 µg/mL streptomycin, and 2 mM L-glutamine (Gibco) at 37°C with 5% CO_2. THP-1 monocytes were seeded at 500,000 cells/well in a 24-well plate and differentiated into macrophages after 12 h of incubation with 150 nM phorbol 12-myristate 13-acetate (PMA). To investigate the anti-inflammatory effects of Keap1 targeting molecules, the PMA-differentiated THP-1 cells were stimulated with compound **24** and **28** at a concentration of 100 µM or DMSO for 24 h.

Inflammasome activation

To assess the inflammasome activation, differentiated THP-1 cells were first primed with LPS (100 ng/mL, 3 h) and subsequently treated with nigericin (10 µM, 1 h). Then, the cell supernatants and cell lysates were collected to check for the expression of target proteins involved in inflammasome activation.

ELISA of IL-1β release

The concentrations of IL-1β released in the conditioned medium of THP-1 cultures were determined by ELISA (R&D systems; DY201). The concentration was quantified using the standard solutions provided by the kit.

Immunoblotting and antibodies

THP-1 macrophage-like cells were lysed in RIPA lysis buffer, 10 min on ice, supplemented with protease inhibitor cocktail (Complete™ ULTRA Tablets, EDTA-free; ROCHE), and Benzonase nuclease (#1014-25KU, Sigma). Cell debris were removed by centrifugation at 10.000 g (4 °C). Protein concentration was determined by a micro-BCA kit (Thermo Fisher Scientific). Both cell lysates and supernatants were boiled in SDS sample buffer (mix containing XT sample buffer #16107991 and XT reducing agent #1610796; Bio RAD) and analysed by SDS-polyacrylamide gel electrophoresis. The supernatant samples were made by adding 20 µl of the supernatant to 10 µl of the SDS sample buffer and boiling it for 5 min at 95 °C. The immunoblotting analysis was performed with the following antibodies: Primary antibodies were mouse IgG1 anti-caspase-1(p20) (Adipogen, #AG-20B-0048, 1/1000), mouse IgG1 anti-IL-1b (p17) (cell signaling #12242, 1/500), mouse IgG2 anti-NLRP3 (Adipogen, #AG-20B-0014, 1/1000), rabbit IgG anti-gasdermin D (Abcam, AB215203-1001, 1/1000), mouse mAB anti-vinculin (sigma, #V9264, 1/10.000), rabbit mAB anti-pTBK-ser172 (cell signaling #5483, 1/1000), rabbit mAB anti-pSTAT1-tyr701 (cell signaling #7649, 1/1000), rabbit mAB anti-pSTING-ser366 (cell signaling #50907, 1/1000), rabbit mAB anti-TBK/NAK (cell signaling #3504, 1/1000), rabbit mAB anti-STAT1 (cell signaling #14994, 1/1000), rabbit mAB anti-STING (cell signaling #13647, 1/1000), rabbit mAB anti-ISG15 (cell signaling #2758, 1/1000), rabbit mAB anti-IFIT1 (cell signaling #14769, 1/1000). Secondary antibodies were Donkey Anti-Mouse IgG (H+L) (Jackson Immuno Research #715-036-150) and Donkey Anti-rabbit IgG (H+L) (Jackson Immuno Research # 711-035-152).

RNA Sequencing and Analysis

RNA isolation

THP-1 cells were treated with PMA (25 ng/ml final concentration) and cultured overnight to allow differentiation and adherence. Cells were challenged with 10 µM SFN, DMF, **24**, **28**, 1 µM KI-696, 0.1 µM CDDO-Me (RTA-402), or vehicle control in 2% FBS in RPMI for 12 or 24 h (n = 3–4 technical replicates). Cells were washed with PBS and total RNA was isolated using the RNeasy Mini Kit according to the manufacturer's recommended protocol (Qiagen Inc., Valencia, CA). Total RNA purity from the seven

experimental groups was checked using the NanoPhotometer NP80 spectrophotometer (IMPLEN, CA, USA). Sequencing libraries (poly A enrichment) were generated by Novogene (Davis, CA). Briefly, messenger RNA was purified from total RNA using poly-T oligo-attached magnetic beads. After fragmentation, the first strand cDNA was synthesized using random hexamer primers followed by the second strand cDNA synthesis. The library was ready after end repair, A-tailing, adapter ligation, size selection, amplification, and purification. The library was checked with Qubit and real-time PCR for quantification and bioanalyzer for size distribution detection.

Illumina RNA sequencing, quantification of differential gene expression, and pathway analysis

Quantified libraries were pooled and sequenced according to effective library concentration and data amount through the Illumina platform using the NovaSeq PE150 strategy, as previously described.[21] The expected number of Fragments Per Kilobase of transcript sequence per Millions base pairs sequenced (FPKM) was used to estimate gene expression levels. Both the principal components analysis (PCA) plots and the Pearson's correlation heat map were generated using normalized reads per kilobases of transcript per 1 million mapped reads (RPKM) count as previously described. The square of the Pearson correlation coefficient of all biological replicates were > 0.9. The variation within each experimental group in the entire dataset was also analyzed via principal components (PCs). The PCA demonstrated expected grouping among replicates within samples and sample groups spread across the two PCs. Genes with an adjusted P-value (p-adj) < 0.05 normalized by DESeq were initially assigned as differentially expressed. The p-adj value is the transformation of the p value after accounting for multiple testing. The p-adj takes into account the false discovery rate (FDR) of p-value, also called a q-value. The baseline is < 0.05 (p-adj < 0.05 is significant, p-adj < 0.01 is highly significant). All differentially expressed genes, Kyoto Encyclopedia of Genes and Genomes (KEGG) enrichment pathways and Gene Ontology (GO) enrichment analysis reported herein had an adjusted P-value < 0.05, and a Log2 FC > 1 (upregulated) or a Log2 FC < -1 (downregulated).

Cytotoxicity Assessment

THP1 cells were exposed to the compounds for 48 hours at 10 and 100 µM. Viability was quantified using propidium iodide exclusion through flow cytometry. PI-positive cells (dead or necrotic cells) were quantified through red fluorescence on a BD C6 Accuri flow cytometer using a 488 nm excitation wavelength and an emission filter of 585 nm ± 40 nm (BD Biosciences). 50,000 events were collected (n = 3 technical replicates per condition). The experiment was repeated twice. Data are presented as mean ± SEM (n = 4–10).

Chemical Synthesis

Material and Methods

All chemicals used for synthesis were obtained from commercial suppliers and used without prior purification. ^{1}H NMR and ^{13}C NMR spectra were recorded using either a 600 MHz Bruker Avance III HD instrument equipped with a cryogenically cooled 5 mm dual probe or a 400 Bruker Avance III instrument equipped with a 5 mm broad band probe. Samples were dissolved in either DMSO-d_6 (VWR Chemicals, 99.8% D) or $CDCl_3$ (Cambridge Isotope Laboratories, Inc., 99.8% D) and analyzed at 300 K. Thin layer chromatography (TLC) analyses were performed using TLC silica gel 60 F_{254} aluminum plates (Merck). Liquid chromatography-mass spectra (LC-MS) were obtained with an Agilent 6410 Triple Quadrupole Mass Spectrometer instrument using electron spray ionization (ESI) coupled to an Agilent 1200 high-performance liquid chromatography system with a C18 reversed-phase column (Zorbax Eclipse XBD-C18, 4.6 mm × 50 mm), an autosampler, and a diode array detector, using a linear gradient of the binary solvent system of buffer A (Milli-Q H_2O/MeCN/formic acid, 95:5:0.1 v/v%) to buffer B (MilliQ H_2O/MeCN/formic acid, 5:95:0.043 v/v%) with a flow rate of 1 mL/min. During LC-MS analysis, evaporative light scattering traces were obtained with a Sedere Sedex 85 Light Scattering Detector. Flash column chromatography was carried out using either prepacked RediSep Rf silica flash cartridges or a RediSep Rf reversed-phase C18 cartridge on a CombiFlash® Rf+ or EZPrep apparatus. All final compounds showed ≥95% purity according to LC-MS results.

General Procedures

General procedure A

To a solution of various acids (1 equiv) in DMF (3.0 mL pr. mmol) cooled to 0 °C was added EDC·HCl (1.5 equiv), HOBt (1.5 equiv) and DIPEA (2 equiv). The mixture was stirred at 0 °C for 30 min. After pre-activation, a solution of various amines (1 equiv) in DMF (2.0 mL pr. mmol) was added by syringe and the mixture stirred under nitrogen at room temperature overnight. Upon reaction completion (as determined by LC-MS), water (10 mL pr. mmol) was added and the mixture extracted with EtOAc (3 × 10 mL pr. mmol). The combined organic layers were washed with 1 M HCl (10 mL pr. mmol), sat. Na_2CO_3 (10 mL pr. mmol) and sat. brine (10 mL pr. mmol), dried over Na_2SO_4, filtered and evaporated to dryness in vacuo. The crude was purified by silica gel column chromatography to afford the desired product.

General procedure B

To a solution of various esters (1 equiv), 1 M aq. NaOH (4 equiv) was added in MeOH (1-3 mL). The mixture was stirred at room temperature for 22 h. Upon reaction completion, the pH of the aq. solution was adjusted to pH to acid with 1 M HCl resulting in compound precipitation, extracted with EtOAc three times, washed with brine, and dried over Na_2SO_4, filtered and concentrated under reduced pressure. The crude was purified by silica gel column chromatography to afford the desired product.

General procedure C

A solution of various unsaturated bromides (1 equiv), methyl acrylate (1.5 equiv), $Pd(OAc)_2$ (0.05 equiv), tri(*o*-tolyl)phosphine (0.1 equiv), and Et_3N (7.17 equiv) in MeCN (10 mL) was stirred at reflux (90 °C) under nitrogen protection for 1.5 h. Upon reaction completion (as determined by LC-MS), water (10 mL pr. mmol) was added and the mixture extracted with EtOAc (3 × 10 mL pr. mmol). The combined organic layers were washed with 1 M HCl (10 mL pr. mmol), sat. $NaHCO_3$ (10 mL pr. mmol) and sat. brine (10 mL pr. mmol), dried over Na_2SO_4, filtered and concentrated under reduced pressure to provide the crude product. The crude was purified by silica gel column chromatography to afford the desired product.

General procedure D

To a 50 mL round-bottomed flask charged with a magnetic stirring bar were added various acrylates (1 equiv) in MeOH (3 mL). The solution was degassed for 10 min. Then 5% Pd/C (20 w/w% wet-basis) was added against a positive nitrogen flow. The flask was capped and subjected to three vacuum-nitrogen cycles. Then hydrogen was introduced via a balloon, and the mixture stirred at room temperature for 22 h. Upon reaction completion, the flask was subjected to three vacuum-nitrogen cycles. The mixture was filtered through a compact bed of celite and the filter cake washed thoroughly with EtOAc. The combined filtrates were evaporated to dryness in vacuum without purification to afford the desired product.

General procedure E

A solution of 2-((7-bromonaphthalen-2-yl)oxy)acetic acid (1 equiv) in DMF (4mL pr. mmol) was made and cooled to -15°C. HATU (2 equiv), DIPEA (3 equiv), and amine (1.5 equiv) were added. The mixture was stirred at room temperature for overnight. Upon reaction completion (as determined by LC-MS), water (10 mL pr. mmol) was added and the mixture extracted with EtOAc (3 × 10 mL pr. mmol). The combined organic layers were washed with 1 M HCl (10 mL pr. mmol), sat. Na_2CO_3 (10 mL pr. mmol) and sat. brine (10 mL pr. mmol), dried over Na_2SO_4, filtered and concentrated under reduced pressure to provide the crude product. The crude was purified by silica gel column chromatography to afford the desired product.

General procedure F

To a vial charged with a magnetic stirring bar were added boronic ester intermediate (**12-I1**, **29-I1** or **39-I2**) (1.5 equiv), various cinnamate esters (0.05 g, 0.25 mmol), $[Rh(COD)Cl]_2$ (0.1 equiv) and TEA(3 equiv) in dioxane/H_2O (1.5 mL/0.5 mL). The mixture was degassed and microwaved at 120 °C for 50 min. Upon reaction completion (as determined by LC-MS), and after cooling down, the solvent was concentrated under reduced pressure to provide the crude product. The crude was purified by silica gel column chromatography to afford the desired product.

General procedure G

To a vial charged with a magnetic stirring bar were added various esters (1 equiv) in methanol (2 mL). Then a solution of 2 M aq. NaOH (2 equiv)) was added in the mixture. The mixture was microwaved at 100 °C for 30 min. Upon reaction completion, the mixture was concentrated in vacuo to remove MeOH. The pH of the aq. solution was then adjusted to acidic pH with 1 M HCl resulting in compound precipitation, and extracted with EtOAc (3 × 10 mL pr. mmol). The combined organic layers were washed with sat. brine (10 mL pr. mmol), dried over Na_2SO_4, filtered and concentrated under reduced pressure to provide the crude product. The crude was purified by silica gel column chromatography to afford the desired product.

Synthesis of Novel Keap1-Nrf2 Inhibitors

Synthesis of fragment hit and analogues (1–4)

N-Cyclohexyl-2-phenoxyacetamide (1)

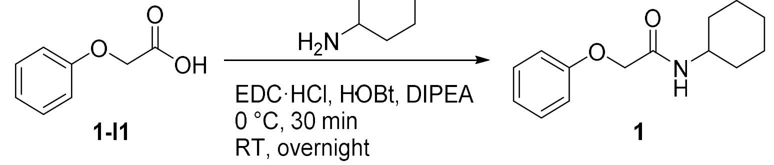

The reaction was conducted following the general procedure **A**, using 2-phenoxyacetic acid (**1-I1**, 0.20 g, 1.32 mmol), EDC·HCl (0.38 g, 1.97 mmol), HOBt (0.27 g, 1.97 mmol), DIPEA (0.46 mL, 2.63 mmol) and cyclohexanamine (0.13 g, 1.32 mmol) to obtain 0.18 g (57%) of **1** as a white solid. ^{1}H NMR (400 MHz, DMSO-d_6) δ 7.83 (d, *J* = 8.1 Hz, 1H), 7.35 – 7.25 (m, 2H), 7.00 – 6.91 (m, 3H), 4.44 (s, 2H), 3.62 (s, 1H), 1.76 – 1.61 (m, 4H), 1.56 (d, *J* = 12.5 Hz, 1H), 1.26 (qd, *J* = 10.5, 2.7 Hz, 4H), 1.11 (d, *J* = 10.7 Hz, 1H). ^{13}C NMR (101 MHz, DMSO-d_6) δ 166.65, 158.00, 129.61 (2C), 121.25, 114.87 (2C), 67.13, 47.59, 32.42 (2C), 25.33, 24.83 (2C). LC-MS (ESI): *m/z* = 234.1 [M+H]$^+$, t_R = 4.35 min.

3-(3-(2-(Cyclohexylamino)-2-oxoethoxy)phenyl)propanoic acid (2)

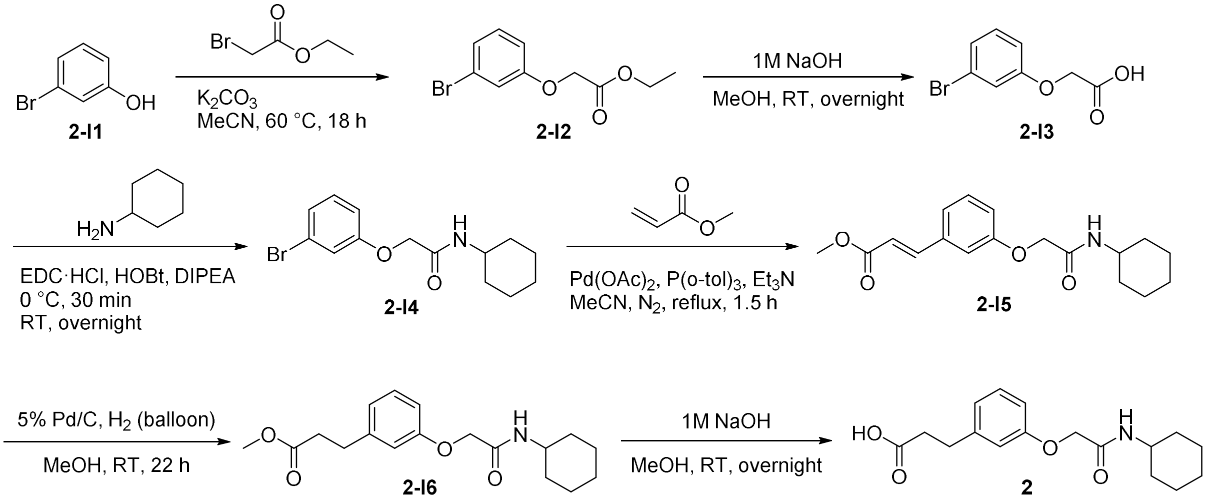

Ethyl 2-(3-bromophenoxy)acetate (2-I2). A mixture of 3-bromophenol (**2-I1**, 1 g, 5.78 mmol), K_2CO_3 (3.2 g, 23.12 mmol) and ethyl 2-bromoacetate (1.33 g, 6.07 mmol) in MeCN (18 mL) was stirred at 60 °C for 18 h. Upon reaction completion, the mixture was cooled to room temperature, extracted with EtOAc three times, washed with brine, and dried over Na_2SO_4, filtered and concentrated under reduced pressure. The crude was purified by silica gel column chromatography to obtain 0.85 g (57%) of **2-I2** as a white solid. ^{1}H NMR (400 MHz, DMSO-d_6) δ 7.26 (t, *J* = 8.3 Hz, 1H), 7.16 (dddd, *J* = 3.7, 2.8, 1.8, 1.0 Hz, 2H), 6.96 (ddd, *J* = 8.3, 2.4, 1.1 Hz, 1H), 4.83 (s, 2H), 4.17 (q, *J* = 7.1 Hz, 2H), 1.21 (t, *J* = 7.1 Hz, 3H). LC-MS (ESI): m/z = 259.0, t_R = 4.57 min.

Ethyl 2-(3-bromophenoxy)acetic acid (2-I3). The reaction was conducted following the general procedure **B**, using **2-I2** (0.80 g, 3.09 mmol) and 1 M aq. NaOH (12 mL) to obtain 0.66 g (92.0%) **2-I3** as a white solid. ^{1}H NMR (400 MHz, DMSO-d_6) δ 13.04 (s, 1H), 7.25 (t, *J* = 8.0 Hz, 1H), 7.17 – 7.11 (m, 2H), 6.95 (ddd, *J* = 8.4, 2.5, 1.0 Hz, 1H), 4.73 (s, 2H). LC-MS (ESI): m/z = 231.0, t_R = 3.76 min.

2-(3-Bromophenoxy)-*N*-cyclohexylacetamide (2-I4). The reaction was conducted following the general procedure **A**, using **2-I3** (0.61 g, 2.64 mmol), EDC·HCl (0.76 g, 3.96 mmol), HOBt (0.54 g, 3.96 mmol), DIPEA

(0.92 mL, 5.28 mmol) and cyclohexanamine (0.26 g, 2.64 mmol) to obtain 0.39 g (47.1%) of **2-I4** as a white solid. ^{1}H NMR (400 MHz, DMSO-d_6) δ 7.89 (d, *J* = 8.1 Hz, 1H), 7.26 (t, *J* = 8.1 Hz, 1H), 7.19 – 7.12 (m, 2H), 6.99 – 6.93 (m, 1H), 4.48 (s, 2H), 3.72 – 3.54 (m, 1H), 1.70 (ddt, *J* = 12.4, 8.9, 3.4 Hz, 4H), 1.36 – 1.18 (m, 6H), 0.92 – 0.81 (m, 1H). LC-MS (ESI): m/z = 313.2, t_R = 4.72 min.

Methyl (*E*)-3-(3-(2-(cyclohexylamino)-2-oxoethoxy)phenyl)acrylate (2-I5). The reaction was conducted following the general procedure **C**, using **2-I4** (0.20 g, 0.64 mmol), methyl acrylate (0.09 mL, 0.96 mmol), $Pd(OAc)_2$ (0.01 g, 0.03 mmol), tri(*o*-tolyl)phosphine (0.02 g, 0.06 mmol), and Et_3N (0.62ml, 4.40 mmol) to obtain 0.20 g (79.3%) of **2-I5** as a white solid. ^{1}H NMR (400 MHz, $CDCl_3$) δ 7.65 (d, *J* = 16.0 Hz, 1H), 7.34 (t, *J* = 7.9 Hz, 1H), 7.19 (d, *J* = 7.7 Hz, 1H), 7.07 (t, *J* = 2.0 Hz, 1H), 6.94 (dd, *J* = 8.3, 2.6 Hz, 1H), 6.43 (d, *J* = 16.0 Hz, 1H), 6.38 (d, *J* = 8.8 Hz, 1H), 4.48 (s, 2H), 3.92 – 3.84 (m, 1H), 3.81 (s, 3H), 1.97 – 1.89 (m, 2H), 1.76 – 1.68 (m, 2H), 1.63 (d, *J* = 13.1 Hz, 2H), 1.39 (q, *J* = 12.2 Hz, 4H), 1.28 – 1.14 (m, 2H). LC-MS (ESI): m/z = 318.1, t_R = 4.49 min.

Methyl 3-(3-(2-(cyclohexylamino)-2-oxoethoxy)phenyl)propanoate (2-I6). The reaction was conducted following the general procedure **D**, using **2-I5** (0.16 g, 0.32 mmol) and 5% Pd/C (0.01 g) to obtain **2-I6** as a crude colorless oil. (0.17g, >100%). LC-MS (ESI): m/z = 320.2, t_R = 4.41 min.

3-(3-(2-(Cyclohexylamino)-2-oxoethoxy)phenyl)propanoic acid (2). The reaction was conducted following the general procedure **B**, using **2-I6** (0.17 g, 5.17 mmol) and 1 M aq. NaOH (3 mL) to obtain 0.03 g (19.1%) of **2** as a white solid. ^{1}H NMR (600 MHz, DMSO-d_6) δ 12.11 (s, 1H), 7.82 (d, *J* = 8.1 Hz, 1H), 7.19 (t, *J* = 7.8 Hz, 1H), 6.85 – 6.80 (m, 2H), 6.76 (ddd, *J* = 8.3, 2.6, 1.0 Hz, 1H), 4.41 (s, 2H), 3.67 – 3.57 (m, 1H), 2.78 (t, *J* = 7.7 Hz, 2H), 2.52 (d, *J* = 7.8 Hz, 2H), 1.76 – 1.63 (m, 4H), 1.59 – 1.52 (m, 1H), 1.32 – 1.19 (m, 4H), 1.11 (tdd, *J* = 12.0, 8.3, 3.7 Hz, 1H). ^{13}C NMR (151 MHz, DMSO-d_6) δ 174.16, 166.96, 158.31, 142.92, 129.74, 121.54, 115.32, 112.59, 67.42, 47.85, 35.56, 32.71 (2C), 30.82, 25.62, 25.12 (2C). LC-MS (ESI): m/z = 306.1, t_R = 3.91 min.

1-(3-(2-(Cyclohexylamino)-2-oxoethoxy)phenyl)piperidine-4-carboxylic acid (3)

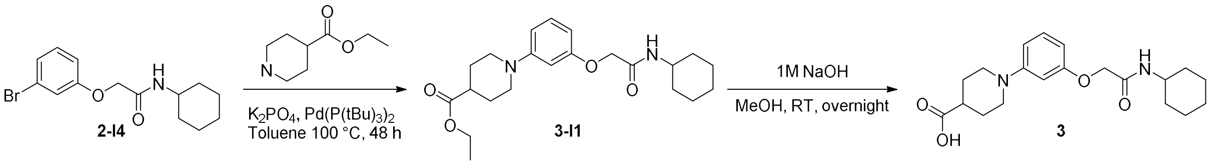

Ethyl 1-(3-(2-(cyclohexylamino)-2-oxoethoxy)phenyl)piperidine-4-carboxylate (3-I1). A solution of **2-I4** (0.38 g, 1.22 mmol), ethyl isonipecotate (0.13 g, 0.81 mmol), bis(tri-t-butylphosphine)palladium(0) (0.04 g, 0.08 mmol), and potassium phosphate (0.34 g, 1.62 mmol) in toluene (5 mL) was made. The reaction mixture was heated to 100 °C and left for 48 h. Upon reaction completion (as determined by LC-MS and TLC), the mixture was cooled to ambient temperature. Solids were filtered off from the resulting mixture, and the collected filtrate was concentrated under reduced pressure to provide the crude product. The crude was purified by silica gel column chromatography to obtain 0.17 g (35%) of 3-I1 as a white solid. ^{1}H NMR (600 MHz, DMSO-d_6) δ 7.77 (d, *J* = 8.2 Hz, 1H), 7.08 (t, *J* = 8.2 Hz, 1H), 6.53 (dd, *J* = 8.0, 2.3 Hz, 1H), 6.49 (t, *J* = 2.4 Hz, 1H), 6.33 (dd, *J* = 8.0, 2.3 Hz, 1H), 4.38 (s, 2H), 4.07 (q, *J* = 7.1 Hz, 2H), 3.61 (dt, *J* = 13.1, 3.9 Hz, 3H), 2.80 – 2.72 (m, 2H), 2.40 (tt, J = 11.1, 3.9 Hz, 1H), 1.87 (dd, *J* = 13.4, 3.7 Hz, 2H), 1.73 – 1.53 (m, 8H), 1.25 – 1.15 (m, 7H). LC-MS (ESI): m/z = 389.2, t_R = 3.76 min.

1-(3-(2-(Cyclohexylamino)-2-oxoethoxy)phenyl)piperidine-4-carboxylic acid (3). The reaction was conducted following the general procedure **B**, using **3-I1** (0.085 g, 0.22 mmol) and 1 M aq. NaOH (1.5 mL) to

obtain 0.04 g (50%) of **3** as a white solid. ^{1}H NMR (600 MHz, DMSO-d_6) δ 12.21 (s, 1H), 7.78 (d, *J* = 8.2 Hz, 1H), 7.09 (t, *J* = 8.2 Hz, 1H), 6.56 – 6.52 (m, 1H), 6.50 (t, *J* = 2.4 Hz, 1H), 6.34 (dd, *J* = 7.9, 2.3 Hz, 1H), 4.39 (s, 2H), 3.61 (dt, *J* = 12.9, 3.4 Hz, 3H), 2.79 – 2.71 (m, 2H), 2.40 (tt, *J* = 11.1, 3.9 Hz, 1H), 1.88 (dt, *J* = 13.2, 3.6 Hz, 2H), 1.76 – 1.53 (m, 8H), 1.26 (qd, *J* = 10.9, 10.3, 2.7 Hz, 4H). ^{13}C NMR (151 MHz, DMSO-d_6) δ 176.32, 167.04, 159.15, 152.65, 130.00, 109.33, 105.00, 103.00, 67.34, 48.33, 47.75, 40.57 (2C), 32.67 (2C), 27.84 (2C), 25.55, 25.05 (2C). LC-MS (ESI): m/z = 361.2, t_R = 3.57 min.

3-(7-(2-(Cyclohexylamino)-2-oxoethoxy)naphthalen-2-yl)propanoic acid (4)

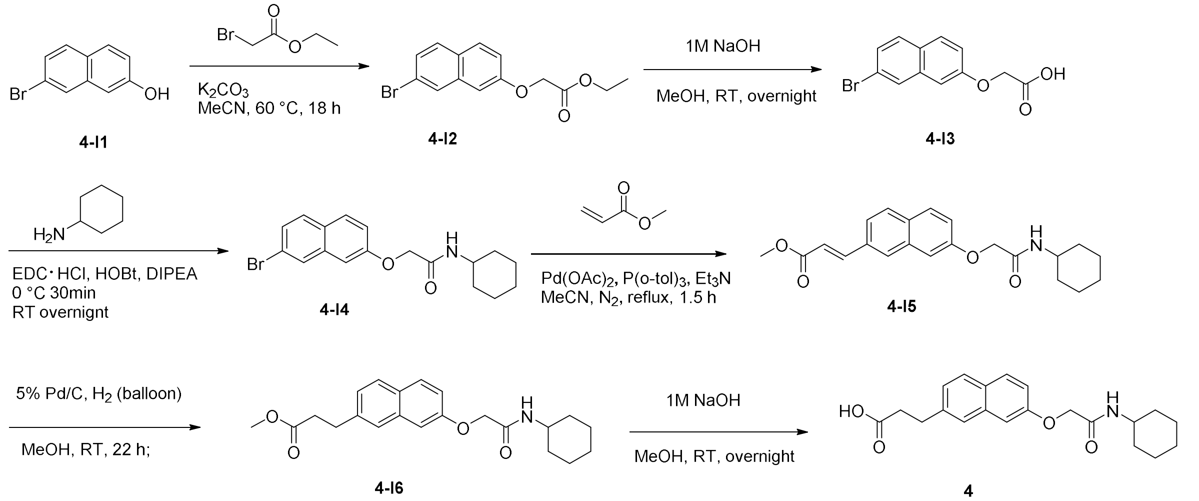

Ethyl 2-((7-bromonaphthalen-2-yl)oxy)acetate (4-I2). A mixture of 7-bromonaphthalen-2-ol (**4-I1**, 1 g, 4.5 mmol), K_2CO_3 (0.64 g, 4.6 mmol) and ethyl 2-bromoacetate (0.79 g, 4.7 mmol) in MeCN (10 mL) was stirred at 60 °C for 18 h. Upon reaction completion, the mixture was cooled to room temperature, extracted with EtOAc three times, washed with brine, and dried over Na_2SO_4, filtered and concentrated under reduced pressure. The crude was purified by silica gel column chromatography to obtain 1.08 g (78.3%) of **4-I2** as a white solid. ^{1}H NMR (400 MHz, DMSO-d_6) δ 8.06 (d, *J* = 2.0 Hz, 1H), 7.88 (d, *J* = 8.9 Hz, 1H), 7.82 (d, *J* = 8.7 Hz, 1H), 7.48 (dd, *J* = 8.7, 2.0 Hz, 1H), 7.30 (d, *J* = 2.6 Hz, 1H), 7.26 (dd, *J* = 8.9, 2.6 Hz, 1H), 4.89 (s, 2H), 4.20 (q, *J* = 7.1 Hz, 2H), 1.23 (t, *J* = 7.1 Hz, 3H). LC-MS (ESI): m/z = 309.0/311.0, t_R = 4.97 min.

2-((7-bromonaphthalen-2-yl)oxy)acetic acid (4-I3). The reaction was conducted following the general procedure **B**, using **4-I2** (1.08 g, 3.50 mmol) and 1 M aq. NaOH (14 mL) to obtain 0.78 g (79.6%) of **4-I3** as a white solid. ^{1}H NMR (600 MHz, DMSO-d_6) δ 12.29 (s, 1H), 8.08 – 8.05 (m, 1H), 7.90 – 7.85 (m, 1H), 7.83 – 7.79 (m, 1H), 7.47 (dd, *J* = 8.6, 2.0 Hz, 1H), 7.29 (d, *J* = 2.6 Hz, 1H), 7.24 (dd, *J* = 8.9, 2.6 Hz, 1H), 4.79 (s, 2H). LC-MS (ESI): m/z = 281.0/283.0, t_R = 4.23 min**.**

2-((7-Bromonaphthalen-2-yl)oxy)-*N*-cyclohexylacetamide (4-I4). The reaction was conducted following the general procedure **A**, using **4-I3** (0.30 g, 1.07 mmol), EDC·HCl (0.31 g, 1.61 mmol), HOBt (0.22 g, 1.61 mmol), DIPEA (0.4 mL, 2.14 mmol) and cyclohexanamine (0.32 g, 3.21 mmol) to obtain 0.12 g (31.6%) of **4-I4** as a white solid. ^{1}H NMR (600 MHz, DMSO-d_6) δ 8.03 (d, *J* = 2.0 Hz, 1H), 7.94 (d, *J* = 8.1 Hz, 1H), 7.89 (d, *J* = 8.9 Hz, 1H), 7.82 (d, *J* = 8.7 Hz, 1H), 7.48 (dd, *J* = 8.7, 2.0 Hz, 1H), 7.30 (dd, *J* = 8.9, 2.6 Hz, 1H), 7.28 (d, *J* = 2.5 Hz, 1H), 4.56 (s, 2H), 3.64 (td, *J* = 9.3, 4.9 Hz, 1H), 1.71 (ddt, *J* = 32.8, 8.9, 4.8 Hz, 4H), 1.59 – 1.54 (m, 1H), 1.30 – 1.25 (m, 4H), 1.16 – 1.09 (m, 1H). LC-MS (ESI): m/z = 362.1/364.2, t_R = 5.09 min.

Methyl (*E*)-3-(7-(2-(cyclohexylamino)-2-oxoethoxy)naphthalen-2-yl)acrylate (4-I5). The reaction was conducted following the general procedure **C**, using **4-I4** (0.12g, 0.33 mmol), methyl acrylate (0.05 mL, 0.50

mmol), $Pd(OAc)_2$ (0.01 g, 0.02 mmol), tri(*o*-tolyl)phosphine (0.02 g, 0.04 mmol), and Et_3N (0.34 mL, 2.38 mmol) to obtain 0.03 g (24.8%) of **4-I5** as a white solid. ^{1}H NMR (600 MHz, DMSO-d_6) δ 7.95 (d, *J* = 8.2 Hz, 1H), 7.88 (dd, *J* = 8.7, 3.7 Hz, 2H), 7.83 – 7.73 (m, 1H), 7.53 (tt, *J* = 7.5, 1.5 Hz, 1H), 7.41 (dd, *J* = 7.7, 4.0 Hz, 2H), 6.98 (ddd, *J* = 13.9, 7.7, 1.4 Hz, 1H), 6.75 (d, *J* = 16.0 Hz, 1H), 4.59 (s, 2H), 3.76 (s, 3H), 3.65 (d, *J* = 4.0 Hz, 1H), 1.79 – 1.63 (m, 4H), 1.57 (m, 4H), 1.12 (d, *J* = 10.7 Hz, 2H). LC-MS (ESI): m/z = 3 68.3, t_R = 4.82 min.

Methyl 3-(7-(2-(cyclohexylamino)-2-oxoethoxy)naphthalen-2-yl)propanoate (4-I6). The reaction was conducted following the general procedure **D**, using **4-I5** (0.03g, 0.08 mmol) and 5% Pd/C (0.01 g) to obtain **4-I6** as a crude colorless oil. LC-MS (ESI): m/z = 370.2, t_R = 4.56 min.

3-(7-(2-(Cyclohexylamino)-2-oxoethoxy)naphthalen-2-yl)propanoic acid (4). The reaction was conducted following the general procedure **B**, using **4-I6** (0.04 g, 0.10 mmol) and 1 M aq. NaOH (1 mL) to obtain 0.013 g (36.9%) of **4** as a white solid. ^{1}H NMR (600 MHz, DMSO-d_6) δ 12.11 (s, 1H), 7.92 (d, *J* = 8.2 Hz, 1H), 7.82 – 7.74 (m, 2H), 7.57 (d, *J* = 1.7 Hz, 1H), 7.26 (dd, *J* = 8.3, 1.7 Hz, 1H), 7.22 – 7.17 (m, 2H), 4.55 (s, 2H), 3.67 – 3.63 (m, 1H), 2.96 (t, *J* = 7.6 Hz, 2H), 2.62 (dq, *J* = 3.7, 1.9 Hz, 2H), 1.81 – 1.67 (m, 4H), 1.57 (d, *J* = 12.7 Hz, 1H), 1.28 – 1.24 (m, 4H), 1.12 (d, *J* = 11.8 Hz, 1H). ^{13}C NMR (151 MHz, DMSO-d_6) δ 173.17, 165.79, 155.27, 138.56, 133.61, 128.43, 126.98, 126.77, 124.60, 124.51, 117.40, 106.40, 66.50, 46.88, 34.53, 31.70 (2C), 29.96, 24.60, 24.11 (2C). LC-MS (ESI): m/z = 356.2, t_R = 4.23 min.

Synthesis of F2L analogues (5–11)

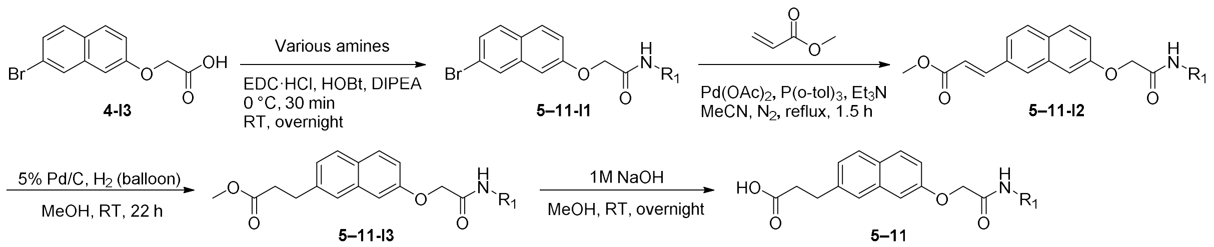

5: R_1 = tetrahydro-2H-pyran-4-yl
6: R_1 = morpholin-4-yl
7: R_1 = cycloheptyl
8: R_1 = phenyl
9: R_1 = 4-methoxyphenyl
10: R_1 = 2-mesityl
11: R_1 = 2,3,5,6-tetramethylphenyl

3-(7-(2-Oxo-2-((tetrahydro-2*H*-pyran-4-yl)amino)ethoxy)naphthalen-2-yl)propanoic acid (5)

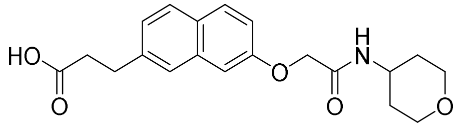

2-((7-Bromonaphthalen-2-yl)oxy)-*N*-(tetrahydro-2*H*-pyran-4-yl)acetamide (5-I1). The reaction was conducted following the general procedure **E**, using **4-I3** (0.37 g, 1.32 mmol), HATU (1.01 g, 2.64 mmol), DIPEA (0.7 mL, 3.95 mmol) and tetrahydro-2*H*-pyran-4-amine (0.20 g, 1.98 mmol) to obtain 0.8 g (92%) of **5-I1** as a white solid. ^1H NMR (600 MHz, DMSO-d_6) δ 8.10 (d, *J* = 7.9 Hz, 1H), 8.05 (d, *J* = 2.0 Hz, 1H), 7.89 (d, *J* = 8.9 Hz, 1H), 7.82 (d, *J* = 8.7 Hz, 1H), 7.48 (dd, *J* = 8.7, 2.0 Hz, 1H), 7.34 – 7.28 (m, 2H), 4.59 (s, 2H), 3.93 – 3.86 (m, 1H), 3.84 (ddd, *J* = 11.8, 4.1, 2.2 Hz, 2H), 3.35 (td, *J* = 11.6, 2.2 Hz, 2H), 1.69 (ddd, *J* = 12.5, 4.5, 2.1 Hz, 2H), 1.53 (dtd, *J* = 12.9, 11.4, 4.4 Hz, 2H). LC-MS (ESI): m/z = 364.0/366.1, t_R = 4.41 min.

Methyl (*E*)-3-(7-(2-oxo-2-((tetrahydro-2*H*-pyran-4-yl)amino)ethoxy)naphthalen-2-yl)acrylate (5-I2). The reaction was conducted following the general procedure **C**, using **5-I1** (0.2 g, 0.5 mmol), methyl acrylate (0.08 mL, 0.83 mmol), $Pd(OAc)_2$ (0.01 g, 0.03 mmol), tri(*o*-tolyl)phosphine (0.02 g, 0.06 mmol), and Et_3N (0.55 mL, 3.94 mmol) to obtain 0.23 g (94%) of **5-I2** as a light brown solid. ^1H NMR (600 MHz, DMSO-d_6) δ 8.11 (d, *J* = 8.0 Hz, 1H), 8.09 – 8.04 (m, 1H), 7.87 (dd, *J* = 8.7, 5.4 Hz, 2H), 7.82 – 7.74 (m, 2H), 7.34 – 7.28 (m, 2H), 6.74 (d, *J* = 16.0 Hz, 1H), 4.60 (s, 2H), 3.88 (dtd, *J* = 11.1, 7.1, 4.0 Hz, 1H), 3.83 (ddd, *J* = 11.8, 4.4, 2.4 Hz, 2H), 3.75 (s, 3H), 3.34 (td, *J* = 11.7, 2.2 Hz, 2H), 1.68 (ddd, *J* = 9.6, 4.6, 2.5 Hz, 2H), 1.57 – 1.47 (m, 2H). LC-MS (ESI): m/z = 370.2, t_R = 4.19 min.

Methyl 3-(7-(2-oxo-2-((tetrahydro-2*H*-pyran-4-yl)amino)ethoxy)naphthalen-2-yl)propanoate (5-I3). The reaction was conducted following the general procedure **D**, using **5-I2** (0.23 g, 0.63 mmol) and 5% Pd/C (0.3 g) to obtain 0.23 g (92.8%) of **5-I3** as a light brown solid. ^1H NMR (600 MHz, DMSO-d_6) δ 8.09 (d, *J* = 8.0 Hz, 1H), 7.80 (d, *J* = 8.7 Hz, 1H), 7.76 (d, *J* = 8.3 Hz, 1H), 7.59 – 7.56 (m, 1H), 7.30 – 7.23 (m, 1H), 7.23 – 7.18 (m, 2H), 4.58 (s, 2H), 3.94 – 3.76 (m, 3H), 3.59 (s, 3H), 3.36 (td, *J* = 11.7, 2.2 Hz, 2H), 3.00 (t, *J* = 7.6 Hz, 2H), 2.75 – 2.68 (m, 2H), 1.69 (ddt, *J* = 12.0, 4.5, 2.3 Hz, 2H), 1.53 (dtd, *J* = 12.9, 11.4, 4.4 Hz, 2H). LC-MS (ESI): m/z = 372.2, t_R = 4.11 min.

3-(7-(2-Oxo-2-((tetrahydro-2*H*-pyran-4-yl)amino)ethoxy)naphthalen-2-yl)propanoic acid (5). The reaction was conducted following the general procedure **B**, using **5-I3** (0.13 g, 0.35 mmol) and 1 M aq. NaOH (2 mL) to obtain 0.08 g (61.6%) of **5** as a white solid. ^1H NMR (600 MHz, DMSO-d_6) δ 12.12 (s, 1H), 8.09 (d, *J* = 8.0 Hz, 1H), 7.80 (d, *J* = 8.6 Hz, 1H), 7.76 (d, *J* = 8.3 Hz, 1H), 7.58 (d, *J* = 1.7 Hz, 1H), 7.26 (dd, *J* = 8.4, 1.7 Hz, 1H), 7.23 – 7.18 (m, 2H), 4.57 (s, 2H), 3.95 – 3.80 (m, 3H), 3.36 (dd, *J* = 11.7, 2.2 Hz, 2H), 2.96 (t, *J* = 7.6 Hz, 2H), 2.62 (t, *J* = 7.6 Hz, 2H), 1.69 (ddt, *J* = 11.3, 4.5, 2.2 Hz, 2H), 1.53 (dtd, *J* = 12.9, 11.4, 4.4 Hz, 2H). ^{13}C NMR

(151 MHz, DMSO-d_6) δ 174.21, 167.15, 156.28, 139.60, 134.64, 129.48, 128.01, 127.82, 125.61 (2C), 118.44, 107.47, 67.53, 66.46 (2C), 45.40, 35.56, 32.77 (2C), 30.99. LC-MS (ESI): m/z = 358.1, t_R = 3.65 min.

3-(7-(2-Morpholinoamino-2-oxoethoxy)naphthalen-2-yl)propanoic acid (6)

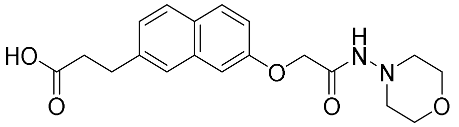

2-((7-Bromonaphthalen-2-yl)oxy)-*N*-morpholinoacetamide (6-I1). The reaction was conducted following the general procedure **E**, using **4-I3** (0.3 g, 1.07 mmol), HATU (0.82 g, 2.14 mmol), DIPEA (0.6 mL, 3.21 mmol) and morpholin-4-amine (0.17 g, 1.60 mmol) to obtain 0.27 g (68.9%) of **6-I1** as a white solid. ^{1}H NMR (600 MHz, DMSO-d_6) δ 7.87 – 7.79 (m, 3H), 7.47 (ddd, *J* = 19.1, 8.7, 2.0 Hz, 2H), 7.30 (d, *J* = 8.2 Hz, 2H), 4.56 (s, 2H), 3.63 (t, *J* = 4.6 Hz, 4H), 2.83 – 2.78 (m, 4H). LC-MS (ESI): m/z = 365.0/367.0, t_R = 4.17 min.

Methyl (*E*)-3-(7-(2-(morpholinoamino)-2-oxoethoxy)naphthalen-2-yl)acrylate (6-I2). The reaction was conducted following the general procedure **C**, using **6-I1** (0.2 g, 0.55 mmol), methyl acrylate (0.08 mL, 0.83 mmol), $Pd(OAc)_2$ (0.01 g, 0.03 mmol), tri(*o*-tolyl)phosphine (0.02 g, 0.06 mmol), and Et_3N (0.55 mL, 3.94 mmol) to obtain 0.17 g (85%) of **6-I2** as a white solid. ^{1}H NMR (600 MHz, DMSO-d_6) δ 9.37 – 8.75 (m, 1H), 7.83 – 7.72 (m, 2H), 7.59 (dd, *J* = 16.4, 1.6 Hz, 2H), 7.28 – 7.16 (m, 1H), 7.14 – 7.08 (m, 1H), 4.98 – 4.55 (s, 2H), 3.63 (t, *J* = 4.7 Hz, 4H), 3.59 (s, 3H), 2.99 (td, *J* = 7.6, 5.4 Hz, 2H), 2.83 – 2.78 (m, 2H), 2.72 (t, *J* = 7.6 Hz, 2H). LC-MS (ESI): m/z = 371.1, t_R = 3.99 min.

Methyl 3-(7-(2-morpholinoamino-2-oxoethoxy)naphthalen-2-yl)propanoate (6-I3). The reaction was conducted following the general procedure **D**, using **6-I2** (0.15 g, 0.46 mmol) and 5% Pd/C (0.3 g) to obtain **6-I3** as a crude colorless oil. LC-MS (ESI): m/z = 373.2, t_R = 3.89 min.

3-(7-(2-Morpholinoamino-2-oxoethoxy)naphthalen-2-yl)propanoic acid (6). The reaction was conducted following the general procedure **B**, using **6-I3** (0.17 g, 0.48 mmol) and 1 M aq. NaOH (5 mL) to obtain 0.043 g (26.4%) of **6** as a white solid. ^{1}H NMR (600 MHz, DMSO-d_6) δ12.12 (s, 1H), 9.37 – 8.81 (m, 1H), 7.95 – 7.64 (m, 2H), 7.59 (dd, *J* = 13.5, 1.7 Hz, 1H), 7.25 (ddd, *J* = 16.0, 8.3, 1.7 Hz, 1H), 7.21 – 7.16 (m, 1H), 7.14 – 7.08 (m, 1H), 4.98 – 4.55 (s, 2H), 3.63 (q, *J* = 5.9 Hz, 4H), 2.96 (td, *J* = 7.6, 4.9 Hz, 2H), 2.83 – 2.71 (m, 4H), 2.62 (td, *J* = 7.6, 1.6 Hz, 2H). ^{13}C NMR (151 MHz, DMSO-d_6) δ 174.22, 169.75, 156.28, 139.62, 134.66 129.51, 129.38, 127.98, 125.63 (2C), 118.40, 107.39, 67.02, 66.38 (2C), 54.99 (2C), 35.56, 31.02. LC-MS (ESI): m/z = 359.2, t_R = 3.44 min.

3-(7-(2-(Cycloheptylamino)-2-oxoethoxy)naphthalen-2-yl)propanoic acid (7)

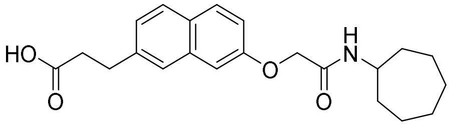

2-((7-Bromonaphthalen-2-yl)oxy)-*N*-cycloheptylacetamide (7-I1). The reaction was conducted following the general procedure **E**, using **4-I3** (0.07 g, 0.25 mmol), HATU (0.2 g, 0.5 mmol), DIPEA (0.15 mL, 0.75 mmol) and cycloheptanamine (0.05 g, 0.04 mmol) to obtain 0.047 g (50%) of **7-I1** as a white solid. ^{1}H NMR (600 MHz, DMSO-d_6) δ 8.04 (d, *J* = 2.0 Hz, 1H), 7.98 (d, *J* = 8.1 Hz, 1H), 7.89 (d, *J* = 8.9 Hz, 1H), 7.82 (d, *J* = 8.7 Hz, 1H), 7.48 (dd, *J* = 8.6, 2.0 Hz, 1H), 7.30 (dd, *J* = 8.9, 2.5 Hz, 1H), 7.28 (d, *J* = 2.5 Hz, 1H), 4.56 (s, 2H), 3.83 (tq, *J* = 12.6, 4.5 Hz, 1H), 1.82 – 1.74 (m, 2H), 1.66 – 1.36 (m, 9H), 1.24 (s, 1H). LC-MS (ESI): m/z = 376.0/378.1, t_R = 5.21 min.

Methyl (*E*)-3-(7-(2-(cycloheptylamino)-2-oxoethoxy)naphthalen-2-yl)acrylate (7-I2). The reaction was conducted following the general procedure **C**, using **7-I1** (0.05 g, 0.13 mmol), methyl acrylate (0.08 mL, 0.83 mmol), $Pd(OAc)_2$ (0.01 g, 0.03 mmol), tri(*o*-tolyl)phosphine (0.02 g, 0.06 mmol), and Et_3N (0.55 mL, 3.94 mmol) to obtain 0.027 g (54%) of **7-I2** as a white solid. ^{1}H NMR (600 MHz, DMSO-d_6) δ 8.07 – 8.03 (m, 1H), 7.99 (d, *J* = 8.1 Hz, 1H), 7.89 – 7.73 (m, 4H), 7.33 – 7.27 (m, 2H), 6.74 (d, *J* = 16.0 Hz, 1H), 4.57 (s, 2H), 3.87 – 3.80 (m, 1H), 3.75 (s, 3H), 1.81 – 1.73 (m, 2H), 1.65 – 1.26 (m, 10H). LC-MS (ESI): m/z = 382.2, t_R = 4.99 min.

Methyl 3-(7-(2-(cycloheptylamino)-2-oxoethoxy)naphthalen-2-yl)propanoate (7-I3). The reaction was conducted following the general procedure **D**, using **7-I2** (0.027 g, 0.07 mmol) and 5% Pd/C (0.31 g) to obtain 0.02 g (74.1%) of **7-I3** as a white solid. ^{1}H NMR (600 MHz, DMSO-d_6) δ 7.96 (d, J = 8.1 Hz, 1H), 7.79 (d, *J* = 8.7 Hz, 1H), 7.75 (d, *J* = 8.3 Hz, 1H), 7.56 (d, *J* = 1.7 Hz, 1H), 7.25 (dd, *J* = 8.3, 1.7 Hz, 1H), 7.21 – 7.16 (m, 2H), 4.54 (s, 2H), 3.83 (qt, *J* = 9.1, 4.4 Hz, 1H), 2.96 (t, *J* = 7.6 Hz, 2H), 2.61 (t, *J* = 7.6 Hz, 2H), 1.82 – 1.73 (m, 2H), 1.66 – 1.34 (m, 8H). LC-MS (ESI): m/z = 384.2, t_R = 4.92 min.

3-(7-(2-(Cycloheptylamino)-2-oxoethoxy)naphthalen-2-yl)propanoic acid (7). The reaction was conducted following the general procedure **B**, using **7-I3** (0.02 g, 0.05 mmol) and 1 M aq. NaOH (1 mL) to obtain 0.007 g (36.8%) of **7** as a white solid. ^{1}H NMR (600 MHz, DMSO-d_6) δ 12.11 (s, 1H), 7.96 (d, *J* = 8.1 Hz, 1H), 7.79 (d, *J* = 8.7 Hz, 1H), 7.75 (d, *J* = 8.3 Hz, 1H), 7.56 (d, *J* = 1.7 Hz, 1H), 7.25 (dd, *J* = 8.3, 1.7 Hz, 1H), 7.21 – 7.16 (m, 2H), 4.54 (s, 2H), 3.83 (qt, *J* = 9.1, 4.4 Hz, 1H), 2.96 (t, *J* = 7.6 Hz, 2H), 2.61 (t, *J* = 7.6 Hz, 2H), 1.82 – 1.73 (m, 2H), 1.66 – 1.34 (m, 10H). ^{13}C NMR (151 MHz, DMSO-d_6) δ 174.01, 166.34, 156.12, 139.39, 134.45, 129.26, 127.81, 127.60, 125.42, 125.34, 118.22, 107.21, 67.34, 49.88, 35.36, 34.50 (2C), 30.79, 27.94 (2C), 24.03 (2C). LC-MS (ESI): m/z = 370.2, t_R = 4.40 min.

3-(7-(2-Oxo-2-(phenylamino)ethoxy)naphthalen-2-yl)propanoic acid (8)

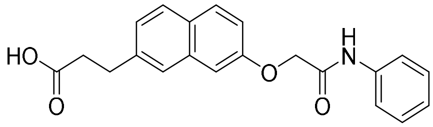

2-((7-bromonaphthalen-2-yl)oxy)-*N*-phenylacetamide (8-I1). The reaction was conducted following the general procedure **E**, using **4-I3** (0.1 g, 0.36 mmol), HATU (0.3 g, 0.71 mmol), DIPEA (0.2 mL, 1.07 mmol) and aniline (0.05 g , 0.53 mmol) to obtain 0.1 g (79.4%) of **8-I1** as a white solid. ^{1}H NMR (400 MHz, DMSO-d_6) δ 10.14 (s, 1H), 8.09 (d, *J* = 2.0 Hz, 1H), 7.95 – 7.89 (m, 1H), 7.84 (d, *J* = 8.7 Hz, 1H), 7.70 – 7.62 (m, 2H), 7.49 (dd, *J* = 8.7, 2.0 Hz, 1H), 7.35 (qd, *J* = 7.3, 2.1 Hz, 4H), 7.14 – 7.05 (m, 1H), 4.83 (s, 2H). LC-MS (ESI): m/z = 356.1/358.1, t_R = 4.85 min.

Methyl (E)-3-(7-(2-oxo-3-phenylpropoxy)naphthalen-2-yl)acrylate (8-I2). The reaction was conducted following the general procedure **C**, using **8-I1** (0.1 g, 0.28 mmol), methyl acrylate (0.04 mL, 0.42 mmol), $Pd(OAc)_2$ (0.03 g, 0.02 mmol), tri(*o*-tolyl)phosphine (0.08 g, 0.03 mmol), and Et_3N (0.3 mL, 2.02 mmol) to obtain 0.07g of **8-I2** as a white solid. ^{1}H NMR (400 MHz, DMSO-d_6) δ 10.14 (s, 1H), 8.10 (s, 1H), 7.89 (t, *J* = 8.5 Hz, 2H), 7.83 – 7.72 (m, 2H), 7.66 (d, *J* = 8.0 Hz, 2H), 7.44 – 7.22 (m, 5H), 7.08 (t, *J* = 7.4 Hz, 1H), 6.74 (d, *J* = 16.0 Hz, 1H), 4.84 (s, 2H), 3.74 (s, 3H). LC-MS (ESI): m/z = 361.1, t_R = 5.25 min.

Methyl 3-(7-(2-oxo-3-phenylpropoxy)naphthalen-2-yl)propanoate (8-I3). The reaction was conducted following the general procedure **D**, using **8-I2** (0.07 g, 0.19 mmol) and 5% Pd/C (0.1 g) to obtain 0.07 g (98.2%) of **8-I3** as a white solid. ^{1}H NMR (600 MHz, DMSO-d_6) δ 10.13 (s, 1H), 7.85 – 7.80 (m, 1H), 7.77 (d, *J* = 8.3 Hz, 1H), 7.69 – 7.64 (m, 2H), 7.60 (s, 1H), 7.37 – 7.30 (m, 2H), 7.27 (ddq, *J* = 9.7, 4.1, 2.3 Hz, 4H), 7.08

(t, J = 7.4 Hz, 1H)4.81 (s, 2H), 3.58 (s, 3H), 2.99 (t, J = 7.6 Hz, 2H), 2.72 (t, J = 7.6 Hz, 2H). LC-MS (ESI): m/z = 363.2, t_R = 5.11 min.

3-(7-(2-Oxo-2-(phenylamino)ethoxy)naphthalen-2-yl)propanoic acid (8). The reaction was conducted following the general procedure **B**, using **8-I3** (0.07 g, 0.19 mmol) and 1 M aq. NaOH (1 ml) to obtain 0.01 g (14.9%) of **8** as a white solid. ^{1}H NMR (600 MHz, DMSO-d_6) δ 12.11 (s, 1H), 10.12 (s, 1H), 7.82 (d, J = 9.6 Hz, 1H), 7.77 (d, J = 8.4 Hz, 1H), 7.69 – 7.64 (m, 2H), 7.60 (s, 1H), 7.36 – 7.30 (m, 2H), 7.29 – 7.22 (m, 3H), 7.09 (t, J = 7.4 Hz, 1H), 4.81 (s, 2H), 2.96 (t, J = 7.6 Hz, 2H), 2.62 (t, J = 7.6 Hz, 2H). ^{13}C NMR (151 MHz, DMSO-d_6) δ 173.44, 166.13, 155.57, 138.87, 138.10, 133.87, 128.80, 128.43 (2C), 127.25, 127.09, 124.92, 124.84, 123.41, 119.46 (2C), 117.65, 106.63, 66.95, 34.79, 30.23. LC-MS (ESI): m/z = 350.1, t_R = 4.15 min.

3-(7-(2-((4-Methoxyphenyl)amino)-2-oxoethoxy)naphthalen-2-yl)propanoic acid (9)

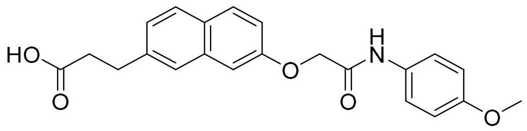

2-((7-Bromonaphthalen-2-yl)oxy)-*N*-(4-methoxyphenyl)acetamide (9-I1). The reaction was conducted following the general procedure **E**, using **4-I3** (0.2 g, 0.72 mmol), HATU (0.6 g, 1.42 mmol), DIPEA (0.4 mL, 2.13 mmol) and 4-methoxyaniline (0.1 mL, 1.07 mmol) to obtain 0.15g (53%) of **9-I1** as a white solid. ^{1}H NMR (600 MHz, DMSO-d_6) δ 9.99 (s, 1H), 8.08 (d, J = 2.0 Hz, 1H), 7.93 – 7.89 (m, 1H), 7.83 (d, J = 8.7 Hz, 1H), 7.59 – 7.53 (m, 2H), 7.48 (dd, J = 8.7, 2.0 Hz, 1H), 7.38 – 7.33 (m, 2H), 6.93 – 6.88 (m, 2H), 4.78 (s, 2H), 3.73 (s, 3H). LC-MS (ESI): m/z = 386.0/388.0, t_R = 4.87 min.

Methyl (*E*)-3-(7-(2-((4-methoxyphenyl)amino)-2-oxoethoxy)naphthalen-2-yl)acrylate (9-I2). The reaction was conducted following the general procedure **C**, using **9-I1** (0.15 g, 0.38 mmol), methyl acrylate (0.06 mL, 0.56 mmol), $Pd(OAc)_2$ (0.011 g, 0.02 mmol), tri(*o*-tolyl)phosphine (0.02 g, 0.04 mmol), and Et_3N (0.40 mL, 2.70 mmol) to obtain 0.11 g (30%) of **9-I2** as a white solid. ^{1}H NMR (600 MHz, DMSO-d_6) δ 10.01 (s, 1H), 8.10 (d, J = 1.7 Hz, 1H), 7.90 (dd, J = 12.0, 8.6 Hz, 2H), 7.82 – 7.74 (m, 2H), 7.59 – 7.54 (m, 2H), 7.39 – 7.34 (m, 2H), 6.93 – 6.88 (m, 2H), 6.75 (d, J = 16.0 Hz, 1H), 4.80 (s, 2H), 3.75 (s, 3H), 3.73 (s, 3H). LC-MS (ESI): m/z = 392.2, t_R = 4.60 min.

Methyl 3-(7-(2-((4-methoxyphenyl)amino)-2-oxoethoxy)naphthalen-2-yl)propanoate (9-I3). The reaction was conducted following the general procedure **D**, using **9-I2** (0.11 g, 0.29 mmol) and 5% Pd/C (0.03 g) to obtain 0.07g (61.9%) of **9-I3** as a white solid. ^{1}H NMR (600 MHz, DMSO-d_6) δ 9.99 (s, 1H), 7.85 – 7.74 (m, 2H), 7.60 (d, J = 1.6 Hz, 1H), 7.58 – 7.55 (m, 2H), 7.26 (tt, J = 4.4, 2.3 Hz, 3H), 6.93 – 6.89 (m, 2H), 4.77 (s, 2H), 3.73 (s, 3H), 3.58 (s, 3H), 2.99 (t, J = 7.6 Hz, 2H), 2.72 (t, J = 7.6 Hz, 2H). LC-MS (ESI): m/z = 394.2, t_R = 4.55 min.

3-(7-(2-((4-Methoxyphenyl)amino)-2-oxoethoxy)naphthalen-2-yl)propanoic acid (9). The reaction was conducted following the general procedure **B**, using **9-I3** (0.05 g, 0.13 mmol) and 1 M aq. NaOH (5 mL) to obtain 0.002 g (4.2%) of **9** as a white solid. ^{1}H NMR (600 MHz, DMSO-d_6) δ 12.13 (s, 1H), 9.99 (s, 1H), 7.81 (d, J = 9.6 Hz, 1H), 7.76 (d, J = 8.3 Hz, 1H), 7.63 – 7.52 (m, 3H), 7.25 (ddt, J = 7.1, 4.5, 2.2 Hz, 3H), 6.93 – 6.86 (m, 2H), 4.76 (s, 2H), 3.72 (s, 3H), 2.96 (t, J = 7.6 Hz, 2H), 2.61 (t, J = 7.6 Hz, 2H). ^{13}C NMR (151 MHz, DMSO-d_6) δ 174.22, 166.43, 156.33, 156.05, 139.63, 134.64, 131.91, 129.56, 128.02, 127.86, 125.70, 125.61, 121.91 (2C), 118.45, 114.32 (2C), 107.41, 67.73, 55.65, 35.56, 31.00. LC-MS (ESI): m/z = 380.2, t_R = 4.07 min.

3-(7-(2-(Mesitylamino)-2-oxoethoxy)naphthalen-2-yl)propanoic acid (10)

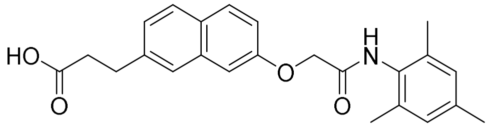

2-((7-Bromonaphthalen-2-yl)oxy)-*N*-mesitylacetamide (10-I1). The reaction was conducted following the general procedure **E**, using **4-I3** (0.07 g, 0.25 mmol), HATU (0.20 g, 0.50 mmol), DIPEA (0.15 mL, 0.75 mmol) and 2,4,6-trimethylaniline (0.6 mL, 0.38 mmol) to obtain 0.05 g (50%) of **10-I1** as a white solid. ^{1}H NMR (600 MHz, DMSO-d_6) δ 9.48 (s, 1H), 8.06 (d, *J* = 2.0 Hz, 1H), 7.92 (d, *J* = 8.9 Hz, 1H), 7.84 (d, *J* = 8.7 Hz, 1H), 7.50 (dd, *J* = 8.7, 2.0 Hz, 1H), 7.40 – 7.34 (m, 2H), 6.88 (s, 2H), 4.84 (s, 2H), 2.22 (s, 3H), 2.08 (s, 6H). LC-MS (ESI): m/z = 398.1/400.1, t_R = 5.10 min.

Methyl (*E*)-3-(7-(2-(mesitylamino)-2-oxoethoxy)naphthalen-2-yl)acrylate (10-I2). The reaction was conducted following the general procedure **C**, using **10-I1** (0.05 g, 0.16 mmol), methyl acrylate (0.02 mL, 0.15 mmol), $Pd(OAc)_2$ (0.01 g, 0.01 mmol), tri(*o*-tolyl)phosphine (0.02 g, 0.02 mmol), and Et_3N (0.30 mL, 0.20 mmol) to obtain 0.01 g (22%) of **10-I2** as a white solid. ^{1}H NMR (600 MHz, DMSO-d_6) δ 9.48 (s, 1H), 8.08 (d, *J* = 1.7 Hz, 1H), 7.95 – 7.75 (m, 4H), 7.39 – 7.36 (m, 2H), 6.87 (s, 2H), 6.75 (d, *J* = 16.0 Hz, 1H), 4.85 (s, 2H), 3.76 (s, 3H), 2.22 (s, 3H), 2.07 (s, 6H). LC-MS (ESI): m/z = 404.2, t_R = 4.89 min.

Methyl 3-(7-(2-(mesitylamino)-2-oxoethoxy)naphthalen-2-yl)propanoate (10-I3). The reaction was conducted following the general procedure **D**, using **10-I2** (0.01 g, 0.03 mmol) and 5% Pd/C (0.03 g) to obtain 0.007 g (61.9%) of **10-I3** as a white solid. LC-MS (ESI): m/z = 406.2, t_R = 4.83 min.

3-(7-(2-(Mesitylamino)-2-oxoethoxy)naphthalen-2-yl)propanoic acid (10). The reaction was conducted following the general procedure **B**, using **10-I3** (0.007 g, 0.02 mmol) and 1 M aq. NaOH (1 mL) to obtain 0.004 g (59.7%) of **10** as a white solid. ^{1}H NMR (600 MHz, DMSO-d_6) δ 9.48 (s, 1H), 7.82 (d, *J* = 8.9 Hz, 1H), 7.77 (d, *J* = 8.3 Hz, 1H), 7.58 (d, *J* = 1.7 Hz, 1H), 7.30 – 7.23 (m, 3H), 6.87 (s, 2H), 4.82 (s, 2H), 2.97 (t, *J* = 7.6 Hz, 2H), 2.63 (t, *J* = 7.6 Hz, 2H), 2.22 (s, 3H), 2.08 (s, 6H). ^{13}C NMR (151 MHz, DMSO-d_6) δ 174.21, 166.94, 156.31, 139.65, 136.11, 135.55 (2C), 134.61, 132.25, 129.52, 128.74 (2C), 128.04, 127.87, 125.62, 125.54, 118.57, 107.45, 67.49, 35.54, 30.99, 20.95, 18.45 (2C). LC-MS (ESI): m/z = 392.2, t_R = 4.35 min.

3-(7-(2-Oxo-2-((2,3,5,6-tetramethylphenyl)amino)ethoxy)naphthalen-2-yl)propanoic acid (11).

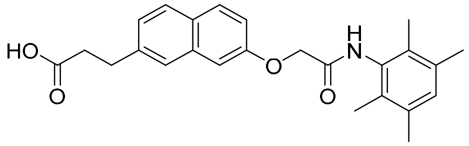

2-((7-Bromonaphthalen-2-yl)oxy)-*N*-(2,3,5,6-tetramethylphenyl)acetamide (11-I1). The reaction was conducted following the general procedure **E**, using **4-I3** (0.21 g, 0.72 mmol), HATU (0.55 g, 1.44 mmol), DIPEA (0.28 mL, 2.15 mmol) and 2,3,5,6-tetramethylaniline hydrochloride (0.21 g , 0.72 mmol) to obtain 0.54 g (95%) of **11-I1** as a white solid. ^{1}H NMR (400 MHz, DMSO-d_6) δ 9.53 (s, 1H), 8.04 (d, *J* = 2.0 Hz, 1H), 7.97 – 7.77 (m, 2H), 7.48 (dd, *J* = 8.7, 2.0Hz, 1H), 7.41 – 7.32 (m, 2H), 6.90 (s, 1H), 4.85 (s, 2H), 4.02 (q, *J* = 7.1 Hz, 1H), 2.16 (s, 6H), 1.97 (s, 6H). LC-MS (ESI): m/z = 312.1/414.1, t_R = 5.27 min.

Methyl (*E*)-3-(7-(2-oxo-2-((2,3,5,6-tetramethylphenyl)amino)ethoxy)naphthalen-2-yl)acrylate (11-I2). The reaction was conducted following the general procedure **C**, using **11-I1** (0.21 g, 0.49 mmol), methyl acrylate (0.07 mL, 0.73 mmol), $Pd(OAc)_2$ (0.01 g, 0.03 mmol), tri(*o*-tolyl)phosphine (0.02 g, 0.05 mmol), and Et_3N (0.49 mL, 3.48 mmol) to obtain 0.30 g (97.2%) of **11-I2** as a white solid. ^{1}H NMR (600 MHz, DMSO-d_6) δ 9.56

(s, 1H), 8.10 – 8.07 (m, 1H), 7.94 – 7.88 (m, 2H), 7.82 (d, J = 16.0 Hz, 1H), 7.78 (dd, J = 8.6, 1.7 Hz, 1H), 7.41 (d, J = 2.6 Hz, 1H), 7.39 (dd, J = 8.8, 2.6 Hz, 1H), 6.91 (s, 1H), 6.76 (d, J = 16.0 Hz, 1H), 4.88 (s, 2H), 3.76 (s, 3H), 2.17 (s, 6H), 1.98 (s, 6H). LC-MS (ESI): m/z = 418.2, t_R = 5.07 min.

Methyl 3-(7-(2-oxo-2-((2,3,5,6-tetramethylphenyl)amino)ethoxy)naphthalen-2-yl)propanoate (11-I3). The reaction was conducted following the general procedure **D**, using **11-I3** (0.30 g, 1.143 mmol) and 5% Pd/C (0.3 g) to obtain 0.30 g (98.2%) of **11-I3** as a white solid. ^{1}H NMR (600 MHz, DMSO-d_6) δ 9.54 (s, 1H), 7.83 (d, J = 8.9 Hz, 1H), 7.78 (d, J = 8.3 Hz, 1H), 7.59 (d, J = 1.7 Hz, 1H), 7.33 – 7.23 (m, 3H), 6.91 (s, 1H), 4.85 (s, 2H), 3.59 (s, 3H), 3.01 (t, J = 7.6 Hz, 2H), 2.73 (t, J = 7.6 Hz, 2H), 2.17 (s, 6H), 1.98 (s, 6H). LC-MS (ESI): m/z = 420.3, t_R = 5.00 min.

3-(7-(2-Oxo-2-((2,3,5,6-tetramethylphenyl)amino)ethoxy)naphthalen-2-yl)propanoic acid (11). The reaction was conducted following the general procedure **B**, using **11-I3** (0.06 g, 0.43 mmol) and 1 M aq. NaOH (2 mL) to obtain 0.30 g (96.1%) of **11** as a white solid. ^{1}H NMR (600 MHz, DMSO-d_6) δ 9.55 (s, 1H), 7.82 (d, J = 8.9 Hz, 1H), 7.77 (d, J = 8.3 Hz, 1H), 7.59 (s, 1H), 7.32 – 7.24 (m, 3H), 6.91 (s, 1H), 4.84 (s, 2H), 2.98 (t, J = 7.6 Hz, 2H), 2.63 (t, J = 7.6 Hz, 2H), 2.17 (s, 6H), 1.99 (s, 6H). ^{13}C NMR (151 MHz, DMSO-d_6) δ 174.22, 166.99, 156.36, 139.65, 134.63, 133.65 (2C), 131.58 (2C), 130.16, 129.53, 128.05, 127.88, 125.60, 125.59, 118.58, 107.49, 67.53, 40.41, 35.56, 31.01, 20.20 (2C), 14.76 (2C). LC-MS (ESI): m/z = 406.2, t_R = 4.54 min.

Synthesis of the cycloheptyl series (12–28)

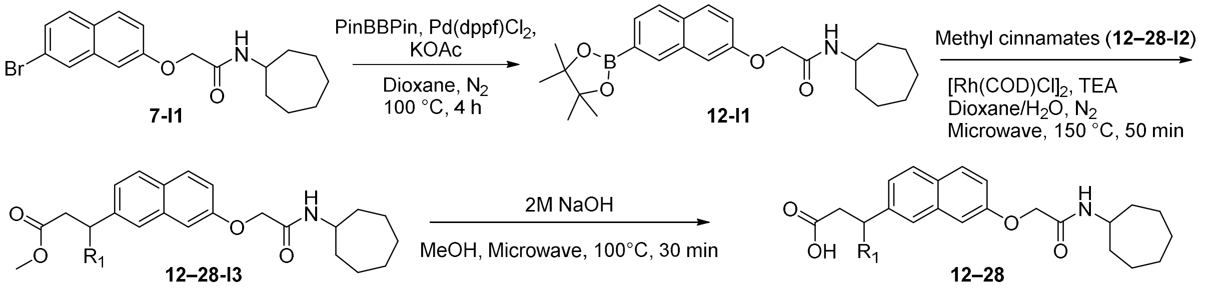

**12**: R_1 = phenyl	**21**: R_1 = benzofuran-5-yl
**13**: R_1 = 4-methoxyphenyl	**22**: R_1 = 2,2-difluorobenzo[*d*][1,3]dioxol-5-yl
**14**: R_1 = 3-methoxyphenyl	**23**: R_1 = 2,3-dihydrobenzo[*b*][1,4]dioxin-6-yl
**15**: R_1 = 4-chlorophenyl	**24**: R_1 = 6-methylbenzo[*d*][1,3]dioxol-5-yl
**16**: R_1 = 3-chlorophenyl	**25**: R_1 = 6-methyl-2,3-dihydrobenzofuran-5-yl
**17**: R_1 = 4-methoxy-2-methylphenyl	**26**: R_1 = 6-methylbenzofuran-5-yl
**18**: R_1 = 3-chloro-2-methylphenyl	**27**: R_1 = 2,2-difluoro-6-methylbenzo[*d*][1,3]dioxol-5-yl
**19**: R_1 = benzo[*d*][1,3]dioxol-5-yl	**28**: R_1 = 7-methyl-2,3-dihydrobenzo[*b*][1,4]dioxin-6-yl
**20**: R_1 = 2,3-dihydrobenzofuran-5-yl	

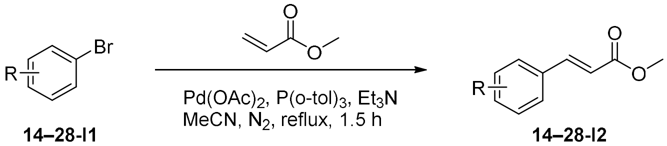

**12-I2**: methyl cinnamate (commercial available)	**21-I2**: methyl (*E*)-3-(benzofuran-5-yl) acrylate
**13-I2**: methyl (*E*)-3-(4-methoxyphenyl)acrylate (commercial available)	**22-I2**: methyl (*E*)-3-(2,2-difluorobenzo[d][1,3]dioxol-5-yl)acrylate
**14-I2**: methyl (*E*)-3-(3-methoxyphenyl)acrylate	**23-I2**: methyl (*E*)-3-(2,3-dihydrobenzo[b][1,4]dioxin-6-yl)acrylate
**15-I2**: methyl (*E*)-3-(4-chlorophenyl)acrylate	**24-I2**: methyl (*E*)-3-(6-methylbenzo[d][1,3]dioxol-5-yl)acrylate
**16-I2**: methyl (*E*)-3-(3-chlorophenyl)acrylate	**25-I2:** methyl (*E*)-3-(6-methyl-2,3-dihydrobenzofuran-5-yl)acrylate
**17-I2**: methyl (*E*)-3-(4-methoxy-2-methylphenyl)acrylate	**26-I2**: methyl (*E*)-3-(6-methylbenzofuran-5-yl)acrylate
**18-I2**: methyl (*E*)-3-(3-chloro-2-methylphenyl)acrylate	**27-I2**: methyl (*E*)-3-(7-methyl-2,3-dihydrobenzo[b][1,4]dioxin-6-yl)acrylate
**19-I2**: methyl (*E*)-3-(benzo[d][1,3]dioxol-5-yl)acrylate	**28-I2**: methyl (*E*)-3-(7-methyl-2,3-dihydrobenzo[b][1,4]dioxin-6-yl)acrylate
**20-I2**: methyl (*E*)-3-(2,3-dihydrobenzofuran-5-yl)acrylate	

3-(7-(2-(Cycloheptylamino)-2-oxoethoxy)naphthalen-2-yl)-3-phenylpropanoic acid (12)

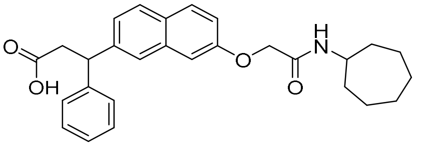

***N*-Cycloheptyl-2-((7-(4,4,5,5-tetramethyl-1,3,2-dioxaborolan-2-yl)naphthalen-2-yl)oxy)acetamide (12-I1).** A solution of **7-I1** (0.30 g, 0.83 mmol), PinB-BPin (0.26 g, 0.99 mmol), KOAc (0.25 g, 2.48 mmol), and Pd(dppf)Cl_2 (0.02 g, 0.025 mmol) in dioxane (3 mL) was stirred at 100 °C for 4 h under N_2 atmosphere. Upon reaction completion (as determined by LC-MS) and after cooling down, the salts were filtered out and the resulting filtrate was concentrated. The crude was purified by silica gel column chromatography to obtain 0.70 g (90%) of **12-I1** as a white solid. ^1H NMR (400 MHz, DMSO-d_6) δ 8.17 (d, *J* = 1.1 Hz, 1H), 7.98 (d, *J* = 8.1 Hz, 1H), 7.84 (dd, *J* = 15.4, 8.6 Hz, 2H), 7.57 (dd, *J* = 8.1, 1.2 Hz, 1H), 7.38 (d, *J* = 2.6 Hz, 1H), 7.32 (dd, *J* = 8.9, 2.6 Hz, 1H), 4.55 (s, 2H), 3.83 (dh, *J* = 13.5, 4.5 Hz, 1H), 1.77 (ddt, *J* = 14.8, 7.4, 3.3 Hz, 2H), 1.63 – 1.40 (m, 5H), 1.33 (s, 12H). LC-MS (ESI): m/z = 424.3, t_R = 5.59 min.

Methyl 3-(7-(2-(cycloheptylamino)-2-oxoethoxy)naphthalen-2-yl)-3-phenylpropanoate (12-I3). The reaction was conducted following the general procedure **F**, using **12-I1** (0.29 g, 0.71 mmol) and methyl cinnamate (**12-I2**, 0.08 g, 0.48 mmol), TEA (0.22 mL, 1.42 mmol), and [Rh(COD)Cl]$_2$ (24 mg, 0.05 mmol) to obtain 0.16 g (51.7%) of **12-I3** as a white solid. ^1H NMR (600 MHz, DMSO-d_6) δ 7.95 (d, *J* = 8.1 Hz, 1H), 7.78 –

7.74 (m, 1H), 7.72 (d, *J* = 8.4 Hz, 1H), 7.67 (d, *J* = 1.7 Hz, 1H), 7.38 – 7.33 (m, 2H), 7.32 – 7.25 (m, 3H), 7.20 – 7.13 (m, 3H), 4.58 (t, *J* = 8.0 Hz, 1H), 4.53 (s, 2H), 3.91 (s, 0H), 3.87 – 3.78 (m, 1H), 3.49 (s, 3H), 3.22 (qd, *J* = 15.9, 8.0 Hz, 2H), 1.75 (ddt, *J* = 14.0, 7.3, 2.5 Hz, 2H), 1.63 – 1.32 (m, 10H). LC-MS (ESI): m/z = 460.4, t_R = 5.19 min.

3-(7-(2-(Cycloheptylamino)-2-oxoethoxy)naphthalen-2-yl)-3-phenylpropanoic acid (12). The reaction was conducted following the general procedure **G**, using **12-I3** (0.16 g, 0.38 mmol) and 2 M aq. NaOH (380 µL) to obtain 0.035 g (20.7%) of **12** as a white solid. ^{1}H NMR (600 MHz, DMSO-d_6) δ 12.12 (s, 1H), 7.96 (d, *J* = 8.1 Hz, 1H), 7.79 – 7.75 (m, 1H), 7.73 (d, *J* = 8.5 Hz, 1H), 7.68 (d, *J* = 1.8 Hz, 1H), 7.38 – 7.33 (m, 2H), 7.33 – 7.25 (m, 3H), 7.21 – 7.14 (m, 3H), 4.55 (d, *J* = 9.2 Hz, 3H), 3.83 (dh, *J* = 13.5, 4.5 Hz, 1H), 3.11 (qd, *J* = 15.9, 8.0 Hz, 2H), 1.77 (dddd, *J* = 14.4, 7.2, 4.9, 2.4 Hz, 2H), 1.65 – 1.35 (m, 9H), 1.25 (d, *J* = 5.0 Hz, 1H). ^{13}C NMR (151 MHz, DMSO-d_6) δ 173.16, 166.54, 156.39, 144.46, 142.76, 134.58, 129.40, 128.90, 128.88 (2C), 128.14, 128.08 (2C), 127.84, 126.75, 124.87, 118.74, 107.62, 67.53, 50.07, 47.21, 40.55, 34.68 (2C), 28.16 (2C), 24.22 (2C). LC-MS (ESI): m/z = 446.4, t_R = 4.689 min.

3-(7-(2-(Cycloheptylamino)-2-oxoethoxy)naphthalen-2-yl)-3-(4-methoxyphenyl)propanoic acid (13)

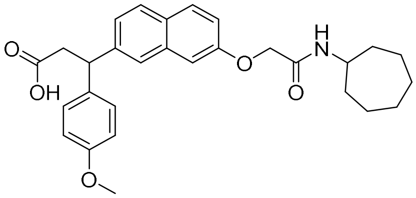

Methyl 3-(7-(2-(cycloheptylamino)-2-oxoethoxy)naphthalen-2-yl)-3-(4-methoxyphenyl)propanoate (13-I3). The reaction was conducted following the general procedure **F**, using **12-I1** (0.31 g, 0.76 mmol) and methyl (*E*)-3-(4-methoxyphenyl) acrylate (**13-I2**, 0.10 g, 0.51 mmol), TEA (0.23 mL, 1.52 mmol), and $[Rh(COD)Cl]_2$ (24 mg, 0.05 mmol) to obtain 0.14 g (39.7%) of **13-I3** as a white solid. ^{1}H NMR (400 MHz, DMSO-d_6) δ 7.96 (d, *J* = 8.1 Hz, 1H), 7.80 – 7.69 (m, 2H), 7.67 – 7.62 (m, 1H), 7.32 – 7.22 (m, 3H), 7.22 – 7.15 (m, 2H), 6.88 – 6.79 (m, 2H), 4.54 (d, *J* = 5.0 Hz, 3H), 3.84 (qd, *J* = 9.0, 8.4, 3.9 Hz, 1H), 3.70 (s, 3H), 3.50 (s, 3H), 3.26 – 3.09 (m, 2H), 1.77 (dt, *J* = 14.4, 5.2 Hz, 2H), 1.67 – 1.33 (m, 10H). LC-MS (ESI): m/z = 490.3, t_R = 5.23 min.

3-(7-(2-(Cycloheptylamino)-2-oxoethoxy)naphthalen-2-yl)-3-(4-methoxyphenyl)propanoic acid (13). The reaction was conducted following the general procedure **G**, using **13-I3** (0.14 g, 0.31 mmol) and 2 M aq. NaOH (310 µL) to obtain 0.07 g (50.7%) of **13** as a white solid. ^{1}H NMR (600 MHz, DMSO-d_6) δ 12.09 (s, 1H), 7.96 (d, *J* = 8.1 Hz, 1H), 7.80 – 7.69 (m, 2H), 7.64 (d, *J* = 1.7 Hz, 1H), 7.29 – 7.23 (m, 3H), 7.21 – 7.16 (m, 2H), 6.87 – 6.81 (m, 2H), 4.54 (s, 2H), 4.50 (t, *J* = 8.0 Hz, 1H), 3.88 – 3.79 (m, 1H), 3.70 (s, 3H), 3.13 – 2.99 (m, 2H), 1.77 (dddd, *J* = 13.4, 6.6, 4.3, 2.2 Hz, 2H), 1.66 – 1.35 (m, 10H). ^{13}C NMR (151 MHz, DMSO-d_6) δ 173.20, 166.53, 158.16, 156.37, 143.15, 136.42, 134.58, 129.38, 129.06 (2C), 128.09, 127.79, 124.87, 124.68, 118.67, 114.26 (2C), 107.61, 67.53, 55.46, 50.05, 46.40, 40.49, 34.68 (2C), 28.16 (2C), 24.22 (2C). LC-MS (ESI): m/z = 476.3, t_R = 4.81 min.

3-(7-(2-(Cycloheptylamino)-2-oxoethoxy)naphthalen-2-yl)-3-(3-methoxyphenyl)propanoic acid (14)

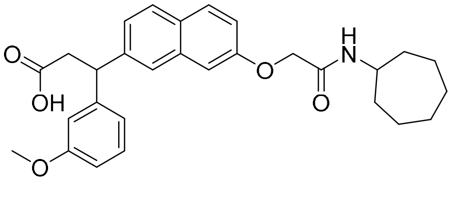

Methyl (*E*)-3-(3-methoxyphenyl)acrylate (14-I2). The reaction was conducted following the general procedure **C**, using 1-bromo-3-methoxybenzene (**14-I1**, 0.5 g, 2.67 mmol), methyl acrylate (0.37 mL, 4.01 mmol), $Pd(OAc)_2$ (0.03 g, 0.13 mmol), tri(*o*-tolyl)phosphine (0.08 g, 0.28 mmol), and Et_3N (2.7 mL, 19.17 mmol) to obtain 0.5 g (97.5%) of **14-I2** as yellow oil. ^1H NMR (600 MHz, DMSO-d_6) δ 7.63 (d, *J* = 16.0 Hz, 1H), 7.36 – 7.26 (m, 3H), 7.00 (ddd, *J* = 8.1, 2.6, 1.0 Hz, 1H), 6.67 (d, *J* = 16.0 Hz, 1H), 3.79 (s, 3H), 3.73 (s, 3H). LC-MS (ESI): m/z = 193.1, t_R = 4.52 min.

Methyl 3-(7-(2-(cycloheptylamino)-2-oxoethoxy)naphthalen-2-yl)-3-(3-methoxyphenyl)propanoate (14-I3). The reaction was conducted following the general procedure **F**, using **12-I1** (0.30 g, 0.73 mmol) and **14-I2** (0.08 mL, 0.49 mmol), TEA (0.21 mL, 1.47 mmol), and $[Rh(COD)Cl]_2$(25 mg, 0.05 mmol) to obtain 0.17 g (48.9%) of **14-I3** as colorless oil. ^1H NMR (600 MHz, DMSO-d_6) δ 7.95 (d, *J* = 8.1 Hz, 1H), 7.78 – 7.74 (m, 1H), 7.72 (d, *J* = 8.5 Hz, 1H), 7.67 (d, *J* = 1.7 Hz, 1H), 7.32 (dd, *J* = 8.5, 1.8 Hz, 1H), 7.18 (dt, *J* = 6.9, 4.4 Hz, 3H), 6.91 (dd, *J* = 7.4, 1.4 Hz, 2H), 6.74 (dt, *J* = 8.2, 1.3 Hz, 1H), 4.54 (d, *J* = 6.0 Hz, 3H), 3.82 (dh, *J* = 13.6, 4.4 Hz, 1H), 3.70 (s, 3H), 3.49 (s, 3H), 3.20 (qd, *J* = 16.0, 8.0 Hz, 2H), 1.75 (dddd, *J* = 14.2, 7.0, 4.7, 2.3 Hz, 2H), 1.65 – 1.35 (m, 10H). LC-MS (ESI): m/z = 490.3, t_R = 5.27 min.

3-(7-(2-(Cycloheptylamino)-2-oxoethoxy)naphthalen-2-yl)-3-(3-methoxyphenyl)propanoic acid (14). The reaction was conducted following the general procedure **G**, using **14-I3** (0.17 g, 0.36 mmol) and 2 M aq. NaOH (360 µL) to obtain 0.13 g (61.4%) of **14** as a white solid. ^1H NMR (600 MHz, DMSO-d_6) δ 12.11 (s, 1H), 7.95 (d, *J* = 8.1 Hz, 1H), 7.78 – 7.70 (m, 2H), 7.67 (d, *J* = 1.7 Hz, 1H), 7.31 (dd, *J* = 8.4, 1.8 Hz, 1H), 7.18 (dd, *J* = 7.7, 3.1 Hz, 3H), 6.91 (dd, *J* = 5.0, 3.1 Hz, 2H), 6.76 – 6.71 (m, 1H), 4.53 (d, *J* = 11.0 Hz, 3H), 3.83 (dh, *J* = 13.5, 4.4 Hz, 1H), 3.70 (s, 3H), 3.08 (qd, *J* = 15.9, 7.9 Hz, 2H), 1.80 – 1.72 (m, 2H), 1.65 – 1.17 (m, 10H). ^{13}C NMR (151 MHz, DMSO-d_6) δ 173.15, 166.53, 159.75, 156.37, 146.03, 142.64, 134.57, 129.90, 129.39, 128.10, 127.85, 124.87 (2C), 120.31, 118.74, 114.17, 111.77, 107.62, 67.54, 55.43, 50.05, 47.16, 40.55, 34.68 (2C), 28.16 (2C), 24.22 (2C). LC-MS (ESI): m/z = 476.3, t_R = 4.86 min.

3-(4-Chlorophenyl)-3-(7-(2-(cycloheptylamino)-2-oxoethoxy)naphthalen-2-yl)propanoic acid(15)

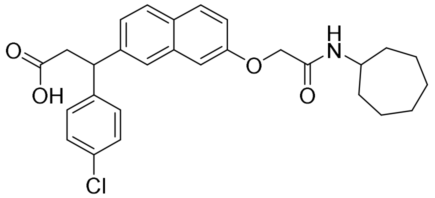

Methyl (*E*)-3-(4-chlorophenyl)acrylate (15-I2). The reaction was conducted following the general procedure **C**, using 1-bromo-4-chlorobenzene (**15-I1**, 0.5 g, 2.62 mmol), methyl acrylate (0.36 mL, 3.92 mmol), $Pd(OAc)_2$ (0.03 g, 0.13 mmol), tri(*o*-tolyl)phosphine (0.08 g, 0.27 mmol), and Et_3N (2.7 mL, 18.73 mmol) to obtain 0.41 g (79.2%) of **15-I2** as a white solid. ^1H NMR (400 MHz, DMSO-d_6) δ 7.81 – 7.73 (m, 2H), 7.66 (d, *J* = 16.1 Hz, 1H), 7.53 – 7.45 (m, 2H), 6.68 (d, *J* = 16.1 Hz, 1H), 3.73 (s, 3H). LC-MS (ESI): m/z = 197.0, t_R = 4.82 min.

Methyl 3-(4-chlorophenyl)-3-(7-(2-(cycloheptylamino)-2-oxoethoxy)naphthalen-2-yl)propanoate (15-I3). The reaction was conducted following the general procedure **F**, using **12-I1** (0.25 g, 0.61 mmol) and **15-I2** (0.08 g, 0.41 mmol), TEA (0.18 mL, 1.23 mmol), and $[Rh(COD)Cl]_2$ (25 mg, 0.05 mmol) to obtain 0.21 g (69.2%) of **15-I3** as colorless oil. ^1H NMR (600 MHz, DMSO-d_6) δ 7.96 (d, *J* = 8.1 Hz, 1H), 7.77 (d, *J* = 8.8 Hz, 1H), 7.74 (d, *J* = 8.5 Hz, 1H), 7.67 (d, *J* = 1.7 Hz, 1H), 7.43 – 7.37 (m, 2H), 7.36 – 7.29 (m, 3H), 7.22 – 7.16 (m, 2H), 4.61 (t, *J* = 7.9 Hz, 1H), 4.55 (s, 2H), 3.83 (ddq, *J* = 13.5, 9.1, 4.4 Hz, 1H), 3.50 (s, 3H), 3.29 – 3.18 (m, 2H), 1.81 – 1.72 (m, 2H), 1.67 – 1.34 (m, 10H). LC-MS (ESI): m/z = 494.3, t_R = 5.48 min.

3-(4-Chlorophenyl)-3-(7-(2-(cycloheptylamino)-2-oxoethoxy)naphthalen-2-yl)propanoic acid (15). The reaction was conducted following the general procedure **G**, using **15-I3** (0.21 g, 0.43 mmol) and 2 M aq. NaOH (430 µL) to obtain 0.025 g (12.3%) of **15** as a white solid. ^{1}H NMR (600 MHz, DMSO-*d$_6$*) δ 12.16 (s, 1H), 7.96 (d, *J* = 8.1 Hz, 1H), 7.79 – 7.72 (m, 2H), 7.66 (d, *J* = 1.7 Hz, 1H), 7.41 – 7.29 (m, 5H), 7.19 (d, *J* = 8.5 Hz, 2H), 4.57 (t, *J* = 8.0 Hz, 1H), 4.54 (s, 2H), 3.87 – 3.79 (m, 1H), 3.16 – 3.05 (m, 2H), 1.80 – 1.72 (m, 2H), 1.63 – 1.13 (m, 10H) ^{13}C NMR (151 MHz, DMSO-*d$_6$*) δ 173.03, 166.51, 156.43, 143.49, 142.31, 134.58, 131.38, 130.02 (2C), 129.41, 128.79 (2C), 128.26, 127.88, 124.91, 124.75, 118.85, 107.63, 67.53, 50.06, 46.49, 40.50, 34.69 (2C), 28.16 (2C), 24.23 (2C). LC-MS (ESI): m/z = 480.2, t$_R$ = 5.10 min.

3-(3-Chlorophenyl)-3-(7-(2-(cycloheptylamino)-2-oxoethoxy)naphthalen-2-yl)propanoic acid (16)

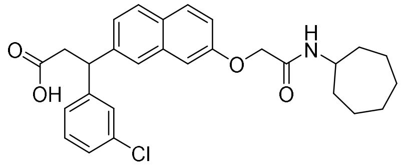

Methyl (*E*)-3-(3-chlorophenyl)acrylate (16-I2). The reaction was conducted following the general procedure **C**, using 1-bromo-3-chlorobenzene (**16-I1**, 0.5 g, 2.62 mmol), methyl acrylate (0.36 mL, 3.91 mmol), Pd(OAc)$_2$ (0.03 g, 0.13 mmol), tri(*o*-tolyl)phosphine (0.08 g, 0.27 mmol), and Et$_3$N (2.7 mL, 18.73 mmol) to obtain 0.49 g (95.5%) of **16-I2** as light yellow oil. ^{1}H NMR (400 MHz, DMSO-*d$_6$*) δ 7.83 (t, *J* = 1.8 Hz, 1H), 7.73 – 7.60 (m, 2H), 7.50 – 7.39 (m, 2H), 6.72 (d, *J* = 16.1 Hz, 1H), 3.73 (s, 3H). LC-MS (ESI): m/z = 197.1, t$_R$ = 4.82 min.

Methyl 3-(3-chlorophenyl)-3-(7-(2-(cycloheptylamino)-2-oxoethoxy)naphthalen-2-yl)propanoate (16-I3). The reaction was conducted following the general procedure **F**, using **12-I1** (0.25 g, 0.61 mmol) and **16-I2** (0.07 mL, 0.41 mmol), TEA (0.18 mL, 1.23 mmol), and [Rh(COD)Cl]$_2$ (21 mg, 0.04 mmol) to obtain 0.21 g (72.0%) of **16-I3** as light yellow oil. ^{1}H NMR (600 MHz, DMSO-*d$_6$*) δ 7.96 (d, *J* = 8.1 Hz, 1H), 7.76 (dd, *J* = 17.2, 8.6 Hz, 2H), 7.71 (d, *J* = 1.8 Hz, 1H), 7.45 (t, *J* = 2.0 Hz, 1H), 7.38 – 7.28 (m, 3H), 7.26 – 7.16 (m, 3H), 4.61 (t, *J* = 8.0 Hz, 1H), 4.55 (s, 2H), 3.83 (dh, *J* = 13.5, 4.4 Hz, 1H), 3.50 (s, 3H), 3.26 (dd, *J* = 8.0, 1.3 Hz, 2H), 1.80 – 1.72 (m, 2H), 1.64 – 1.36 (m, 10H). LC-MS (ESI): m/z = 494.3, t$_R$ = 5.47 min.

3-(3-Chlorophenyl)-3-(7-(2-(cycloheptylamino)-2-oxoethoxy)naphthalen-2-yl)propanoic acid (16). The reaction was conducted following the general procedure **G**, using **16-I3** (0.21 g, 0.43 mmol) and 2 M aq. NaOH (240 µL) to obtain 0.035 g (17.2%) of **16** as a white solid. ^{1}H NMR (600 MHz, DMSO-*d$_6$*) δ 7.97 (d, *J* = 8.1 Hz, 1H), 7.80 – 7.73 (m, 2H), 7.71 (d, *J* = 1.8 Hz, 1H), 7.44 (t, *J* = 1.9 Hz, 1H), 7.36 – 7.29 (m, 2H), 7.20 (d, *J* = 7.4 Hz, 2H), 4.59 (t, *J* = 8.0 Hz, 1H), 4.55 (s, 2H), 3.88 – 3.79 (m, 1H), 3.14 (d, *J* = 8.0 Hz, 2H), 1.77 (ddq, *J* = 14.0, 7.0, 2.2 Hz, 2H), 1.66 – 1.20 (m, 10H). ^{13}C NMR (151 MHz, DMSO-*d$_6$*) δ 173.02, 166.52, 156.44, 147.09, 142.11, 134.59, 133.50, 130.75, 129.43, 128.29, 127.98, 127.92, 126.85, 126.77, 124.98, 124.74, 118.89, 107.64, 67.54, 50.07, 46.79, 40.49, 34.68 (2C), 28.15 (2C), 24.23 (2C). LC-MS (ESI): m/z = 480.2, t$_R$ = 5.10 min.

3-(7-(2-(Cycloheptylamino)-2-oxoethoxy)naphthalen-2-yl)-3-(4-methoxy-2-methylphenyl)propanoic acid (17).

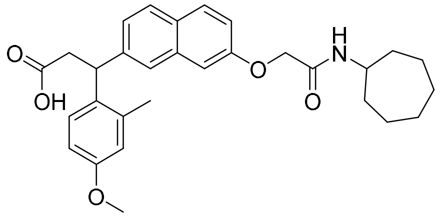

Methyl (*E*)-3-(4-methoxy-2-methylphenyl)acrylate (17-I2). The reaction was conducted following the general procedure **C**, using 1-bromo-4-methoxy-2-methylbenzene (**17-I1**, 0.5 g, 2.49 mmol), methyl acrylate (0.34 mL, 3.73 mmol), $Pd(OAc)_2$ (0.03 g, 0.13 mmol), tri(*o*-tolyl)phosphine (0.08 g, 0.25 mmol), and Et_3N (2.5 mL, 17.83 mmol) to obtain 0.31 g (60.8%) of **17-I2** as a white solid. ^{1}H NMR (400 MHz, DMSO-d_6) δ 7.81 (d, *J* = 15.9 Hz, 1H), 7.71 (d, *J* = 8.6 Hz, 1H), 6.88 – 6.77 (m, 2H), 6.41 (d, *J* = 15.8 Hz, 1H), 3.78 (s, 3H), 3.71 (s, 3H), 2.38 (s, 3H).LC-MS (ESI): m/z = 207.1, t_R = 4.67 min.

Methyl 3-(7-(2-(cycloheptylamino)-2-oxoethoxy)naphthalen-2-yl)-3-(4-methoxy-2-methylphenyl)propanoate (I7-I3). The reaction was conducted following the general procedure **F**, using **12-I1** (0.30 g, 0.73 mmol) and **17-I2** (0.10 g, 0.49 mmol), TEA (0.21 mL, 1.47 mmol), and $[Rh(COD)Cl]_2$ (25 mg, 0.05 mmol) to obtain 0.21 g (58.1%) of **17-I3** as colorless oil. ^{1}H NMR (600 MHz, DMSO-d_6) δ 7.96 (d, *J* = 8.1 Hz, 1H), 7.76 (d, *J* = 8.9 Hz, 1H), 7.72 (d, *J* = 8.5 Hz, 1H), 7.55 – 7.51 (m, 1H), 7.28 (d, *J* = 8.5 Hz, 1H), 7.24 (dd, *J* = 8.5, 1.8 Hz, 1H), 7.20 – 7.14 (m, 2H), 6.75 (dd, *J* = 8.5, 2.8 Hz, 1H), 6.72 (d, *J* = 2.8 Hz, 1H), 4.70 (t, *J* = 7.9 Hz, 1H), 4.54 (s, 2H), 3.87 – 3.78 (m, 1H), 3.71 (s, 3H), 3.51 (s, 3H), 3.18 – 3.07 (m, 2H), 2.27 (s, 3H), 1.80 – 1.71 (m, 2H), 1.65 – 1.33 (m, 10H). LC-MS (ESI): m/z = 504.3, t_R = 5.37 min.

3-(7-(2-(Cycloheptylamino)-2-oxoethoxy)naphthalen-2-yl)-3-(4-methoxy-2-methylphenyl)propanoic acid (17). The reaction was conducted following the general procedure **G**, using **17-I3** (0.21 g, 0.36 mmol) and 2M aq. NaOH (360 µL) to obtain 0.077 g (46.7%) of **17** as a white solid. ^{1}H NMR (600 MHz, DMSO-d_6) δ 12.11 (s, 1H), 7.96 (d, *J* = 8.1 Hz, 1H), 7.76 (d, *J* = 8.8 Hz, 1H), 7.72 (d, *J* = 8.5 Hz, 1H), 7.53 (d, *J* = 1.8 Hz, 1H), 7.29 – 7.22 (m, 2H), 7.20 – 7.14 (m, 2H), 6.76 (dd, *J* = 8.5, 2.8 Hz, 1H), 6.72 (d, *J* = 2.7 Hz, 1H), 4.68 (t, *J* = 7.9 Hz, 1H), 4.54 (s, 2H), 3.87 – 3.78 (m, 1H), 3.71 (s, 3H), 3.00 (h, *J* = 8.1 Hz, 2H), 2.27 (s, 3H), 1.80 – 1.72 (m, 2H), 1.65 – 1.435 (m, 10H) ^{13}C NMR (151 MHz, DMSO-d_6) δ 173.23, 166.54, 157.89, 156.36, 142.59, 137.51, 134.48, 134.20, 129.36, 128.06, 127.99, 127.73, 125.05 (2C), 118.72, 116.39, 111.63, 107.57, 67.51, 55.34, 50.01, 42.37, 40.55, 34.67 (2C), 28.17 (2C), 24.21 (2C), 20.07. LC-MS (ESI): m/z = 490.3, t_R = 4.95 min.

3-(3-Chloro-2-methylphenyl)-3-(7-(2-(cycloheptylamino)-2-oxoethoxy)naphthalen-2-yl)propanoic acid (18)

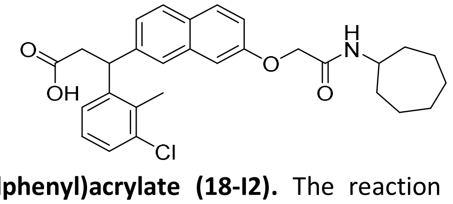

Methyl (*E*)-3-(3-chloro-2-methylphenyl)acrylate (18-I2). The reaction was conducted following the general procedure **C**, using 1-bromo-3-chloro-2-methylbenzene (**18-I1**, 1.0 g, 4.86 mmol), methyl acrylate (0.66 mL, 7.20 mmol), $Pd(OAc)_2$ (0.06 g, 0.26 mmol), tri(*o*-tolyl)phosphine (0.15 g, 0.50 mmol), and Et_3N (4.4 mL, 31.40 mmol) to obtain 0.79 g (79.0%) of **18-I2** as colorless oil. ^{1}H NMR (400 MHz, DMSO-d_6) δ 7.90 (d, *J* = 15.9 Hz, 1H), 7.68 (dd, *J* = 7.8, 1.2 Hz, 1H), 7.50 (dd, *J* = 8.1, 1.2 Hz, 1H), 7.28 (q, *J* = 7.1 Hz, 1H), 6.54 (d, *J* = 15.8 Hz, 1H), 3.75 (s, 3H), 2.42 (s, 3H). LC-MS (ESI): m/z = 211.1, t_R = 4.79 min.

Methyl 3-(3-chloro-2-methylphenyl)-3-(7-(2-(cycloheptylamino)-2-oxoethoxy)naphthalen-2-yl)propanoate (18-I3). The reaction was conducted following the general procedure **F**, using **12-I1** (0.19 g, 0.45 mmol) and **18-I2** (0.06 g, 0.30 mmol), TEA (0.19 mL, 1.35 mmol), and $[Rh(COD)Cl]_2$ (15 mg, 0.03 mmol) to obtain 0.12 g (52.9%) of **18-I3** as colorless oil. ^{1}H NMR (600 MHz, DMSO-d_6) δ 7.96 (d, J = 8.1 Hz, 1H), 7.76 (dd, J = 17.3, 8.7 Hz, 2H), 7.55 (d, J = 1.7 Hz, 1H), 7.38 (dd, J = 7.8, 1.2 Hz, 1H), 7.31 (dd, J = 8.1, 1.2 Hz, 1H), 7.29 – 7.18 (m, 3H), 7.16 (d, J = 2.6 Hz, 1H), 4.86 (t, J = 7.8 Hz, 1H), 4.54 (s, 2H), 3.82 (ddq, J = 13.5, 9.1, 4.4 Hz, 1H), 3.52 (s, 3H), 3.19 (d, J = 7.9 Hz, 2H), 2.37 (s, 3H), 1.80 – 1.70 (m, 2H), 1.62 – 1.31 (m, 10H). LC-MS (ESI): m/z = 508.2, t_R = 5.31 min.

3-(3-Chloro-2-methylphenyl)-3-(7-(2-(cycloheptylamino)-2-oxoethoxy)naphthalen-2-yl)propanoic acid (18). The reaction was conducted following the general procedure **G**, using **18-I3** (0.12 g, 0.24 mmol) and 2M aq. NaOH (1 mL) to obtain 0.08 g (71%) of **18** as a white solid. ^{1}H NMR (600 MHz, DMSO-d_6) δ 12.23 (s, 1H), 7.96 (d, J = 8.1 Hz, 1H), 7.76 (dd, J = 17.6, 8.7 Hz, 2H), 7.55 (d, J = 1.7 Hz, 1H), 7.39 – 7.12 (m, 6H), 4.84 (t, J = 7.8 Hz, 1H), 4.54 (s, 2H), 3.82 (dh, J = 13.5, 4.4 Hz, 1H), 3.07 (d, J = 7.8 Hz, 2H), 2.62 (p, J = 1.9 Hz, 1H), 2.37 (s, 3H), 1.82 – 1.70 (m, 2H), 1.65 – 1.44 (m, 7H), 1.39 (dtt, J = 12.8, 9.8, 2.9 Hz, 2H). ^{13}C NMR (151 MHz, DMSO-d_6) δ 173.04, 166.54, 156.44, 144.60, 141.60, 134.54, 134.50, 134.01, 129.42, 128.30, 127.85, 127.66, 126.22, 125.30, 124.93, 118.95, 107.60, 67.50, 50.03, 43.63, 40.78, 40.54, 34.66 (2C), 28.16 (2C), 24.20 (2C), 16.26. LC-MS (ESI): m/z = 404.2, t_R = 4.94 min.

3-(Benzo[d][1,3]dioxol-5-yl)-3-(7-(2-(cycloheptylamino)-2-oxoethoxy)naphthalen-2-yl)propanoic acid (19)

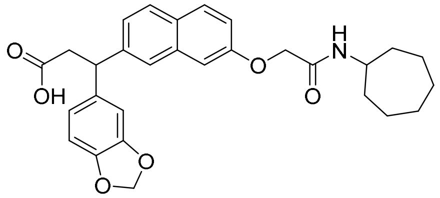

Methyl (*E*)-3-(benzo[d][1,3]dioxol-5-yl)acrylate (19-I2). The reaction was conducted following the general procedure **C**, using 5-bromobenzo[d][1,3]dioxole (**19-I1**, 0.3 mL, 2.49 mmol), methyl acrylate (0.40 mL, 3.73 mmol), $Pd(OAc)_2$ (0.03 g, 0.12 mmol), tri(*o*-tolyl)phosphine (0.08 g, 0.24 mmol), and Et_3N (2.5 mL, 17.83 mmol) to obtain 0.45 g (88.2%) of **19-I2** as a white solid. ^{1}H NMR (400 MHz, DMSO-d_6) δ 7.58 (d, J = 15.9 Hz, 1H), 7.41 (d, J = 1.7 Hz, 1H), 7.20 (dd, J = 8.0, 1.7 Hz, 1H), 6.95 (d, J = 8.0 Hz, 1H), 6.51 (d, J = 15.9 Hz, 1H), 6.08 (s, 2H), 3.70 (s, 3H). LC-MS (ESI): m/z = 207.1, t_R = 4.35 min.

Methyl 3-(benzo[d][1,3]dioxol-5-yl)-3-(7-(2-(cycloheptylamino)-2-oxoethoxy)naphthalen-2-yl)propanoate (19-I3). The reaction was conducted following the general procedure **F**, using **12-I1** (0.30 g, 0.73 mmol) and **19-I2** (0.10 g, 0.49 mmol), TEA (0.22 mL, 1.47 mmol), and $[Rh(COD)Cl]_2$ (15 mg, 0.03 mmol) to obtain 0.12 g (33.8%) of **19-I3** as colorless oil. ^{1}H NMR (400 MHz, DMSO-d_6) δ 7.95 (d, J = 8.1 Hz, 1H), 7.79 – 7.68 (m, 2H), 7.66 (d, J = 1.8 Hz, 1H), 7.30 (dd, J = 8.4, 1.7 Hz, 1H), 7.18 (d, J = 7.5 Hz, 2H), 6.95 (d, J = 1.6 Hz, 1H), 6.86 – 6.76 (m, 2H), 5.93 (dd, J = 4.2, 1.0 Hz, 2H), 4.52 (d, J = 13.6 Hz, 3H), 3.82 (qt, J = 9.0, 4.4 Hz, 1H), 3.49 (s, 3H), 3.26 – 3.09 (m, 2H), 1.81 – 1.70 (m, 2H), 1.66 – 1.32 (m, 10H). LC-MS (ESI): m/z = 504.2, t_R = 5.20 min.

3-(Benzo[d][1,3]dioxol-5-yl)-3-(7-(2-(cycloheptylamino)-2-oxoethoxy)naphthalen-2-yl)propanoic acid (19). The reaction was conducted following the general procedure **G**, using **19-I3** (0.16 g, 0.38 mmol) and 2M aq. NaOH (380 µL) to obtain 0.08 g (47.8%) of **19** as a white solid. ^{1}H NMR (600 MHz, DMSO-d_6) δ 12.11 (s, 1H), 7.96 (d, J = 8.1 Hz, 1H), 7.76 (d, J = 9.6 Hz, 1H), 7.72 (d, J = 8.5 Hz, 1H), 7.66 (d, J = 1.7 Hz, 1H), 7.30 (dd, J = 8.5, 1.8 Hz, 1H), 7.18 (dq, J = 5.3, 2.5 Hz, 2H), 6.95 (d, J = 1.6 Hz, 1H), 6.84 – 6.77 (m, 2H), 5.93 (dd, J

= 5.3, 1.0 Hz, 2H), 4.54 (s, 2H), 4.48 (t, *J* = 8.0 Hz, 1H), 3.83 (ddt, *J* = 13.4, 9.1, 4.6 Hz, 1H), 3.12 – 2.98 (m, 2H), 1.76 (dddt, *J* = 15.5, 7.9, 3.7, 1.7 Hz, 2H), 1.65 – 1.42 (m, 8H), 1.44 – 1.35 (m, 2H). ^{13}C NMR (151 MHz, DMSO-d_6) δ 173.17, 166.54, 156.37, 147.74, 146.04, 142.93, 138.46, 134.58, 129.38, 128.10, 127.83, 124.82, 124.69, 121.05, 118.71, 108.55, 108.52, 107.63, 101.24, 67.54, 50.06, 46.87, 40.41, 34.68 (2C), 28.15 (2C), 24.22 (2C). LC-MS (ESI): m/z = 490.2, t_R = 4.79 min.

3-(7-(2-(Cycloheptylamino)-2-oxoethoxy) naphthalen-2-yl)-3-(2,3-dihydrobenzofuran-5-yl)propanoic acid (20)

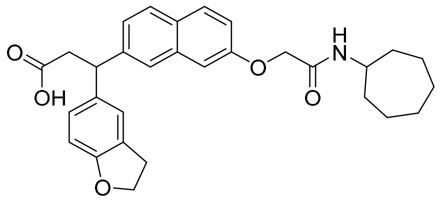

Methyl (*E*)-3-(2,3-dihydrobenzofuran-5-yl)acrylate (20-I2). The reaction was conducted following the general procedure **C**, using 5-bromo-2,3-dihydrobenzofuran (**20-I1**, 0.50 g, 2.52 mmol), methyl acrylate (0.40 mL, 3.77 mmol), $Pd(OAc)_2$ (0.03 g, 0.12 mmol), tri(*o*-tolyl)phosphine (0.08 g, 0.24 mmol), and Et_3N (2.5 mL, 17.83 mmol) to obtain 0.12 g (23.5%) of **20-I2** as a white solid. ^{1}H NMR (600 MHz, DMSO-d_6) δ 7.64 (q, *J* = 1.4 Hz, 1H), 7.59 (d, *J* = 16.0 Hz, 1H), 7.45 (dd, *J* = 8.2, 1.9 Hz, 1H), 6.79 (d, *J* = 8.2 Hz, 1H), 6.43 (d, *J* = 16.0 Hz, 1H), 4.58 (t, *J* = 8.7 Hz, 2H), 3.69 (s, 3H), 3.19 (t, *J* = 8.7 Hz, 2H). LC-MS (ESI): m/z = 205.1, t_R = 4.72 min.

Methyl 3-(7-(2-(cycloheptylamino)-2-oxoethoxy)naphthalen-2-yl)-3-(2,3-dihydrobenzofuran-5-yl)propanoate (20-I3). The reaction was conducted following the general procedure **F**, using **12-I1** (0.03 g, 0.74 mmol) and **20-I2** (0.1 g, 0.49 mmol), TEA (0.21 mL, 1.47 mmol), and $[Rh(COD)Cl]_2$ (25 mg, 0.05 mmol) to obtain 0.05 g (21.7%) of **20-I3** as colorless oil. ^{1}H NMR (600 MHz, DMSO-d_6) δ 7.96 (d, *J* = 8.1 Hz, 1H), 7.76 (d, *J* = 9.6 Hz, 1H), 7.72 (d, *J* = 8.4 Hz, 1H), 7.67 – 7.63 (m, 1H), 7.29 (dd, *J* = 8.4, 1.8 Hz, 1H), 7.18 (dq, *J* = 4.4, 2.6 Hz, 3H), 7.07 (dd, *J* = 8.4, 2.0 Hz, 1H), 6.65 (d, *J* = 8.2 Hz, 1H), 4.55 (s, 2H), 4.51 (t, *J* = 8.0 Hz, 1H), 4.45 (t, *J* = 8.7 Hz, 2H), 3.83 (ddq, *J* = 13.5, 9.1, 4.5 Hz, 1H), 3.50 (s, 3H), 3.20 (dd, *J* = 15.8, 8.2 Hz, 1H), 3.12 (dt, *J* = 22.2, 8.3 Hz, 3H), 1.80 – 1.73 (m, 2H), 1.65 – 1.22 (m, 10H). LC-MS (ESI): m/z = 502.3, t_R = 5.45 min.

3-(7-(2-(cycloheptylamino)-2-oxoethoxy) naphthalen-2-yl)-3-(2,3-dihydrobenzofuran-5-yl)propanoic acid (20). The reaction was conducted following the general procedure **G**, using **20-I3** (0.05 g, 0.11 mmol) and 2 M aq. NaOH (110 µL) to obtain 0.03 g (51.0%) of **20** as a white solid. ^{1}H NMR (600 MHz, DMSO-d_6) δ 12.09 (s, 1H), 7.96 (d, *J* = 8.1 Hz, 1H), 7.75 (d, *J* = 8.6 Hz, 1H), 7.71 (d, *J* = 8.5 Hz, 1H), 7.64 (d, *J* = 1.7 Hz, 1H), 7.28 (dd, *J* = 8.5, 1.8 Hz, 1H), 7.20 – 7.15 (m, 3H), 7.06 (dd, *J* = 8.3, 1.9 Hz, 1H), 6.64 (d, *J* = 8.2 Hz, 1H), 4.54 (s, 2H), 4.51 – 4.41 (m, 3H), 3.88 – 3.79 (m, 1H), 3.12 – 3.04 (m, 3H), 3.01 (dd, *J* = 15.7, 7.9 Hz, 1H), 1.80 – 1.72 (m, 2H), 1.65 – 1.35 (m, 10H). ^{13}C NMR (151 MHz, DMSO-d_6) δ 171.12, 164.44, 156.53, 154.25, 141.17, 134.33, 132.48, 127.26, 125.96, 125.70, 125.67, 125.26, 122.81, 122.60, 122.48, 116.53, 106.89, 105.52, 69.16, 65.42, 47.95, 44.57, 38.55, 32.57 (2C), 27.47, 26.04 (2C), 22.11 (2C). LC-MS (ESI): m/z = 488.3, t_R = 5.13 min.

3-(Benzofuran-5-yl)-3-(7-(2-(cycloheptylamino)-2-oxoethoxy) naphthalen-2-yl)propanoic acid (21)

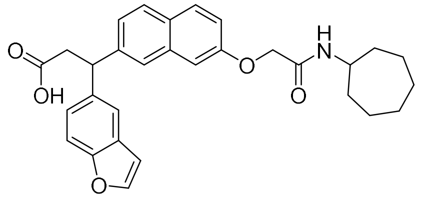

Methyl (*E*)-3-(benzofuran-5-yl) acrylate (21-I2). The reaction was conducted following the general procedure **C**, using 5-bromobenzofuran (**21-I1**, 0.5 g, 2.54 mmol), methyl acrylate (0.33 mL, 3.81 mmol), $Pd(OAc)_2$ (0.03 g, 0.13 mmol), tri(*o*-tolyl)phosphine (0.08 g, 0.26 mmol), and Et_3N (2.6 mL, 18.2 mmol) to obtain 0.50 g (97.5%) as a white solid. ^{1}H NMR (600 MHz, DMSO-d_6) δ 8.05 (d, *J* = 2.2 Hz, 1H), 8.03 (d, *J* = 1.8 Hz, 1H), 7.78 (d, *J* = 16.0 Hz, 1H), 7.72 (dd, *J* = 8.6, 1.8 Hz, 1H), 7.64 (d, *J* = 8.6 Hz, 1H), 7.00 (dd, *J* = 2.2, 1.0 Hz, 1H), 6.64 (d, *J* = 16.0 Hz, 1H), 3.73 (s, 3H). LC-MS (ESI): m/z = 203.1, t_R = 4.93 min.

Methyl 3-(benzofuran-5-yl)-3-(7-(2-(cycloheptylamino)-2-oxoethoxy) naphthalen-2-yl)propanoate (21-I3). The reaction was conducted following the general procedure **F**, using **12-I1** (0.30 g, 0.74 mmol) and **21-I2** (0.1 g, 0.49 mmol), TEA (0.21 mL, 1.47 mmol), and $[Rh(COD)Cl]_2$ (25 mg, 0.05 mmol) to obtain 0.23 g (92.1%) of **21-I3** as a white solid. ^{1}H NMR (600 MHz, DMSO-d_6) δ 7.98 – 7.91 (m, 2H), 7.79 – 7.74 (m, 1H), 7.73 (d, *J* = 8.5 Hz, 1H), 7.71 – 7.68 (m, 1H), 7.64 (d, *J* = 1.8 Hz, 1H), 7.48 (d, *J* = 8.5 Hz, 1H), 7.36 – 7.24 (m, 2H), 7.19 (dq, *J* = 5.0, 2.5 Hz, 2H), 6.90 (dd, *J* = 2.2, 1.0 Hz, 1H), 4.71 (t, *J* = 8.0 Hz, 1H), 4.54 (s, 2H), 3.83 (tq, *J* = 12.7, 4.5 Hz, 1H), 3.50 (s, 3H), 3.30 – 3.23 (m, 2H), 1.76 (dtd, *J* = 13.6, 7.3, 3.4 Hz, 2H), 1.66 – 1.26 (m, 10H). LC-MS (ESI): m/z = 500.2, t_R = 6.57 min.

3-(Benzofuran-5-yl)-3-(7-(2-(cycloheptylamino)-2-oxoethoxy) naphthalen-2-yl)propanoic acid (21). The reaction was conducted following the general procedure **G**, using **21-I3** (0.23 g, 0.47 mmol) and 2 M aq. NaOH (470 µL) to obtain 0.027 g (12.1%) of **21** as a white solid. ^{1}H NMR (600 MHz, DMSO-d_6) δ 12.12 (s, 1H), 7.98 – 7.93 (m, 2H), 7.76 (d, *J* = 8.7 Hz, 1H), 7.74 – 7.68 (m, 2H), 7.63 (d, *J* = 1.8 Hz, 1H), 7.48 (d, *J* = 8.6 Hz, 1H), 7.35 – 7.27 (m, 2H), 7.19 (d, *J* = 8.2 Hz, 2H), 6.90 (dd, *J* = 2.2, 1.0 Hz, 1H), 4.68 (t, *J* = 7.9 Hz, 1H), 4.54 (s, 2H), 3.83 (dh, *J* = 13.5, 4.5 Hz, 1H), 3.21 – 3.10 (m, 2H), 1.76 (ddt, *J* = 11.0, 7.5, 3.7 Hz, 2H), 1.66 – 1.19 (m, 10H). ^{13}C NMR (151 MHz, DMSO-d_6) δ 173.21, 166.52, 156.38, 153.49, 146.75, 143.14, 139.21, 134.59, 129.38, 128.12, 127.77 (2C), 125.48, 124.75, 124.14, 120.28, 118.72, 111.52, 107.64, 107.17, 67.53, 50.04, 47.03, 40.49, 34.68 (2C), 28.16 (2C), 24.22 (2C). LC-MS (ESI): m/z = 486.2, t_R = 5.24 min.

3-(7-(2-(Cycloheptylamino)-2-oxoethoxy) naphthalen-2-yl)-3-(2,2-difluorobenzo[d][1,3]dioxol-5-yl)propanoic acid (22)

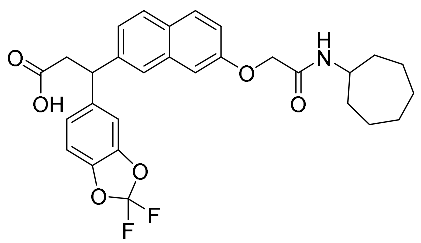

Methyl (*E*)-3-(2,2-difluorobenzo[d][1,3]dioxol-5-yl)acrylate (22-I2). The reaction was conducted following the general procedure **C**, using 5-bromo-2,2-difluorobenzo[d][1,3]dioxole (**22-I1**, 0.50 g, 2.12 mmol), methyl acrylate (0.30 mL, 3.18 mmol), $Pd(OAc)_2$ (0.02 g, 0.11 mmol), tri(*o*-tolyl)phosphine (0.07 g, 0.22 mmol), and Et_3N (2.2 mL, 15.2 mmol) to obtain 0.45 g (87.7%) of **22-I2** as a white solid. ^{1}H NMR (400 MHz, DMSO-d_6) δ 7.92 (d, *J* = 1.7 Hz, 1H), 7.67 (d, *J* = 16.0 Hz, 1H), 7.58 (dd, *J* = 8.4, 1.7 Hz, 1H), 7.46 (d, *J* = 8.3 Hz, 1H), 6.68 (d, *J* = 16.0 Hz, 1H), 3.72 (s, 3H). LC-MS (ESI): m/z = 243.0, t_R = 4.67 min.

Methyl 3-(7-(2-(cycloheptylamino)-2-oxoethoxy)naphthalen-2-yl)-3-(2,2-difluorobenzo[d][1,3]dioxol-5-yl)propanoate (22-I3). The reaction was conducted following the general procedure **F**, using **12-I1** (0.02 g, 0.45 mmol) and **22-I2** (0.07 g, 0.30 mmol), TEA (0.19 mL, 1.35 mmol), and $[Rh(COD)Cl]_2$ (15 mg, 0.03 mmol) to obtain 0.10 g (41.7%) of **22-I3** as colorless oil. ^{1}H NMR (400 MHz, DMSO-d_6) δ 7.96 (d, J = 8.1 Hz, 1H), 7.76 (dd, J = 11.6, 8.6 Hz, 2H), 7.70 (d, J = 1.8 Hz, 1H), 7.52 (d, J = 1.7 Hz, 1H), 7.35 (dd, J = 8.5, 1.8 Hz, 1H), 7.30 (d, J = 8.3 Hz, 1H), 7.27 – 7.15 (m, 3H), 4.65 (t, J = 8.0 Hz, 1H), 4.55 (s, 2H), 3.83 (dh, J = 13.3, 4.4 Hz, 1H), 3.51 (s, 3H), 3.27 (dd, J = 8.0, 4.7 Hz, 2H), 1.84 – 1.69 (m, 2H), 1.67 – 1.33 (m, 10H). LC-MS (ESI): m/z = 540.2, t_R = 5.30 min.

3-(7-(2-(Cycloheptylamino)-2-oxoethoxy) naphthalen-2-yl)-3-(2,2-difluorobenzo[d][1,3]dioxol-5-yl)propanoic acid (22). The reaction was conducted following the general procedure **G**, using **22-I3** (0.10 g, 0.18 mmol) and 2 M aq. NaOH (810 µL) to obtain 0.05 g (53.0%) of **22** as a white solid. ^{1}H NMR (600 MHz, DMSO-d_6) δ 12.18 (s, 1H), 7.96 (d, J = 8.1 Hz, 1H), 7.76 (dd, J = 18.1, 8.6 Hz, 2H), 7.70 (s, 1H), 7.51 (t, J = 1.4 Hz, 1H), 7.35 (dt, J = 8.5, 1.4 Hz, 1H), 7.30 (dd, J = 8.3, 1.0 Hz, 1H), 7.25 – 7.16 (m, 3H), 4.61 (t, J = 7.9 Hz, 1H), 4.55 (s, 2H), 3.83 (tp, J = 9.0, 4.4 Hz, 1H), 3.23 – 3.00 (m, 2H), 1.80 – 1.69 (m, 2H), 1.68 – 1.32 (m, 10H). ^{13}C NMR (151 MHz, DMSO-d_6) δ 173.02, 166.53, 156.44, 143.27, 142.28, 141.71, 141.63, 134.59, 131.63, 129.43, 128.29, 127.91, 124.90, 124.66, 124.12, 118.89, 110.29, 109.90, 107.63, 67.54, 50.06, 46.87, 34.68 (2C), 28.14 (2C), 24.22 (2C). LC-MS (ESI): m/z = 526.2, t_R = 4.87 min.

3-(7-(2-(Cycloheptylamino)-2-oxoethoxy) naphthalen-2-yl)-3-(2,3-dihydrobenzo[b][1,4]dioxin-6-yl)propanoic acid (23)

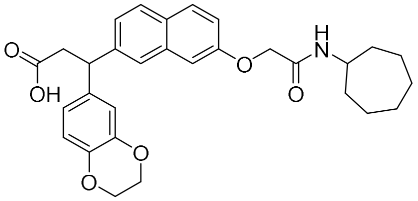

Methyl (*E*)-3-(2,3-dihydrobenzo[b][1,4]dioxin-6-yl)acrylate (23-I2). The reaction was conducted following the general procedure **C**, using 6-bromo-2,3-dihydrobenzo[b][1,4]dioxine (**23-I1**, 0.50 g, 2.33 mmol), methyl acrylate (0.32 mL, 3.49 mmol), $Pd(OAc)_2$ (0.03 g, 0.12 mmol), tri(*o*-tolyl)phosphine (0.07 g, 0.23 mmol), and Et_3N (2.4 mL, 16.7 mmol) to obtain 0.41 g (80.1%) of **23-I2** as a white solid. ^{1}H NMR (400 MHz, DMSO-d_6) δ 7.55 (d, J = 16.0 Hz, 1H), 7.27 (d, J = 2.1 Hz, 1H), 7.20 (dd, J = 8.4, 2.1 Hz, 1H), 6.89 (d, J = 8.3 Hz, 1H), 6.48 (d, J = 16.0 Hz, 1H), 4.32 – 4.22 (m, 4H), 3.70 (s, 3H). LC-MS (ESI): m/z = 221.1, t_R = 4.19 min.

Methyl 3-(7-(2-(cycloheptylamino)-2-oxoethoxy)naphthalen-2-yl)-3-(2,3-dihydrobenzo[b][1,4]dioxin-6-yl)propanoate (23-I3). The reaction was conducted following the general procedure F, using **12-I1** (0.03 g, 0.71 mmol) and **23-I2** (0.10 g, 0.47 mmol), TEA (0.30 mL, 2.1 mmol), and $[Rh(COD)Cl]_2$ (23 mg, 0.05 mmol) to obtain 0.12 g (32.7%) of **23-I3** as colorless oil. ^{1}H NMR (400 MHz, DMSO-d_6) δ 7.96 (d, J = 8.2 Hz, 1H), 7.74 (dd, J = 17.2, 9.1 Hz, 2H), 7.65 (s, 1H), 7.29 (dd, J = 8.4, 1.7 Hz, 1H), 7.22 – 7.16 (m, 2H), 6.87 – 6.70 (m, 3H), 4.55 (s, 2H), 4.47 (t, J = 8.0 Hz, 1H), 4.17 (s, 4H), 3.84 (ddq, J = 13.3, 9.0, 4.3 Hz, 1H), 3.50 (s, 3H), 3.24 – 3.05 (m, 2H), 1.83 – 1.70 (m, 2H), 1.68 – 1.33 (m, 10H). LC-MS (ESI): m/z = 518.2, t_R = 4.89 min.

3-(7-(2-(cycloheptylamino)-2-oxoethoxy) naphthalen-2-yl)-3-(2,3-dihydrobenzo[b][1,4]dioxin-6-yl)propanoic acid (23). The reaction was conducted following the general procedure **G**, using **23-I3** (0.12 g, 0.23 mmol) and 2 M aq. NaOH (1 mL) to obtain 0.06 g (51.2%) of **23** as a white solid. ^{1}H NMR (400 MHz, DMSO-d_6) δ 12.10 (s, 1H), 7.97 (d, J = 8.1 Hz, 1H), 7.78 – 7.75 (m, 1H), 7.72 (d, J = 8.5 Hz, 1H), 7.65 (d, J = 1.7

Hz, 1H), 7.29 (dd, *J* = 8.5, 1.8 Hz, 1H), 7.19 (d, *J* = 7.9 Hz, 2H), 6.83 (d, *J* = 2.1 Hz, 1H), 6.79 (dd, *J* = 8.4, 2.1 Hz, 1H), 6.74 (d, *J* = 8.3 Hz, 1H), 4.55 (s, 2H), 4.44 (t, *J* = 8.0 Hz, 1H), 4.17 (h, *J* = 3.3 Hz, 4H), 3.89 – 3.79 (m, 1H), 3.04 (qd, *J* = 15.8, 8.0 Hz, 2H), 1.77 (ddtd, *J* = 12.3, 6.2, 3.0, 1.5 Hz, 2H), 1.66 – 1.45 (m, 8H), 1.44 – 1.36 (m, 2H). ^{13}C NMR (151 MHz, DMSO-d_6) δ 171.06, 164.42, 154.24, 141.43, 140.84, 140.15, 135.46, 132.46, 127.26, 125.96, 125.70, 122.75, 122.56, 118.64, 116.57, 115.17, 114.41, 105.50, 65.42, 62.39, 62.29, 47.95, 44.35, 38.42, 32.57 (2C), 26.04 (2C), 22.11 (2C). LC-MS (ESI): m/z = 504.2, t_R = 4.52 min.

3-(7-(2-(Cycloheptylamino)-2-oxoethoxy)naphthalen-2-yl)-3-(6-methylbenzo[d][1,3]dioxol-5-yl)propanoic acid (24)

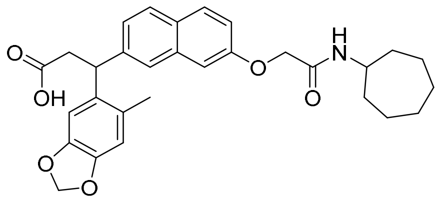

Methyl (*E*)-3-(6-methylbenzo[d][1,3]dioxol-5-yl)acrylate (24-I2). The reaction was conducted following the general procedure **C**, using 5-bromo-6-methylbenzo[d][1,3]dioxole (**24-I1**, 0.2 g, 0.93 mmol), methyl acrylate (0.13 mL, 1.40 mmol), $Pd(OAc)_2$ (0.01 g, 0.05 mmol), tri(*o*-tolyl)phosphine (0.03 g, 0.10 mmol), and Et_3N (0.93 mL, 6.67 mmol) to obtain 0.09 g (44.1%) of **24-I2** as a white solid. ^{1}H NMR (400 MHz, DMSO-d_6) δ 7.78 (d, *J* = 15.8 Hz, 1H), 7.37 (s, 1H), 6.87 (s, 1H), 6.46 (d, *J* = 15.7 Hz, 1H), 6.03 (s, 2H), 3.71 (s, 3H), 2.33 (s, 4H). LC-MS (ESI): m/z = 221.1, t_R = 4.50 min.

Methyl 3-(7-(2-(cycloheptylamino)-2-oxoethoxy)naphthalen-2-yl)-3-(6-methylbenzo[d][1,3]dioxol-5-yl)propanoate (24-I3). The reaction was conducted following the general procedure **F**, using **12-I1** (0.20 g, 0.47 mmol) and **24-I2** (0.07 g, 0.31 mmol), TEA (0.13 mL, 0.93 mmol), and $[Rh(COD)Cl]_2$ (16 mg, 0.03 mmol) to obtain 0.10 g (65.0%) of **24-I3** as colorless oil. ^{1}H NMR (600 MHz, DMSO-d_6) δ 7.96 (d, *J* = 8.1 Hz, 1H), 7.77 (d, *J* = 8.7 Hz, 1H), 7.73 (d, *J* = 8.5 Hz, 1H), 7.58 (d, *J* = 1.8 Hz, 1H), 7.27 (dd, *J* = 8.5, 1.8 Hz, 1H), 7.19 (d, *J* = 8.3 Hz, 2H), 6.98 (s, 1H), 6.73 (s, 1H), 5.95 (d, *J* = 1.0 Hz, 1H), 5.91 (d, *J* = 1.0 Hz, 1H), 4.69 (t, *J* = 7.9 Hz, 1H), 4.54 (s, 2H), 3.83 (dh, *J* = 13.5, 4.4 Hz, 1H), 3.51 (s, 3H), 3.13 (h, *J* = 8.1 Hz, 2H), 2.25 (s, 3H), 1.80 – 1.72 (m, 2H), 1.68 – 1.33 (m, 10H). LC-MS (ESI): m/z = 518.3, t_R = 5.28 min.

3-(7-(2-(Cycloheptylamino)-2-oxoethoxy)naphthalen-2-yl)-3-(6-methylbenzo[d][1,3]dioxol-5-yl)propanoic acid (24). The reaction was conducted following the general procedure **G**, using **24-I3** (0.10 g, 0.20 mmol) and 2 M aq. NaOH (80 µL) to obtain 0.02 g (22.0%) of **24** as a white solid. ^{1}H NMR (600 MHz, DMSO-d_6) δ 12.38 (s, 1H), 8.21 (d, *J* = 8.1 Hz, 1H), 8.04 – 8.00 (m, 1H), 7.98 (d, *J* = 8.5 Hz, 1H), 7.83 (d, *J* = 1.7 Hz, 1H), 7.53 (dd, *J* = 8.5, 1.8 Hz, 1H), 7.44 (dd, *J* = 6.7, 2.7 Hz, 2H), 7.22 (s, 1H), 6.98 (s, 1H), 6.20 (d, *J* = 1.0 Hz, 1H), 6.17 (d, *J* = 1.0 Hz, 1H), 4.92 (t, *J* = 7.8 Hz, 1H), 4.80 (s, 2H), 4.09 (dh, *J* = 13.5, 4.4 Hz, 1H), 3.32 – 3.21 (m, 2H), 2.50 (s, 3H), 2.06 – 1.97 (m, 2H), 1.93 – 1.69 (m, 8H), 1.65 (dtd, *J* = 16.9, 9.3, 4.9 Hz, 2H). ^{13}C NMR (151 MHz, DMSO-d_6) δ 173.22, 166.54, 156.36, 146.01, 145.66, 142.31, 135.28, 134.50, 129.36, 129.13, 128.10, 127.77, 125.04, 124.97, 118.75, 110.84, 107.61 (2C), 101.08, 67.52, 50.03, 42.76, 40.77, 34.67 (2C), 28.16 (2C), 24.21 (2C), 19.79. LC-MS (ESI): m/z = 504.2, t_R = 4.85 min.

3-(7-(2-(Cycloheptylamino)-2-oxoethoxy) naphthalen-2-yl)-3-(6-methyl-2,3-dihydrobenzofuran-5-yl)propanoic acid (25)

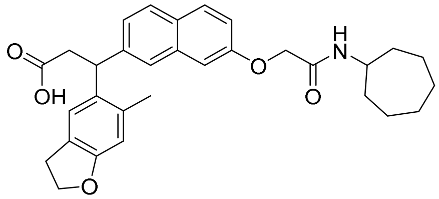

Methyl (*E*)-3-(6-methyl-2,3-dihydrobenzofuran-5-yl)acrylate (25-I2). The reaction was conducted following the general procedure **C**, using 5-bromo-6-methyl-2,3-dihydrobenzofuran (**25-I1**, 0.11 g, 0.52 mmol), methyl acrylate (0.081 mL, 0.78 mmol), $Pd(OAc)_2$ (0.0057 g, 0.025 mmol), tri(*o*-tolyl)phosphine (0.016 g, 0.048 mmol), and Et_3N (0.5 mL, 3.65 mmol) to obtain 0.03 g (30%) of **25-I2** as colorless oil. ^{1}H NMR (600 MHz, DMSO-d_6) δ 7.80 (dd, *J* = 15.9, 1.3 Hz, 1H), 7.52 (d, *J* = 1.4 Hz, 1H), 6.57 (s, 1H), 6.26 (dd, *J* = 15.9, 1.2 Hz, 1H), 4.48 (td, *J* = 8.7, 1.3 Hz, 2H), 4.18 (s, 3H), 3.13 (m, *J* = 1.6 Hz, 2H), 2.48 (s, *J* = 1.9 Hz, 3H). LC-MS (ESI): m/z = 219.2, t_R = 4.40 min.

Methyl 3-(7-(2-(cycloheptylamino)-2-oxoethoxy) naphthalen-2-yl)-3-(6-methyl-2,3-dihydrobenzofuran-5-yl)propanoate (25-I3). The reaction was conducted following the general procedure **F**, using **12-I1** (0.090 g, 0.21 mmol) and **25-I2** (0.030 g, 0.14 mmol), TEA (0.13 mL, 0.7 mmol), and $[Rh(COD)Cl]_2$ (9 mg, 0.019 mmol) to obtain 0.026 g (24%) of **25-I3** as a white solid. LC-MS (ESI): m/z = 516.3, t_R = 5.17 min.

3-(7-(2-(Cycloheptylamino)-2-oxoethoxy) naphthalen-2-yl)-3-(6-methyl-2,3-dihydrobenzofuran-5-yl)propanoic acid (25). The reaction was conducted following the general procedure **G**, using **25-I3** (0.026 g, 0.05 mmol) and 2 M aq. NaOH (240 µL) to obtain 0.003 g (13%) of **25** as a white solid. ^{1}H NMR (600 MHz, DMSO-d_6) δ 12.11 (s, 1H), 7.96 (d, *J* = 8.1 Hz, 1H), 7.79 – 7.74 (m, 1H), 7.72 (d, *J* = 8.5 Hz, 1H), 7.58 – 7.55 (m, 1H), 7.24 (dd, *J* = 8.5, 1.8 Hz, 1H), 7.21 – 7.16 (m, 3H), 6.56 (s, 1H), 4.68 (t, *J* = 7.9 Hz, 1H), 4.54 (s, 2H), 4.45 (ddd, *J* = 15.5, 9.1, 7.7 Hz, 2H), 3.83 (tq, *J* = 12.6, 4.5 Hz, 1H), 3.11 (t, *J* = 8.7 Hz, 2H), 3.06 – 2.93 (m, 2H), 2.26 (s, 3H), 1.80 – 1.72 (m, 2H), 1.68 – 1.32 (m, 9H), 1.25 (s, 1H). ^{13}C NMR (151 MHz, DMSO-d_6) δ 173.28, 166.55, 158.49, 156.35, 142.73, 135.63, 134.50, 134.04, 129.36, 128.06, 127.73, 125.11, 125.09, 124.93, 123.59, 118.68, 111.12, 107.61, 71.27, 67.52, 50.02, 42.45, 40.98, 34.67 (2C), 29.55, 28.17 (2C), 24.21 (2C), 20.24. LC-MS (ESI): m/z = 502.4, t_R = 4.78 min.

3-(7-(2-(Cycloheptylamino)-2-oxoethoxy) naphthalen-2-yl)-3-(6-methylbenzo[d][1,3]dioxol-5-yl)propanoic acid (26)

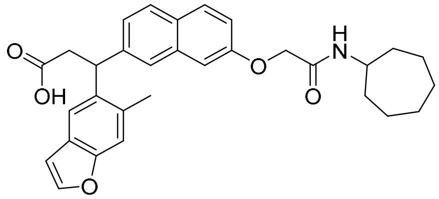

Methyl (*E*)-3-(6-methylbenzofuran-5-yl)acrylate (26-I2). The reaction was conducted following the general procedure **C**, using 5-bromo-6-methylbenzofuran (**26-I1**, 0.25 g, 1.19 mmol), methyl acrylate (0.13 mL, 1.8 mmol), $Pd(OAc)_2$ (0.0014 g, 0.06 mmol), tri(*o*-tolyl)phosphine (0.04 g, 0.13 mmol), and Et_3N (1.2 mL, 8.53 mmol) to obtain 0.15 g (59.4%) of **26-I2** as colorless oil. ^{1}H NMR (600 MHz, DMSO-d_6) δ 8.04 (s, 1H), 7.98 – 7.92 (m, 2H), 7.51 (s, 1H), 6.91 (dd, *J* = 2.2, 1.0 Hz, 1H), 6.53 (d, *J* = 15.8 Hz, 1H), 3.73 (s, 3H). 2.50 (s, 3H). LC-MS (ESI): m/z = 217.1, t_R = 4.66 min.

Methyl 3-(7-(2-(cycloheptylamino)-2-oxoethoxy) naphthalen-2-yl)-3-(6-methylbenzofuran-5-yl)propanoate (26-I3). The reaction was conducted following the general procedure **F**, using **12-I1** (0.12 g,

0.35 mmol) and **26-I2** (0.04 g, 0.24 mmol), TEA (0.1 mL, 0.71 mmol), and $[Rh(COD)Cl]_2$ (12 mg, 0.024 mmol) to obtain 0.073 g (80.1%) of **26-I3** as a white solid. ^{1}H NMR (600 MHz, DMSO-*d₆*) δ 7.95 (d, *J* = 8.2 Hz, 1H), 7.89 (d, *J* = 2.2 Hz, 1H), 7.79 – 7.71 (m, 2H), 7.64 (s, 1H), 7.55 – 7.52 (m, 1H), 7.40 (s, 1H), 7.28 (dd, *J* = 8.5, 1.8 Hz, 1H), 7.18 (dd, *J* = 8.9, 2.5 Hz, 1H), 7.15 (d, *J* = 2.6 Hz, 1H), 6.89 (dd, *J* = 2.2, 1.0 Hz, 1H), 4.84 (d, *J* = 7.9 Hz, 1H), 4.53 (s, 2H), 3.81 (dp, *J* = 13.4, 4.5 Hz, 1H), 3.51 (s, 3H), 3.25 – 3.17 (m, 2H), 2.40 (d, *J* = 5.9 Hz, 3H), 1.74 (dh, *J* = 12.5, 4.1 Hz, 2H), 1.64 – 1.32 (m, 10H).LC-MS (ESI): m/z = 514.3, t_R = 5.31 min.

3-(7-(2-(Cycloheptylamino)-2-oxoethoxy) naphthalen-2-yl)-3-(6-methylbenzo[d][1,3]dioxol-5-yl)propanoic acid (26). The reaction was conducted following the general procedure **G**, using **26-I3** (0.073 g, 0.15 mmol) and 2 M aq. NaOH (150 µL) to obtain 0.008 g (11.2%) of **26** as a white solid. ^{1}H NMR (600 MHz, DMSO-*d₆*) δ 12.14 (s, 1H), 7.95 (d, *J* = 8.1 Hz, 1H), 7.88 (d, *J* = 2.2 Hz, 1H), 7.78 – 7.70 (m, 2H), 7.63 (s, 1H), 7.53 (s, 1H), 7.38 (s, 1H), 7.28 (dd, *J* = 8.5, 1.8 Hz, 1H), 7.20 – 7.12 (m, 2H), 6.88 (d, *J* = 2.7 Hz, 1H), 4.82 (t, *J* = 7.8 Hz, 1H), 4.52 (s, 2H), 3.81 (ddq, *J* = 13.4, 8.9, 4.4 Hz, 1H), 3.12 – 3.05 (m, 2H), 2.39 (s, 3H), 1.73 (dtd, *J* = 12.5, 8.0, 3.8 Hz, 2H), 1.63 – 1.42 (m, 8H), 1.38 (ddt, *J* = 16.2, 10.0, 2.7 Hz, 2H). ^{13}C NMR (151 MHz, DMSO-*d₆*) δ 173.22, 166.54, 156.38, 153.58, 145.84, 142.47, 137.28, 134.49, 133.19, 129.37, 128.13, 127.76, 125.66, 125.19, 119.43, 118.79, 112.91, 107.56, 107.09, 67.49, 49.99, 42.99, 41.32, 34.65 (2C), 28.16 (2C), 24.19 (2C), 20.53. LC-MS (ESI): m/z = 500.2, t_R = 4.87 min.

3-(7-(2-(Cycloheptylamino)-2-oxoethoxy) naphthalen-2-yl)-3-(2,2-difluoro-6-methylbenzo[d][1,3]dioxol-5-yl)propanoic acid (27)

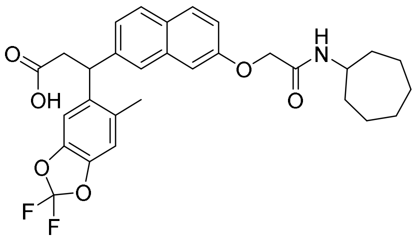

Methyl (*E*)-3-(7-methyl-2,3-dihydrobenzo[b][1,4]dioxin-6-yl)acrylate (27-I2). The reaction was conducted following the general procedure **C**, using 5-bromo-2,2,6-trimethylbenzo[d][1,3]dioxole (**27-I1**, 0.05 g, 0.2 mmol), methyl acrylate (0.027 mL, 0.3 mmol), $Pd(OAc)_2$ (0.0024 g, 0.01 mmol), tri(*o*-tolyl)phosphine (0.006 g, 0.02 mmol), and Et_3N (0.2 mL, 1.39 mmol) to obtain 0.09 g (30%) of **27-I2** as a yellow solid. ^{1}H NMR (600 MHz, DMSO-*d₆*) δ 7.87 (s, 1H), 7.81 (d, *J* = 15.8 Hz, 1H), 7.40 – 7.36 (m, 1H), 6.61 (d, *J* = 15.8 Hz, 1H), 3.74 (s, 3H), 2.42 (d, *J* = 0.6 Hz, 3H). LC-MS (ESI): m/z = 257.1, t_R = 4.82 min.

Methyl 3-(7-(2-(cyclohepten-1-ylamino)-2-oxoethoxy)naphthalen-2-yl)-3-(2,2-difluoro-6-methylbenzo[d][1,3]dioxol-5-yl)propanoate (27-I3). The reaction was conducted following the general procedure **F**, using **12-I1** (0.075 g, 0.18 mmol) and **27-I2** (0.030 g, 0.12 mmol), TEA (0.11 mL, 0.6 mmol), and $[Rh(COD)Cl]_2$ (8 mg, 0.016 mmol) to obtain 0.050 g (50%) of **27-I3** as a white solid. ^{1}H NMR (600 MHz, DMSO-*d₆*) δ 7.97 (d, *J* = 8.2 Hz, 1H), 7.76 (dd, *J* = 15.6, 8.7 Hz, 2H), 7.57 (d, *J* = 1.7 Hz, 1H), 7.52 (s, 1H), 7.30 (dd, *J* = 8.5, 1.8 Hz, 1H), 7.23 (s, 1H), 7.22 – 7.15 (m, 2H), 4.77 (t, *J* = 7.8 Hz, 1H), 4.54 (s, 2H), 3.82 (ddp, *J* = 12.6, 9.0, 4.4 Hz, 1H), 3.52 (s, 3H), 3.27 – 3.16 (m, 2H), 2.34 (s, 3H), 1.75 (qt, *J* = 7.7, 2.5 Hz, 2H), 1.67 – 1.13 (m, 10H). LC-MS (ESI): m/z = 554.2, t_R = 5.40 min.

3-(7-(2-(Cycloheptylamino)-2-oxoethoxy) naphthalen-2-yl)-3-(2,2-difluoro-6-methylbenzo[d][1,3]dioxol-5-yl)propanoic acid (27). The reaction was conducted following the general procedure **G**, using **27-I3** (0.05 g, 0.09 mmol) and 2 M aq. NaOH (500 µL) to obtain 0.002 g (4%) of **27** as a white solid. ^{1}H NMR (600 MHz, DMSO-*d₆*) δ 12.20 (s, 1H), 7.96 (d, *J* = 8.1 Hz, 1H), 7.76 (dd, *J* = 16.3, 8.7 Hz, 2H), 7.57 (d, *J* = 1.8 Hz, 1H), 7.51

(s, 1H), 7.31 (dd, J = 8.5, 1.8 Hz, 1H), 7.23 (s, 1H), 7.22 – 7.14 (m, 2H), 4.74 (t, J = 7.8 Hz, 1H), 4.54 (s, 2H), 3.83 (qt, J = 9.0, 4.4 Hz, 1H), 3.19 – 3.02 (m, 2H), 2.34 (s, 3H), 1.83 – 1.69 (m, 2H), 1.69 – 1.33 (m, 9H), 1.28 – 1.20 (m, 1H). ^{13}C NMR (151 MHz, DMSO-d_6) δ 216.15, 173.08, 166.52, 156.42, 141.79, 141.51, 141.34, 138.80, 134.50, 132.82, 129.39, 128.28, 127.85, 125.28, 124.85, 118.94, 112.00, 109.01, 107.60, 67.51, 50.02, 42.93, 40.68, 34.67 (2C), 28.14 (2C), 24.21 (2C), 19.93. LC-MS (ESI): m/z = 540.2, t_R = 5.12 min.

3-(7-(2-(Cycloheptylamino)-2-oxoethoxy) naphthalen-2-yl)-3-(7-methyl-2,3-dihydrobenzo[b][1,4]dioxin-6-yl)propanoic acid (28)

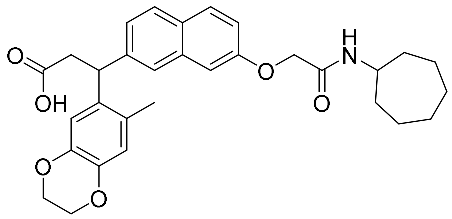

Methyl (*E*)-3-(7-methyl-2,3-dihydrobenzo[b][1,4]dioxin-6-yl)acrylate (28-I2). The reaction was conducted following the general procedure C, using 6-bromo-7-methyl-2,3-dihydrobenzo[b][1,4]dioxine (**28-I1**, 0.04 g, 0.17 mmol), methyl acrylate (0.023 mL, 0.26 mmol), $Pd(OAc)_2$ (0.002 g, 0.009 mmol), tri(*o*-tolyl)phosphine (0.0053 g, 0.016 mmol), and Et_3N (0.17 mL, 1.18 mmol) to obtain 0.005 g (13%) of **28-I2** as a yellow solid. ^{1}H NMR (600 MHz, DMSO-d_6) δ 7.74 (d, J = 15.8 Hz, 1H), 7.27 (s, 1H), 6.76 (d, J = 0.8 Hz, 1H), 6.40 (d, J = 15.8 Hz, 1H), 4.28 – 4.20 (m, 4H), 3.71 (s, 3H), 2.28 (d, J = 0.7 Hz, 3H). LC-MS (ESI): m/z = 235.1, t_R = 4.27 min.

Methyl 3-(7-(2-(cycloheptylamino)-2-oxoethoxy) naphthalen-2-yl)-3-(7-methyl-2,3-dihydrobenzo[b][1,4]dioxin-6-yl)propanoate (28-I3). The reaction was conducted following the general procedure **F**, using **12-I1** (0.075 g, 0.18 mmol) and **28-I2** (0.028 g, 0.12 mmol), TEA (0.1 mL, 0.54 mmol), and $[Rh(COD)Cl]_2$ (7mg, 0.013 mmol) to obtain 0.02 g (22%) of **28-I3** as a white solid. ^{1}H NMR (600 MHz, $CDCl_3$) δ 7.59 (d, J = 8.5 Hz, 1H), 7.44 – 7.41 (m, 1H), 7.13 (dd, J = 8.5, 1.8 Hz, 1H), 7.04 (dd, J = 8.9, 2.6 Hz, 1H), 6.99 (d, J = 2.6 Hz, 1H), 6.70 (s, 1H), 6.56 (s, 1H), 6.44 (d, J = 8.5 Hz, 1H), 4.68 (t, J = 7.8 Hz, 1H), 4.48 (s, 2H), 4.14 (s, 4H), 3.99 (tp, J = 8.2, 4.2 Hz, 1H), 3.77 (s, 1H), 3.52 (s, 3H), 3.01 – 2.90 (m, 2H), 2.12 (s, 3H), 1.86 (qd, J = 7.7, 3.7 Hz, 2H), 1.46 – 1.36 (m, 10H). LC-MS (ESI): m/z = 532.3, t_R = 4.99 min.

3-(7-(2-(Cycloheptylamino)-2-oxoethoxy) naphthalen-2-yl)-3-(7-methyl-2,3-dihydrobenzo[b][1,4]dioxin-6-yl)propanoic acid (28). The reaction was conducted following the general procedure **G**, using **28-I3** (0.02 g, 0.04 mmol) and 2 M aq. NaOH (200 µL) to obtain 0.0073 g (37%) of **28** as a white solid. ^{1}H NMR (600 MHz, DMSO-d_6) δ 12.13 (s, 1H), 7.97 (d, J = 8.1 Hz, 1H), 7.79 – 7.75 (m, 1H), 7.73 (d, J = 8.5 Hz, 1H), 7.57 – 7.54 (m, 1H), 7.25 (dd, J = 8.5, 1.8 Hz, 1H), 7.21 – 7.16 (m, 2H), 6.82 (s, 1H), 6.63 (d, J = 0.8 Hz, 1H), 4.61 (t, J = 7.9 Hz, 1H), 4.54 (s, 2H), 4.22 – 4.13 (m, 4H), 3.83 (dtd, J = 12.6, 9.0, 4.5 Hz, 1H), 3.02 – 2.92 (m, 2H), 2.18 (s, 3H), 1.80 – 1.72 (m, 2H), 1.66 - 1.35 (m, 10H). ^{13}C NMR (151 MHz, DMSO-d_6) δ 173.18, 166.56, 158.38, 156.35, 142.36, 141.77, 141.72, 135.04, 134.49, 129.37, 128.82, 128.10, 127.76, 125.03, 118.94, 118.74, 115.72, 107.57, 67.51, 64.51, 64.50, 50.03, 42.54, 40.85, 34.67 (2C), 28.15 (2C), 24.21 (2C), 19.03. LC-MS (ESI): m/z = 518.2, t_R = 4.61 min.

Synthesis of the cyclohexyl series (29–38)

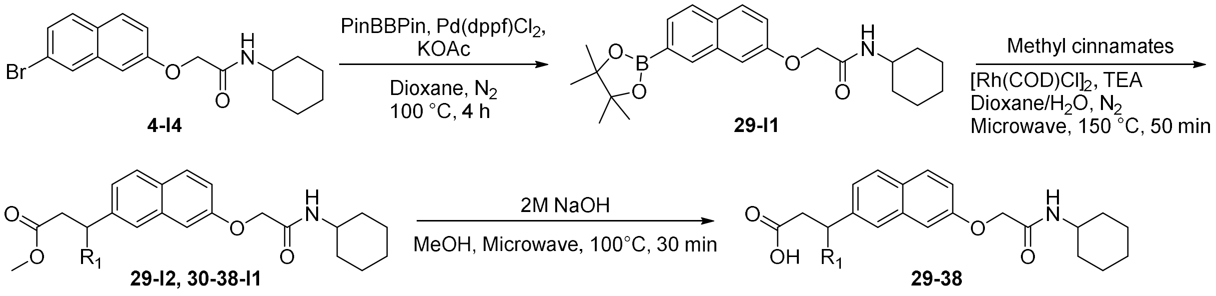

29: R_1 = benzo[*d*][1,3]dioxol-5-yl
30: R_1 = 2,3-dihydrobenzofuran-5-yl
31: R_1 = benzofuran-5-yl
32: R_1 = 2,2-difluorobenzo[*d*][1,3]dioxol-5-yl
33: R_1 = 2,3-dihydrobenzo[*b*][1,4]dioxin-6-yl
34: R_1 = 6-methylbenzo[*d*][1,3]dioxol-5-yl
35: R_1 = 6-methyl-2,3-dihydrobenzofuran-5-yl
36: R_1 = 6-methylbenzofuran-5-yl
37: R_1 = 2,2-difluoro-6-methylbenzo[*d*][1,3]dioxol-5-yl
38: R_1 = 7-methyl-2,3-dihydrobenzo[*b*][1,4]dioxin-6-yl

3-(Benzo[d][1,3]dioxol-5-yl)-3-(7-(2-(cyclohexylamino)-2-oxoethoxy)naphthalen-2-yl)propanoic acid (29)

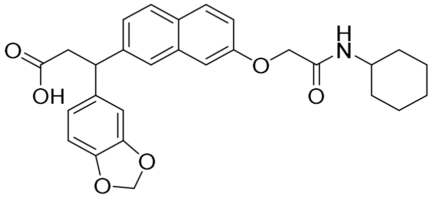

***N*-Cyclohexyl-2-((7-(4,4,5,5-tetramethyl-1,3,2-dioxaborolan-2-yl)naphthalen-2-yl)oxy)acetamide (29-I1).** A solution of **4-I4** (0.30 g, 0.80 mmol), PinB-BPin (0.26 g, 1.00 mmol), KOAc (0.24 g, 2.48 mmol), and $Pd(dppf)Cl_2$ (0.02 g, 0.025 mmol) in dioxane (3 mL) was stirred at 100 °C for 4 h under N_2 atmosphere. Upon reaction completion (as determined by LC-MS) and after cooling down, the salts were filtered out and the resulting filtrate was concentrated. The crude was purified by silica gel column chromatography to obtain 0.97 g (89.1%) of **29-I1** as a afford white solid. ^1H NMR (600 MHz, DMSO-d_6) δ 8.18 (d, *J* = 1.1 Hz, 1H), 7.96 – 7.91 (m, 1H), 7.85 (dd, *J* = 23.6, 8.5 Hz, 2H), 7.58 (dd, *J* = 8.1, 1.1 Hz, 1H), 7.39 (d, *J* = 2.6 Hz, 1H), 7.33 (dd, *J* = 8.9, 2.6 Hz, 1H), 4.56 (s, 2H), 1.72 (ddd, *J* = 28.2, 8.2, 3.8 Hz, 4H), 1.57 (d, *J* = 12.8 Hz, 1H), 1.34 (s, 12H), 1.32 – 1.22 (m, 4H). 1.12 (d, *J* = 10.5 Hz, 1H). LC-MS (ESI): m/z = 410.3, t_R = 5.41 min.

Methyl 3-(benzo[d][1,3]dioxol-5-yl)-3-(7-(2-(cyclohexylamino)-2-oxoethoxy)naphthalen-2-yl)propanoate (29-I2). The reaction was conducted following the general procedure **F**, using **29-I1** (0.15 g, 0.37 mmol) and **19-I2** (0.05 g, 0.25 mmol), TEA (0.18 mL, 0.74 mmol), and $[Rh(COD)Cl]_2$ (12 mg, 0.03 mmol) to obtain 0.76 g (47.8%) of **29-I2** as a white solid. ^1H NMR (600 MHz, DMSO-d_6) δ 7.92 (d, *J* = 8.1 Hz, 1H), 7.79 – 7.71 (m, 2H), 7.69 – 7.66 (m, 1H), 7.32 (dd, *J* = 8.5, 1.8 Hz, 1H), 7.19 (d, *J* = 7.4 Hz, 2H), 6.97 (d, *J* = 1.7 Hz, 1H), 6.85 – 6.78 (m, 2H), 5.94 (dd, *J* = 6.2, 1.0 Hz, 2H), 4.57 – 4.49 (m, 3H), 3.68 – 3.60 (m, 1H), 3.50 (s, 3H), 3.25 – 3.12 (m, 2H), 1.76 – 1.66 (m, 4H), 1.57 (d, *J* = 13.0 Hz, 1H), 1.32 – 1.20 (m, 5H). LC-MS (ESI): m/z = 490.2, t_R = 5.01 min.

3-(Benzo[d][1,3]dioxol-5-yl)-3-(7-(2-(cyclohexylamino)-2-oxoethoxy)naphthalen-2-yl)propanoic acid (29). The reaction was conducted following the general procedure **G**, using **29-I2** (0.04 g, 0.09 mmol) and 2 M aq. NaOH (30 μL) to obtain 0.03 g (89.6%) of **29** as a white solid. ^1H NMR (600 MHz, DMSO-d_6) δ 12.08 (s, 1H), 7.92 (d, *J* = 8.2 Hz, 1H), 7.79 – 7.74 (m, 1H), 7.72 (d, *J* = 8.5 Hz, 1H), 7.67 (d, *J* = 1.7 Hz, 1H), 7.31 (dd, *J* = 8.5, 1.8 Hz, 1H), 7.19 (d, *J* = 7.1 Hz, 2H), 6.95 (d, *J* = 1.6 Hz, 1H), 6.87 – 6.78 (m, 2H), 5.94 (d, *J* = 5.1 Hz, 2H), 4.55 (s, 2H), 4.48 (t, *J* = 7.9 Hz, 1H), 3.65 (s, 1H), 3.13 – 3.00 (m, 2H), 1.78 – 1.53 (m, 6H), 1.33 – 1.07 (m, 6H). ^{13}C NMR (151 MHz, DMSO-d_6) δ 173.04, 166.82, 156.42, 144.60, 141.59, 134.54, 134.01, 129.41, 128.30,

127.86, 127.65, 127.62, 126.23, 125.33, 124.93, 118.95, 107.65, 67.50, 47.88, 43.63, 40.54, 32.71, 25.63 (2C), 25.10 (2C), 16.26. LC-MS (ESI): m/z = 476.2, t_R = 4.59 min.

3-(7-(2-(Cyclohexylamino)-2-oxoethoxy)naphthalen-2-yl)-3-(2,3-dihydrobenzofuran-5-yl)propanoic acid (30)

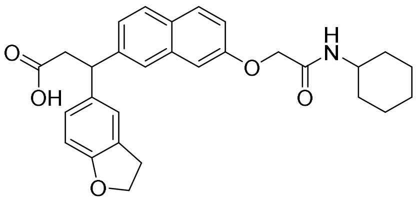

Methyl 3-(7-(2-(cyclohexylamino)-2-oxoethoxy)naphthalen-2-yl)-3-(2,3-dihydrobenzofuran-5-yl)propanoate (30-I1). The reaction was conducted following the general procedure **F**, using **29-I1** (0.15 g, 0.40 mmol) and **20-I2** (0.05 g, 0.25 mmol), TEA (0.11 mL, 0.73 mmol), and $[Rh(COD)Cl]_2$ (13 mg, 0.03 mmol) to obtain 0.07 g (61.5%) of **30-I1** as colorless oil. ^{1}H NMR (600 MHz, DMSO-d_6) δ 7.92 (t, *J* = 4.1 Hz, 1H), 7.79 – 7.74 (m, 1H), 7.72 (d, *J* = 8.5 Hz, 1H), 7.67 – 7.64 (m, 1H), 7.29 (dd, *J* = 8.5, 1.8 Hz, 1H), 7.22 – 7.15 (m, 3H), 7.07 (dd, *J* = 8.3, 2.0 Hz, 1H), 6.65 (d, *J* = 8.2 Hz, 1H), 4.57 – 4.48 (m, 3H), 4.45 (t, *J* = 8.7 Hz, 2H), 3.92 (s, 3H), 3.67 – 3.61 (m, 1H), 3.20 (dd, *J* = 15.8, 8.1 Hz, 1H), 3.17 – 3.07 (m, 3H), 1.75 – 1.66 (m, 4H), 1.57 (d, *J* = 12.5 Hz, 1H), 1.26 (d, *J* = 11.0 Hz, 5H). LC-MS (ESI): m/z = 488.2, t_R = 5.10 min.

3-(7-(2-(Cyclohexylamino)-2-oxoethoxy)naphthalen-2-yl)-3-(2,3-dihydrobenzofuran-5-yl)propanoic acid (30). The reaction was conducted following the general procedure **G**, using **30-I1** (0.07 g, 0.15 mmol) and 2 M aq. NaOH (700 µL) to obtain 0.02 g (32.2%) of **30** as a white solid. ^{1}H NMR (600 MHz, DMSO-d_6) δ 12.08 (s, 1H), 7.92 (d, *J* = 8.2 Hz, 1H), 7.76 (d, *J* = 8.7 Hz, 1H), 7.72 (d, *J* = 8.5 Hz, 1H), 7.66 (d, *J* = 1.7 Hz, 1H), 7.29 (dd, *J* = 8.5, 1.8 Hz, 1H), 7.19 (dq, *J* = 8.5, 2.4 Hz, 3H), 7.07 (dd, *J* = 8.3, 2.0 Hz, 1H), 6.65 (d, *J* = 8.2 Hz, 1H), 4.55 (s, 2H), 4.47 (dt, *J* = 17.4, 8.3 Hz, 3H), 3.7 (m, 1H), 3.13 – 3.05 (m, 3H), 3.02 (dd, *J* = 15.7, 7.9 Hz, 1H), 1.76 – 1.67 (m, 4H), 1.57 (d, *J* = 13.1 Hz, 1H), 1.26 (q, *J* = 9.6 Hz, 5H). ^{13}C NMR (151 MHz, DMSO-d_6) δ 172.73, 166.33, 158.14, 155.85, 142.78, 135.95, 134.09, 128.88, 127.58, 127.32, 127.28, 126.88, 124.42, 124.22, 124.11, 118.15, 108.50, 107.16, 70.78, 67.03, 47.40, 46.19, 40.00, 32.23 (2C), 29.08, 25.14, 24.63 (2C). LC-MS (ESI): m/z = 474.2, t_R = 4.64 min.

3-(Benzofuran-5-yl)-3-(7-(2-(cyclohexylamino)-2-oxoethoxy)naphthalen-2-yl)propanoic acid (31)

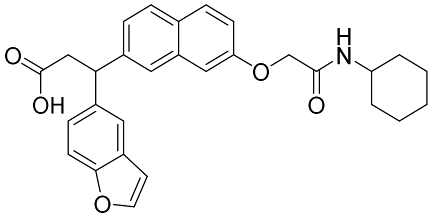

Methyl 3-(benzofuran-5-yl)-3-(7-(2-(cyclohexylamino)-2-oxoethoxy)naphthalen-2-yl)propanoate (31-I1). The reaction was conducted following the general procedure **F**, using **29-I1** (0.50 g, 1.22 mmol) and **21-I2** (0.16 g, 0.81 mmol), TEA (0.35 mL, 2.47 mmol), and $[Rh(COD)Cl]_2$ (41 mg, 0.08 mmol) to obtain 0.3 g (51%) of **31-I1** as colorless oil. ^{1}H NMR (400 MHz, DMSO-d_6) δ 7.95 (d, *J* = 2.2 Hz, 1H), 7.92 (d, *J* = 7.9 Hz, 1H), 7.83 – 7.68 (m, 3H), 7.64 (d, *J* = 1.8 Hz, 1H), 7.49 (d, *J* = 8.5 Hz, 1H), 7.33 (ddd, *J* = 13.2, 8.5, 1.8 Hz, 2H), 7.25 – 7.15 (m, 2H), 6.90 (dd, *J* = 2.2, 1.0 Hz, 1H), 4.71 (t, *J* = 8.0 Hz, 1H), 4.55 (s, 2H), 3.66 (d, *J* = 14.7 Hz, 1H), 3.50 (s, 3H), 1.82 – 1.63 (m, 4H), 1.57 (d, *J* = 12.8 Hz, 1H), 1.26 (d, *J* = 7.2 Hz, 4H), 1.17 (s, 1H), 1.08 (s, 2H). LC-MS (ESI): m/z = 486.2. t_R = 4.99 min.

3-(Benzofuran-5-yl)-3-(7-(2-(cyclohexylamino)-2-oxoethoxy)naphthalen-2-yl)propanoic acid (31). The reaction was conducted following the general procedure **G**, using **31-I1** (0.10 g, 0.20 mmol) and 2M aq. NaOH (900 μL) to obtain 0.038 g (40%) of **31** as a white solid. ^{1}H NMR (400 MHz, DMSO-d_6) δ 12.13 (s, 1H), 7.99 – 7.85 (m, 2H), 7.80 – 7.68 (m, 3H), 7.63 (d, *J* = 1.9 Hz, 1H), 7.48 (d, *J* = 8.5 Hz, 1H), 7.31 (ddd, *J* = 14.7, 8.5, 1.8 Hz, 2H), 7.19 (d, *J* = 8.5 Hz, 2H), 6.93 – 6.84 (m, 1H), 4.68 (t, *J* = 7.9 Hz, 1H), 4.55 (s, 2H), 3.64 (s, 1H), 3.24 – 3.05 (m, 2H), 1.71 (d, *J* = 19.9 Hz, 4H), 1.57 (d, *J* = 12.7 Hz, 1H), 1.25 (d, *J* = 9.8 Hz, 4H), 1.09 (d, *J* = 25.1 Hz, 1H). ^{13}C NMR (101 MHz, DMSO-d_6) δ 173.22, 166.83, 156.36, 153.49, 146.75, 143.14, 139.21, 134.59, 129.38, 128.12, 127.81, 127.74, 124.95, 124.86, 124.78, 120.28, 118.72, 111.52, 107.67, 107.17, 67.52, 47.89, 47.03, 40.68, 32.71 (2C), 25.62 (2C), 25.11. LC-MS (ESI): m/z = 472.2, t_R = 4.57 min.

3-(7-(2-(Cyclohexylamino)-2-oxoethoxy)naphthalen-2-yl)-3-(2,2-difluorobenzo[d][1,3]dioxol-5-yl)propanoic acid (32)

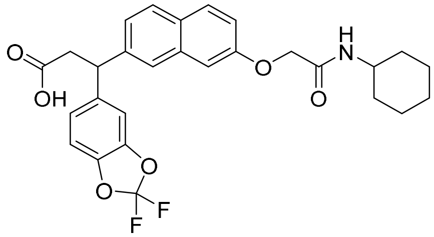

Methyl 3-(7-(2-(cyclohexylamino)-2-oxoethoxy)naphthalen-2-yl)-3-(2,2-difluorobenzo[d][1,3]dioxol-5-yl)propanoate (32-I1). The reaction was conducted following the general procedure **F**, using **29-I1** (0.10 g, 0.25 mmol) and **22-I2** (0.04 g, 0.17 mmol), TEA (0.10 mL, 0.53 mmol), and $[Rh(COD)Cl]_2$ (8 mg, 0.02 mmol) to obtain 0.03 g (34.9%) of **32-I1** as colorless oil. ^{1}H NMR (600 MHz, DMSO-d_6) δ 7.91 (s, 1H), 7.76 (dd, *J* = 18.0, 8.7 Hz, 2H), 7.70 (d, *J* = 1.7 Hz, 1H), 7.52 (d, *J* = 1.7 Hz, 1H), 7.35 (dd, *J* = 8.4, 1.8 Hz, 1H), 7.30 (d, *J* = 8.4 Hz, 1H), 7.26 – 7.16 (m, 3H), 4.65 (t, *J* = 8.0 Hz, 1H), 4.56 (s, 2H), 3.73 – 3.57 (m, 1H), 3.51 (s, 3H), 3.31 – 3.19 (m, 2H), 1.76 – 1.65 (m, 4H), 1.57 (d, *J* = 13.0 Hz, 1H), 1.30 – 1.22 (m, 5H). LC-MS (ESI): m/z = 526.2, t_R = 5.22 min.

3-(7-(2-(Cyclohexylamino)-2-oxoethoxy)naphthalen-2-yl)-3-(2,2-difluorobenzo[d][1,3]dioxol-5-yl)propanoic acid (32). The reaction was conducted following the general procedure **G**, using **32-I1** (0.03 g, 0.06 mmol) and 2 M aq. NaOH (300 μL) to obtain 0.01 g (59.3%) of **32** as a white solid. ^{1}H NMR (600 MHz, DMSO-d_6) δ 12.17 (s, 1H), 7.91 (d, *J* = 8.1 Hz, 1H), 7.80 – 7.71 (m, 2H), 7.69 (d, *J* = 1.7 Hz, 1H), 7.50 (d, *J* = 1.7 Hz, 1H), 7.34 (dd, *J* = 8.5, 1.8 Hz, 1H), 7.31 – 7.17 (m, 4H), 4.61 (t, *J* = 8.0 Hz, 1H), 4.55 (s, 2H), 3.64 (dh, *J* = 9.3, 4.1 Hz, 1H), 3.14 (h, *J* = 8.0 Hz, 2H), 1.75 – 1.65 (m, 4H), 1.55 (dd, *J* = 10.3, 6.5 Hz, 1H), 1.32 – 1.19 (m, 4H), 1.11 (qt, *J* = 11.7, 4.0 Hz, 1H). ^{13}C NMR (151 MHz, DMSO-d_6) δ 173.02, 166.81, 156.41, 143.26, 142.27, 141.66 (2C), 134.58, 129.42, 128.28, 127.92, 124.90, 124.66, 124.12, 118.89, 110.29, 109.90, 107.66, 67.53, 47.90, 46.87, 40.42 (2C), 32.72 (2C), 25.62, 25.12 (2C). LC-MS (ESI): m/z = 512.2, t_R = 4.82 min.

3-(7-(2-(Cyclohexylamino)-2-oxoethoxy)naphthalen-2-yl)-3-(2,3-dihydrobenzo[b][1,4]dioxin-6-yl)propanoic acid (33)

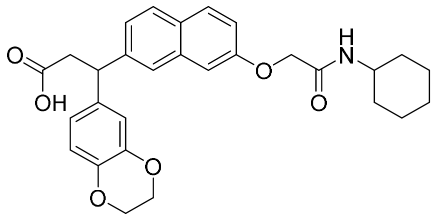

Methyl 3-(7-(2-(cyclohexylamino)-2-oxoethoxy)naphthalen-2-yl)-3-(2,3-dihydrobenzo[b][1,4]dioxin-6-yl)propanoate (33-I1). The reaction was conducted following the general procedure **F**, using **29-I1** (0.10 g,

0.25 mmol) and **23-I2** (0.04 g, 0.17 mmol), TEA (0.10 mL, 0.53 mmol), and $[Rh(COD)Cl]_2$ (8 mg, 0.02 mmol) to obtain 0.04 g (32.8%) of **33-I1** as colorless oil. ^1H NMR (600 MHz, DMSO-d_6) δ 7.92 (d, *J* = 8.1 Hz, 1H), 7.78 – 7.70 (m, 2H), 7.68 – 7.65 (m, 1H), 7.29 (dd, *J* = 8.5, 1.8 Hz, 1H), 7.22 – 7.16 (m, 2H), 6.84 (d, *J* = 2.1 Hz, 1H), 6.80 (dd, *J* = 8.4, 2.2 Hz, 1H), 6.73 (d, *J* = 8.3 Hz, 1H), 4.55 (s, 2H), 4.47 (t, *J* = 8.0 Hz, 1H), 4.20 – 4.15 (m, *J* = 1.7 Hz, 4H), 3.68 – 3.61 (m, 1H), 3.50 (s, 3H), 3.21 – 3.10 (m, 2H), 1.76 – 1.67 (m, 4H), 1.57 (d, *J* = 13.0 Hz, 1H), 1.25 (t, *J* = 7.1 Hz, 4H), 1.11 (d, *J* = 10.8 Hz, 1H). LC-MS (ESI): m/z = 504.3, t_R = 4.88 min.

3-(7-(2-(Cyclohexylamino)-2-oxoethoxy)naphthalen-2-yl)-3-(2,3-dihydrobenzo[b][1,4]dioxin-6-yl)propanoic acid (33). The reaction was conducted following the general procedure **G**, using **33-I1** (0.04 g, 0.08 mmol) and 2 M aq. NaOH (400 µL) to obtain 0.01 g (17.6%) of **33** as a white solid. ^1H NMR (600 MHz, DMSO-d_6) δ 12.09 (s, 1H), 7.92 (d, *J* = 8.1 Hz, 1H), 7.77 (d, *J* = 8.6 Hz, 1H), 7.72 (d, *J* = 8.5 Hz, 1H), 7.66 (d, *J* = 1.7 Hz, 1H), 7.29 (dd, *J* = 8.5, 1.8 Hz, 1H), 7.19 (d, *J* = 8.6 Hz, 2H), 6.83 (d, *J* = 2.1 Hz, 1H), 6.79 (dd, *J* = 8.4, 2.1 Hz, 1H), 6.74 (d, *J* = 8.3 Hz, 1H), 4.55 (s, 2H), 4.44 (t, *J* = 7.9 Hz, 1H), 4.21 – 4.14 (m, 4H), 3.78(m, 1H), 3.04 (qd, *J* = 15.8, 8.0 Hz, 2H), 1.78 – 1.65 (m, 4H), 1.57 (d, *J* = 12.6 Hz, 1H), 1.33 – 1.22 (m, 4H), 1.17 – 0.98 (m, 1H). ^{13}C NMR (151 MHz, DMSO-d_6) δ 173.18, 166.82, 156.34, 143.54, 142.96, 142.26, 137.58, 134.58, 129.38, 128.08, 127.82, 124.86, 124.69, 120.76, 118.69, 117.29, 116.54, 107.65, 67.53, 64.50, 64.40, 47.90, 46.47, 40.43, 32.73 (2C), 25.64, 25.13 (2C). LC-MS (ESI): m/z = 490.2, t_R = 4.45 min.

3-(7-(2-(Cyclohexylamino)-2-oxoethoxy)naphthalen-2-yl)-3-(6-methylbenzo[d][1,3]dioxol-5-yl)propanoic acid (34)

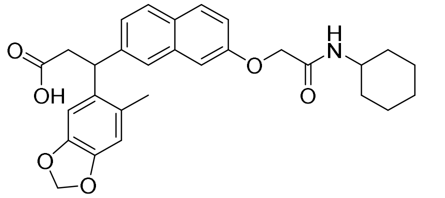

Methyl 3-(7-(2-(cyclohexylamino)-2-oxoethoxy)naphthalen-2-yl)-3-(6-methylbenzo[d][1,3]dioxol-5-yl)propanoate (34-I1). The reaction was conducted following the general procedure **F**, using **29-I1** (0.08 g, 0.21 mmol) and **24-I2** (0.03 g, 0.14 mmol), TEA (0.11 mL, 0.78 mmol), and $[Rh(COD)Cl]_2$ (13 mg, 0.03 mmol) to obtain 0.03 g (33.7%) of **34-I1** as a white solid. ^1H NMR (600 MHz, DMSO-d_6) δ 7.91 (s, 1H), 7.79 – 7.71 (m, 2H), 7.58 (d, *J* = 1.8 Hz, 1H), 7.27 (dd, *J* = 8.5, 1.8 Hz, 1H), 7.22 – 7.17 (m, 2H), 6.99 (s, 1H), 6.73 (s, 1H), 5.95 (d, *J* = 1.0 Hz, 1H), 5.91 (d, *J* = 1.0 Hz, 1H), 4.69 (t, *J* = 7.9 Hz, 1H), 4.55 (s, 2H), 3.64 (d, *J* = 11.2 Hz, 1H), 3.51 (s, 3H), 3.13 (h, *J* = 8.1 Hz, 2H), 2.25 (s, 3H), 1.75 – 1.66 (m, 4H), 1.57 (d, *J* = 12.5 Hz, 1H), 1.29 (dt, *J* = 7.5, 4.1 Hz, 5H). LC-MS (ESI): m/z = 504.3, t_R = 5.16 min.

3-(7-(2-(Cyclohexylamino)-2-oxoethoxy)naphthalen-2-yl)-3-(6-methylbenzo[d][1,3]dioxol-5-yl)propanoic acid (34). The reaction was conducted following the general procedure **G**, using **34-I1** (0.03 g, 0.06 mmol) and 2 M aq. NaOH (700 µL) to obtain 0.002 g (6.9%) of **34** as a white solid. ^1H NMR (600 MHz, DMSO-d_6) δ 12.12 (s, 1H), 7.91 (d, *J* = 8.2 Hz, 1H), 7.79 – 7.74 (m, 1H), 7.72 (d, *J* = 8.5 Hz, 1H), 7.58 (s, 1H), 7.27 (dd, *J* = 8.5, 1.8 Hz, 1H), 7.18 (dd, *J* = 6.5, 2.6 Hz, 2H), 6.96 (s, 1H), 6.73 (s, 1H), 5.93 (d, *J* = 19.3 Hz, 2H), 4.67 (t, *J* = 7.9 Hz, 1H), 4.54 (s, 1H), 3.01 (qd, *J* = 15.9, 7.9 Hz, 2H), 2.24 (s, 3H), 1.75 – 1.66 (m, 4H), 1.59 – 1.54 (m, 1H), 1.32 – 1.20 (m, 7H), 1.14 (tt, *J* = 12.1, 3.6 Hz, 1H). ^{13}C NMR (151 MHz, DMSO-d_6) δ 172.87, 166.47, 155.98, 145.65, 145.30, 141.94, 134.94, 134.14, 129.00, 128.77, 127.74, 127.41, 124.66 (2C), 118.39, 110.48, 107.28 (2C), 100.72, 67.16, 47.51, 42.40, 40.39, 32.36 (2C), 25.28, 24.75 (2C), 19.43. LC-MS (ESI): m/z = 490.2, t_R = 4.60 min.

3-(7-(2-(Cyclohexylamino)-2-oxoethoxy)naphthalen-2-yl)-3-(6-methyl-2,3-dihydrobenzofuran-5-yl)propanoic acid (35)

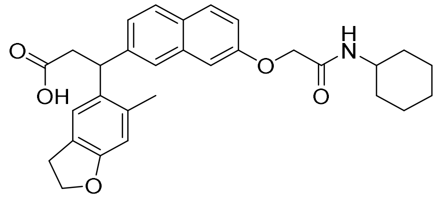

Methyl 3-(7-(2-(cyclohexylamino)-2-oxoethoxy)naphthalen-2-yl)-3-(6-methyl-2,3-dihydrobenzofuran-5-yl)propanoate (35-I1). The reaction was conducted following the general procedure **F**, using **29-I1** (0.12 g, 0.29 mmol) and **25-I2** (0.043 g, 0.2 mmol), TEA (0.1 mL, 0.71 mmol), and $[Rh(COD)Cl]_2$ (12 mg, 0.024 mmol) to obtain 0.021 g (30.9%) of **35-I1** as a white solid. ^{1}H NMR (400 MHz, DMSO-d_6) δ 7.91 (d, *J* = 6.3 Hz, 1H), 7.80 – 7.69 (m, 2H), 7.57 (d, *J* = 1.7 Hz, 1H), 7.26 – 7.16 (m, 4H), 6.56 (s, 1H), 4.70 (t, *J* = 7.8 Hz, 1H), 4.55 (s, 2H), 4.46 (td, *J* = 8.7, 5.1 Hz, 2H), 4.08 (dd, *J* = 10.9, 5.6 Hz, 1H), 3.51 (s, 3H), 3.10 (td, *J* = 14.0, 7.7 Hz, 4H), 2.26 (s, 3H), 1.79 – 1.62 (m, 4H), 1.57 (d, *J* = 12.4 Hz, 1H), 1.30 – 1.19 (m, 4H), 1.11 (d, *J* = 11.9 Hz, 1H). LC-MS (ESI): m/z = 502.2. t_R = 5.09 min.

3-(7-(2-(cyclohexylamino)-2-oxoethoxy)naphthalen-2-yl)-3-(6-methyl-2,3-dihydrobenzofuran-5-yl)propanoic acid (35). The reaction was conducted following the general procedure **G**, using **35-I1** (0.02 g, 0.04 mmol) and 2M aq. NaOH (100 µL) to obtain 0.001 g (5.2%) of **35** as a white solid. ^{1}H NMR (600 MHz, DMSO-d_6) δ 12.16 (s, 1H), δ 7.92 (d, *J* = 8.2 Hz, 1H), 7.76 (d, *J* = 9.6 Hz, 1H), 7.72 (d, *J* = 8.5 Hz, 1H), 7.58 – 7.55 (m, 1H), 7.24 (dd, *J* = 8.5, 1.8 Hz, 1H), 7.18 (h, *J* = 2.6 Hz, 3H), 6.56 (s, 1H), 4.68 (t, *J* = 7.8 Hz, 1H), 4.55 (s, 2H), 4.45 (td, *J* = 8.8, 6.9 Hz, 2H), 3.64 (m, 1H), 3.10 (t, *J* = 8.7 Hz, 2H), 3.06 – 2.93 (m, 2H), 2.26 (s, 3H), 1.84 – 1.63 (m, 4H), 1.57 (d, *J* = 12.2 Hz, 1H), 1.26 (d, *J* = 10.9 Hz, 4H), 1.12 (d, *J* = 12.5 Hz, 1H). ^{13}C NMR (151 MHz, DMSO-d_6) δ 173.21, 166.77, 158.42, 156.26, 142.65, 135.56, 134.43, 133.97, 129.28, 127.99, 127.66, 125.03 (2C), 124.87, 123.53, 118.60, 111.05, 107.58, 71.20, 67.44, 47.80, 42.37, 40.90, 32.64 (2C), 29.48, 25.57, 25.03 (2C), 20.17. LC-MS (ESI): m/z = 488.3, t_R = 4.61 min.

3-(7-(2-(Cyclohexylamino)-2-oxoethoxy)naphthalen-2-yl)-3-(6-methylbenzofuran-5-yl)propanoic acid (36)

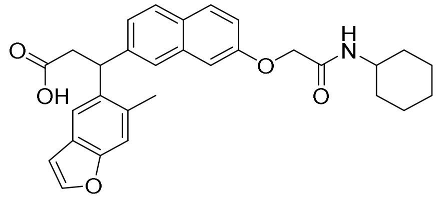

Methyl 3-(7-(2-(cyclohexylamino)-2-oxoethoxy)naphthalen-2-yl)-3-(6-methylbenzofuran-5-yl)propanoate (36-I1). The reaction was conducted following the general procedure **F**, using **29-I1** (0.12 g, 0.29 mmol) and **26-I2** (0.043 g, 0.2 mmol), TEA (0.1 mL, 0.71 mmol), and $[Rh(COD)Cl]_2$ (12 mg, 0.024 mmol) to obtain 0.02 g (50.5%) of **36-I1** as colorless oil. ^{1}H NMR (400 MHz, DMSO-d_6) δ 7.95 – 7.84 (m, 2H), 7.74 (dd, *J* = 14.0, 8.6 Hz, 2H), 7.64 (s, 1H), 7.55 (d, *J* = 1.8 Hz, 1H), 7.38 (s, 1H), 7.27 (dd, *J* = 8.5, 1.8 Hz, 1H), 7.24 – 7.14 (m, 2H), 6.88 (dd, *J* = 2.3, 0.9 Hz, 1H), 4.85 (t, *J* = 7.8 Hz, 1H), 4.54 (s, 2H), 3.63 (s, 1H), 3.50 (s, 3H), 3.21 (d, *J* = 7.9 Hz, 2H), 2.50 (h, *J* = 1.9 Hz, 2H), 2.40 (s, 3H), 1.75 – 1.62 (m, 6H), 1.55 (d, *J* = 12.4 Hz, 2H). LC-MS (ESI): m/z = 500.2. t_R = 5.19 min.

3-(7-(2-(Cyclohexylamino)-2-oxoethoxy)naphthalen-2-yl)-3-(6-methylbenzofuran-5-yl)propanoic acid (36). The reaction was conducted following the general procedure **G**, using **36-I1** (0.05 g, 0.09 mmol) and 2M aq. NaOH (280 µL) to obtain 0.021 g (30.9%) of **36** as a white solid. ^{1}H NMR (600 MHz, DMSO-d_6) δ 12.16 (s, 1H), 7.93 – 7.86 (m, 2H), 7.78 – 7.71 (m, 2H), 7.63 (s, 1H), 7.54 (d, *J* = 1.8 Hz, 1H), 7.39 (s, 1H), 7.28 (dd, *J* =

8.5, 1.8 Hz, 1H), 7.20 – 7.14 (m, 2H), 6.89 (dd, *J* = 2.2, 1.0 Hz, 1H), 4.82 (t, *J* = 7.8 Hz, 1H), 4.53 (s, 2H), 3.13 – 3.05 (m, 2H), 2.62 (p, *J* = 1.9 Hz, 1H), 2.39 (s, 3H), 1.74 – 1.64 (m, 4H), 1.56 (d, *J* = 12.9 Hz, 1H), 1.30 – 1.19 (m, 4H), 1.11 (d, *J* = 11.9 Hz, 1H). ^{13}C NMR (151 MHz, DMSO-d_6) δ 173.23, 166.82, 156.36, 153.58, 145.85, 142.46, 137.30, 134.49, 133.19, 129.37, 128.13, 127.77, 125.66, 125.20, 125.15, 119.44, 118.79, 112.91, 107.61, 107.09, 67.48, 47.84, 42.98, 41.32, 32.70 (2C), 25.64, 25.08, 20.54 (2C). LC-MS (ESI): m/z = 486.3, t_R = 4.73 min.

3-(7-(2-(Cycloheptylamino)-2-oxoethoxy)naphthalen-2-yl)-3-(2,2-difluoro-6-methylbenzo[d][1,3]dioxol-5-yl)propanoic acid (37)

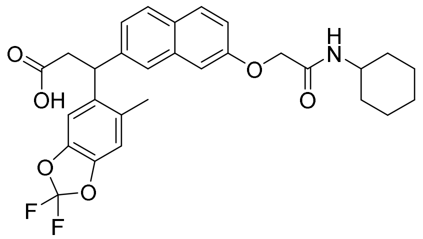

Methyl 3-(7-(2-(cyclohexen-1-ylamino)-2-oxoethoxy)naphthalen-2-yl)-3-(2,2-difluoro-6-methylbenzo[d][1,3]dioxol-5-yl)propanoate (37-I1). The reaction was conducted following the general procedure **F**, using **29-I1** (0.08 g, 0.2 mmol) and **27-I2** (0.03 g, 0.12 mol), TEA (0.11 mL, 0.6 mmol), and $[Rh(COD)Cl]_2$ (8 mg, 0.016 mmol) to obtain 0.02 g (21%) of **37-I1** as a white solid. ^1H NMR (600 MHz, DMSO-d_6) δ 7.93 (d, *J* = 8.2 Hz, 1H), 7.76 (dd, *J* = 16.5, 8.7 Hz, 2H), 7.58 (d, *J* = 1.7 Hz, 1H), 7.53 (s, 1H), 7.30 (dd, *J* = 8.5, 1.8 Hz, 1H), 7.25 – 7.16 (m, 3H), 4.77 (t, *J* = 7.8 Hz, 1H), 4.55 (s, 2H), 3.93 (s, 1H), 3.52 (s, 3H), 3.27 – 3.16 (m, 2H), 2.35 (s, 3H), 1.76 – 1.64 (m, 4H), 1.57 (d, *J* = 13.1 Hz, 1H), 1.30 – 1.21 (m, 4H), 1.18 – 1.11 (m, 1H). LC-MS (ESI): m/z = 540.2. t_R = 5.30 min.

3-(7-(2-(Cycloheptylamino)-2-oxoethoxy)naphthalen-2-yl)-3-(2,2-difluoro-6-methylbenzo[d][1,3]dioxol-5-yl)propanoic acid (37). The reaction was conducted following the general procedure **G**, using **29-I1** (0.05 g, 0.09 mmol) and 2M aq. NaOH (200 µL) to obtain 0.008 g (38%) of **37** as a white solid. ^1H NMR (600 MHz, DMSO-d_6) δ 12.20 (s, 1H), 7.92 (d, *J* = 8.2 Hz, 1H), 7.77 (dd, *J* = 17.2, 8.6 Hz, 2H), 7.58 (d, *J* = 1.8 Hz, 1H), 7.51 (s, 1H), 7.31 (dd, *J* = 8.5, 1.8 Hz, 1H), 7.24 (s, 1H), 7.22 – 7.17 (m, 2H), 4.75 (t, *J* = 7.8 Hz, 1H), 4.55 (s, 2H), 3.15 – 3.04 (m, 2H), 2.34 (s, 3H), 1.76 – 1.66 (m, 3H), 1.60 – 1.54 (m, 1H), 1.32 – 1.20 (m, 6H), 1.13 (td, *J* = 12.2, 3.7 Hz, 1H). ^{13}C NMR (151 MHz, DMSO-d_6) δ 173.08, 166.80, 156.40, 141.79, 141.50, 141.34, 138.81, 134.49, 132.82, 131.72, 129.39, 128.28, 127.86, 125.30, 124.85, 118.94, 111.99, 109.02, 107.64, 67.50, 47.86, 42.93, 40.56, 32.71 (2C), 25.62 (2C), 25.10, 19.94. LC-MS (ESI): m/z = 526.2, t_R = 4.97 min.

3-(7-(2-(Cyclohexylamino)-2-oxoethoxy)naphthalen-2-yl)-3-(7-methyl-2,3-dihydrobenzo[b][1,4]dioxin-6-yl)propanoic acid (38).

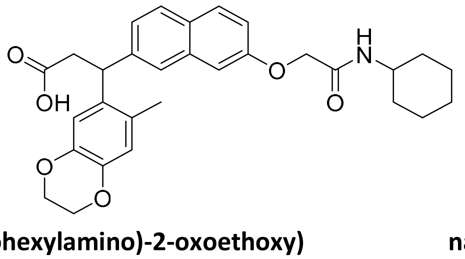

Methyl 3-(7-(2-(cyclohexylamino)-2-oxoethoxy) naphthalen-2-yl)-3-(7-methyl-2,3-dihydrobenzo[b][1,4]dioxin-6-yl)propanoate (38-I1). The reaction was conducted following the general procedure **F**, using **29-I1** (0.08 g, 0.2 mmol) and **28-I2** (0.03 g, 0.13 mmol), TEA (0.11 mL, 0.6 mmol), and $[Rh(COD)Cl]_2$ (8 mg, 0.016 mmol) to obtain 0.032 g (31%) of **38-I1** as white solid. ^1H NMR (600 MHz, DMSO-d_6) δ 7.93 (d, *J* = 8.2 Hz, 1H), 7.77 (d, *J* = 9.6 Hz, 1H), 7.73 (d, *J* = 8.5 Hz, 1H), 7.58 – 7.55 (m, 1H), 7.24 (dd, *J* =

8.5, 1.8 Hz, 1H), 7.21 – 7.17 (m, 2H), 6.83 (s, 1H), 6.63 (d, *J* = 0.8 Hz, 1H), 4.64 (t, *J* = 7.9 Hz, 1H), 4.55 (s, 2H), 4.22 – 4.13 (m, 4H), 3.51 (s, 3H), 3.15 – 3.04 (m, 2H), 2.20 – 2.17 (m, 3H), 1.75 – 1.66 (m, 4H), 1.57 (d, *J* = 12.9 Hz, 1H), 1.31 – 1.24 (m, 4H), 1.18 (t, *J* = 7.1 Hz, 1H), 1.08 (s, 1H). LC-MS (ESI): m/z = 518.3. t_R = 4.60 min.

3-(7-(2-(Cyclohexylamino)-2-oxoethoxy)naphthalen-2-yl)-3-(7-methyl-2,3-dihydrobenzo[b][1,4]dioxin-6-yl)propanoic acid (38). The reaction was conducted following the general procedure **G**, using **38-I1** (0.032 g, 0.062 mmol) and 2M aq. NaOH (300 µL) to obtain 0.02 g (65%) of **38** as a white solid. ^{1}H NMR (600 MHz, DMSO-d_6) δ 12.09 (s, 1H), 7.93 (d, *J* = 8.2 Hz, 1H), 7.79 – 7.75 (m, 1H), 7.73 (d, *J* = 8.5 Hz, 1H), 7.56 (d, *J* = 1.7 Hz, 1H), 7.25 (dd, *J* = 8.4, 1.8 Hz, 1H), 7.21 – 7.16 (m, 2H), 6.82 (s, 1H), 6.63 (s, 1H), 4.61 (t, *J* = 7.9 Hz, 1H), 4.55 (s, 2H), 4.22 – 4.13 (m, 4H), 3.65 (tdt, *J* = 10.5, 7.0, 4.1 Hz, 1H), 3.03 – 2.92 (m, 2H), 2.18 (s, 3H), 1.71 (ddd, *J* = 22.1, 8.4, 3.8 Hz, 3H), 1.60 – 1.54 (m, 1H), 1.33 – 1.21 (m, 5H), 1.13 (ddt, *J* = 15.5, 11.8, 5.8 Hz, 1H). ^{13}C NMR (151 MHz, DMSO-d_6) δ 173.19, 166.85, 156.33, 142.35, 141.77, 141.73, 135.04, 134.49, 129.37, 128.83, 128.10, 127.77, 125.06, 125.03, 118.94, 118.74, 115.73, 107.62, 67.50, 64.51, 64.50, 47.88, 42.54, 40.84, 32.71 (2C), 25.63 (2C), 25.10, 19.03. LC-MS (ESI): m/z = 504.2, t_R = 4.58 min.

Synthesis of the cyclohex-2-enyl series (39–48)

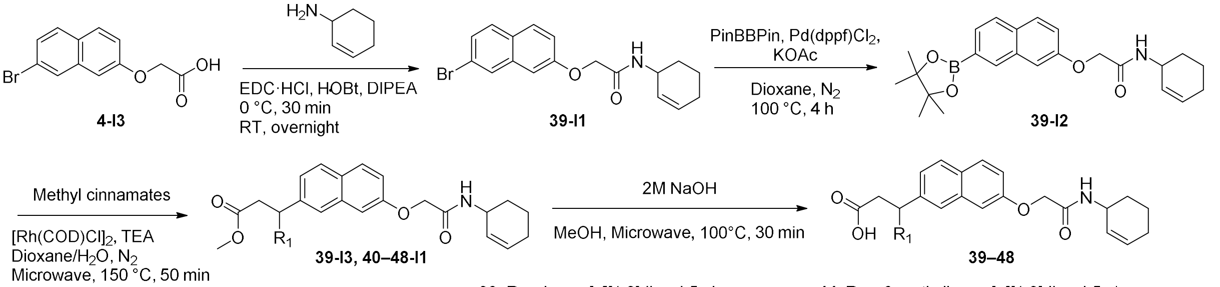

39: R_1 = benzo[*d*][1,3]dioxol-5-yl
40: R_1 = 2,3-dihydrobenzofuran-5-yl
41: R_1 = benzofuran-5-yl
42: R_1 = 2,2-difluorobenzo[*d*][1,3]dioxol-5-yl
43: R_1 = 2,3-dihydrobenzo[*b*][1,4]dioxin-6-yl
44: R_1 = 6-methylbenzo[*d*][1,3]dioxol-5-yl
45: R_1 = 6-methyl-2,3-dihydrobenzofuran-5-yl
46: R_1 = 6-methylbenzofuran-5-yl
47: R_1 = 2,2-difluoro-6-methylbenzo[*d*][1,3]dioxol-5-yl
48: R_1 = 7-methyl-2,3-dihydrobenzo[*b*][1,4]dioxin-6-yl

3-(Benzo[d][1,3]dioxol-5-yl)-3-(7-(2-(cyclohex-2-en-1-ylamino)-2-oxoethoxy)naphthalen-2-yl)propanoic acid (39)

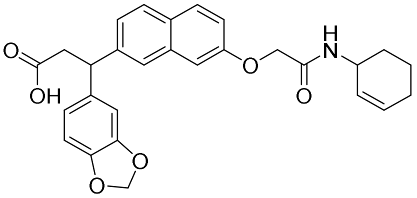

2-((7-Bromonaphthalen-2-yl)oxy)-*N*-(cyclohex-2-en-1-yl)acetamide (39-I1). The reaction was conducted following the general procedure **E**, using **4-I3** (0.5 g, 1.77 mmol), HATU (1.37 g, 3.61 mmol), DIPEA (1.00 mL, 5.74 mmol) and cyclohex-2-en-1-amine (0.27 mL, 2.43 mmol) to obtain 0.7 g (97%) of **39-I1** as a white solid. ^{1}H NMR (600 MHz, $CDCl_3$) δ 7.90 (d, *J* = 2.0 Hz, 1H), 7.75 (d, *J* = 8.9 Hz, 1H), 7.67 – 7.59 (m, 1H), 7.47 – 7.38 (m, 1H), 7.19 (dd, *J* = 8.9, 2.5 Hz, 1H), 7.05 (d, *J* = 2.5 Hz, 1H), 6.53 (d, *J* = 8.7 Hz, 1H), 5.89 (dtd, *J* = 9.7, 3.7, 1.8 Hz, 1H), 5.59 (dq, *J* = 9.9, 2.4 Hz, 1H), 4.59 (s, 3H), 2.06 – 1.90 (m, 2H), 1.75 – 1.46 (m, 2H), 1.32 – 1.20 (m, 2H). ^{13}C NMR (151 MHz, $CDCl_3$) δ 167.02, 155.79, 135.52, 131.54, 129.90 (2C), 129.29, 128.99, 127.78, 127.15, 121.03, 118.52, 106.89, 67.46, 44.48, 29.42, 24.74, 19.74. LC-MS (ESI): m/z = 360.1/362.0, t_R = 5.02 min.

***N*-(cyclohex-2-en-1-yl)-2-((7-(4,4,5,5-tetramethyl-1,3,2-dioxaborolan-2-yl)naphthalen-2-yl)oxy)acetamide (39-I2).** A solution of **39-I1** (0.15 g, 0.4 mmol), PinB-BPin (0.13 g, 0.05 mmol), KOAc (0.125 g, 1.24 mmol), and Pd(dppf)Cl_2 (0.01 g, 0.012 mmol) in dioxane (5 mL) was stirred at 100 °C for 4 h under N_2 atmosphere. Upon reaction completion (as determined by LC-MS) and after cooling down, the salts were filtered out and the resulting filtrate was concentrated. The crude was purified by silica gel column chromatography to obtain 0.15 g (88.7%) of **39-I2** as a white solid. ^{1}H NMR (400 MHz, DMSO-*d$_6$*) δ 8.18 (d, *J* = 1.2 Hz, 1H), 8.12 (d, *J* = 8.2 Hz, 1H), 7.84 (dd, *J* = 14.3, 8.6 Hz, 2H), 7.57 (dd, *J* = 8.1, 1.2 Hz, 1H), 7.38 (d, *J* = 2.5 Hz, 1H), 7.32 (dd, *J* = 8.9, 2.5 Hz, 1H), 5.81 (dtd, *J* = 9.7, 3.7, 2.0 Hz, 1H), 5.54 (dq, *J* = 10.0, 2.4 Hz, 1H), 4.59 (s, 2H), 4.43 – 4.33 (m, 1H), 1.98 (q, *J* = 5.2, 4.2 Hz, 2H), 1.86 – 1.67 (m, 2H), 1.58 – 1.48 (m, 2H), 1.33 (s, 12H).LC-MS (ESI): m/z = 408.2, t_R = 5.33 min.

Methyl 3-(benzo[d][1,3]dioxol-5-yl)-3-(7-(2-(cyclohex-2-en-1-ylamino)-2-oxoethoxy)naphthalen-2-yl)propanoate (39-I3). The reaction was conducted following the general procedure **F**, using **39-I2** (0.15 g, 0.37 mmol) and **19-I2** (0.05 g, 0.25 mmol), TEA (0.11 mL, 0.74 mmol), and [Rh(COD)Cl]$_2$ (13 mg, 0.03 mmol) to obtain 0.023 g (31.2%) of **39-I3** as a white solid. ^{1}H NMR (600 MHz, DMSO-*d$_6$*) δ 8.10 (d, *J* = 8.2 Hz, 1H), 7.79 – 7.74 (m, 1H), 7.73 (d, *J* = 8.5 Hz, 1H), 7.69 – 7.65 (m, 1H), 7.31 (dd, *J* = 8.5, 1.8 Hz, 1H), 7.19 (d, *J* = 7.8 Hz, 2H), 6.97 (d, *J* = 1.7 Hz, 1H), 6.85 – 6.78 (m, 2H), 5.94 (dd, *J* = 5.8, 1.0 Hz, 2H), 5.82 (ddq, *J* = 9.3, 3.6, 1.8

Hz, 1H), 5.55 (dq, J = 10.0, 2.6 Hz, 1H), 4.58 (s, 2H), 4.51 (t, J = 8.0 Hz, 1H), 4.38 (ddt, J = 9.6, 7.0, 3.5 Hz, 1H), 3.50 (s, 3H), 3.22 (dd, J = 15.9, 8.1 Hz, 1H), 3.15 (dd, J = 15.9, 7.9 Hz, 1H), 1.82 – 1.67 (m, 1H), 1.59 – 1.48 (m, 2H), 1.31 – 1.21 (m, 3H). LC-MS (ESI): m/z = 488.2, t_R = 4.94 min.

3-(Benzo[d][1,3]dioxol-5-yl)-3-(7-(2-(cyclohex-2-en-1-ylamino)-2-oxoethoxy)naphthalen-2-yl)propanoic acid (39). The reaction was conducted following the general procedure **G**, using **39-I3** (0.08 g, 0.16 mmol) and 2 M aq. NaOH (300 µL) to obtain 0.023 g (31.2%) of **39** as a white solid. ^{1}H NMR (600 MHz, DMSO-d_6) δ 12.12 (s, 1H), 8.10 (d, J = 8.2 Hz, 1H), 7.78 – 7.74 (m, 1H), 7.72 (d, J = 8.5 Hz, 1H), 7.67 (d, J = 1.8 Hz, 1H), 7.31 (dd, J = 8.5, 1.8 Hz, 1H), 7.21 – 7.16 (m, 2H), 6.95 (d, J = 1.6 Hz, 1H), 6.84 – 6.78 (m, 2H), 5.94 (d, J = 4.7 Hz, 2H), 5.82 (ddp, J = 9.6, 3.7, 1.8 Hz, 1H), 5.55 (dq, J = 10.0, 2.5 Hz, 1H), 4.58 (s, 2H), 4.48 (t, J = 7.9 Hz, 1H), 4.38 (th, J = 8.3, 2.6 Hz, 1H), 3.09 (dd, J = 15.8, 8.1 Hz, 1H), 3.03 (dd, J = 15.9, 7.9 Hz, 1H), 2.05 – 1.92 (m, 2H), 1.83 – 1.66 (m, 2H), 1.54 (tdd, J = 14.0, 7.9, 2.6 Hz, 2H). ^{13}C NMR (151 MHz, DMSO-d_6) δ 172.96, 167.02, 156.18, 147.53, 145.83, 142.71, 138.25, 134.36, 129.91, 129.17, 128.63, 127.89, 127.61, 124.48 (2C), 120.85, 118.51, 108.34 (2C), 107.35, 101.03, 67.18, 46.66, 44.35, 40.28, 29.02, 24.59, 20.06. LC-MS (ESI): m/z = 474.2, t_R = 4.55 min.

3-(7-(2-(Cyclohex-2-en-1-ylamino)-2-oxoethoxy)naphthalen-2-yl)-3-(2,3-dihydrobenzofuran-5-yl)propanoic acid (40)

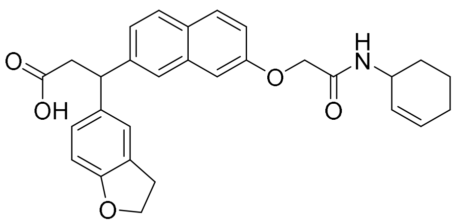

Methyl 3-(7-(2-(cyclohex-2-en-1-ylamino)-2-oxoethoxy)naphthalen-2-yl)-3-(2,3-dihydrobenzofuran-5-yl)propanoate (40-I1). The reaction was conducted following the general procedure **F**, using **39-I2** (0.11 g, 0.3 mmol) and **20-I2** (0.042 g, 0.2 mmol), TEA (0.13 mL, 0.90 mmol), and $[Rh(COD)Cl]_2$ (10 mg, 0.02 mmol) to obtain 0.03 g (22%) of **40-I1** as a white solid. ^{1}H NMR (400 MHz, DMSO-d_6) δ 8.10 (d, J = 8.2 Hz, 1H), 7.74 (dd, J = 15.8, 9.1 Hz, 2H), 7.65 (s, 1H), 7.29 (dd, J = 8.5, 1.8 Hz, 1H), 7.19 (h, J = 2.7 Hz, 3H), 7.07 (d, J = 8.3 Hz, 1H), 6.65 (d, J = 8.2 Hz, 1H), 5.82 (d, J = 10.1 Hz, 1H), 5.58 – 5.51 (m, 1H), 4.58 (s, 2H), 4.48 (dt, J = 24.1, 8.3 Hz, 3H), 4.37 (s, 1H), 3.50 (s, 2H), 3.26 – 3.06 (m, 6H), 1.80 – 1.68 (m, 2H), 1.62 – 1.46 (m, 3H), 1.24 (s, 1H).

3-(7-(2-(Cyclohex-2-en-1-ylamino)-2-oxoethoxy)naphthalen-2-yl)-3-(2,3-dihydrobenzofuran-5-yl)propanoic acid (40). The reaction was conducted following the general procedure **G**, using **40-I1** (0.03 g, 0.06 mmol) and 2 M aq. NaOH (310 µL) to obtain 0.017 g (59%) of **40** as a white solid. ^{1}H NMR (600 MHz, DMSO-d_6) δ 12.10 (s, 1H), 8.11 (d, J = 8.2 Hz, 1H), 7.74 (dd, J = 24.1, 8.5 Hz, 2H), 7.68 – 7.64 (m, 1H), 7.29 (dd, J = 8.5, 1.8 Hz, 1H), 7.21 – 7.18 (m, 3H), 7.07 (dd, J = 8.3, 2.0 Hz, 1H), 6.65 (d, J = 8.2 Hz, 1H), 5.82 (dtd, J = 9.5, 3.5, 1.6 Hz, 1H), 5.55 (dp, J = 10.0, 2.4 Hz, 1H), 4.58 (s, 2H), 4.45 (t, J = 8.7 Hz, 3H), 4.38 (dtq, J = 7.9, 5.3, 2.6 Hz, 1H), 3.13 – 3.05 (m, 3H), 3.02 (dd, J = 15.8, 7.9 Hz, 1H), 2.06 – 1.91 (m, 2H), 1.82 – 1.68 (m, 2H), 1.60 – 1.48 (m, 2H). ^{13}C NMR (151 MHz, DMSO-d_6) δ 173.24, 167.25, 158.64, 156.38, 143.27, 136.44, 134.58, 130.14, 129.38, 128.82, 128.08, 127.83, 127.77, 127.37, 124.92, 124.73, 124.59, 118.66, 109.01, 107.55, 71.28, 67.37, 46.68, 44.55, 40.53, 29.58, 29.23, 24.80, 20.26. LC-MS (ESI): m/z = 472.2, t_R = 4.37 min.

3-(Benzofuran-5-yl)-3-(7-(2-(cyclohex-2-en-1-ylamino)-2-oxoethoxy)naphthalen-2-yl)propanoic acid (41)

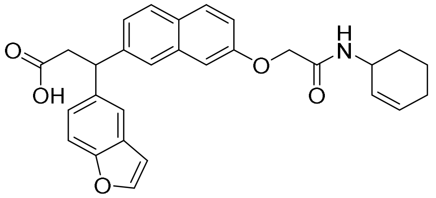

Methyl 3-(benzofuran-5-yl)-3-(7-(2-(cyclohex-2-en-1-ylamino)-2-oxoethoxy)naphthalen-2-yl)propanoate (41-I1). The reaction was conducted following the general procedure **F**, using **39-I2** (0.11 g, 0.30 mmol) and **21-I2** (0.04 g, 0.2 mmol), TEA (0.13 mL, 0.90 mmol), and $[Rh(COD)Cl]_2$ (10 mg, 0.02 mmol) to obtain 0.053 g (37%) of **41-I1** as a white solid. ^{1}H NMR (600 MHz, DMSO-d_6) δ 8.11 (d, J = 8.2 Hz, 1H), 7.94 (d, J = 2.2 Hz, 1H), 7.79 – 7.66 (m, 3H), 7.64 (d, J = 1.8 Hz, 1H), 7.49 (d, J = 8.6 Hz, 1H), 7.32 (ddd, J = 20.5, 8.5, 1.8 Hz, 2H), 7.23 – 7.16 (m, 2H), 6.90 (dd, J = 2.2, 0.9 Hz, 1H), 5.81 (ddt, J = 10.2, 4.5, 2.8 Hz, 1H), 5.54 (ddq, J = 9.6, 4.6, 2.3 Hz, 1H), 4.71 (t, J = 8.0 Hz, 1H), 4.58 (s, 2H), 4.36 (d, J = 8.6 Hz, 1H), 3.50 (s, 3H), 1.98 (d, J = 13.6 Hz, 2H), 1.82 – 1.73 (m, 1H), 1.70 (dq, J = 7.3, 5.4 Hz, 0H), 1.58 – 1.47 (m, 2H), 1.26 (dd, J = 18.4, 6.3 Hz, 2H). LC-MS (ESI): m/z = 484.2, t_R = 4.85 min.

3-(Benzofuran-5-yl)-3-(7-(2-(cyclohex-2-en-1-ylamino)-2-oxoethoxy)naphthalen-2-yl)propanoic acid (41). The reaction was conducted following the general procedure **G**, using **41-I1** (0.053 g, 0.11 mmol) and 2 M aq. NaOH (600 µL) to obtain 0.034 g (66%) of **41** as a white solid. ^{1}H NMR (600 MHz, DMSO-d_6) δ 12.14 (s, 1H), 8.11 (d, J = 8.2 Hz, 1H), 7.94 (d, J = 2.2 Hz, 1H), 7.79 – 7.67 (m, 3H), 7.63 (d, J = 1.8 Hz, 1H), 7.49 (d, J = 8.6 Hz, 1H), 7.32 (ddd, J = 22.5, 8.5, 1.8 Hz, 2H), 7.19 (d, J = 8.7 Hz, 2H), 6.90 (dd, J = 2.2, 1.0 Hz, 1H), 5.85 – 5.78 (m, 1H), 5.57 – 5.51 (m, 1H), 4.68 (t, J = 7.9 Hz, 1H), 4.58 (s, 2H), 4.37 (d, J = 8.6 Hz, 1H), 3.16 (qd, J = 15.9, 8.0 Hz, 2H), 2.04 – 1.92 (m, 2H), 1.82 – 1.74 (m, 1H), 1.74 – 1.65 (m, 1H), 1.53 (tqd, J = 10.2, 6.3, 2.4 Hz, 2H). ^{13}C NMR (151 MHz, DMSO-d_6) δ 173.24, 167.25, 156.41, 153.50, 146.76, 143.15, 139.21, 134.59, 130.15, 129.40, 128.81, 128.13, 127.80, 127.76, 124.88, 120.30, 118.74, 111.54, 107.56, 107.19, 67.37, 47.03, 44.54, 40.53, 29.22, 24.80, 20.23. LC-MS (ESI): m/z = 470.2, t_R = 4.46 min.

3-(7-(2-(Cyclohex-2-en-1-ylamino)-2-oxoethoxy)naphthalen-2-yl)-3-(2,2-difluorobenzo[d][1,3]dioxol-5-yl)propanoic acid (42)

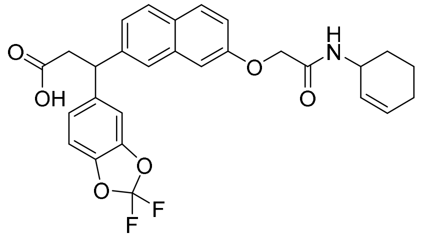

Methyl 3-(7-(2-(cyclohex-2-en-1-ylamino)-2-oxoethoxy)naphthalen-2-yl)-3-(2,2-difluorobenzo[d][1,3]dioxol-5-yl)propanoate (42-I1). The reaction was conducted following the general procedure **F**, using **39-I2** (0.13 g, 0.32 mmol) and **22-I2** (0.05 g, 0.21 mmol), TEA (0.14 mL, 0.99 mmol), and $[Rh(COD)Cl]_2$ (11 mg, 0.02 mmol) to obtain 0.07 g (42%) of **42-I1** as a white solid. ^{1}H NMR (600 MHz, DMSO-d_6) δ 8.14 – 8.08 (m, 1H), 7.76 (dd, J = 15.9, 8.7 Hz, 2H), 7.72 – 7.68 (m, 1H), 7.52 (d, J = 1.8 Hz, 1H), 7.35 (dd, J = 8.5, 1.8 Hz, 1H), 7.30 (d, J = 8.4 Hz, 1H), 7.26 – 7.16 (m, 3H), 5.81 (dtt, J = 9.8, 3.9, 2.2 Hz, 1H), 5.54 (dq, J = 9.2, 2.8 Hz, 1H), 4.65 (t, J = 8.0 Hz, 1H), 4.59 (s, 2H), 4.37 (ddp, J = 8.3, 5.5, 2.8 Hz, 2H), 3.51 (s, 3H), 3.26 (h, J = 8.2 Hz, 3H), 1.83 – 1.75 (m, 1H), 1.71 (ddtd, J = 12.8, 7.5, 5.3, 2.1 Hz, 1H), 1.59 – 1.47 (m, 2H). LC-MS (ESI): m/z = 524.2, t_R = 5.04 min.

3-(7-(2-(Cyclohex-2-en-1-ylamino)-2-oxoethoxy)naphthalen-2-yl)-3-(2,2-difluorobenzo[d][1,3]dioxol-5-yl)propanoic acid (42). The reaction was conducted following the general procedure **G**, using **42-I1** (0.07 g, 0.13 mmol) and 2 M aq. NaOH (599 µL) to obtain 0.023 g (34%) of **42** as a white solid. ^{1}H NMR (600 MHz, DMSO-d_6) δ 12.19 (s, 1H), 8.11 (dd, *J* = 8.2, 1.8 Hz, 1H), 7.81 – 7.67 (m, 3H), 7.51 (d, *J* = 1.8 Hz, 1H), 7.35 (dd, *J* = 8.5, 1.8 Hz, 1H), 7.30 (d, *J* = 8.3 Hz, 1H), 7.26 – 7.16 (m, 3H), 5.81 (ddp, *J* = 9.7, 3.7, 2.2 Hz, 1H), 5.54 (dt, *J* = 9.9, 2.9 Hz, 1H), 4.60 (d, *J* = 18.6 Hz, 3H), 4.38 (ddp, *J* = 8.2, 5.5, 2.7 Hz, 1H), 3.14 (h, *J* = 8.0 Hz, 2H), 2.05 – 1.91 (m, 2H), 1.86 – 1.65 (m, 2H), 1.63 – 1.44 (m, 2H). ^{13}C NMR (151 MHz, DMSO-d_6) δ 173.02, 167.22, 156.45, 143.26, 142.26, 141.70, 141.62, 134.58, 130.11, 129.42, 128.83, 128.29, 127.90, 124.89, 124.67, 124.64, 124.12, 118.89, 110.29, 109.91, 107.56, 67.38, 46.87, 44.56, 40.53, 40.42, 40.28, 40.14, 40.00, 39.86, 39.72, 39.58, 29.22, 24.79, 20.26. LC-MS (ESI): m/z = 510.1, t_R = 4.67 min.

3-(7-(2-(Cyclohex-2-en-1-ylamino)-2-oxoethoxy)naphthalen-2-yl)-3-(2,3-dihydrobenzo[b][1,4]dioxin-6-yl)propanoic acid (43).

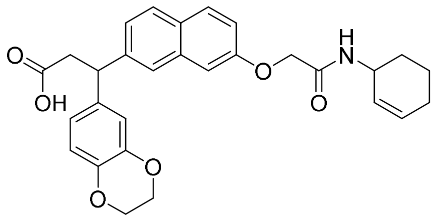

Methyl 3-(7-(2-(cyclohex-2-en-amino)-2-oxoethoxy)naphthalen-2-yl)-3-(2,3-dihydrobenzo[b][1,4]dioxin-6-yl)propanoate (43-I1). The reaction was conducted following the general procedure **F**, using **39-I2** (0.12 g, 0.3 mmol) and **23-I2** (0.05 g, 0.22 mmol), TEA (0.14 mL, 0.99 mmol), and $[Rh(COD)Cl]_2$ (11mg, 0.02 mmol) to obtain 0.048 g (32%) of **43-I1** as a white solid. ^{1}H NMR (600 MHz, DMSO-d_6) δ 8.11 (d, *J* = 8.2 Hz, 1H), 7.79 – 7.74 (m, 1H), 7.72 (d, *J* = 8.5 Hz, 1H), 7.69 – 7.64 (m, 1H), 7.29 (dd, *J* = 8.5, 1.8 Hz, 1H), 7.19 (tt, *J* = 4.9, 2.7 Hz, 2H), 6.84 (d, *J* = 2.1 Hz, 1H), 6.80 (dd, *J* = 8.4, 2.2 Hz, 1H), 6.74 (d, *J* = 8.3 Hz, 1H), 5.85 – 5.79 (m, 1H), 5.55 (dq, *J* = 10.0, 2.6 Hz, 1H), 4.58 (s, 2H), 4.47 (t, *J* = 8.0 Hz, 1H), 4.37 (dtq, *J* = 7.7, 5.0, 2.5 Hz, 1H), 4.20 – 4.14 (m, 4H), 3.50 (s, 3H), 3.16 (qd, *J* = 15.9, 8.0 Hz, 3H), 2.55 (s, 2H), 1.76 – 1.68 (m, 1H), 1.57 – 1.48 (m, 2H). LC-MS (ESI): m/z = 502.2, t_R = 4.68 min.

3-(7-(2-(Cyclohex-2-en-1-ylamino)-2-oxoethoxy)naphthalen-2-yl)-3-(2,3-dihydrobenzo[b][1,4]dioxin-6-yl)propanoic acid (43). The reaction was conducted following the general procedure **G**, using **43-I1** (0.048 g, 0.10 mmol) and 2 M aq. NaOH (450 µL) to obtain 0.023 g (48%) of **43** as a white solid. ^{1}H NMR (600 MHz, DMSO-d_6) δ 12.11 (s, 1H), 8.11 (d, *J* = 8.2 Hz, 1H), 7.76 (d, *J* = 8.6 Hz, 1H), 7.72 (d, *J* = 8.4 Hz, 1H), 7.68 – 7.64 (m, 1H), 7.29 (dd, *J* = 8.5, 1.8 Hz, 1H), 7.19 (d, *J* = 8.2 Hz, 2H), 6.90 – 6.64 (m, 3H), 5.82 (ddt, *J* = 9.0, 3.8, 2.1 Hz, 1H), 5.55 (dp, *J* = 10.1, 2.5 Hz, 1H), 4.58 (s, 2H), 4.50 – 4.31 (m, 2H), 4.24 – 4.11 (m, 3H), 3.04 (qd, *J* = 15.8, 8.0 Hz, 2H), 2.05 – 1.92 (m, 2H), 1.83 – 1.67 (m, 2H), 1.54 (ddtd, *J* = 21.7, 11.9, 10.0, 7.6 Hz, 2H), 1.24 (s, 1H). ^{13}C NMR (151 MHz, DMSO-d_6) δ 173.18, 167.25, 156.38, 143.55, 142.96, 142.27, 137.59, 134.58, 130.13, 129.38, 128.84, 128.09, 127.82, 124.87, 124.68, 120.77, 118.70, 117.30, 116.54, 107.55, 67.38, 64.51, 64.41, 46.47, 44.56, 40.54, 29.23, 24.81, 20.26. LC-MS (ESI): m/z = 488.2, t_R = 4.29 min.

3-(7-(2-(Cyclohex-2-en-1-ylamino)-2-oxoethoxy)naphthalen-2-yl)-3-(6-methylbenzo[d][1,3]dioxol-5-yl)propanoic acid (44)

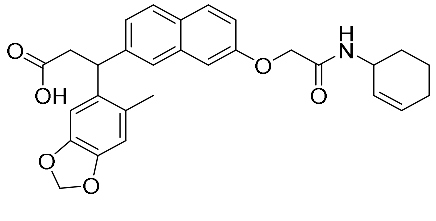

Methyl 3-(7-(2-(cyclohex-2-en-1-ylamino)-2-oxoethoxy)naphthalen-2-yl)-3-(6-methylbenzo[d][1,3]dioxol-5-yl)propanoate (44-I1). The reaction was conducted following the general procedure **F**, using **39-I2** (0.09 g, 0.37 mmol) and **24-I2** (0.032 g, 0.14 mmol), TEA (0.10 mL, 0.74 mmol), and $[Rh(COD)Cl]_2$ (10 mg, 0.02 mmol) to obtain 0.03 g (30%) of **44-I1** as a white solid. ^1H NMR (600 MHz, DMSO-d_6) δ 8.11 (d, *J* = 8.2 Hz, 1H), 7.75 (dd, *J* = 20.7, 9.1 Hz, 2H), 7.58 (s, 1H), 7.27 (dd, *J* = 8.5, 1.8 Hz, 1H), 7.22 – 7.17 (m, 2H), 6.98 (d, *J* = 1.0 Hz, 1H), 6.73 (s, 1H), 5.95 (d, *J* = 1.0 Hz, 1H), 5.91 (d, *J* = 1.0 Hz, 1H), 5.82 (dtd, *J* = 9.5, 3.6, 1.8 Hz, 1H), 5.54 (dt, *J* = 9.9, 2.8 Hz, 1H), 4.69 (t, *J* = 7.9 Hz, 1H), 4.58 (s, 2H), 4.42 – 4.32 (m, 0H), 3.51 (s, 3H), 3.13 (h, *J* = 8.1 Hz, 2H), 2.25 (s, 3H), 1.98 (d, *J* = 9.1 Hz, 2H), 1.81 – 1.66 (m, 1H), 1.60 – 1.44 (m, 1H), 1.24 (s, 1H). LC-MS (ESI): m/z = 502.2, t_R = 4.85 min.

3-(7-(2-(Cyclohex-2-en-1-ylamino)-2-oxoethoxy)naphthalen-2-yl)-3-(6-methylbenzo[d][1,3]dioxol-5-yl)propanoic acid (44). The reaction was conducted following the general procedure **G**, using **44-I1** (0.03 g, 0.06 mmol) and 2 M aq. NaOH (310 µL) to obtain 0.006 g (21%) of **44** as a white solid. ^1H NMR (600 MHz, DMSO-d_6) δ 12.14 (s, 1H), 8.11 (d, *J* = 8.2 Hz, 1H), 7.80 – 7.71 (m, 2H), 7.58 (s, 1H), 7.27 (dd, *J* = 8.5, 1.8 Hz, 1H), 7.22 – 7.16 (m, 2H), 6.96 (d, *J* = 1.7 Hz, 1H), 6.73 (s, 1H), 5.93 (dd, *J* = 17.2, 1.0 Hz, 2H), 5.82 (ddp, *J* = 9.4, 3.7, 1.8 Hz, 1H), 5.54 (dp, *J* = 8.2, 2.6 Hz, 1H), 4.67 (t, *J* = 7.8 Hz, 1H), 4.58 (s, 2H), 4.37 (dtq, *J* = 7.9, 5.3, 2.6 Hz, 1H), 3.07 – 2.95 (m, 2H), 2.25 (s, 3H), 2.02 – 1.93 (m, 2H), 1.81 – 1.67 (m, 2H), 1.53 (ddddd, *J* = 22.1, 19.2, 9.8, 4.7, 2.1 Hz, 2H). ^{13}C NMR (151 MHz, DMSO-d_6) δ 173.23, 167.25, 156.38, 146.01, 145.66, 142.31, 135.29, 134.50, 130.16, 129.37, 129.14, 128.78, 128.11, 127.76, 125.04, 124.98, 118.77, 110.84, 107.62, 107.53, 101.08, 67.36, 44.52, 42.77, 40.77, 29.21, 24.80, 20.22, 19.79. LC-MS (ESI): m/z = 488.2, t_R = 4.45 min.

3-(7-(2-(Cyclohex-2-en-1-ylamino)-2-oxoethoxy)naphthalen-2-yl)-3-(6-methyl-2,3-dihydrobenzofuran-5-yl)propanoic acid (45)

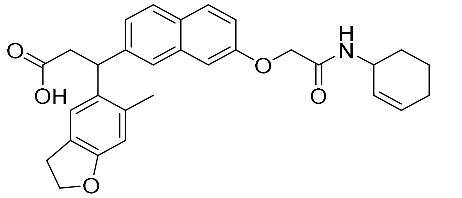

Methyl 3-(7-(2-(cyclohex-2-en-1-ylamino)-2-oxoethoxy)naphthalen-2-yl)-3-(6-methyl-2,3-dihydrobenzofuran-5-yl)propanoate (45-I1). The reaction was conducted following the general procedure **F**, using **39-I2** (0.12 g, 0.35 mmol) and **25-I2** (0.04 g, 0.24 mmol), TEA (0.1 mL, 0.71 mmol), and $[Rh(COD)Cl]_2$ (12 mg, 0.024 mmol) to obtain 0.03 g (35%) of **45-I1** as colorless oil. ^1H NMR (400 MHz, DMSO-d_6) δ 8.09 (d, *J* = 8.1 Hz, 1H), 7.74 (dd, *J* = 15.7, 9.0 Hz, 2H), 7.56 (s, 1H), 7.26 – 7.15 (m, 4H), 6.56 (s, 1H), 5.84 – 5.75 (m, 1H), 5.54 (d, *J* = 10.2 Hz, 1H), 4.71 (t, *J* = 7.8 Hz, 1H), 4.58 (s, 2H), 4.45 (td, *J* = 8.7, 4.7 Hz, 2H), 4.37 (s, 1H), 3.51 (s, 3H), 3.25 – 3.07 (m, 4H), 2.26 (s, 3H), 1.87 – 1.64 (m, 2H), 1.54 (d, *J* = 8.1 Hz, 4H). LC-MS (ESI): m/z = 500.2, t_R = 5.02 min.

3-(7-(2-(Cyclohex-2-en-1-ylamino)-2-oxoethoxy)naphthalen-2-yl)-3-(6-methyl-2,3-dihydrobenzofuran-5-yl)propanoic acid (45). The reaction was conducted following the general procedure **G**, using **45-I1** (0.03 g, 0.06 mmol) and 2 M aq. NaOH (500 µL) to obtain 0.006 g (20.7%) of **45** as a white solid. ^1H NMR (600 MHz, DMSO-d_6) δ 12.09 (s, 1H), 8.09 (d, *J* = 8.3 Hz, 1H), 7.78 – 7.69 (m, 2H), 7.55 (s, 1H), 7.23 (dd, *J* = 8.5, 1.8 Hz, 1H), 7.17 (h, *J* = 2.6 Hz, 3H), 6.55 (s, 1H), 5.84 – 5.78 (m, 1H), 5.53 (dd, *J* = 10.1, 2.7 Hz, 1H), 4.67 (t, *J* = 7.8 Hz, 1H), 4.57 (s, 2H), 4.44 (td, *J* = 8.7, 6.5 Hz, 2H), 4.36 (dtt, *J* = 8.2, 5.5, 2.7 Hz, 1H), 3.09 (t, *J* = 8.7 Hz, 2H), 3.05 – 2.91 (m, 2H), 2.25 (s, 3H), 2.02 – 1.92 (m, 2H), 1.80 – 1.66 (m, 2H), 1.52 (dddd, *J* = 15.3, 12.0, 8.2, 2.8 Hz, 2H). ^{13}C NMR (151 MHz, DMSO-d_6) δ 173.28, 167.24, 158.47, 156.38, 142.72, 135.64, 134.50, 134.05,

130.14, 129.36, 128.80, 128.07, 127.72, 125.09 (2C), 124.92, 123.61, 118.69, 111.12, 107.54, 71.27, 67.36, 44.51, 42.46, 40.97, 29.55, 29.22, 24.81, 20.24 (2C). LC-MS (ESI): m/z = 486.2, t_R = 4.54 min.

3-(7-(2-(Cycloheptylamino)-2-oxoethoxy)naphthalen-2-yl)-3-(6-methylbenzo[d][1,3]dioxol-5-yl)propanoic acid (46)

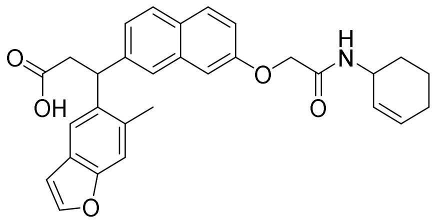

Methyl 3-(7-(2-(cyclohex-2-en-1-ylamino)-2-oxoethoxy)naphthalen-2-yl)-3-(6-methylbenzofuran-5-yl)propanoate (46-I1). The reaction was conducted following the general procedure **F**, using **39-I2** (0.12 g, 0.35 mmol) and **26-I2** (0.04 g, 0.24 mmol), TEA (0.1 mL, 0.71 mmol), and $[Rh(COD)Cl]_2$ (12 mg, 0.024 mmol) to obtain 0.04 g (44%) of **46-I1** as a white solid. ^{1}H NMR (600 MHz, DMSO-d_6) δ 8.08 (d, *J* = 8.3 Hz, 1H), 7.88 (d, *J* = 2.2 Hz, 1H), 7.74 (dd, *J* = 17.9, 8.7 Hz, 2H), 7.63 (s, 1H), 7.57 – 7.51 (m, 1H), 7.39 (s, 1H), 7.27 (dd, *J* = 8.4, 1.7 Hz, 1H), 7.20 – 7.13 (m, 2H), 6.88 (d, *J* = 2.2 Hz, 1H), 5.82 – 5.73 (m, 1H), 5.51 (td, *J* = 9.7, 2.6 Hz, 1H), 4.84 (t, *J* = 7.8 Hz, 1H), 4.56 (s, 2H), 4.39 – 4.27 (m, 1H), 3.50 (s, 3H), 3.24 – 3.17 (m, 2H), 2.39 (s, 3H), 2.01 – 1.90 (m, 2H), 1.81 – 1.40 (m, 4H). LC-MS (ESI): m/z = 498.2, t_R = 5.11 min.

3-(7-(2-(Cycloheptylamino)-2-oxoethoxy)naphthalen-2-yl)-3-(6-methylbenzo[d][1,3]dioxol-5-yl)propanoic acid (46). The reaction was conducted following the general procedure G, using **46-I1** (0.04 g, 0.08 mmol) and 2 M aq. NaOH (500 µL) to obtain 0.017 g (58%) of **46** as a white solid. ^{1}H NMR (600 MHz, DMSO-d_6) δ 12.16 (s, 1H), 8.10 (d, *J* = 8.2 Hz, 1H), 7.89 (d, *J* = 2.2 Hz, 1H), 7.75 (dd, *J* = 17.9, 8.7 Hz, 2H), 7.64 (s, 1H), 7.53 (t, *J* = 2.4 Hz, 1H), 7.40 (s, 1H), 7.29 (dd, *J* = 8.5, 1.7 Hz, 1H), 7.21 – 7.14 (m, 2H), 6.91 – 6.88 (m, 1H), 5.84 – 5.76 (m, 1H), 5.52 (td, *J* = 9.7, 2.7 Hz, 1H), 4.83 (t, *J* = 7.9 Hz, 1H), 4.56 (s, 2H), 4.35 (s, 1H), 3.10 (dd, *J* = 7.9, 1.3 Hz, 2H), 2.39 (d, *J* = 2.6 Hz, 3H), 2.04 – 1.92 (m, 2H), 1.80 – 1.65 (m, 1H), 1.57 – 1.44 (m, 1H), 1.25 (s, 2H). ^{13}C NMR (151 MHz, DMSO-d_6) δ 173.23, 167.22, 156.40, 153.58, 145.84, 142.48, 137.27, 134.49, 133.21, 130.15, 129.37, 128.74, 128.13, 127.76, 125.67, 125.18, 125.16, 119.44, 118.80, 112.91, 107.48, 107.11, 67.33, 44.47, 42.99, 41.33, 29.20, 24.81, 20.54, 20.18. LC-MS (ESI): m/z = 484.2, t_R = 4.65 min.

3-(7-(2-(Cyclohex-2-en-1-ylamino)-2-oxoethoxy)naphthalen-2-yl)-3-(2,2-difluoro-6-methylbenzo[d][1,3]dioxol-5-yl)propanoic acid (47)

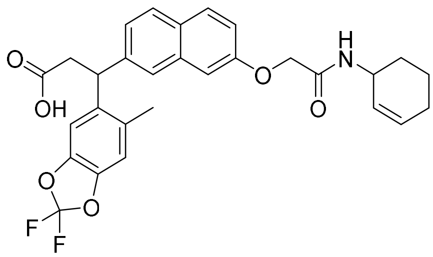

Methyl 3-(7-(2-(cyclohex-2-en-1-ylamino) -2-oxoethoxy)naphthalen-2-yl)-3-(2,2-difluoro-6-methylbenzo[d][1,3]dioxol-5-yl)propanoate (47-I1). The reaction was conducted following the general procedure **F**, using **39-I2** (0.075 g, 0.2 mmol) and **27-I2** (0.03 g, 0.12 mmol), TEA (0.11 mL, 0.6 mmol), and $[Rh(COD)Cl]_2$ (8 mg, 0.016 mmol) to obtain 0.03 g (30%) of **47-I1** as a white solid. ^{1}H NMR (600 MHz, DMSO-d_6) δ 8.11 (d, *J* = 8.2 Hz, 1H), 7.76 (dd, *J* = 14.3, 8.7 Hz, 2H), 7.58 (t, *J* = 2.3 Hz, 1H), 7.53 (s, 1H), 7.30 (dd, *J* = 8.5, 1.8 Hz, 1H), 7.25 – 7.22 (m, 1H), 7.22 – 7.16 (m, 2H), 5.81 (dddd, *J* = 9.6, 5.6, 3.7, 2.0 Hz, 1H), 5.53 (ddq, *J* = 9.8, 5.0, 2.3 Hz, 1H), 4.77 (t, *J* = 7.8 Hz, 1H), 4.58 (s, 2H), 4.36 (s, 1H), 3.52 (s, 3H), 3.27 – 3.16 (m, 2H),

2.35 (s, 3H), 2.04 – 1.95 (m, 2H), 1.81 – 1.73 (m, 1H), 1.73 – 1.66 (m, 1H), 1.58 – 1.46 (m, 1H), 1.32 – 1.21 (m, 1H). LC-MS (ESI): m/z = 538.2, t_R = 5.24 min.

3-(7-(2-(Cyclohex-2-en-1-ylamino)-2-oxoethoxy)naphthalen-2-yl)-3-(2,2-difluoro-6-methylbenzo[d][1,3]dioxol-5-yl)propanoic acid (47). The reaction was conducted following the general procedure **G**, using **47-I1** (0.03 g, 0.06 mmol) and 2 M aq. NaOH (300 µL) to obtain 0.002 g (58%) of **47** as a white solid. ^{1}H NMR (600 MHz, DMSO-d_6) δ 12.20 (s, 1H), 8.10 (d, *J* = 8.2 Hz, 1H), 7.76 (dd, *J* = 14.9, 8.6 Hz, 2H), 7.62 – 7.56 (m, 1H), 7.51 (s, 1H), 7.31 (dd, *J* = 8.5, 1.8 Hz, 1H), 7.25 – 7.23 (m, 1H), 7.23 – 7.16 (m, 2H), 5.81 (dqd, *J* = 9.7, 3.8, 2.0 Hz, 1H), 5.54 (ddd, *J* = 9.4, 6.0, 2.8 Hz, 1H), 4.75 (t, *J* = 7.8 Hz, 1H), 4.58 (s, 2H), 4.40 – 4.34 (m, 1H), 3.15 – 3.04 (m, 2H), 2.34 (s, 3H), 2.04 – 1.93 (m, 2H), 1.83 – 1.64 (m, 2H), 1.60 – 1.45 (m, 1H), 1.30 – 1.18 (m, 1H). ^{13}C NMR (151 MHz, DMSO-d_6) δ 173.08, 167.21, 156.44, 141.79, 141.49, 141.34, 138.81, 134.49, 132.83, 131.72, 130.12, 129.39, 128.80, 128.29, 127.85, 125.29, 124.86, 118.95, 112.00, 109.03, 107.53, 67.35, 44.51, 42.94, 40.68, 29.22, 24.79, 20.23, 19.94. LC-MS (ESI): m/z = 524.2, t_R = 4.90 min.

3-(7-(2-(Cyclohex-2-en-1-ylamino)-2-oxoethoxy)naphthalen-2-yl)-3-(7-methyl-2,3-dihydrobenzo[b][1,4]dioxin-6-yl)propanoic acid (48)

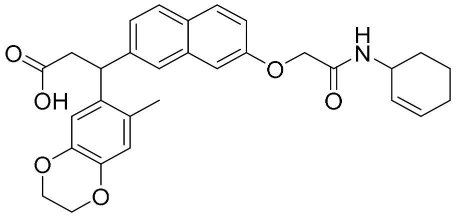

Methyl 3-(7-(2-(cyclohex-2-en-1-ylamino)-2-oxoethoxy)naphthalen-2-yl)-3-(7-methyl-2,3-dihydrobenzo[b][1,4]dioxin-6-yl)propanoate (48-I1). The reaction was conducted following the general procedure **F**, using **39-I2** (0.07 g, 0.18 mmol) and **28-I2** (0.03 g, 0.12 mmol), TEA (0.1 mL, 0.54 mmol), and $[Rh(COD)Cl]_2$ (7 mg, 0.013 mmol) to 0.02 g (22%) of **48-I1** as a white solid. ^{1}H NMR (600 MHz, DMSO-d_6) δ 8.11 (d, *J* = 8.2 Hz, 1H), 7.75 (dd, *J* = 22.0, 9.1 Hz, 2H), 7.57 (s, 1H), 7.24 (dd, *J* = 8.4, 1.8 Hz, 1H), 7.22 – 7.16 (m, 2H), 6.83 (d, *J* = 1.7 Hz, 1H), 6.63 (s, 1H), 5.82 (dt, *J* = 9.9, 2.9 Hz, 1H), 5.54 (dq, *J* = 10.1, 2.5 Hz, 1H), 4.64 (t, *J* = 7.9 Hz, 1H), 4.58 (s, 2H), 4.17 (q, *J* = 1.7 Hz, 4H), 3.51 (s, 3H), 3.15 – 3.04 (m, 2H), 2.19 (s, 3H), 1.98 (s, 2H), 1.83 – 1.64 (m, 1H), 1.58 – 1.48 (m, 2H), 1.31 – 1.23 (m, 2H). LC-MS (ESI): m/z = 516.3, t_R = 4.80 min.

3-(7-(2-(Cyclohex-2-en-1-ylamino)-2-oxoethoxy)naphthalen-2-yl)-3-(7-methyl-2,3-dihydrobenzo[b][1,4]dioxin-6-yl)propanoic acid (48). The reaction was conducted following the general procedure **G**, using **48-I1** (0.02 g, 0.06 mmol) and 2 M aq. NaOH (200 µL) to obtain 0.014 g (70%) of **48** as a white solid. ^{1}H NMR (600 MHz, DMSO-d_6) δ 12.13 (s, 1H), 8.11 (d, *J* = 8.2 Hz, 1H), 7.80 – 7.74 (m, 1H), 7.73 (d, *J* = 8.5 Hz, 1H), 7.56 (d, *J* = 1.6 Hz, 1H), 7.25 (dd, *J* = 8.5, 1.8 Hz, 1H), 7.22 – 7.16 (m, 2H), 6.82 (d, *J* = 2.2 Hz, 1H), 6.63 (d, *J* = 0.8 Hz, 1H), 5.82 (dtd, *J* = 9.7, 3.7, 1.9 Hz, 1H), 5.54 (dp, *J* = 10.1, 2.5 Hz, 1H), 4.61 (t, *J* = 7.8 Hz, 1H), 4.58 (s, 2H), 4.37 (dtq, *J* = 7.5, 4.9, 2.5 Hz, 1H), 4.18 (t, *J* = 1.4 Hz, 4H), 3.03 – 2.92 (m, 2H), 2.18 (s, 3H), 2.05 – 1.95 (m, 2H), 1.83 – 1.67 (m, 2H), 1.53 (ddddt, *J* = 16.9, 12.1, 7.5, 5.1, 2.6 Hz, 2H). ^{13}C NMR (151 MHz, DMSO-d_6) δ 173.21, 167.27, 156.38, 142.36, 141.78, 141.73, 135.06, 134.50, 130.17, 129.39, 128.84, 128.80, 128.12, 127.77, 125.06, 118.95, 118.76, 115.74, 107.52, 67.36, 64.51, 64.50, 44.52, 42.56, 40.85, 40.53, 40.41, 40.39, 40.27, 40.13, 39.99, 39.85, 39.72, 39.58, 29.22, 24.81, 20.23, 19.03. LC-MS (ESI): m/z = 502.2, t_R = 4.41 min.

NMR and LC-MS Spectra (UV250) of Final Compounds (1–48)

^{1}H NMR (400 MHz, DMSO-d_6) of compound **1**

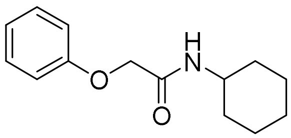

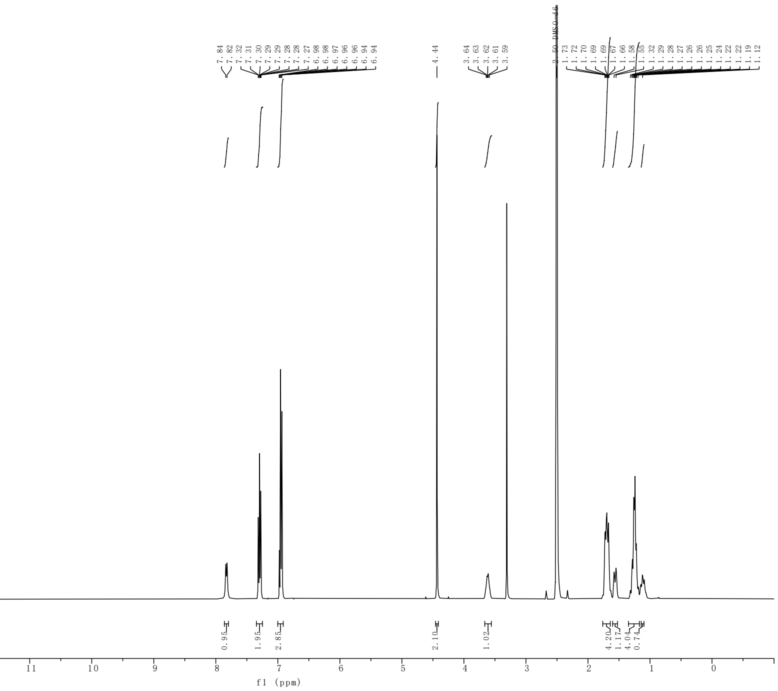

^{13}C NMR (101 MHz, DMSO-d_6) of compound **1**

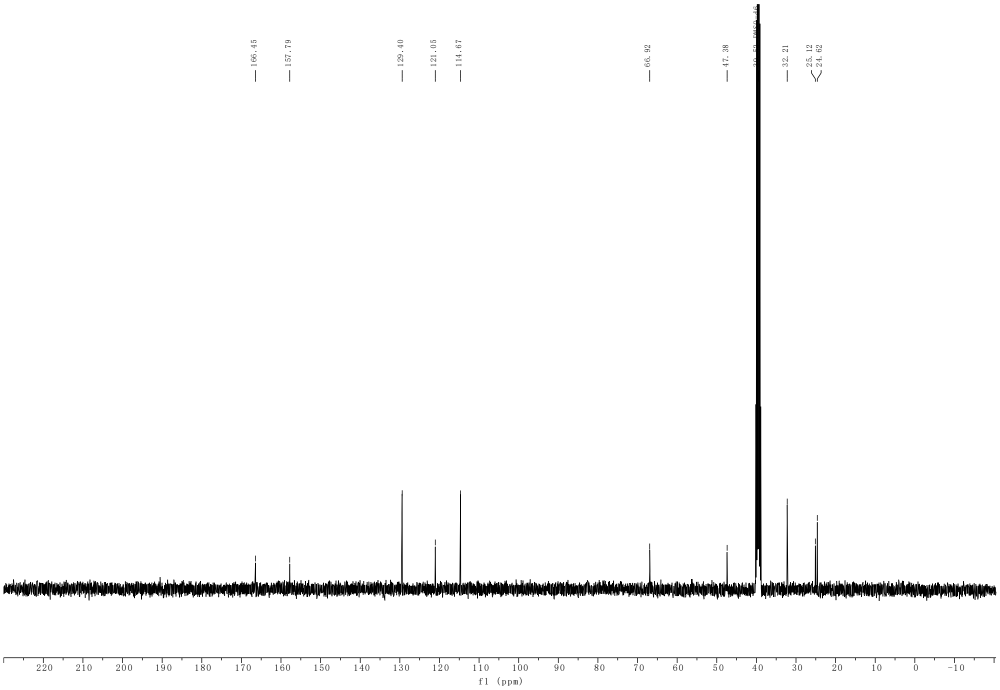

LC-MS Spectra (UV250) of compound **1**

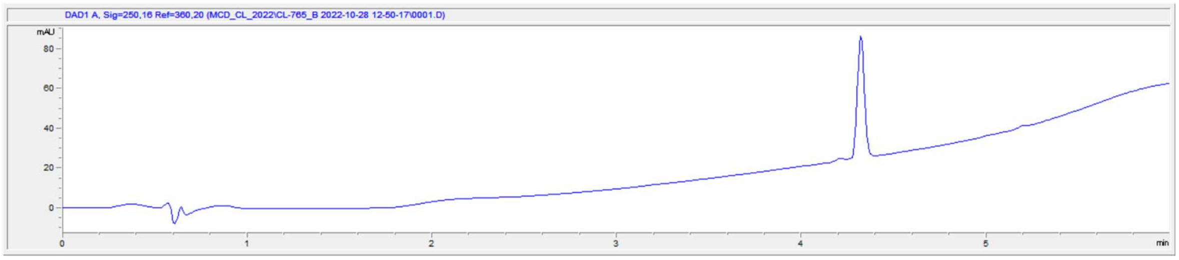

^{1}H NMR (600 MHz, DMSO-d_6) of compound **2**

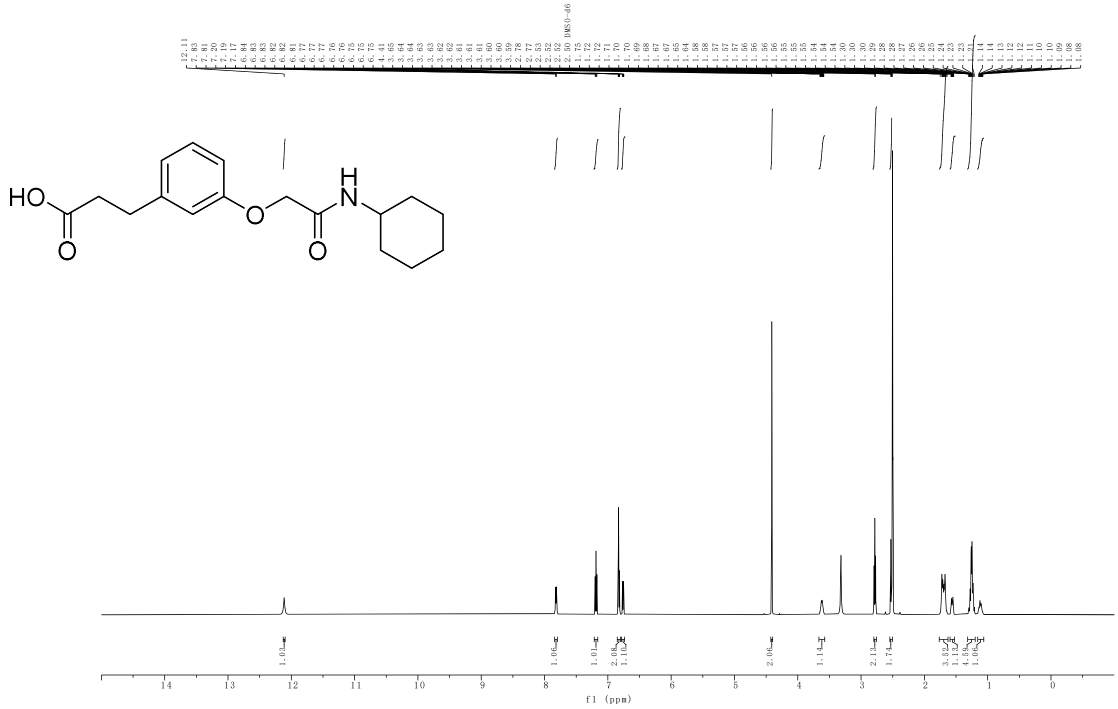

^{13}C NMR (151 MHz, DMSO-d_6) of compound **2**

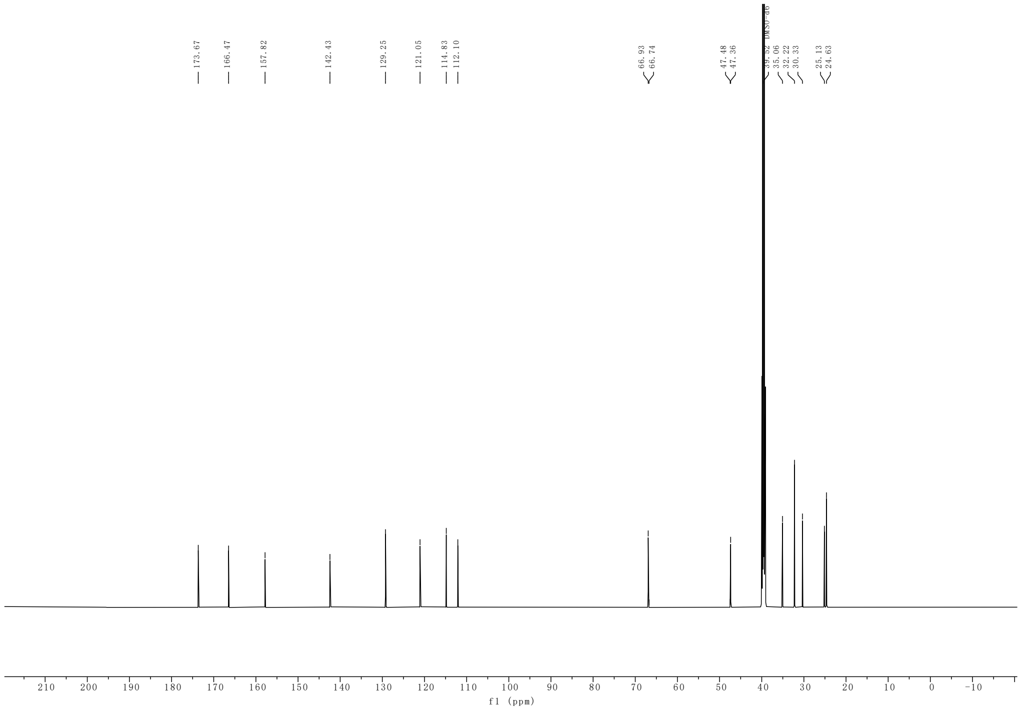

LC-MS Spectra (UV250) of compound **2**

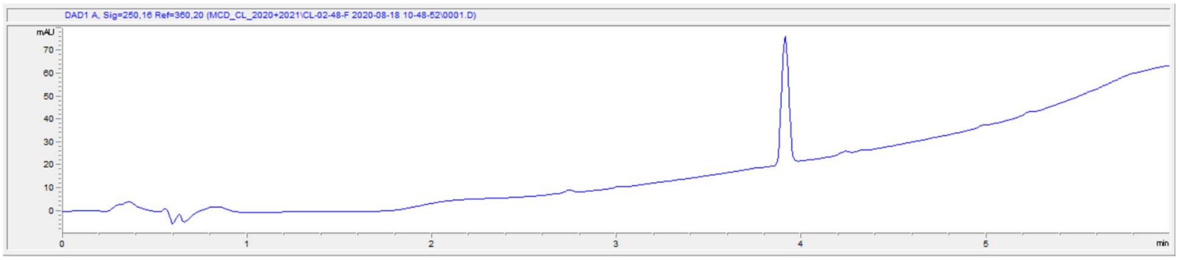

¹H NMR (600 MHz, DMSO-d_6) of compound **3**

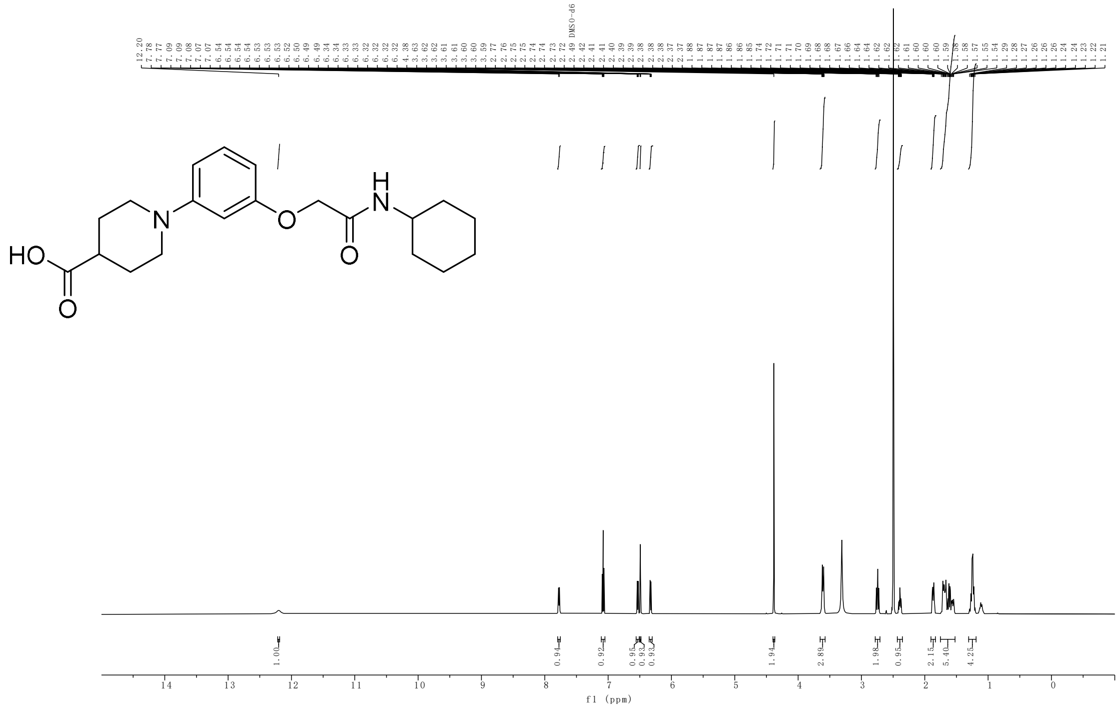

¹³C NMR (151 MHz, DMSO-d_6) of compound **3**

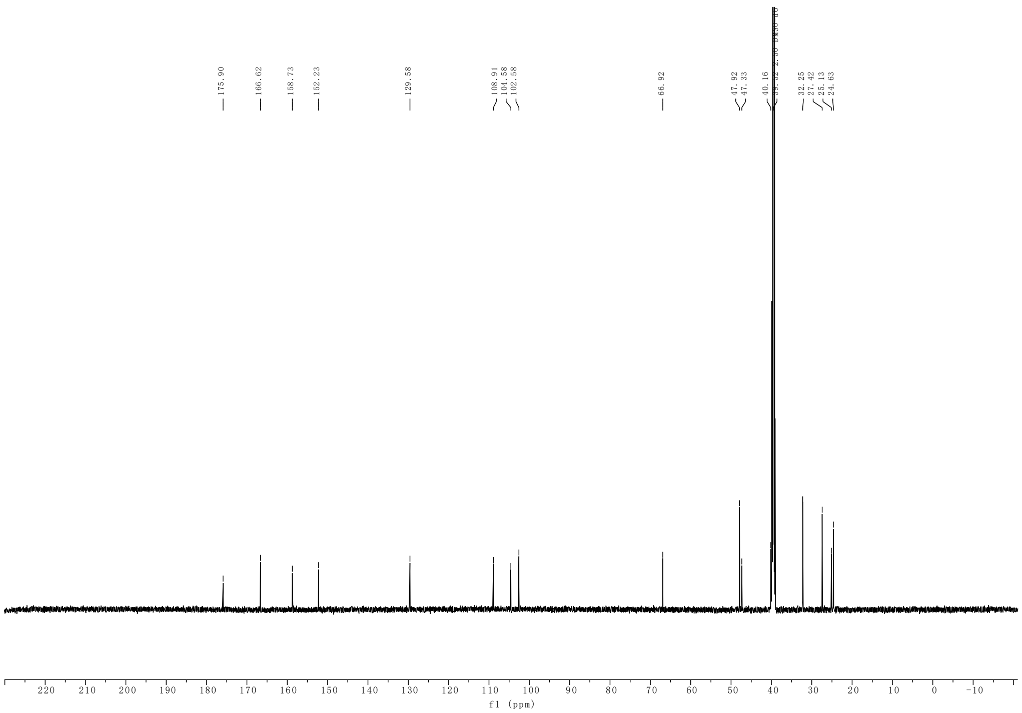

LC-MS Spectra (UV250) of compound **3**

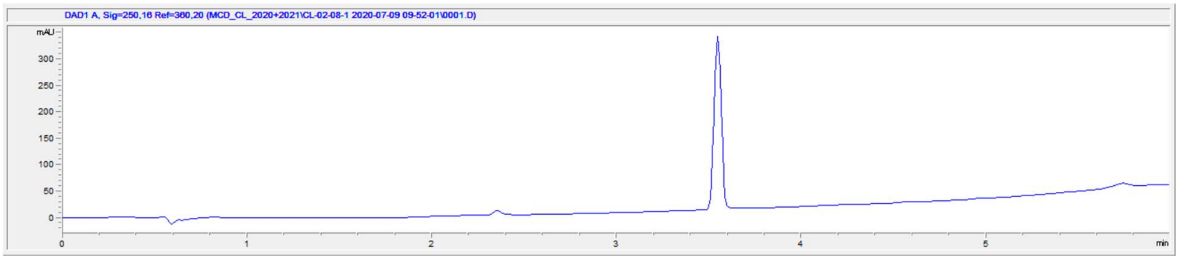

^{1}H NMR (600 MHz, DMSO-d_6) of compound **4**

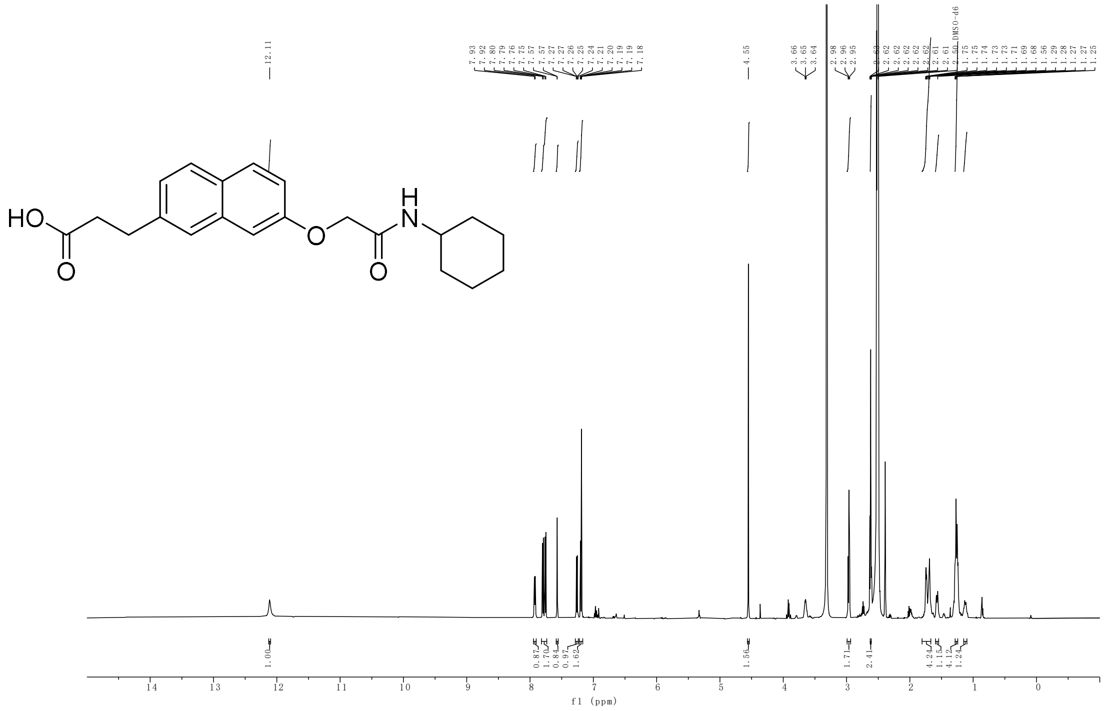

^{13}C NMR (151 MHz, DMSO-d_6) of compound **4**

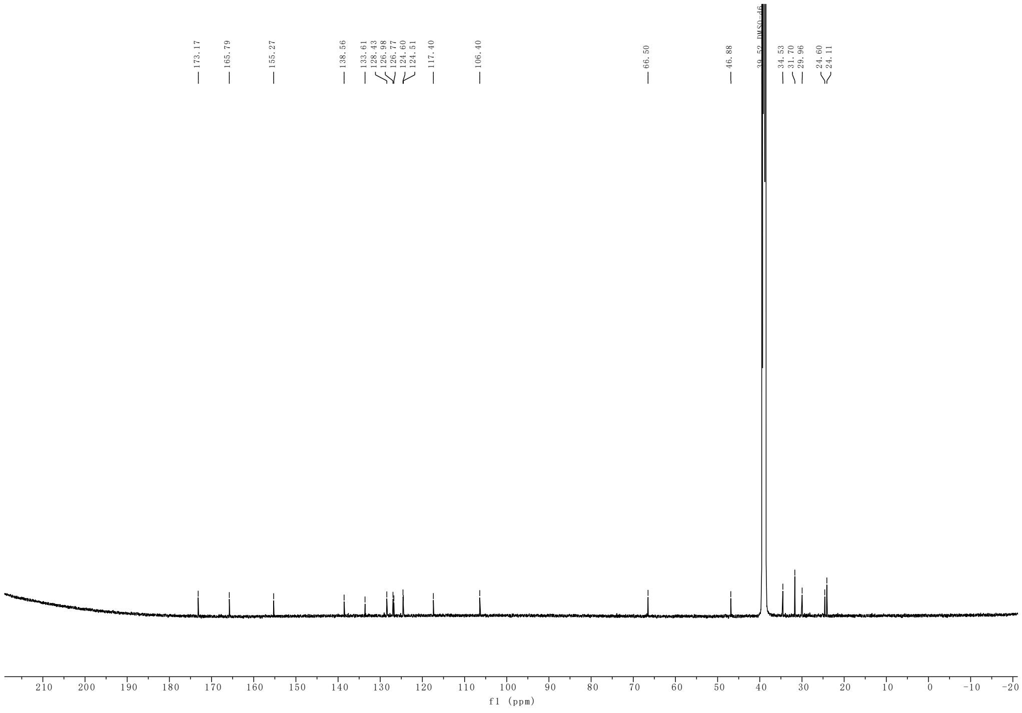

LC-MS Spectra (UV250) of compound **4**

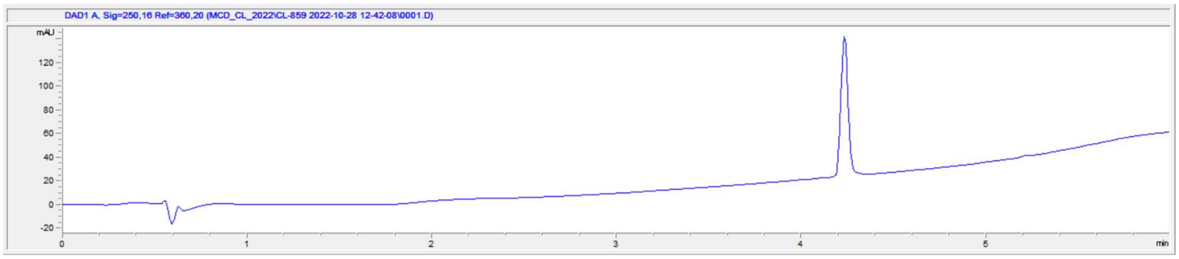

^{1}H NMR (600 MHz, DMSO-d_6) of compound **5**

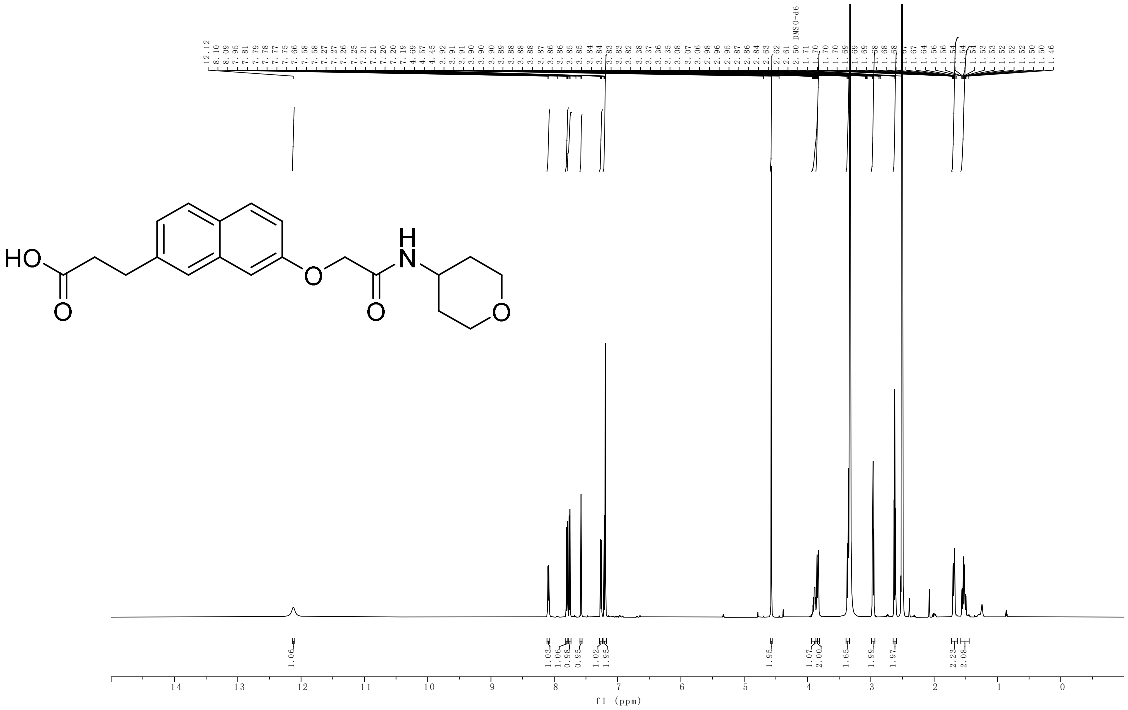

^{13}C NMR (151 MHz, DMSO-d_6) of compound **5**

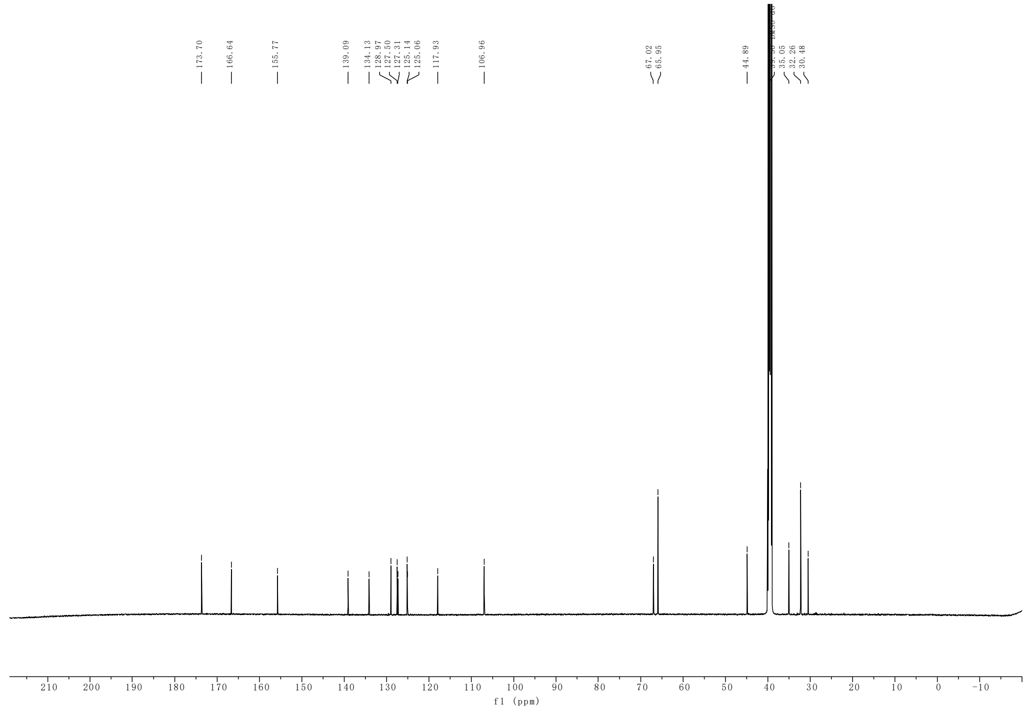

LC-MS Spectra (UV250) of compound **5**

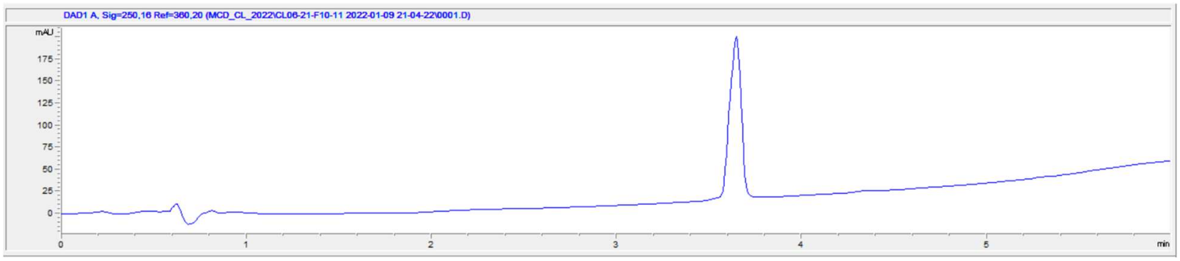

^{1}H NMR (600 MHz, DMSO-d_6) of compound **6**

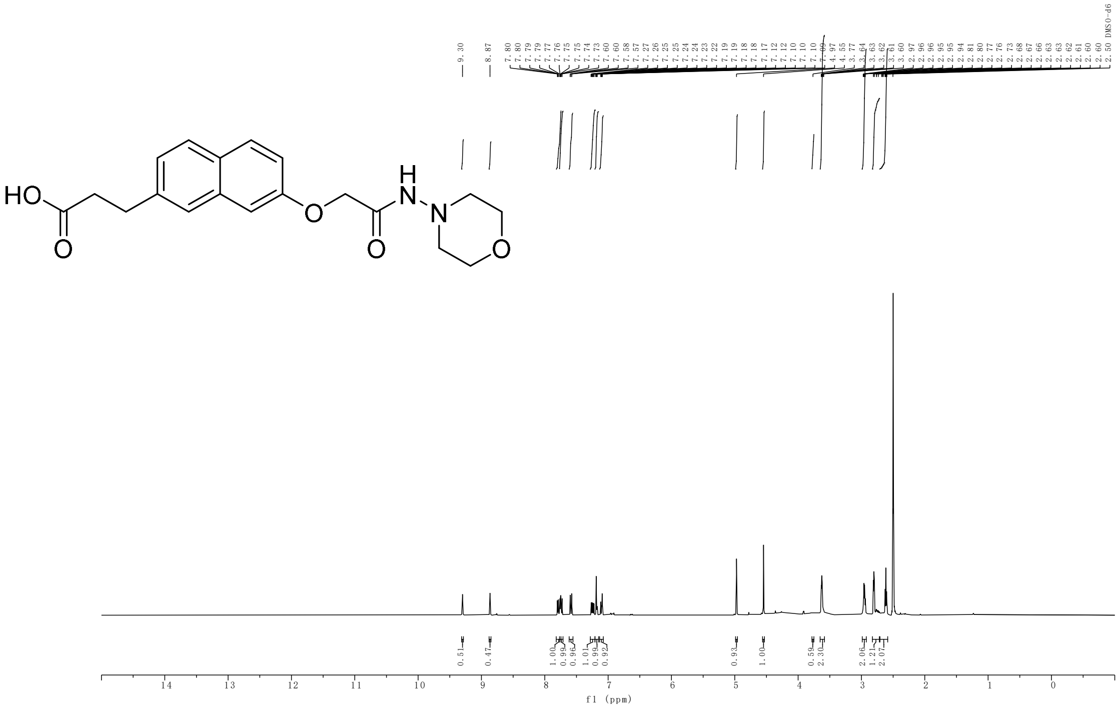

^{13}C NMR (151 MHz, DMSO-d_6) of compound **6**

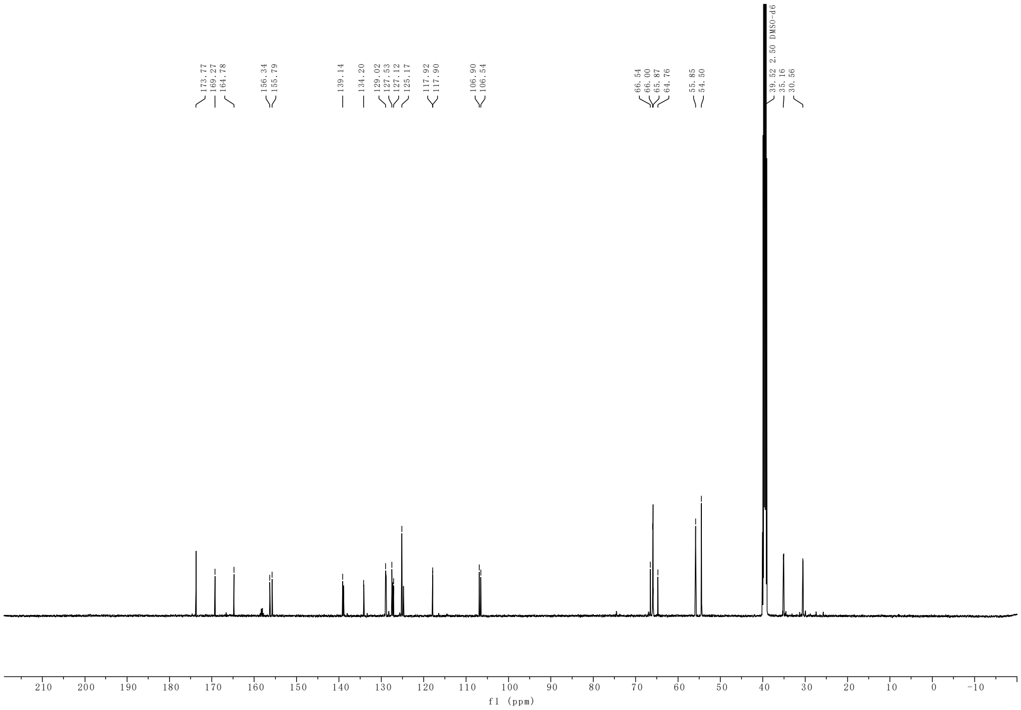

LC-MS Spectra (UV250) of compound **6**

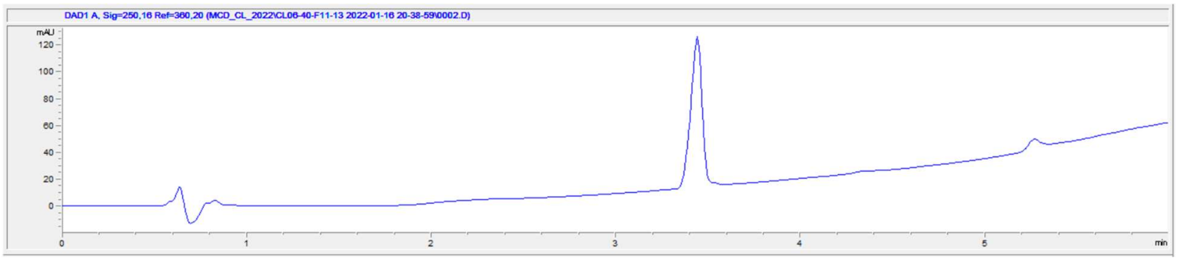

^{1}H NMR (600 MHz, DMSO-d_6) of compound **7**

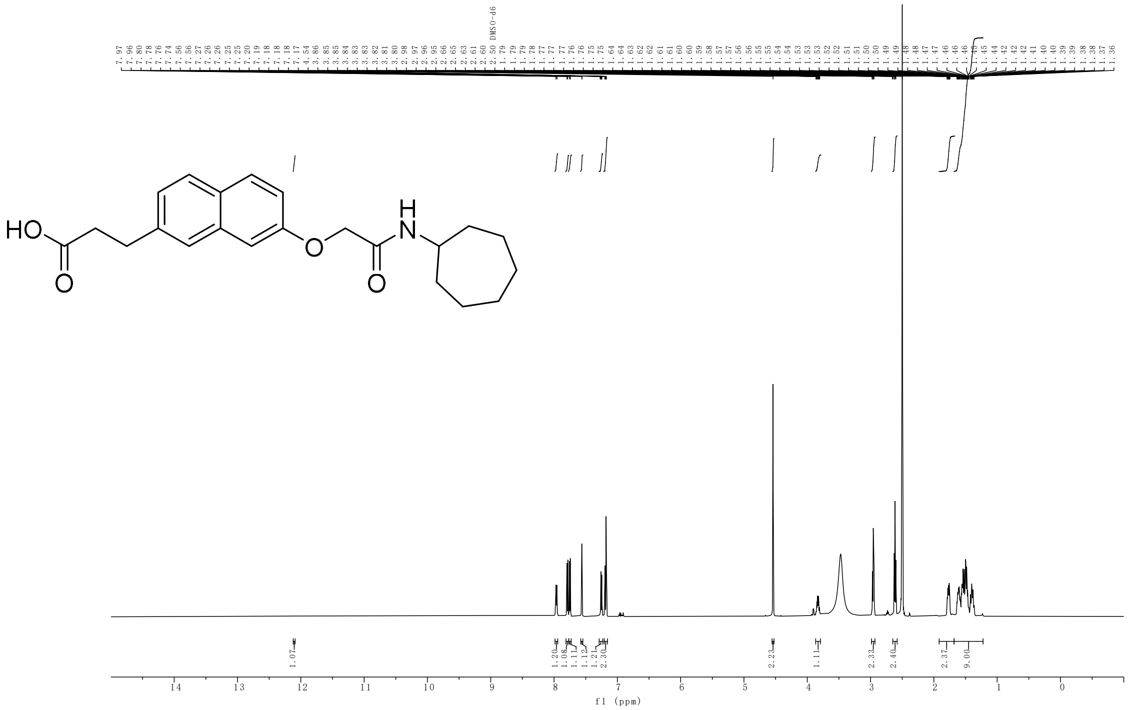

^{13}C NMR (151 MHz, DMSO-d_6) of compound **7**

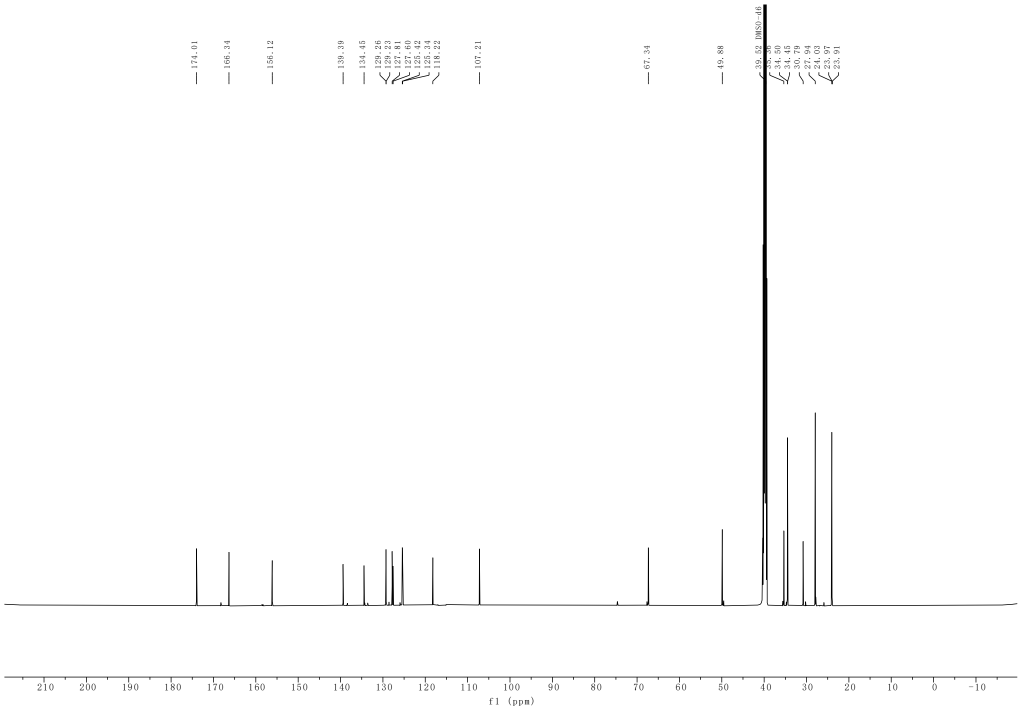

LC-MS Spectra (UV250) of compound **7**

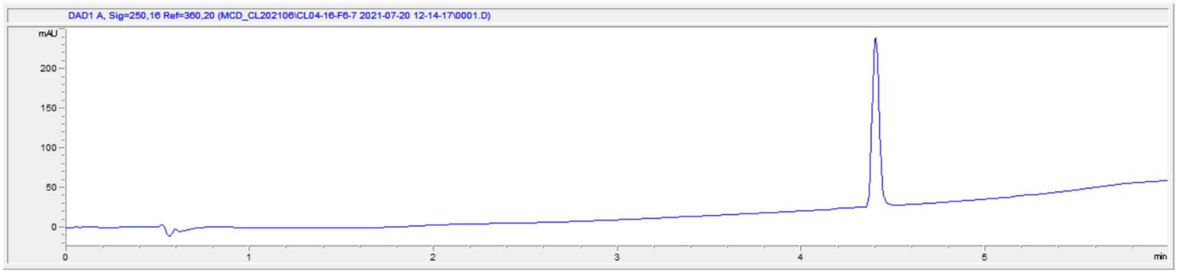

^{1}H NMR (600 MHz, DMSO-d_6) of compound **8**

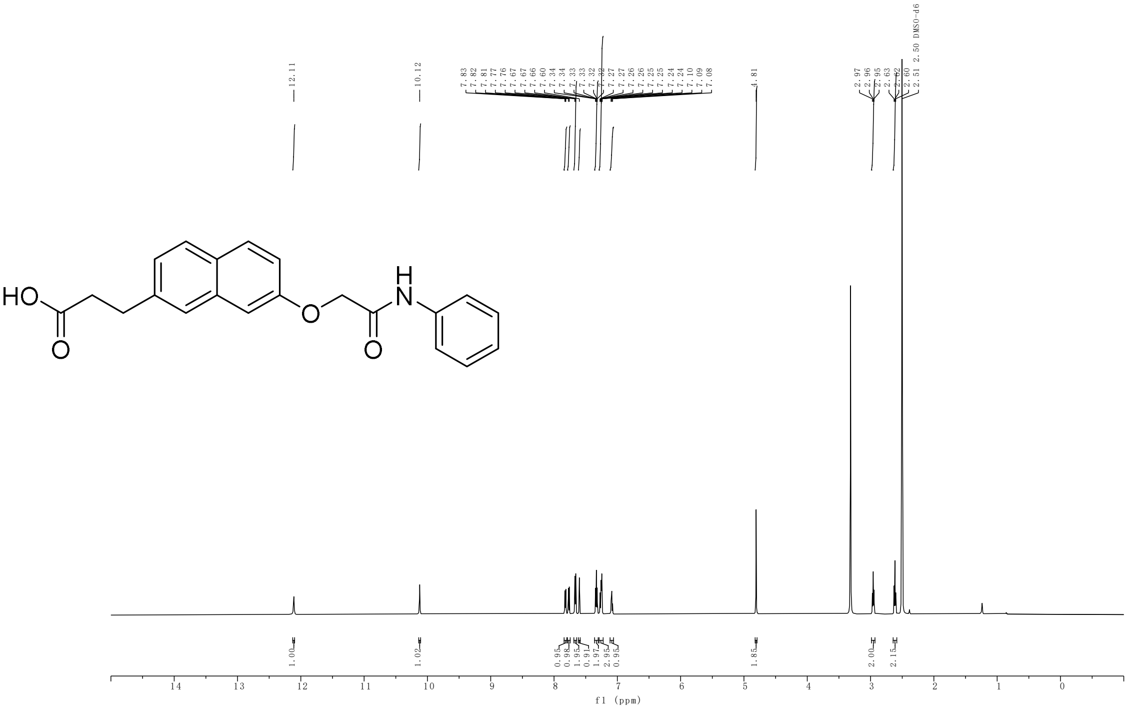

^{13}C NMR (151 MHz, DMSO-d_6) of compound **8**

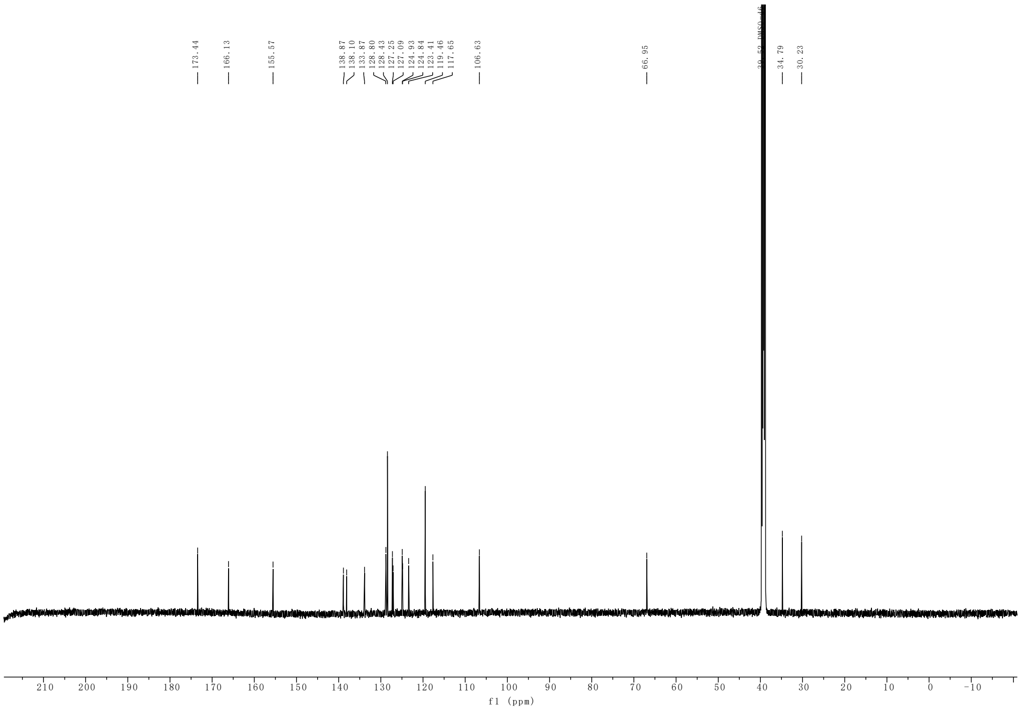

LC-MS Spectra (UV250) of compound **8**

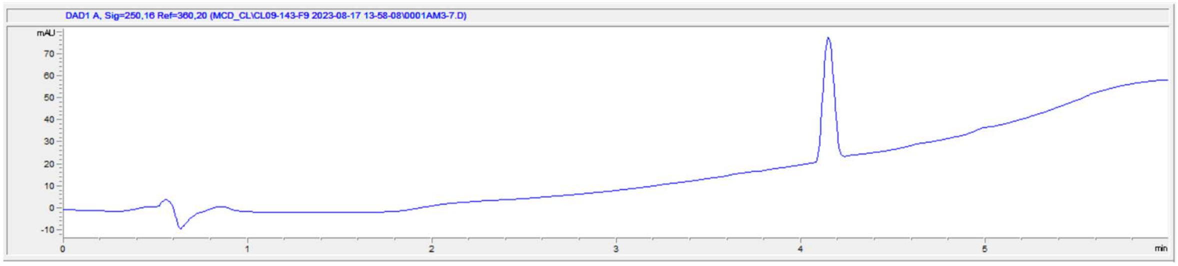

^{1}H NMR (600 MHz, DMSO-d_6) of compound **9**

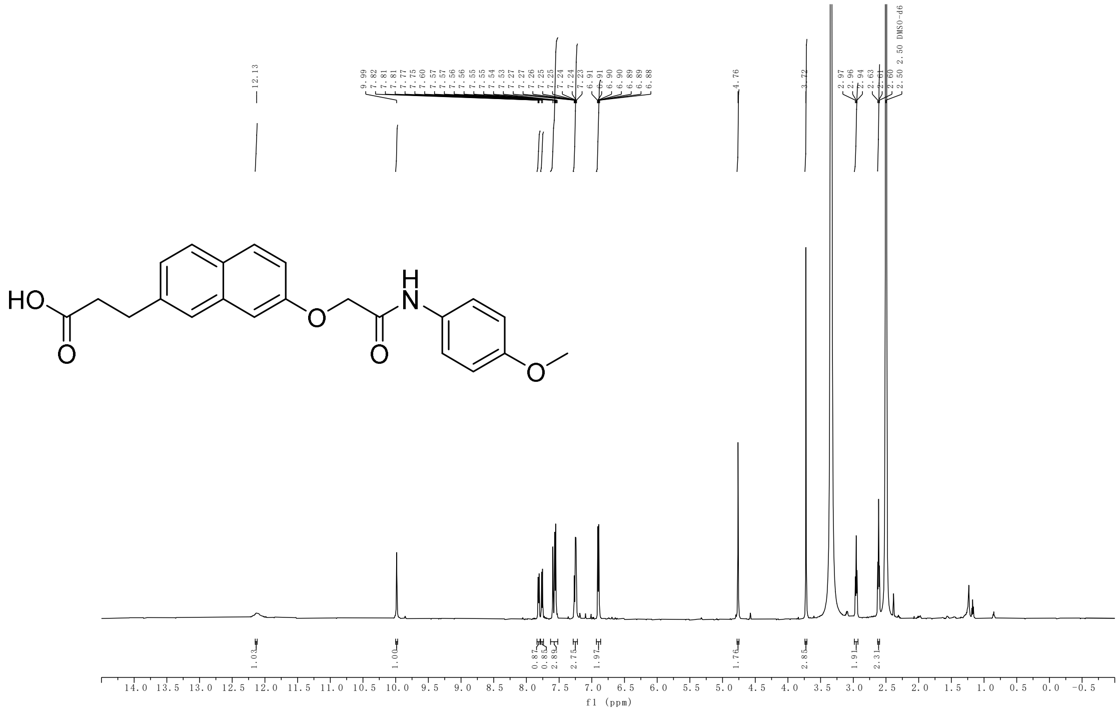

^{13}C NMR (151 MHz, DMSO-d_6) of compound **9**

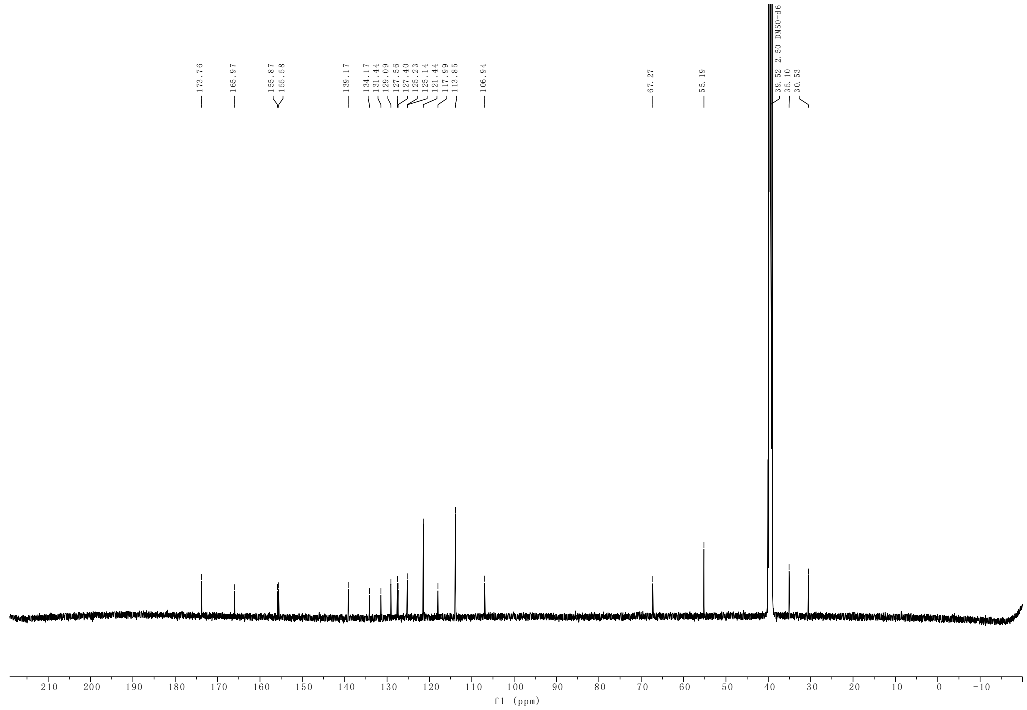

LC-MS Spectra (UV250) of compound **9**

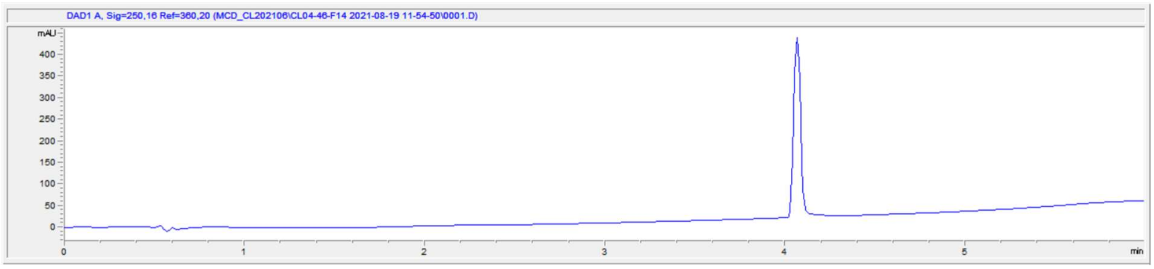

^{1}H NMR (600 MHz, DMSO-d_6) of compound **10**

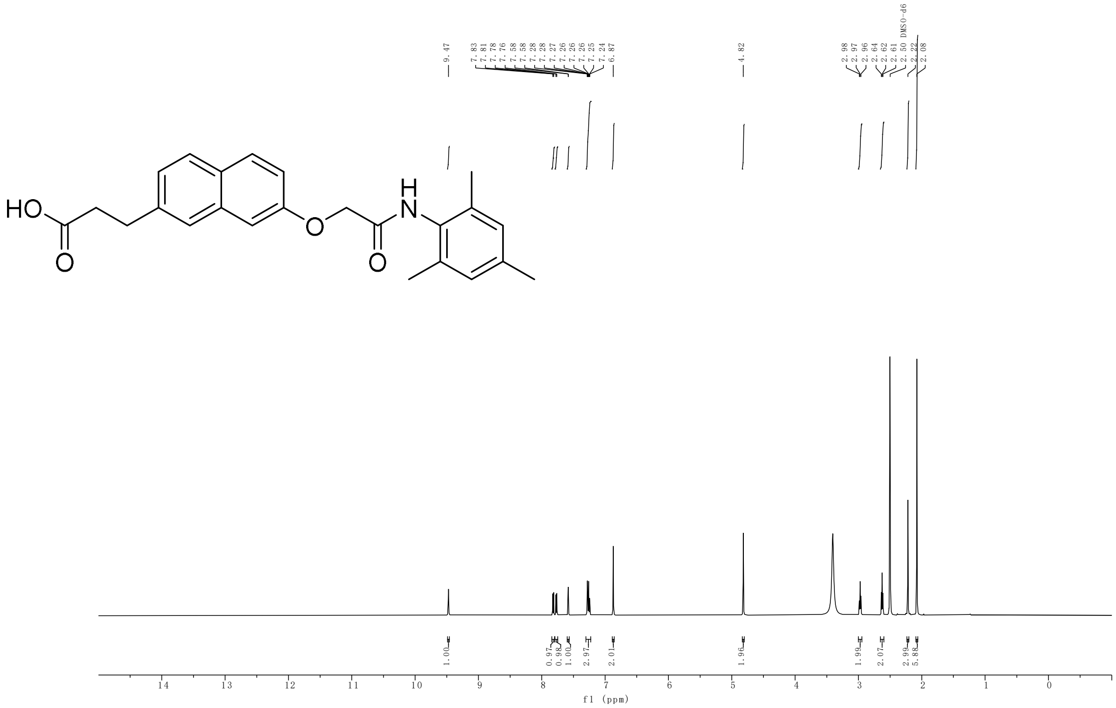

^{13}C NMR (151 MHz, DMSO-d_6) of compound **10**

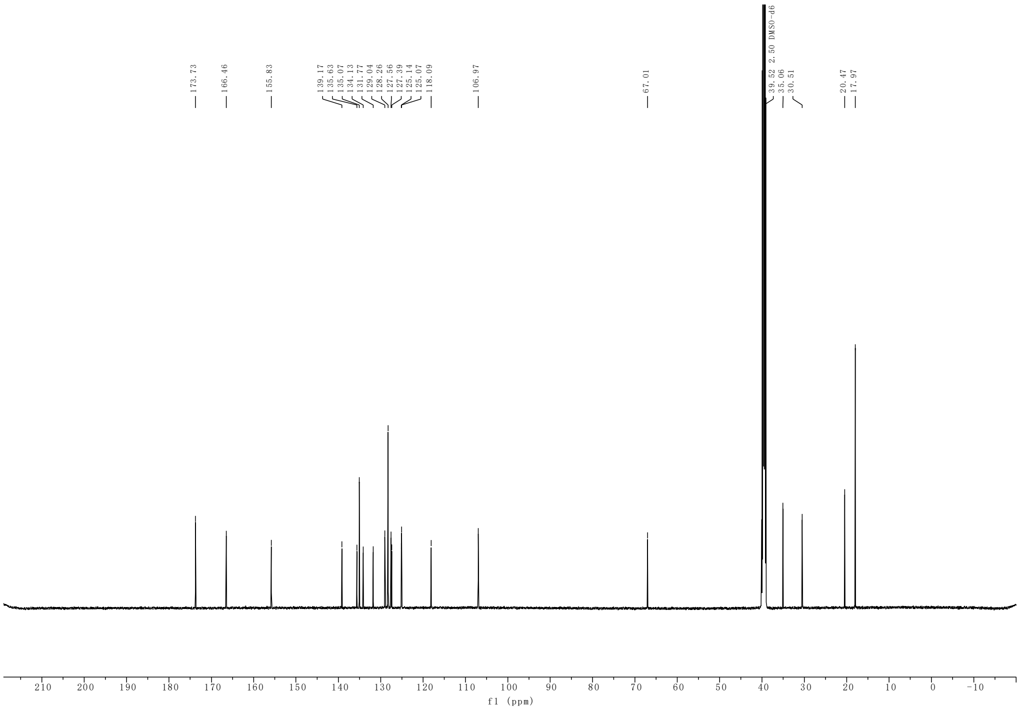

LC-MS Spectra (UV250) of compound **10**

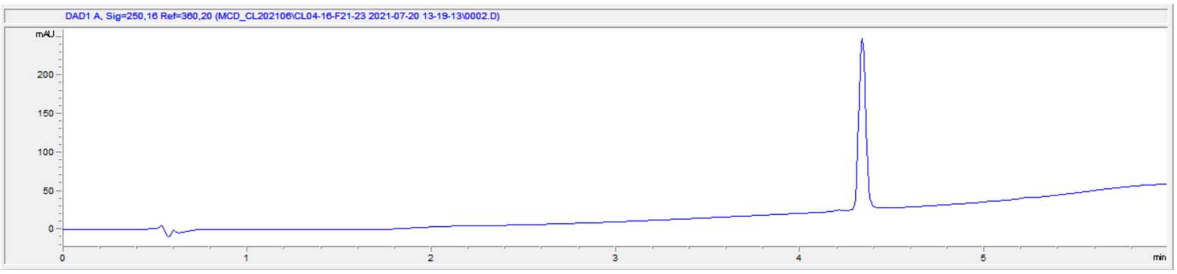

^{1}H NMR (600 MHz, DMSO-d_6) of compound **11**

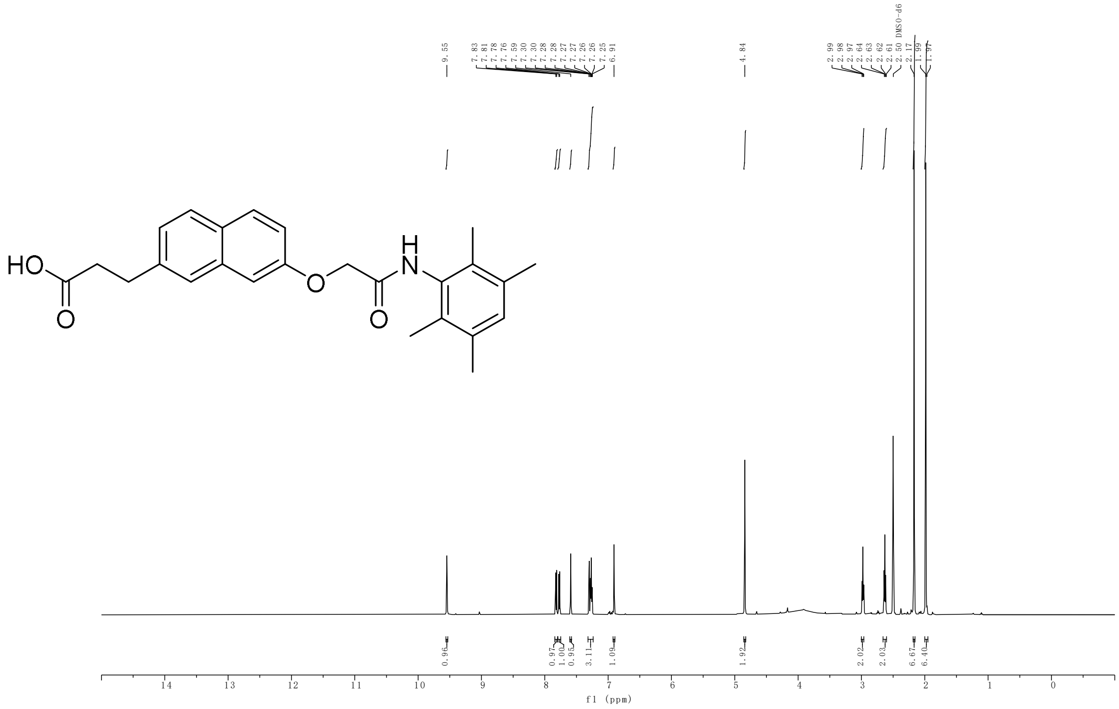

^{13}C NMR (151 MHz, DMSO-d_6) of compound **11**

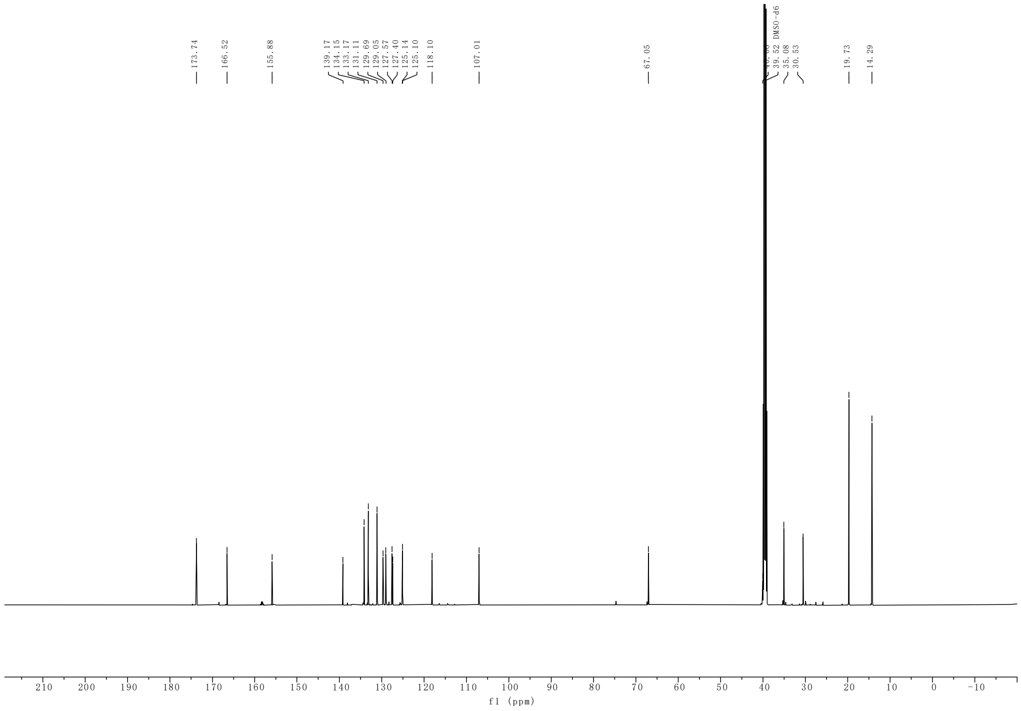

LC-MS Spectra (UV250) of compound **11**

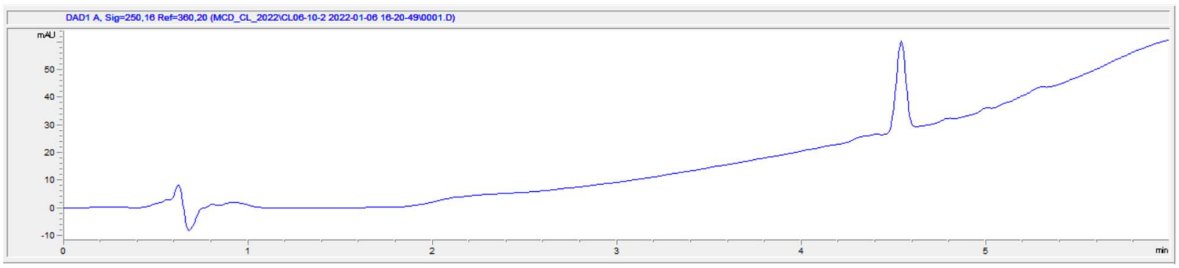

^{1}H NMR (600 MHz, DMSO-d_6) of compound **12**

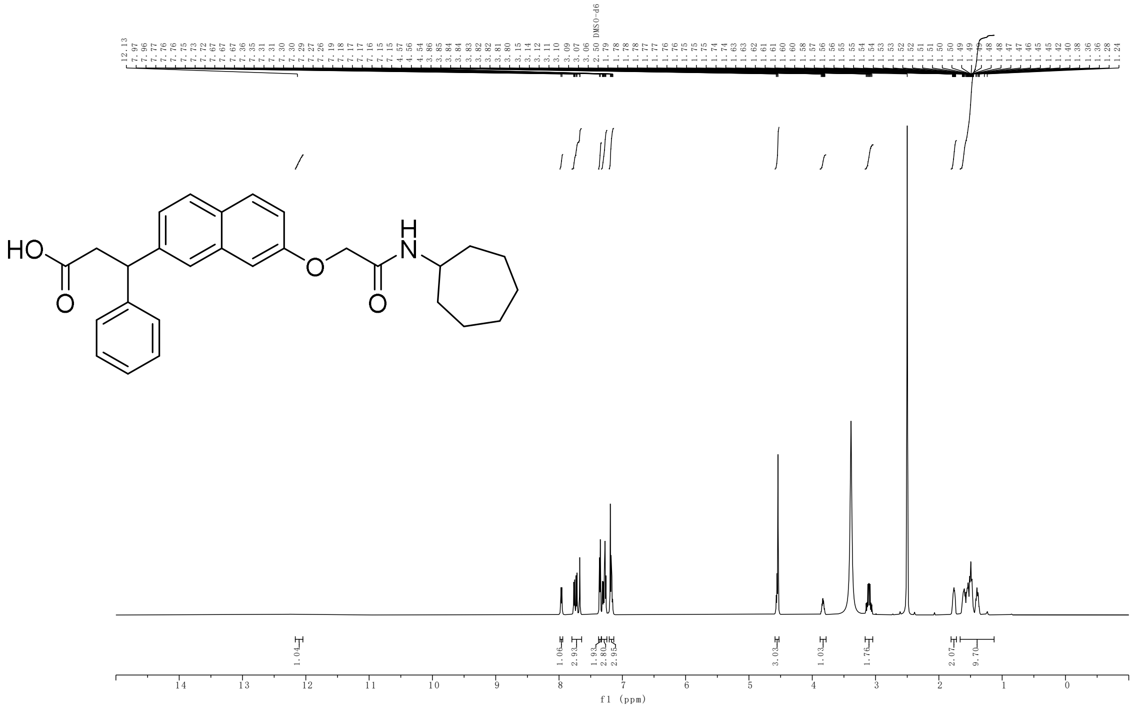

^{13}C NMR (151 MHz, DMSO-d_6) of compound **12**

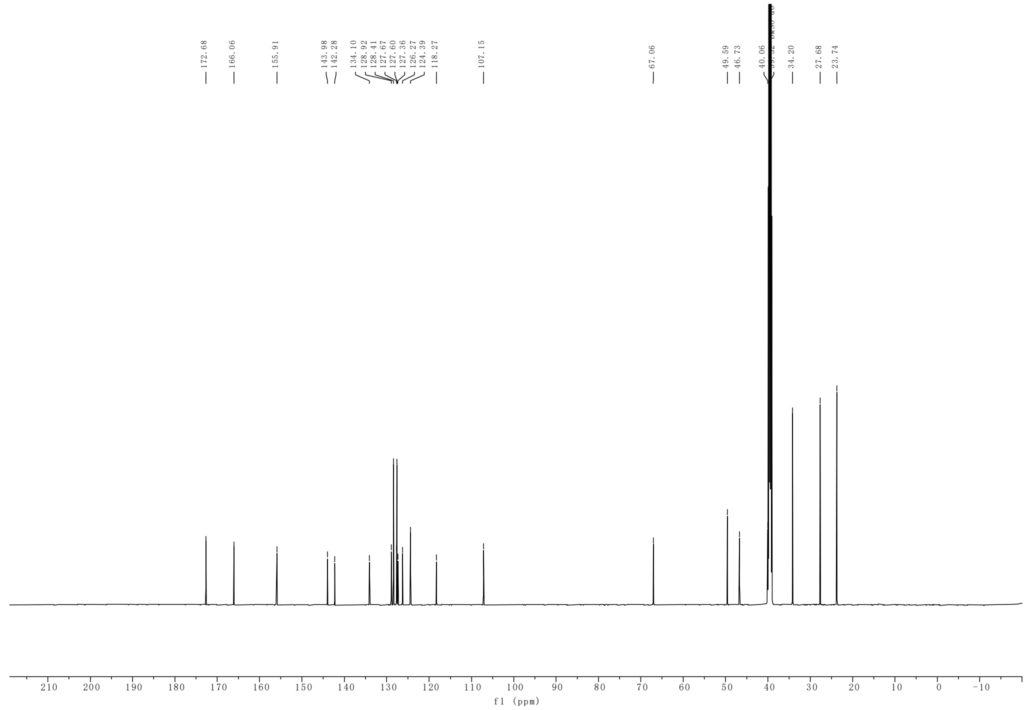

LC-MS Spectra (UV250) of compound **12**

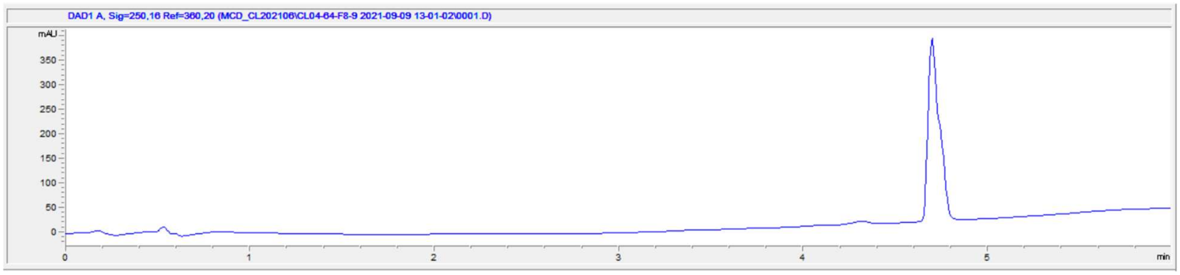

^{1}H NMR (600 MHz, DMSO-d_6) of compound **13**

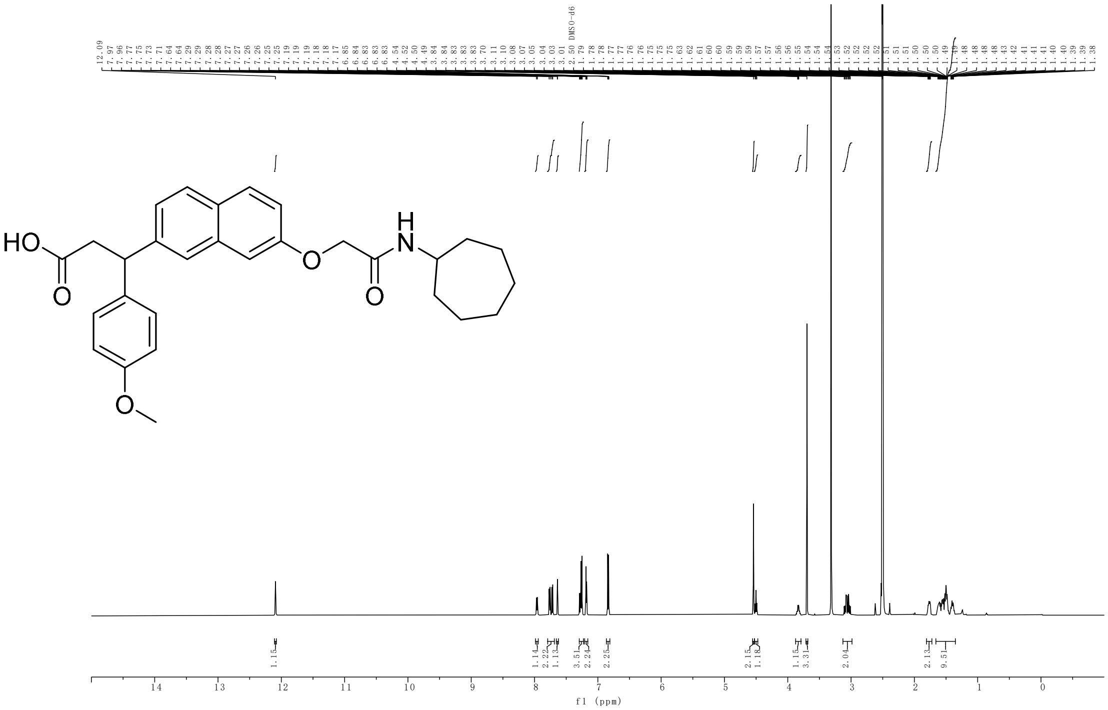

^{13}C NMR (151 MHz, DMSO-d_6) of compound **13**

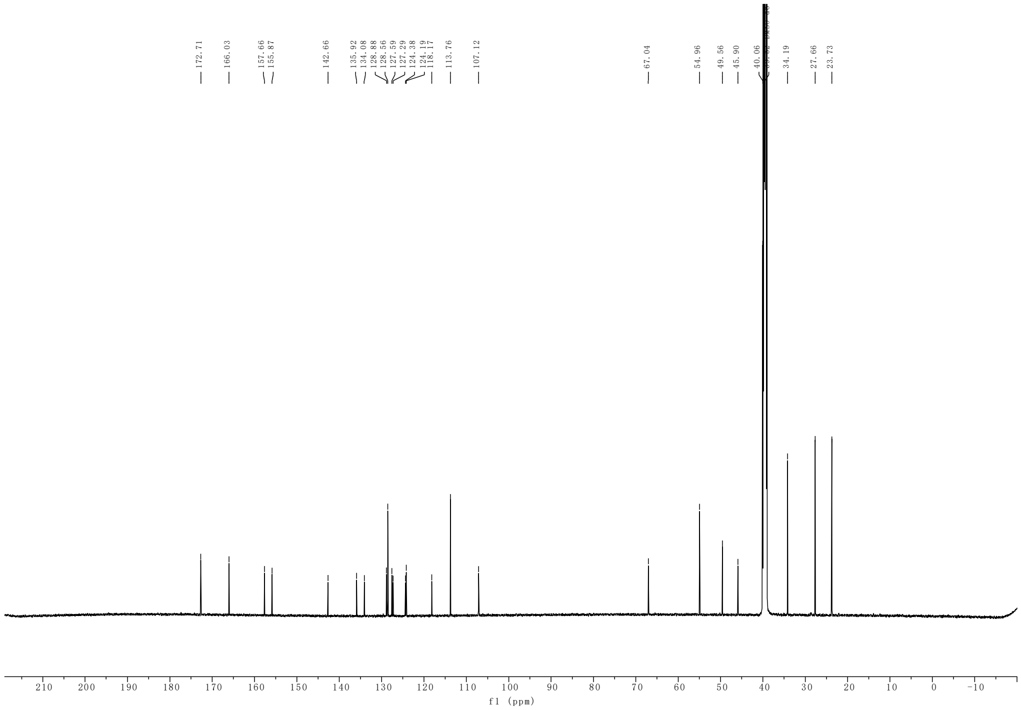

LC-MS Spectra (UV250) of compound **13**

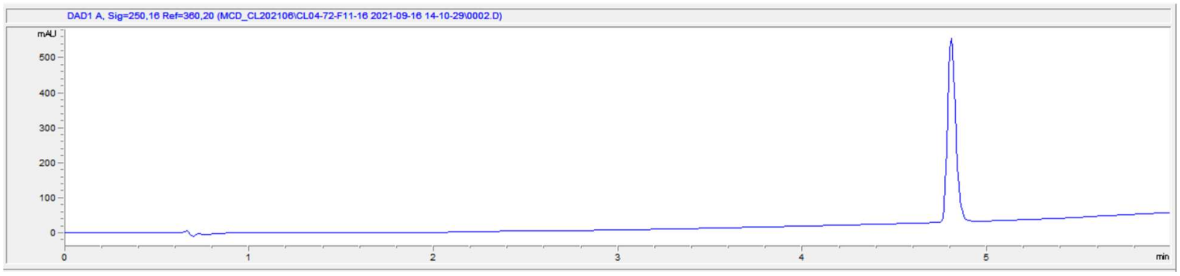

^{1}H NMR (600 MHz, DMSO-d_6) of compound **14**

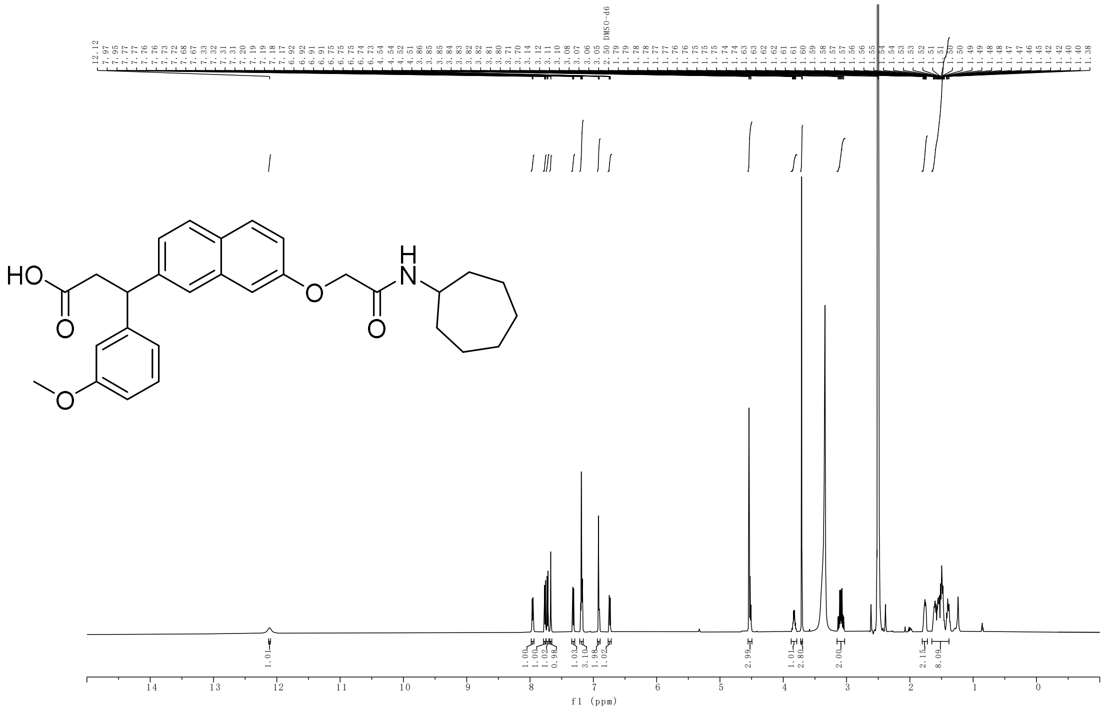

^{13}C NMR (151 MHz, DMSO-d_6) of compound **14**

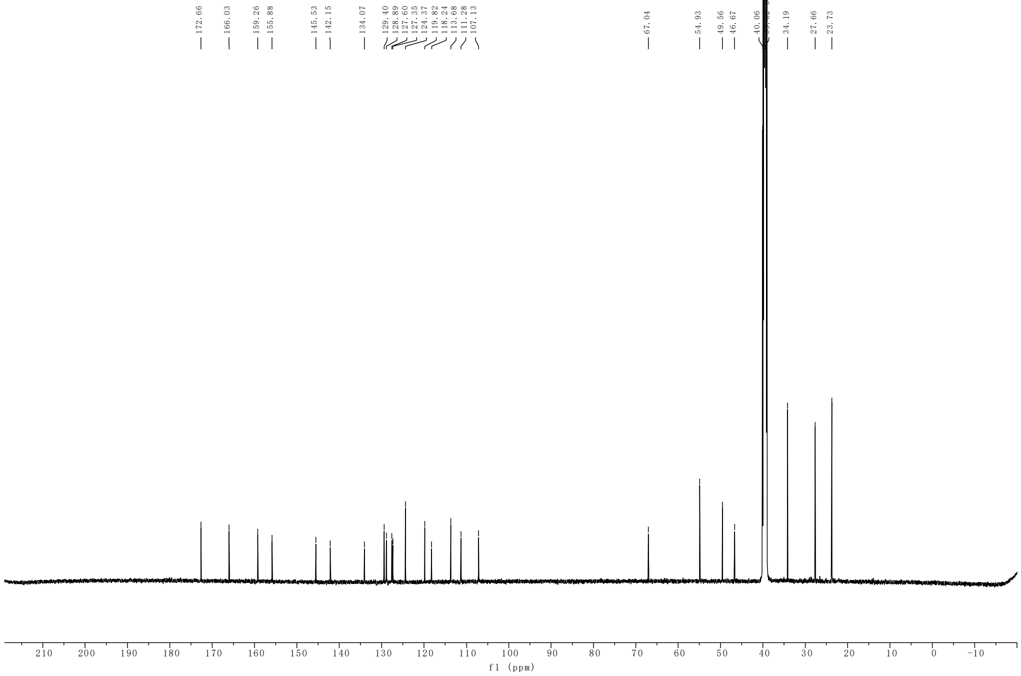

LC-MS Spectra (UV250) of compound **14**

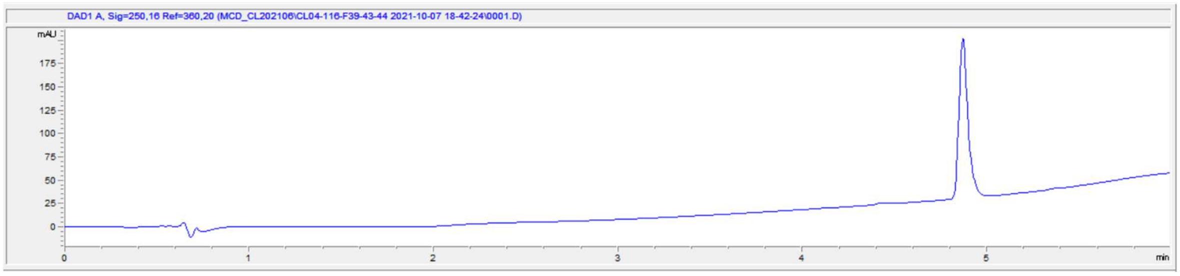

^{1}H NMR (600 MHz, DMSO-d_6) of compound **15**

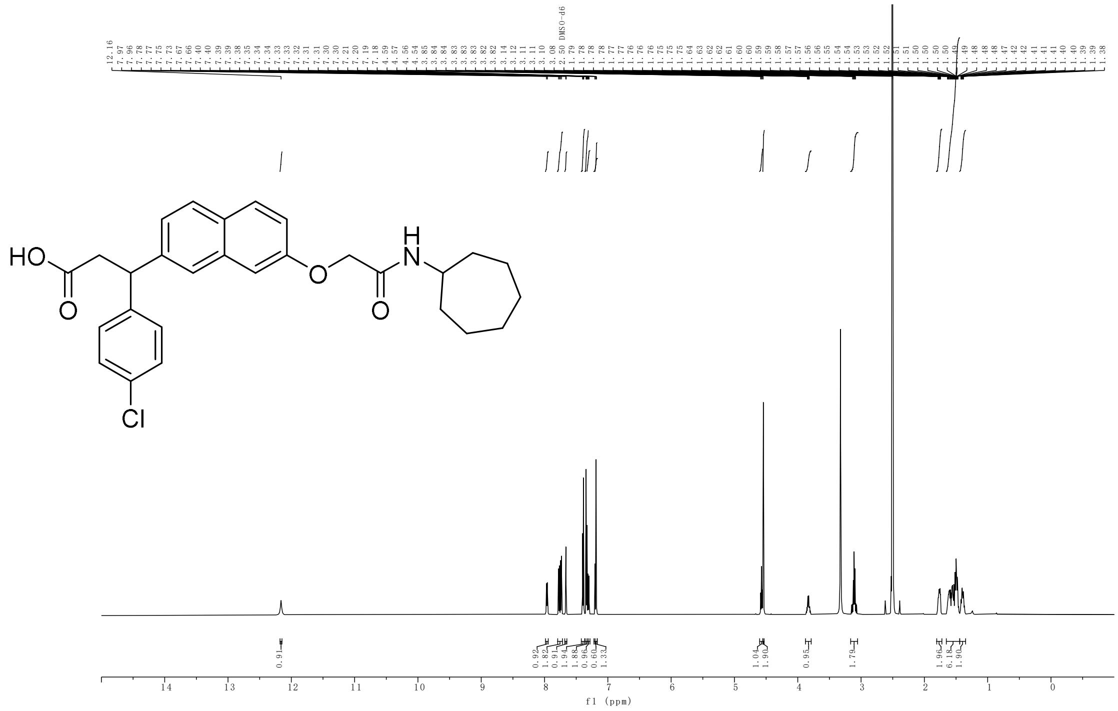

^{13}C NMR (151 MHz, DMSO-d_6) of compound **15**

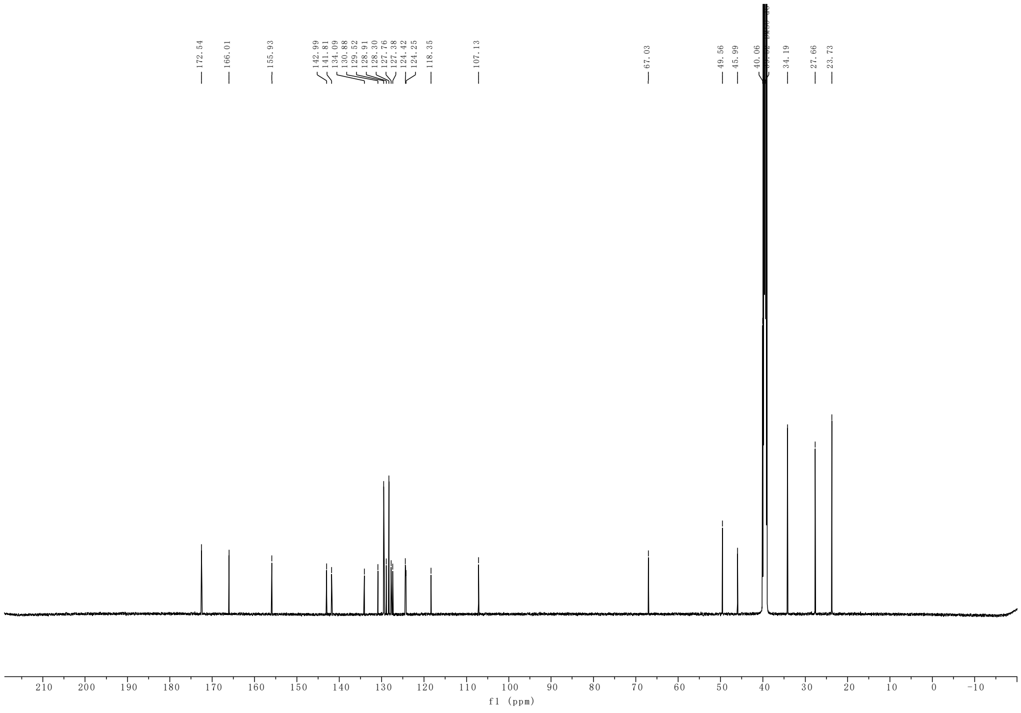

LC-MS Spectra (UV250) of compound **15**

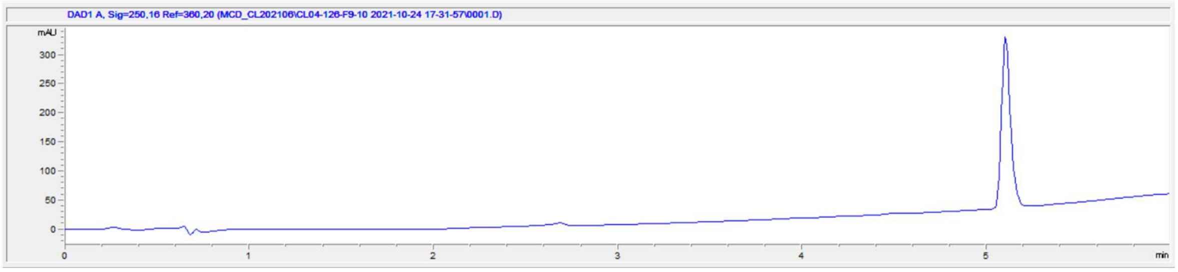

^{1}H NMR (600 MHz, DMSO-d_6) of compound **16**

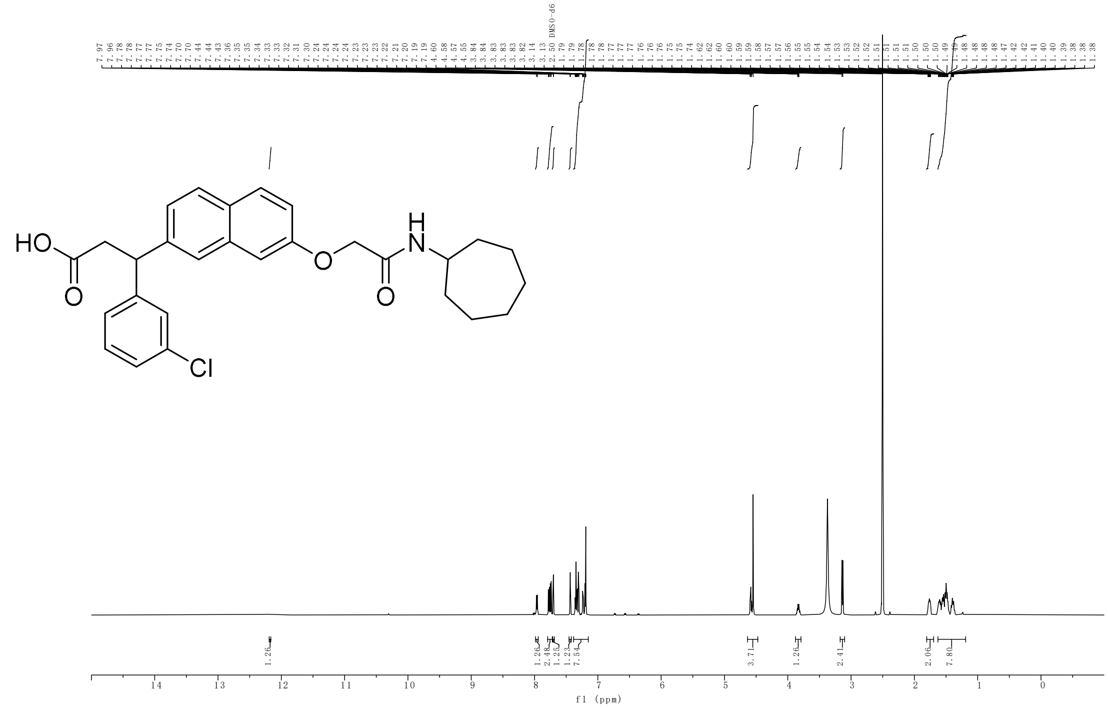

^{13}C NMR (151 MHz, DMSO-d_6) of compound **16**

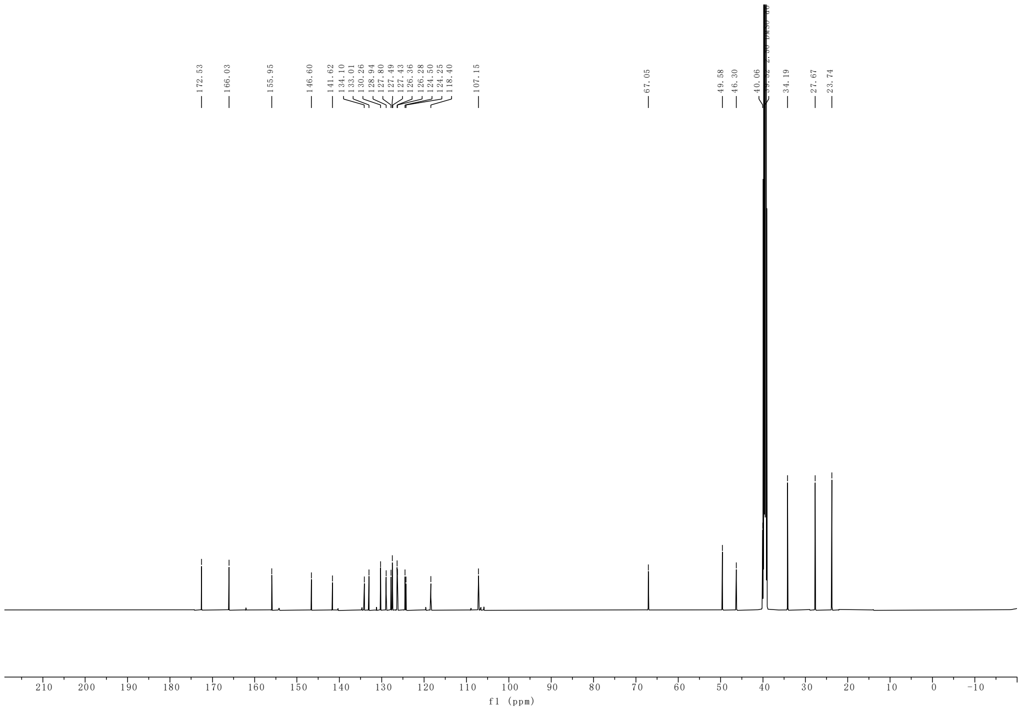

LC-MS Spectra (UV250) of compound **16**

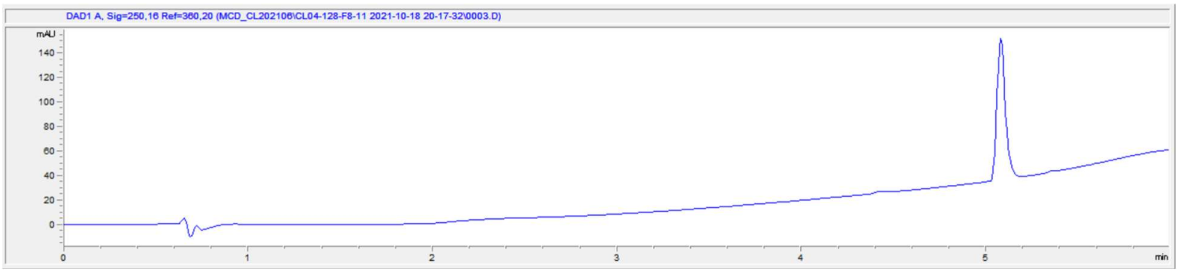

^{1}H NMR (600 MHz, DMSO-d_6) of compound **17**

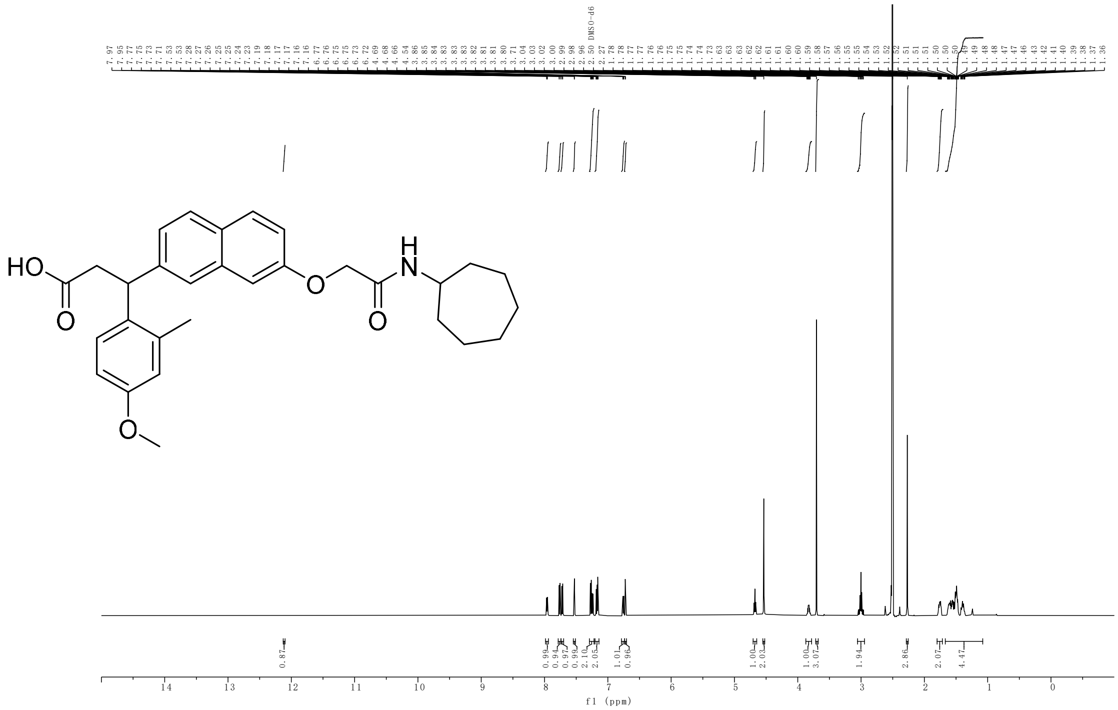

^{13}C NMR (151 MHz, DMSO-d_6) of compound **17**

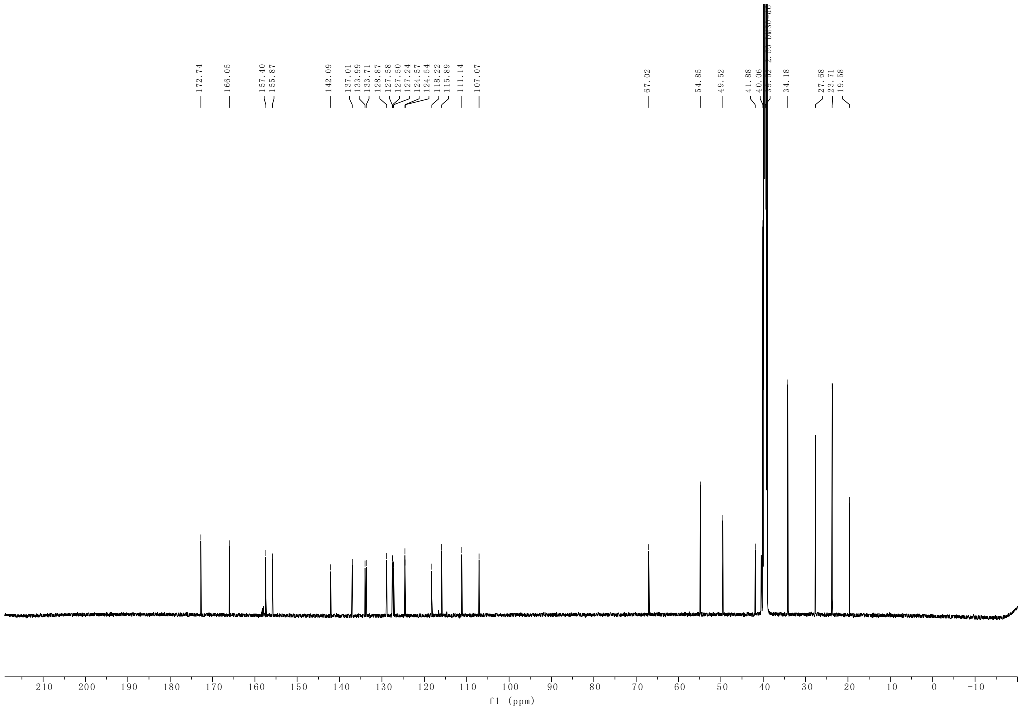

LC-MS Spectra (UV250) of compound **17**

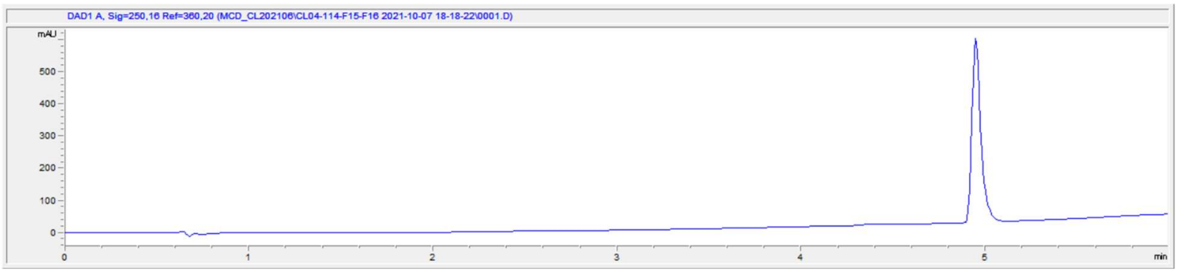

^{1}H NMR (600 MHz, DMSO-d_6) of compound **18**

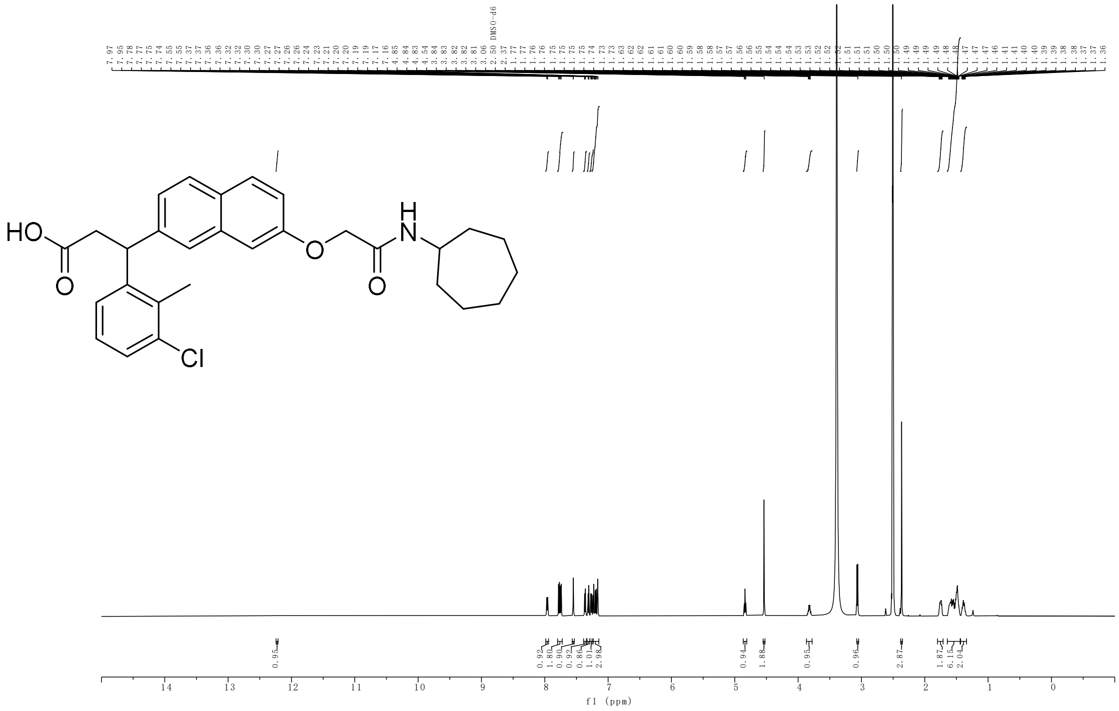

^{13}C NMR (151 MHz, DMSO-d_6) of compound **18**

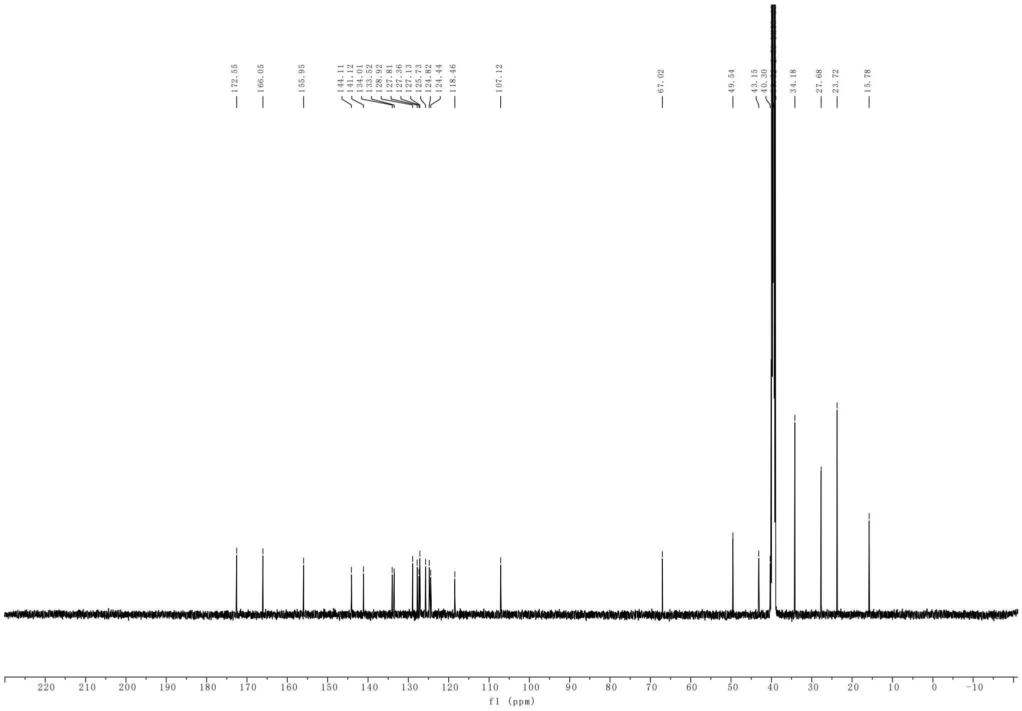

LC-MS Spectra (UV250) of compound **18**

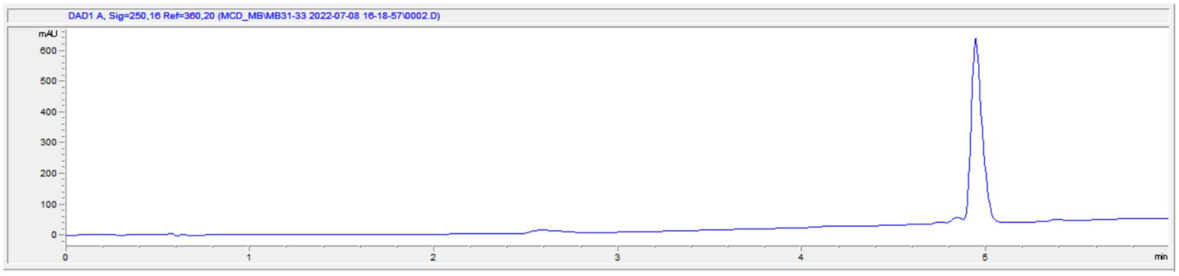

^{1}H NMR (600 MHz, DMSO-d_6) of compound **19**

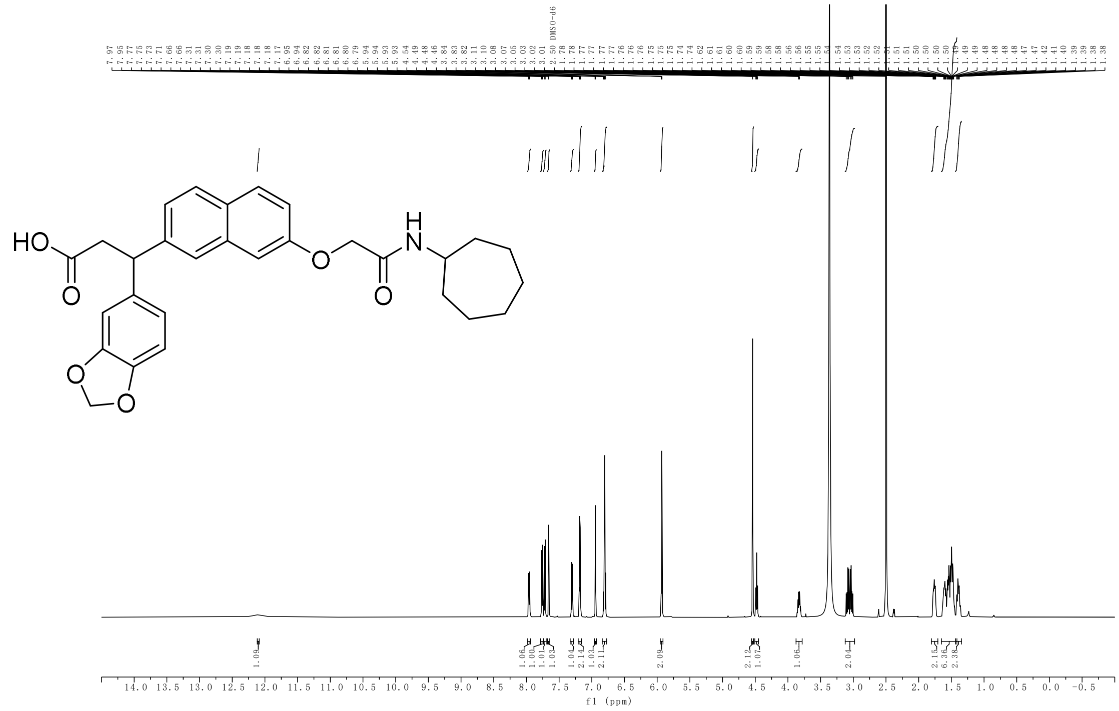

^{13}C NMR (151 MHz, DMSO-d_6) of compound **19**

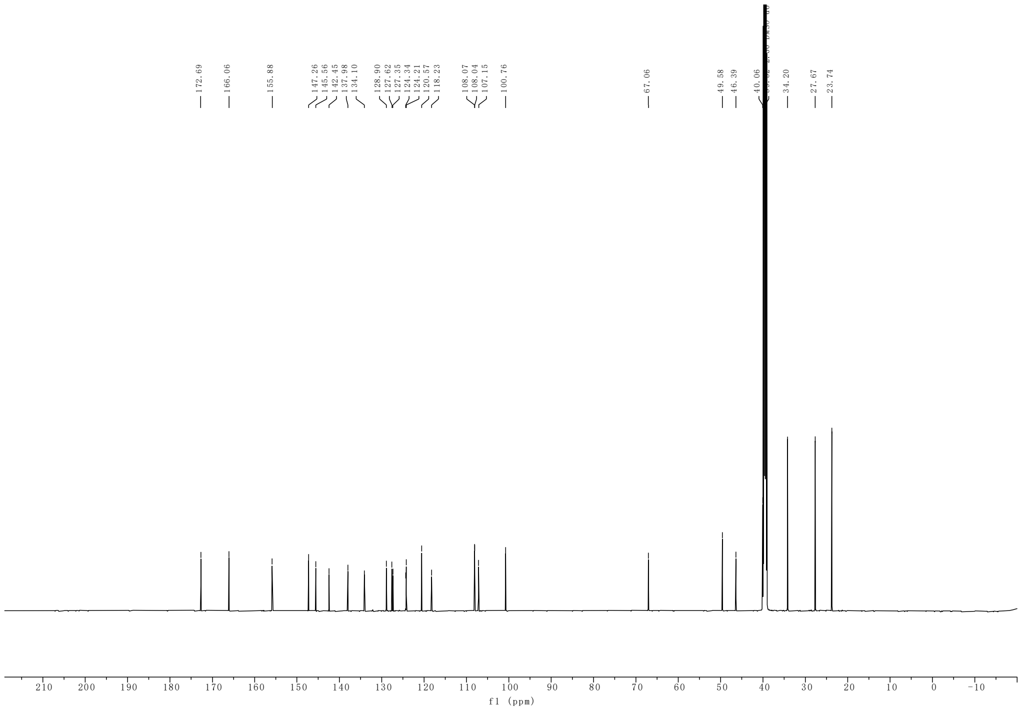

LC-MS Spectra (UV250) of compound **19**

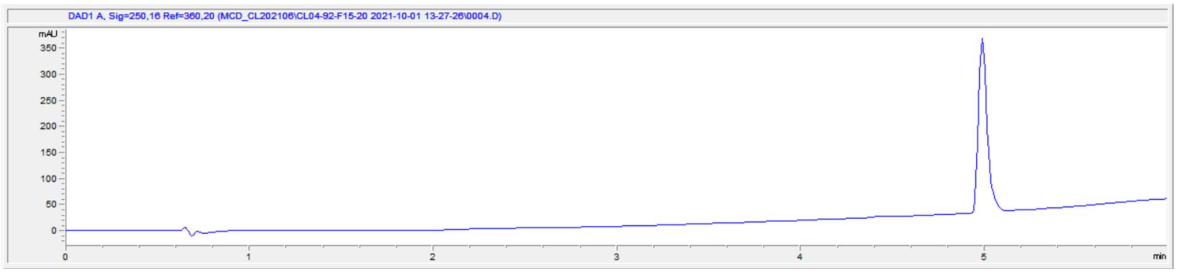

^{1}H NMR (600 MHz, DMSO-d_6) of compound **20**

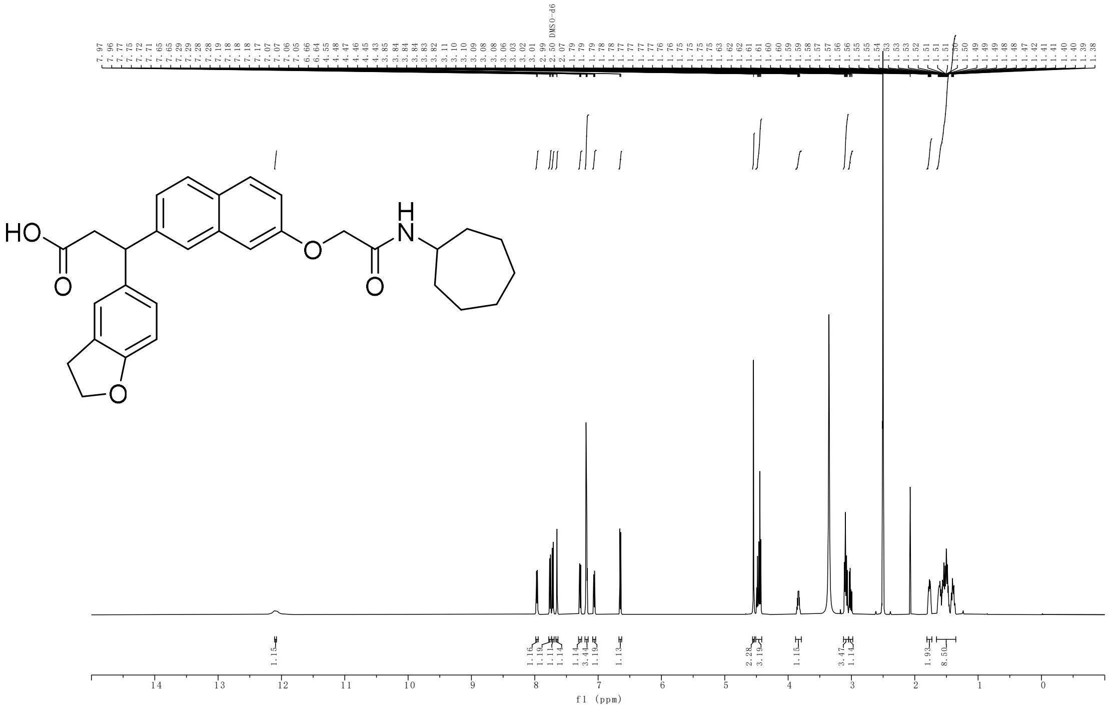

^{13}C NMR (151 MHz, DMSO-d_6) of compound **20**

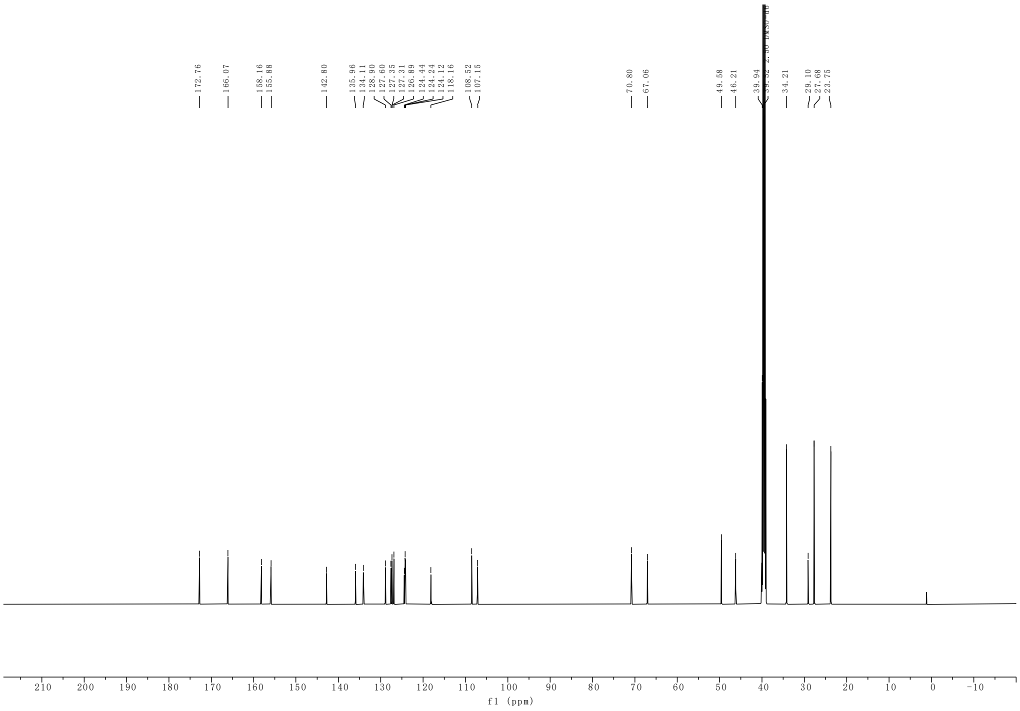

LC-MS Spectra (UV250) of compound **20**

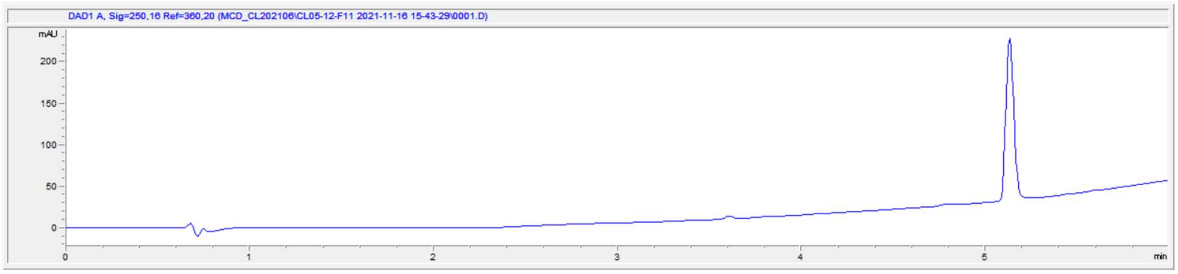

^{1}H NMR (600 MHz, DMSO-d_6) of compound **21**

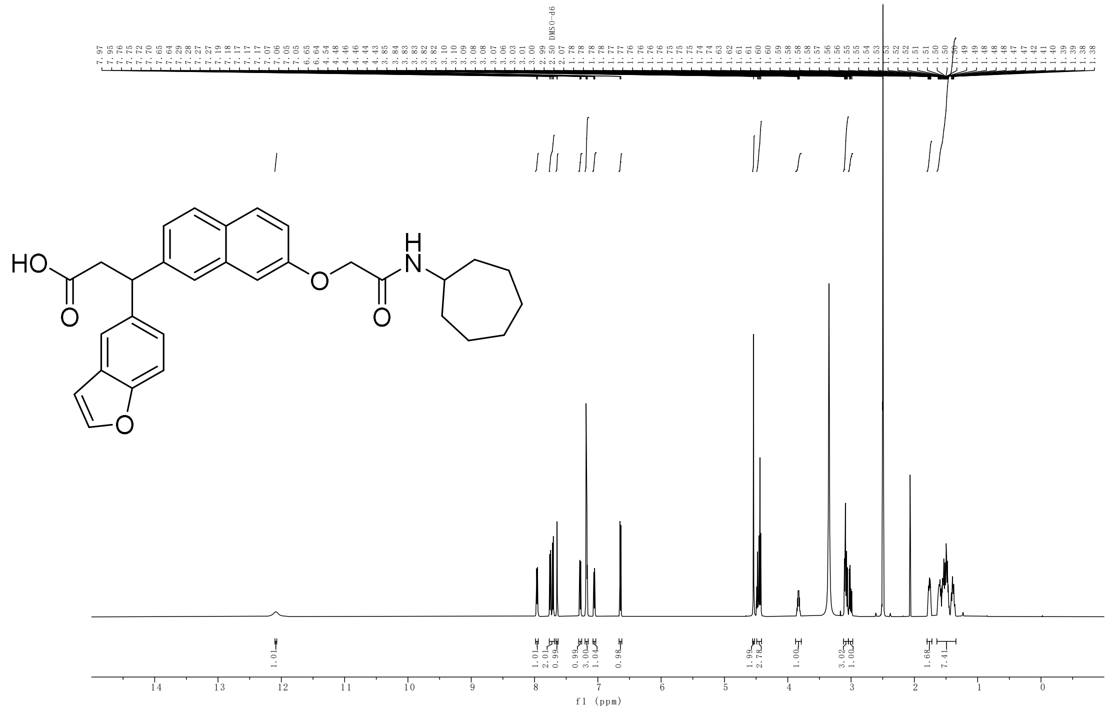

^{13}C NMR (151 MHz, DMSO-d_6) of compound **21**

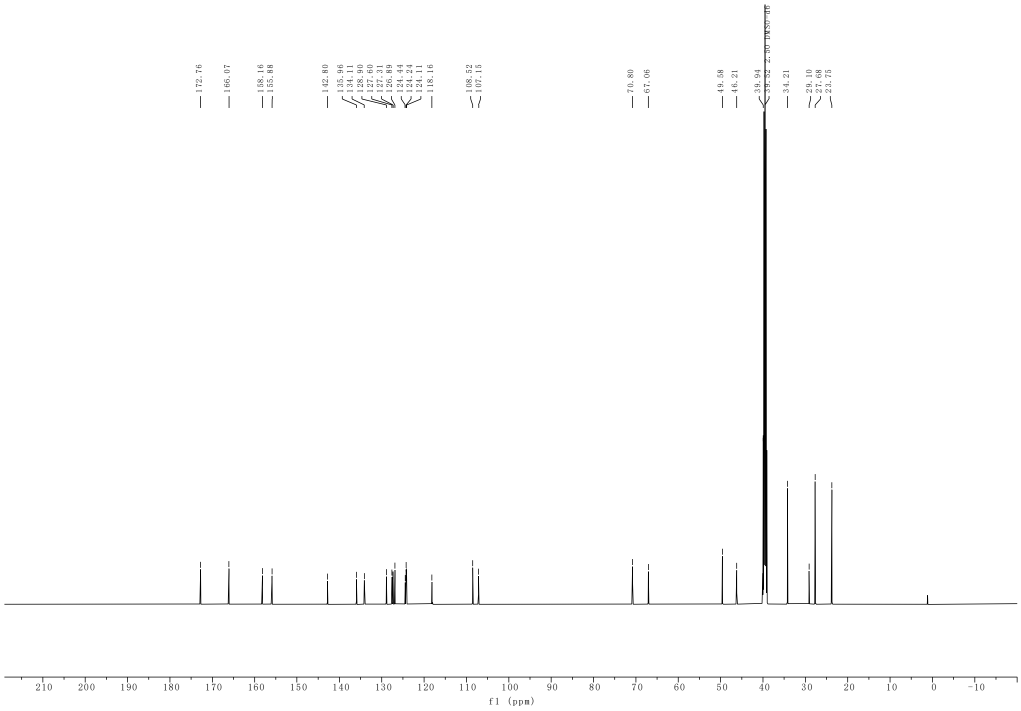

LC-MS Spectra (UV250) of compound **21**

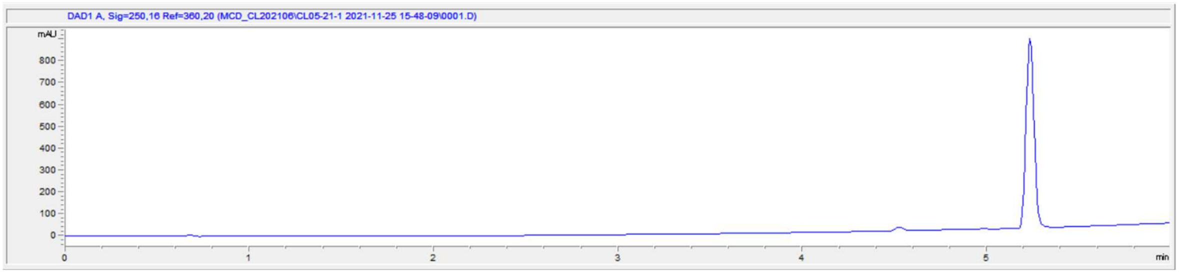

^{1}H NMR (600 MHz, DMSO-d_6) of compound **22**

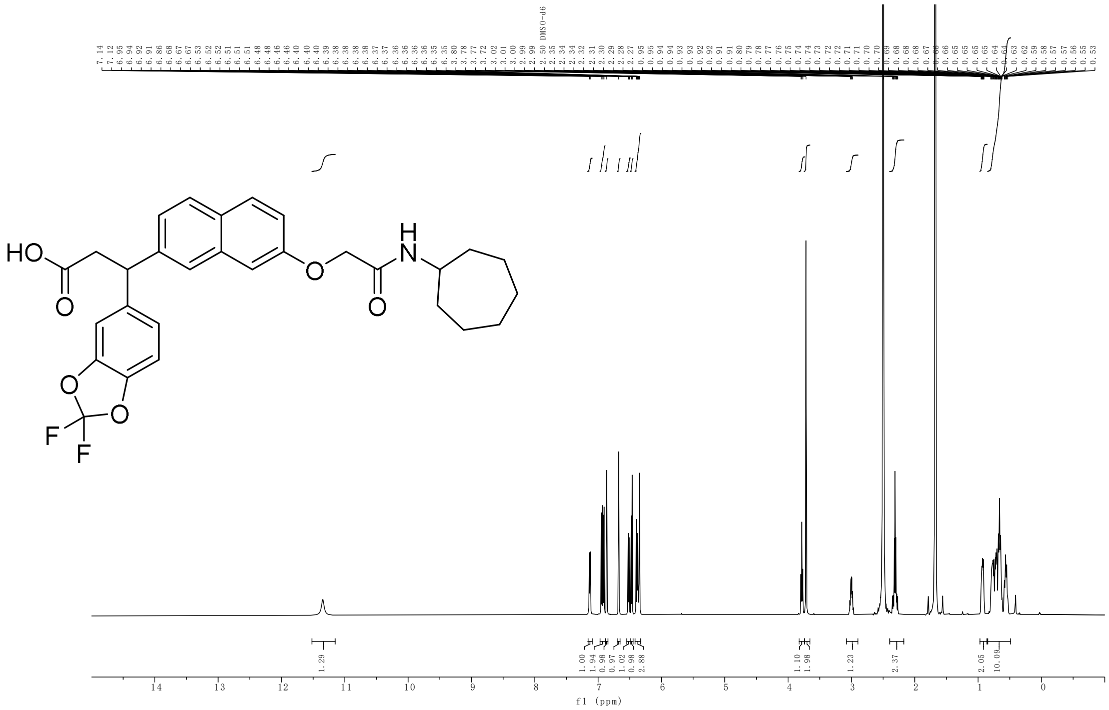

^{13}C NMR (151 MHz, DMSO-d_6) of compound **22**

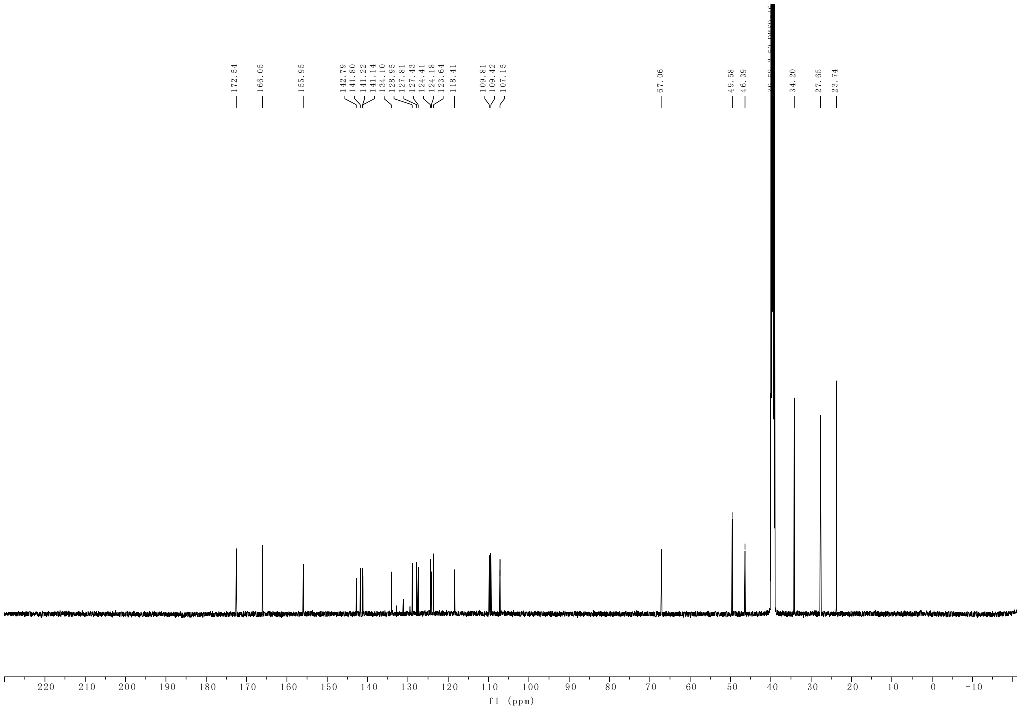

LC-MS Spectra (UV250) of compound **22**

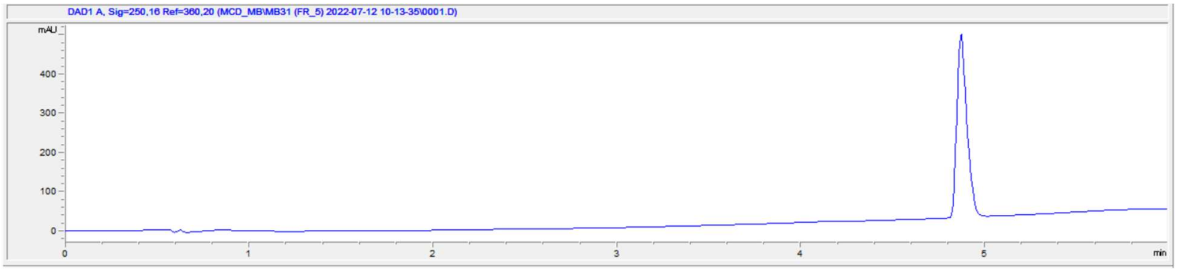

^1H NMR (600 MHz, DMSO-d_6) of compound **23**

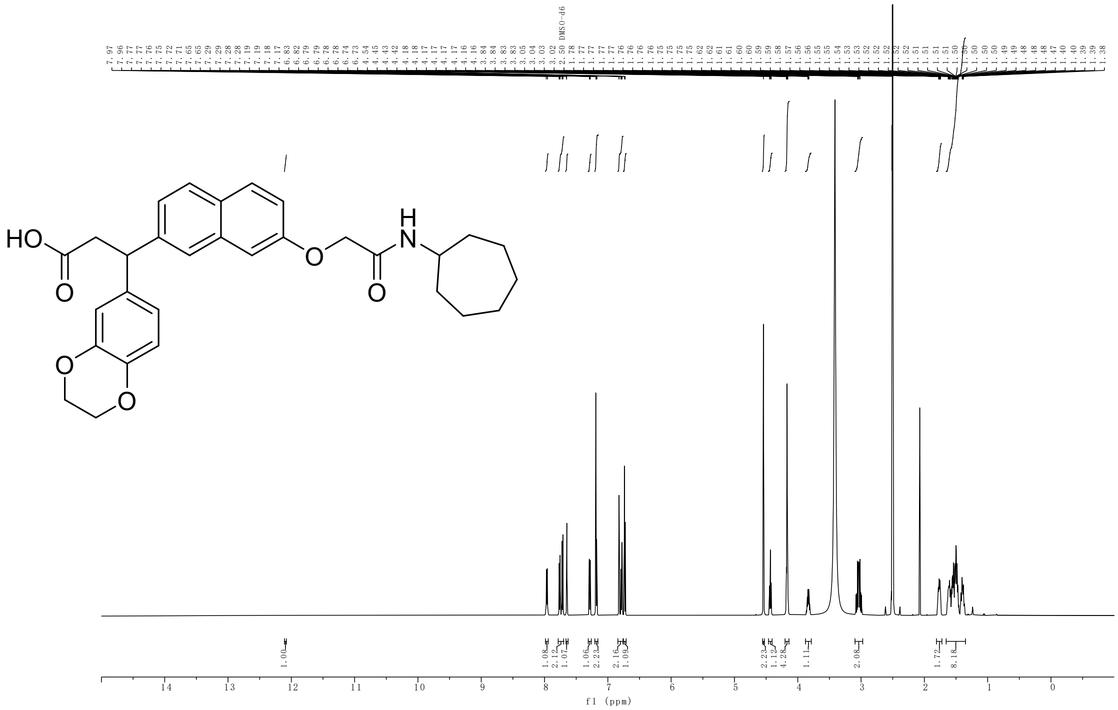

^{13}C NMR (151 MHz, DMSO-d_6) of compound **23**

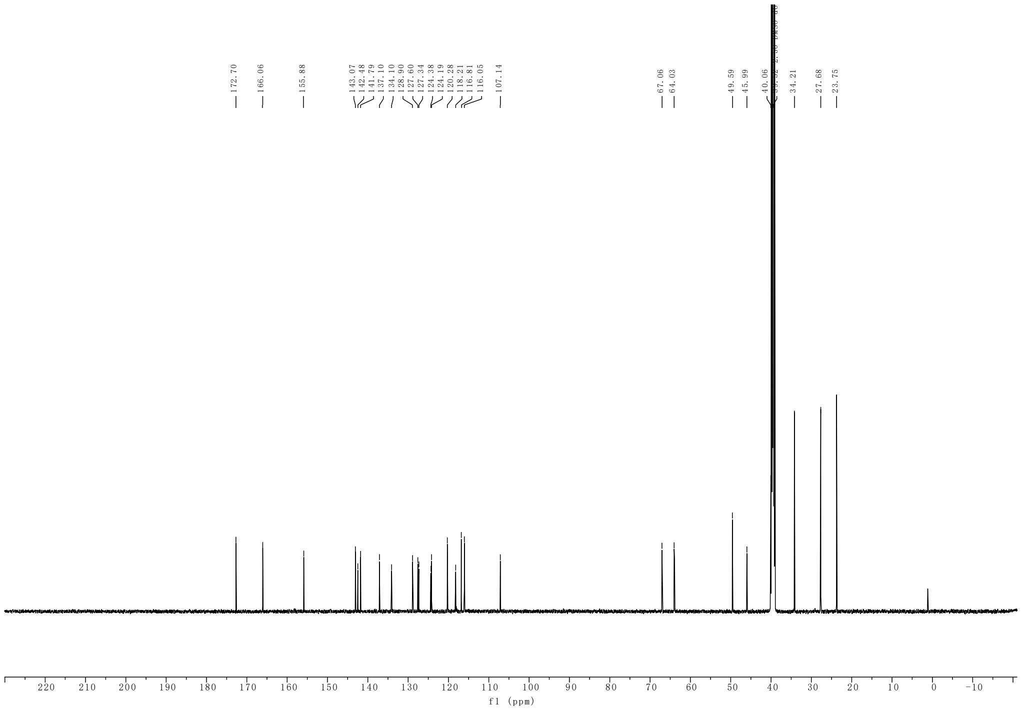

LC-MS Spectra (UV250) of compound **23**

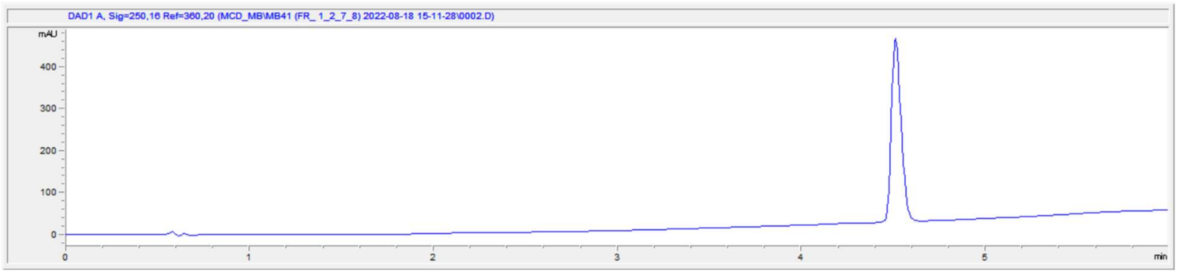

^{1}H NMR (600 MHz, DMSO-d_6) of compound **24**

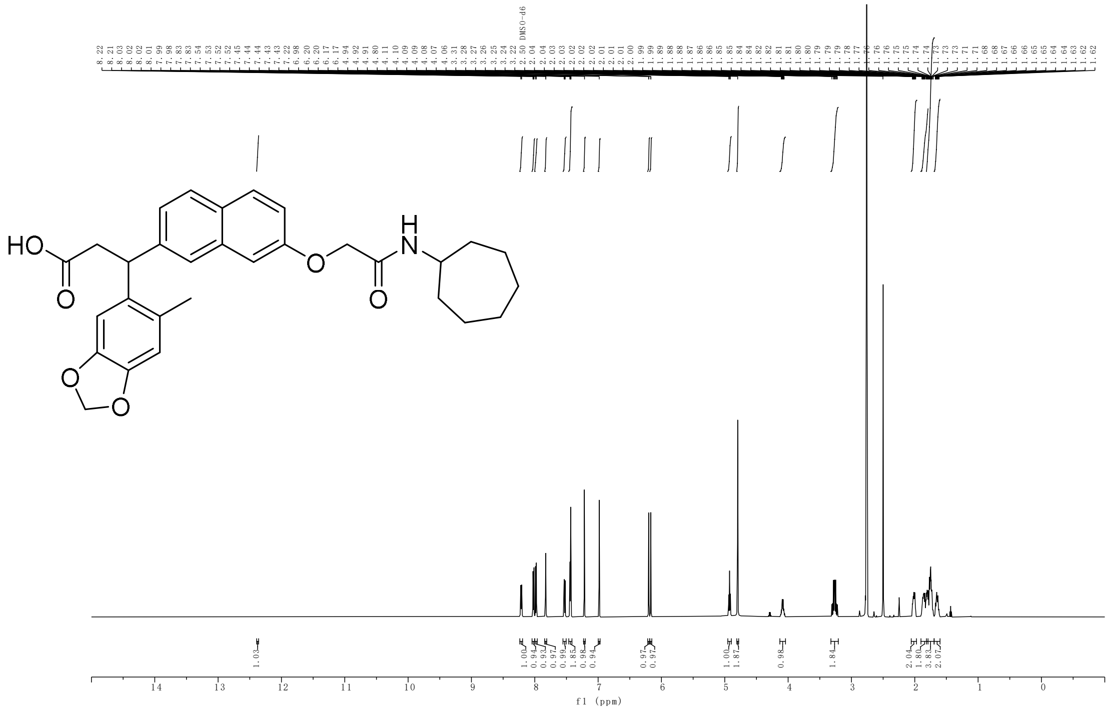

^{13}C NMR (151 MHz, DMSO-d_6) of compound **24**

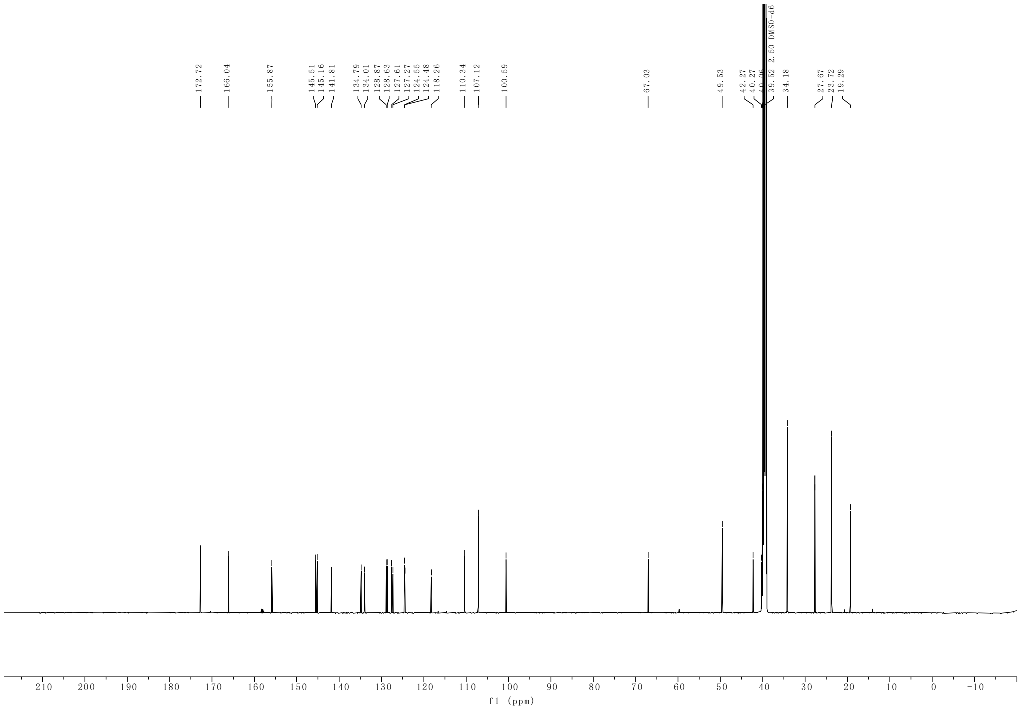

LC-MS Spectra (UV250) of compound **24**

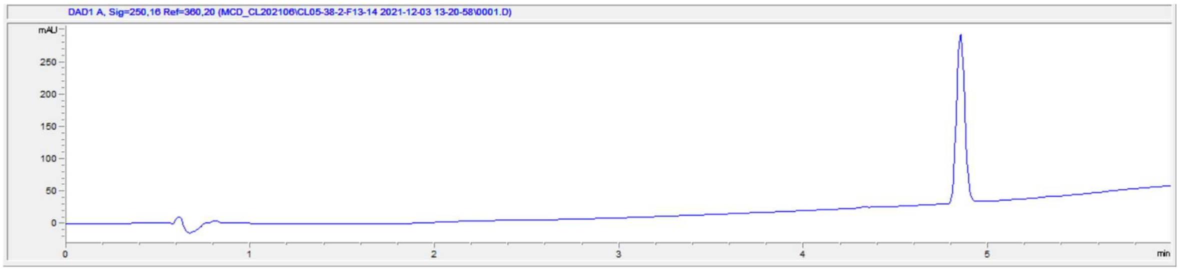

^{1}H NMR (600 MHz, DMSO-d_6) of compound **25**

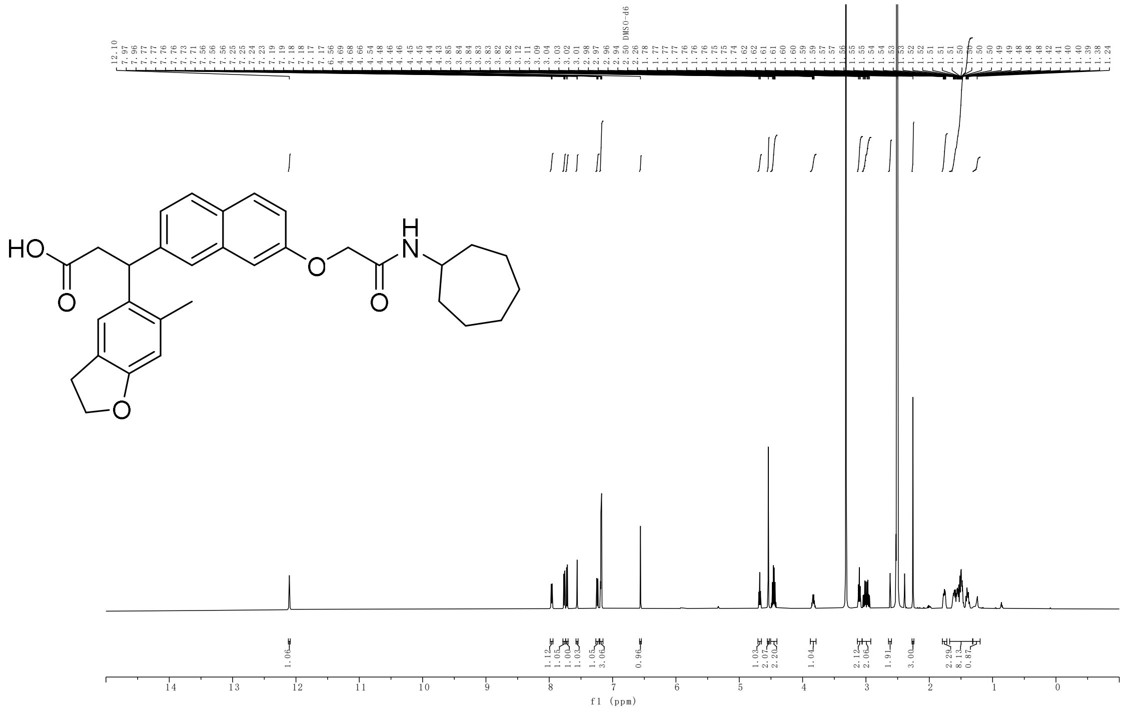

^{13}C NMR (151 MHz, DMSO-d_6) of compound **25**

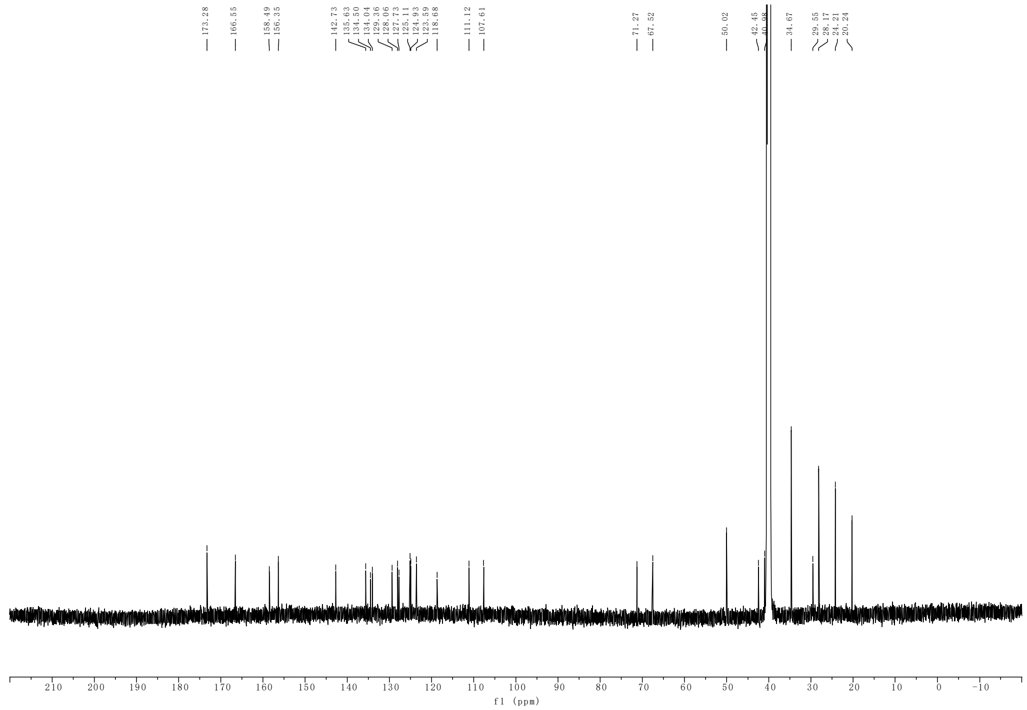

LC-MS Spectra (UV250) of compound **25**

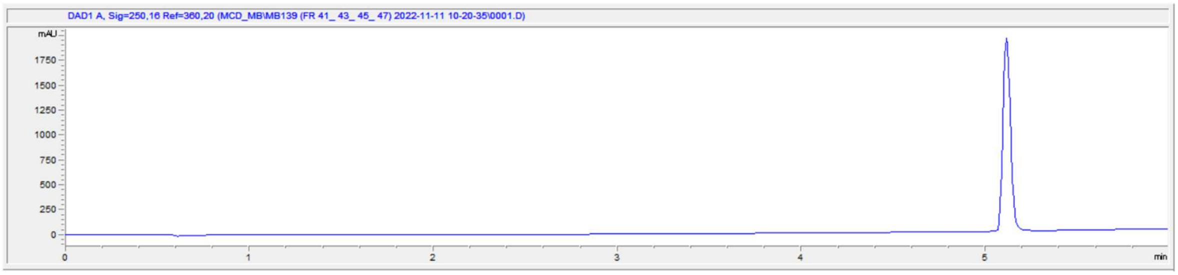

^1H NMR (600 MHz, DMSO-d_6) of compound **26**

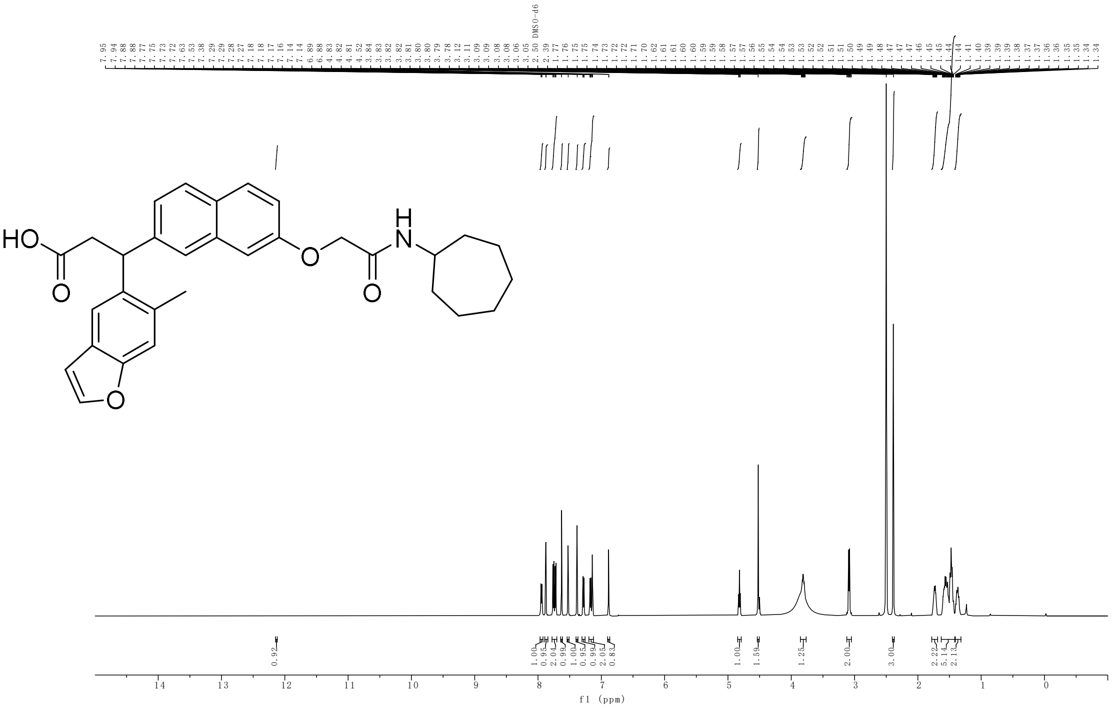

^{13}C NMR (151 MHz, DMSO-d_6) of compound **26**

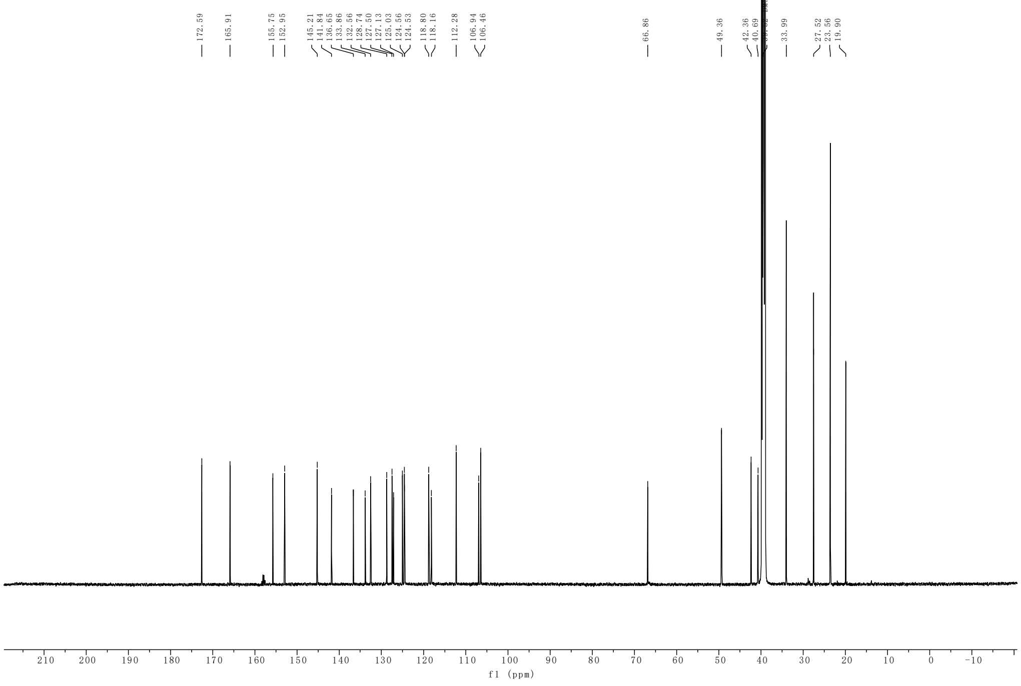

LC-MS Spectra (UV250) of compound **26**

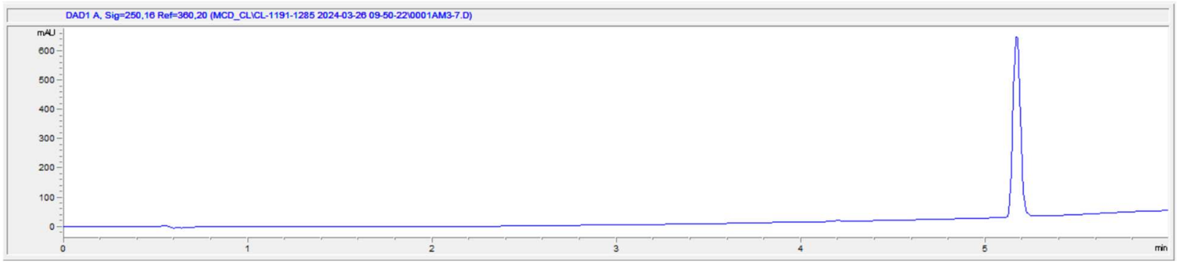

^{1}H NMR (600 MHz, DMSO-d_6) of compound **27**

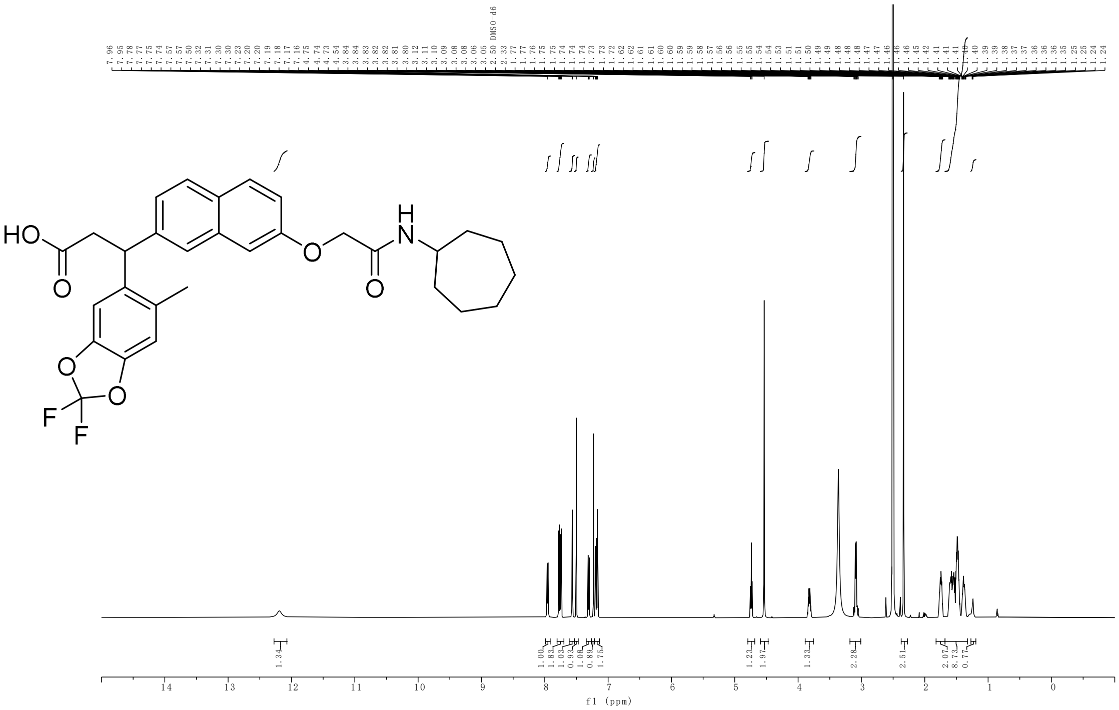

^{13}C NMR (151 MHz, DMSO-d_6) of compound **27**

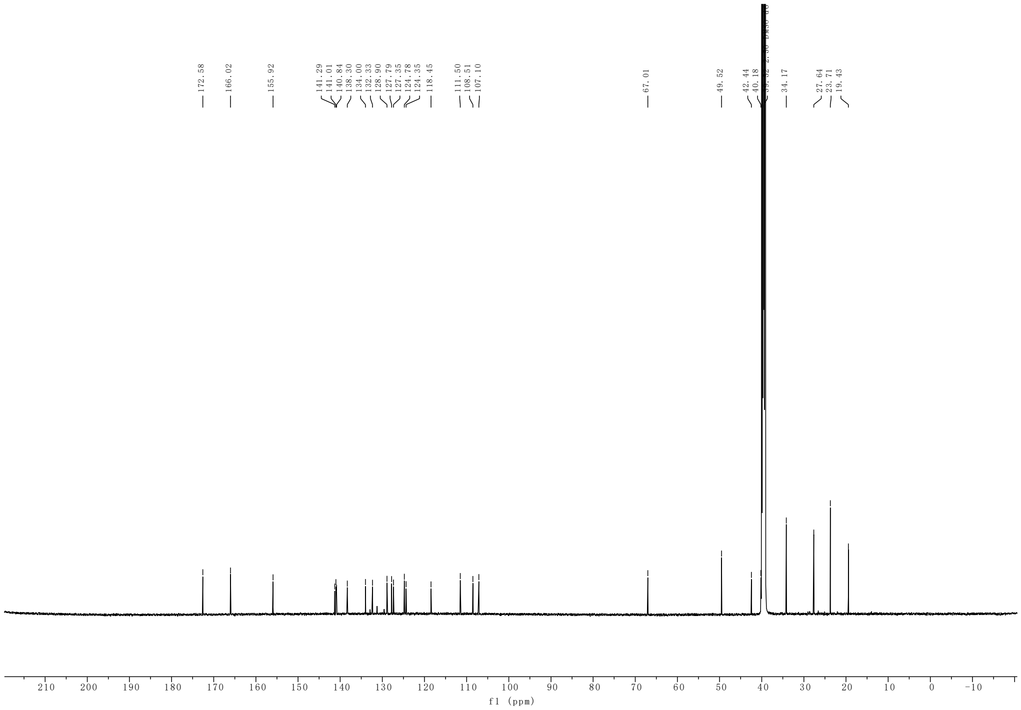

LC-MS Spectra (UV250) of compound **27**

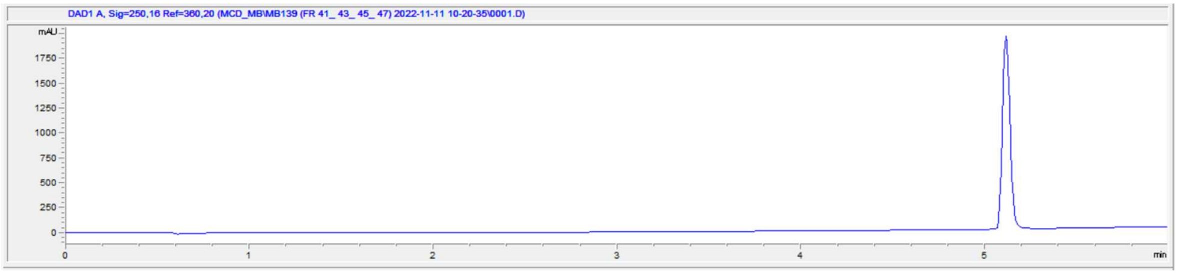

^{1}H NMR (600 MHz, DMSO-d_6) of compound **28**

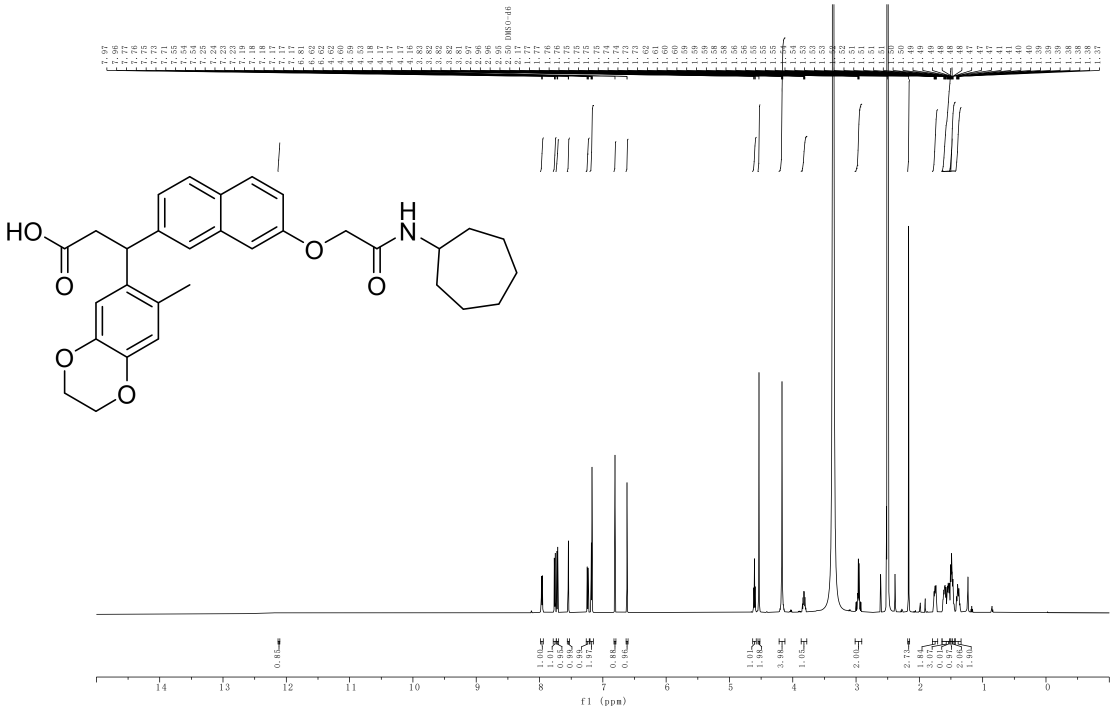

^{13}C NMR (151 MHz, DMSO-d_6) of compound **28**

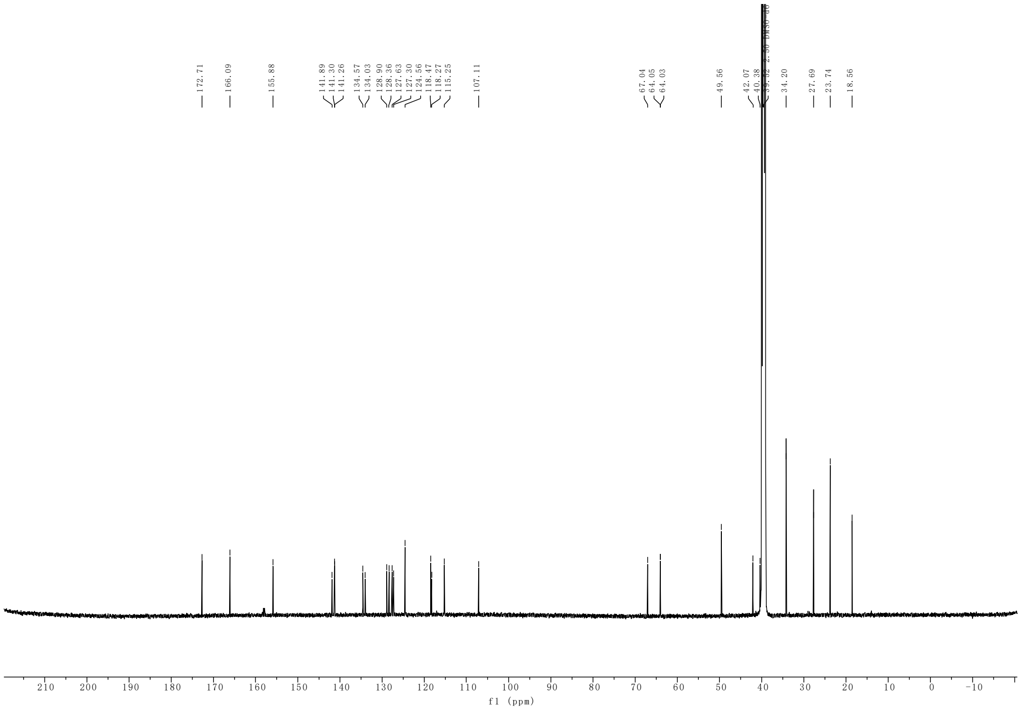

LC-MS Spectra (UV250) of compound **28**

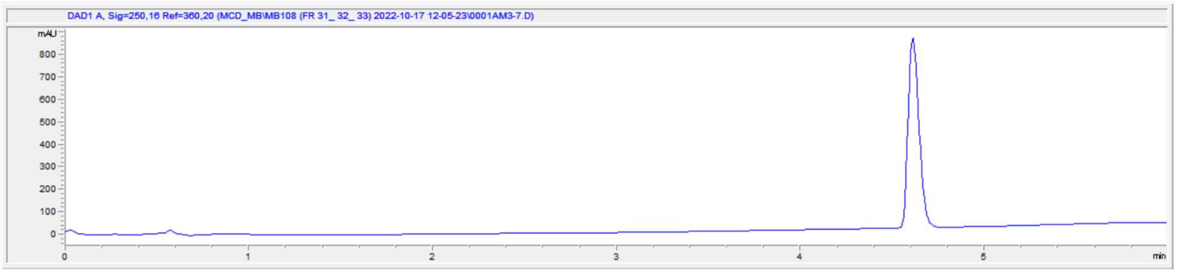

^{1}H NMR (600 MHz, DMSO-d_6) of compound **29**

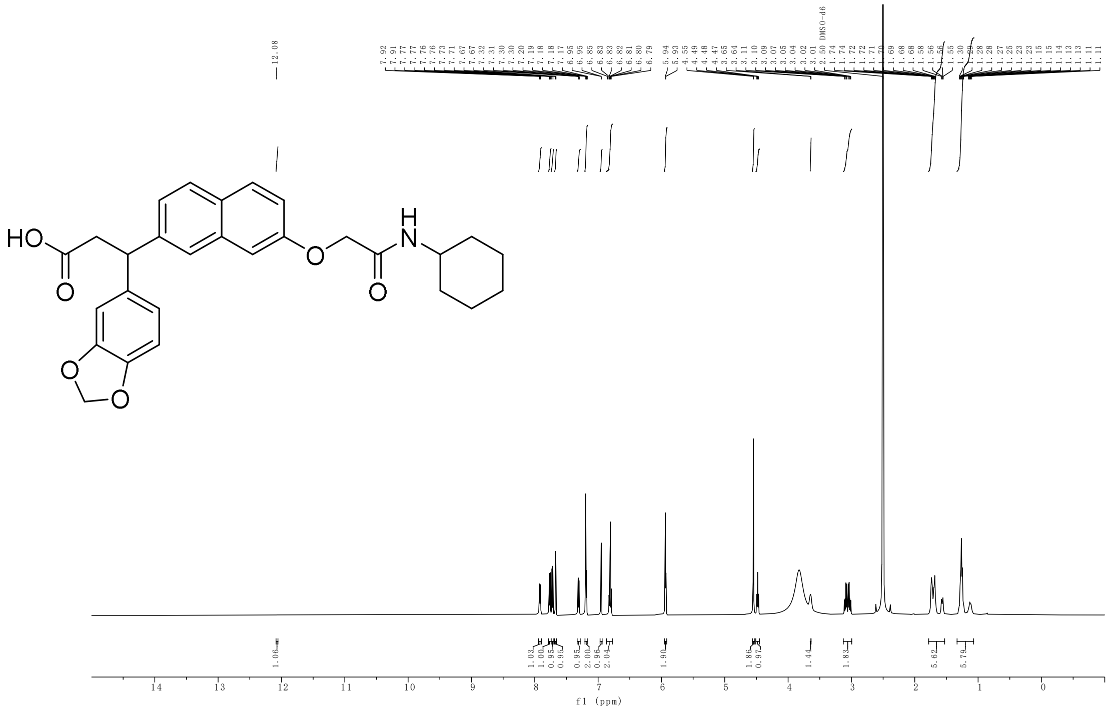

^{13}C NMR (151 MHz, DMSO-d_6) of compound **29**

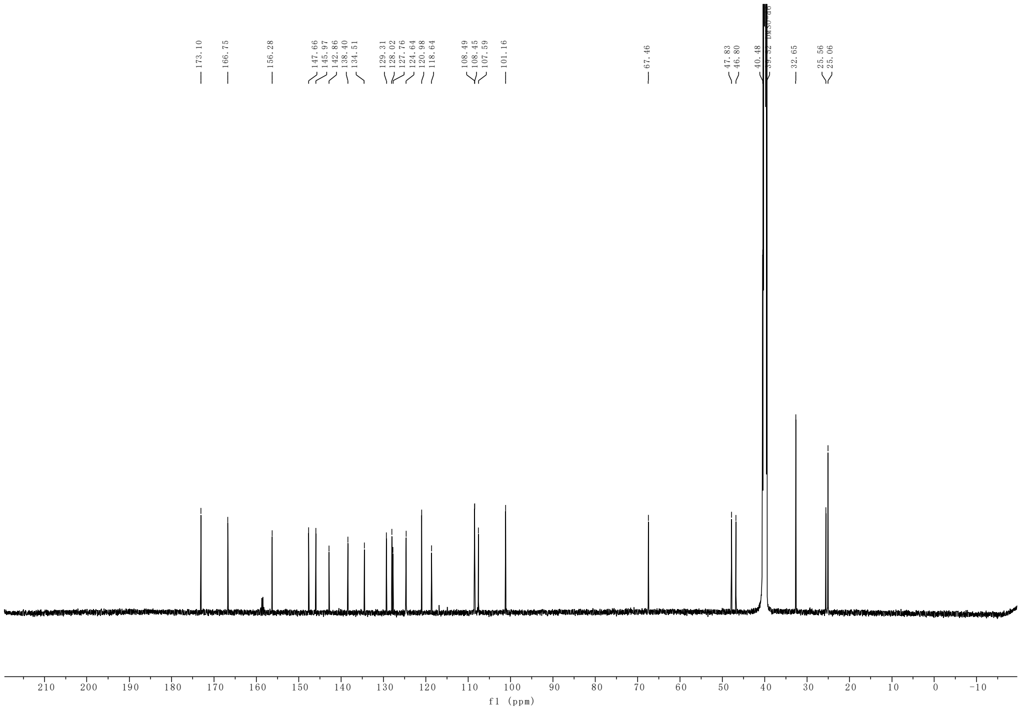

LC-MS Spectra (UV250) of compound **29**

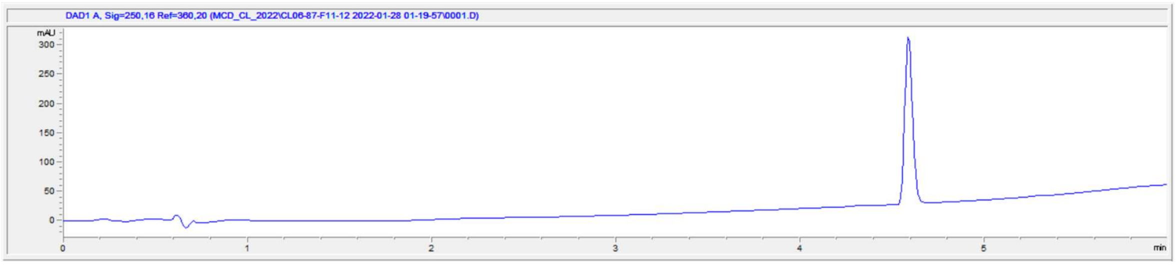

^{1}H NMR (600 MHz, DMSO-d_6) of compound **30**

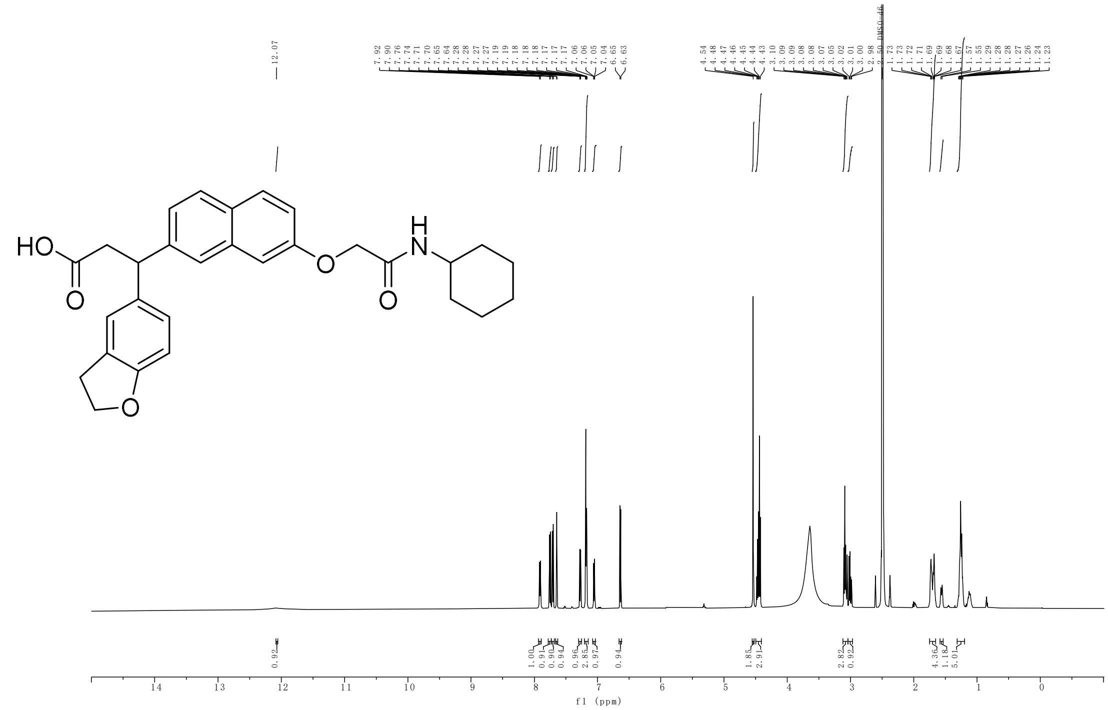

^{13}C NMR (151 MHz, DMSO-d_6) of compound **30**

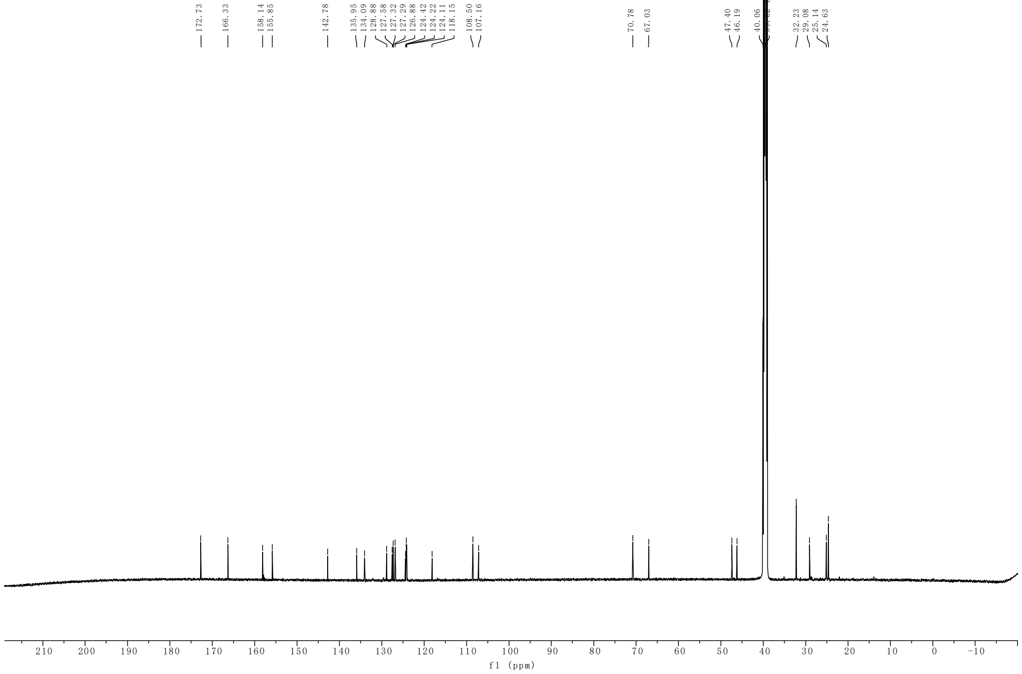

LC-MS Spectra (UV250) of compound **30**

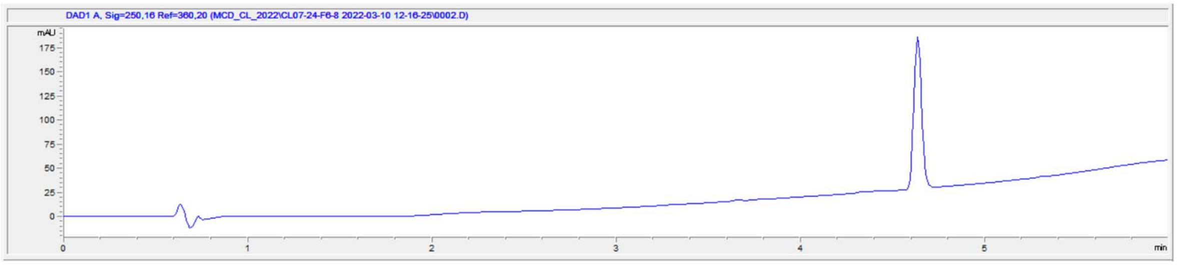

^{1}H NMR (400 MHz, DMSO-d_6) of compound **31**

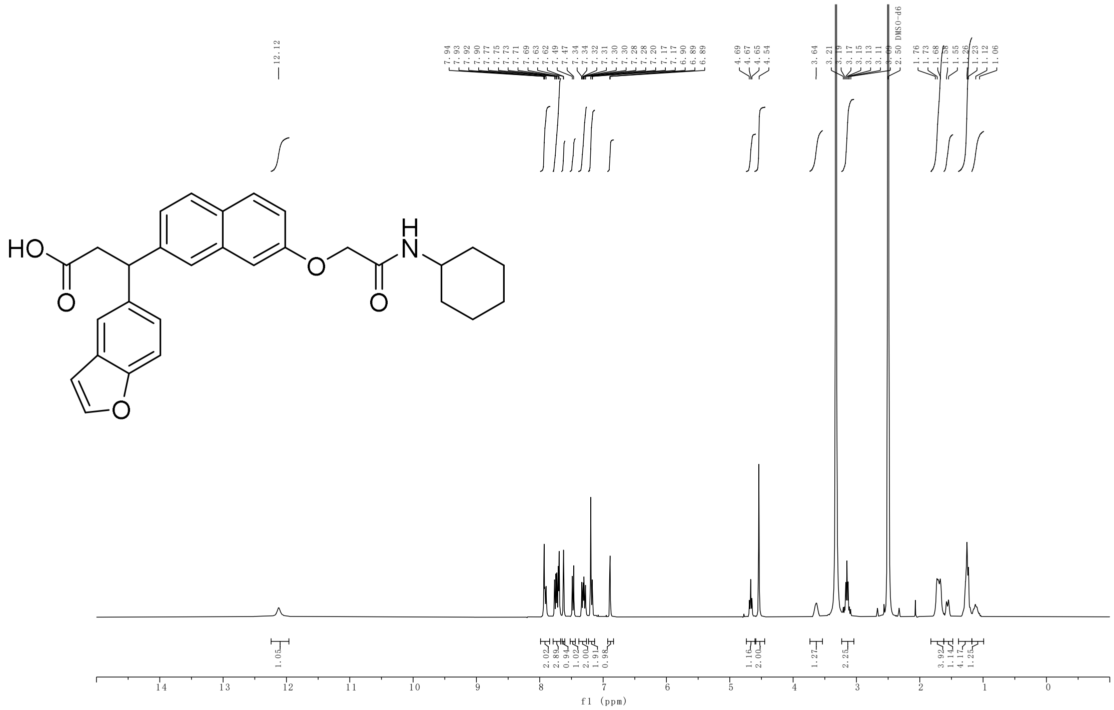

^{13}C NMR (101 MHz, DMSO-d_6) of compound **31**

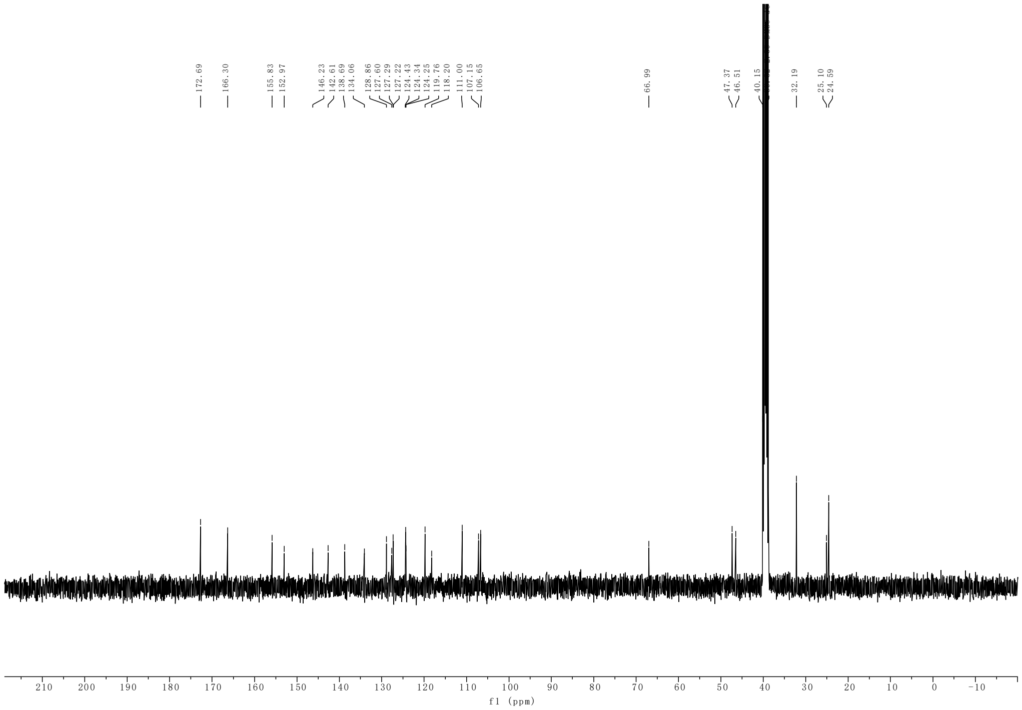

LC-MS Spectra (UV250) of compound **31**

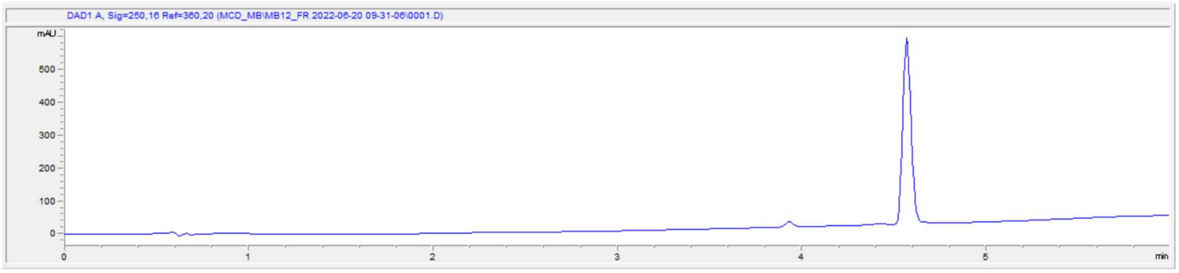

^{1}H NMR (600 MHz, DMSO-d_6) of compound **32**

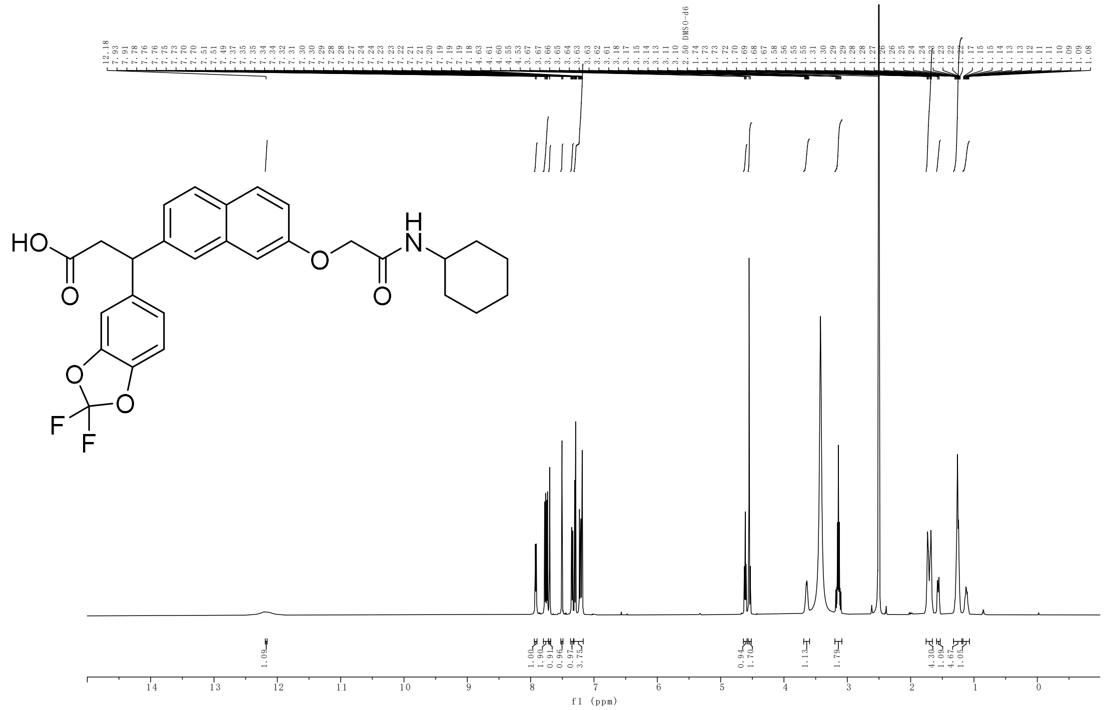

^{13}C NMR (151 MHz, DMSO-d_6) of compound **32**

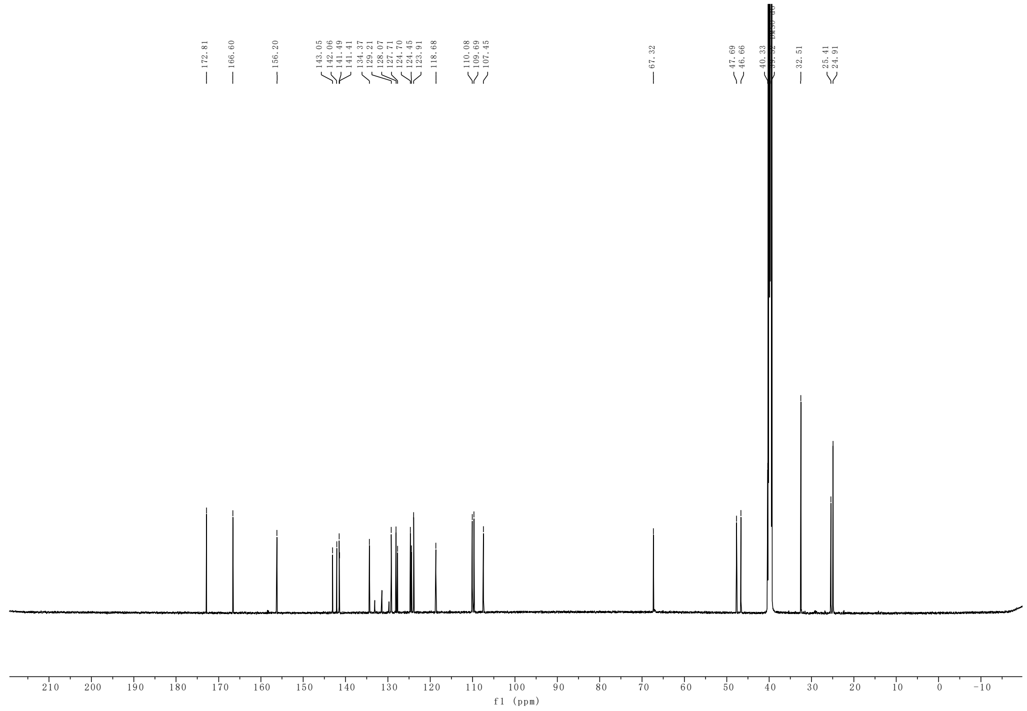

LC-MS Spectra (UV250) of compound **32**

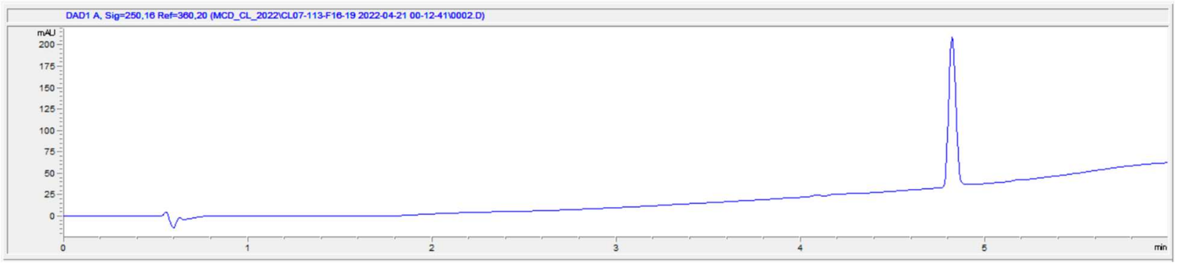

^{1}H NMR (600 MHz, DMSO-d_6) of compound **33**

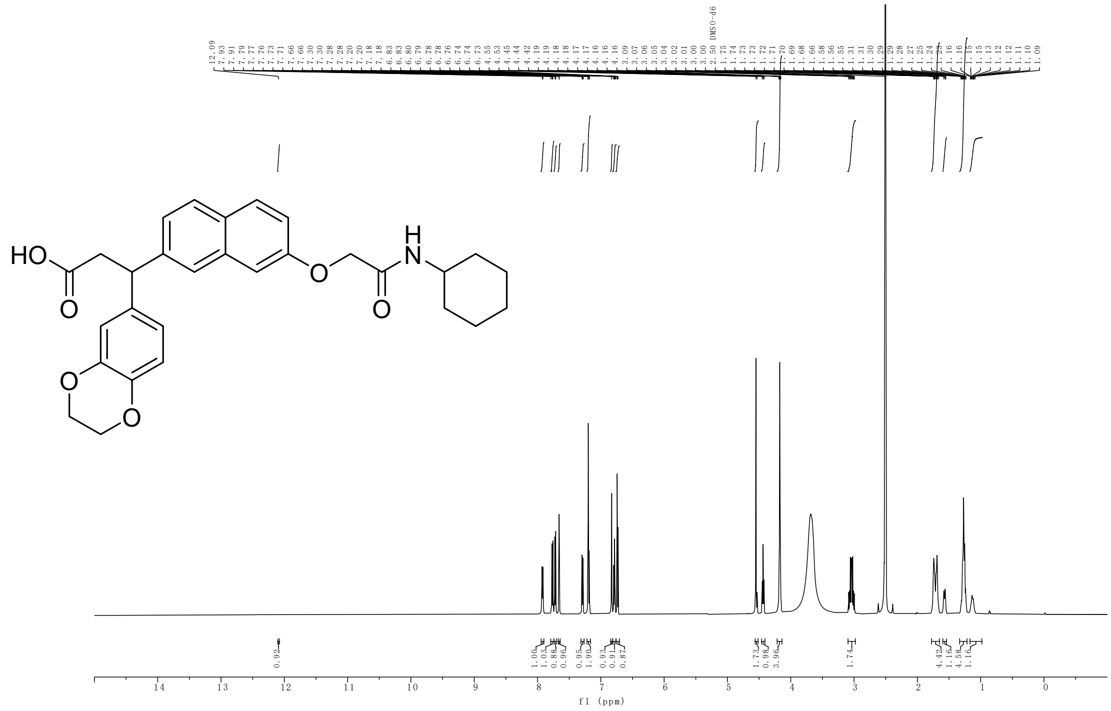

^{13}C NMR (151 MHz, DMSO-d_6) of compound **33**

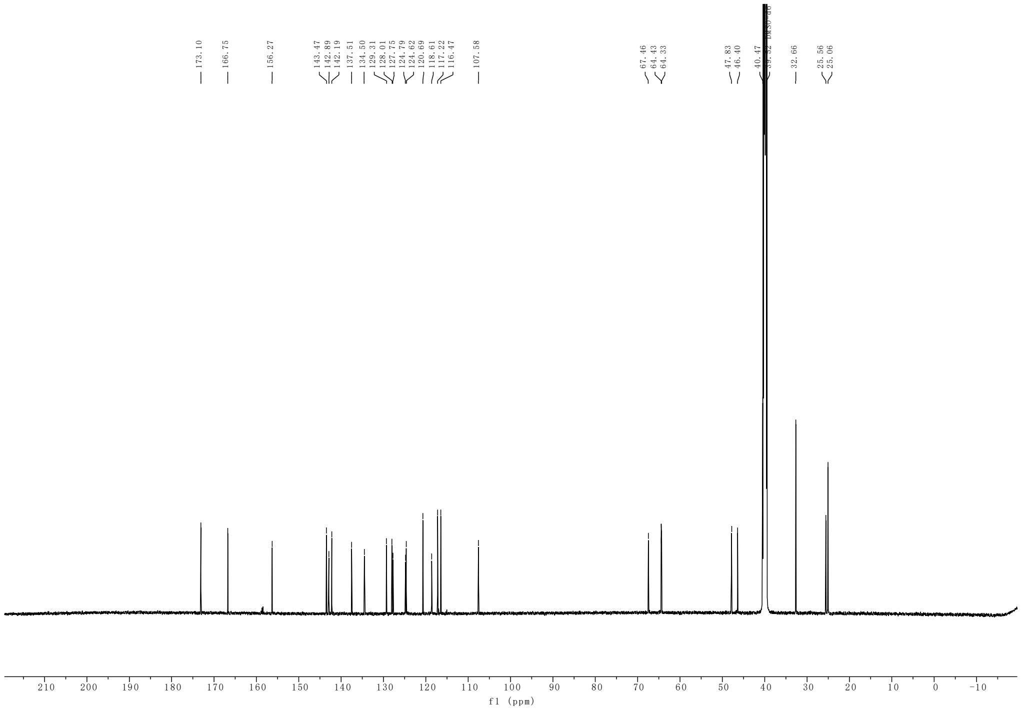

LC-MS Spectra (UV250) of compound **33**

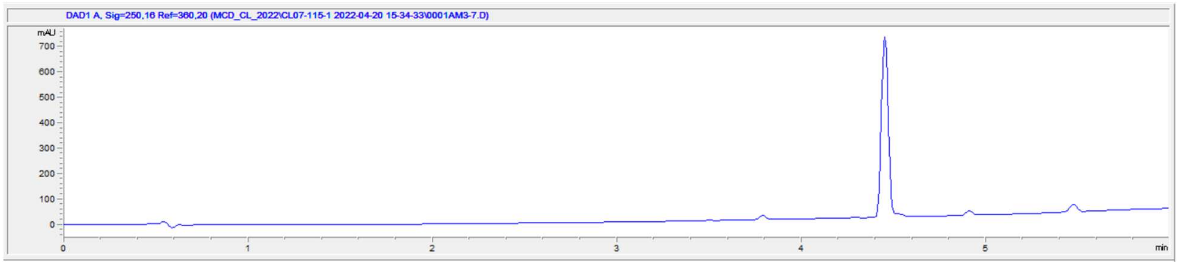

^{1}H NMR (600 MHz, DMSO-d_6) of compound **34**

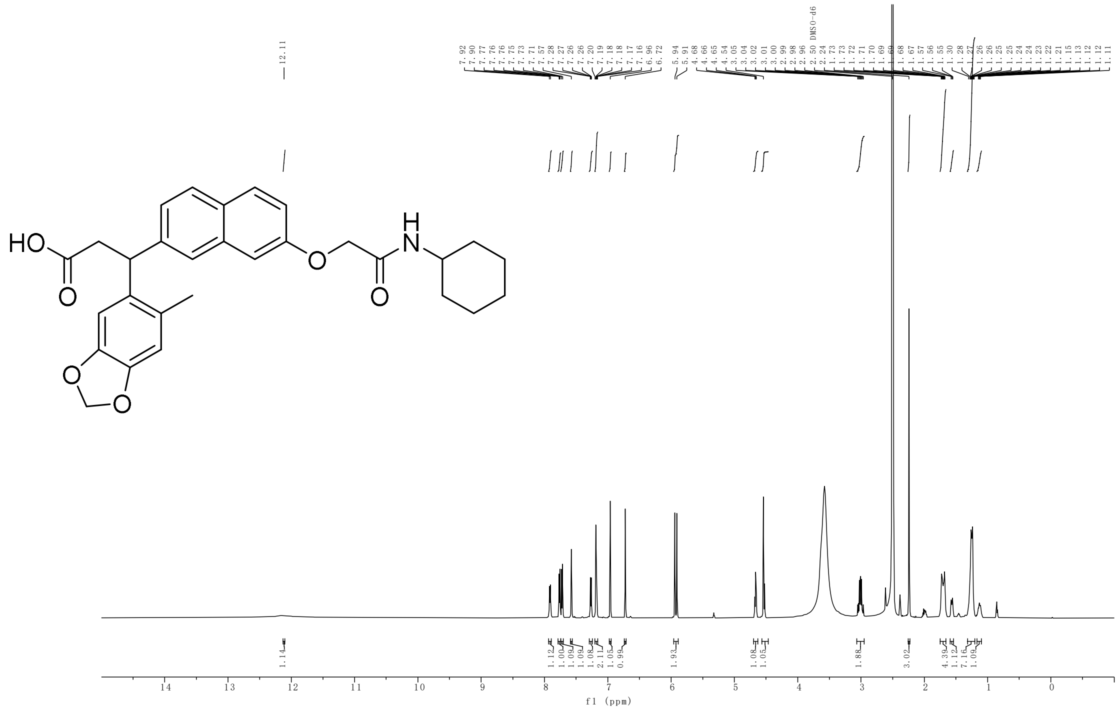

^{13}C NMR (151 MHz, DMSO-d_6) of compound **34**

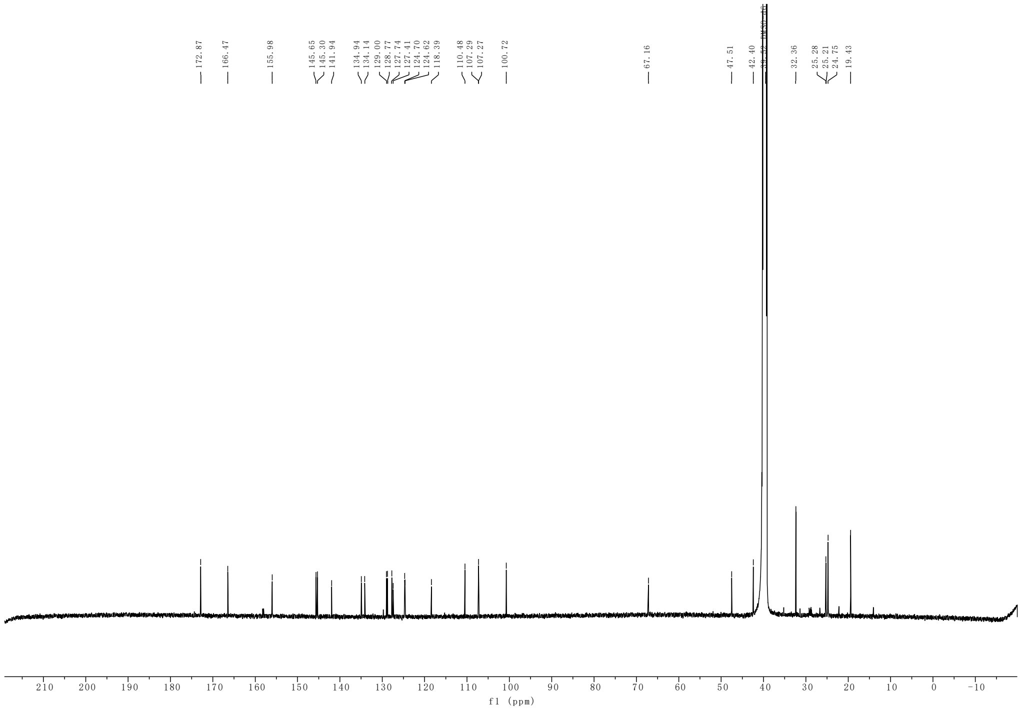

LC-MS Spectra (UV250) of compound **34**

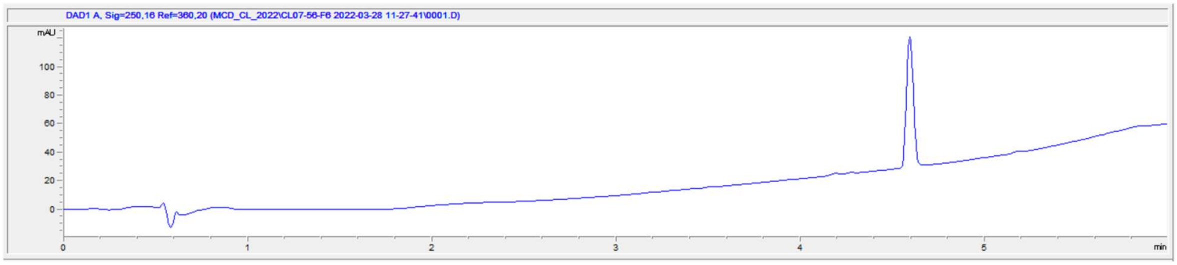

^1H NMR (600 MHz, DMSO-d_6) of compound **35**

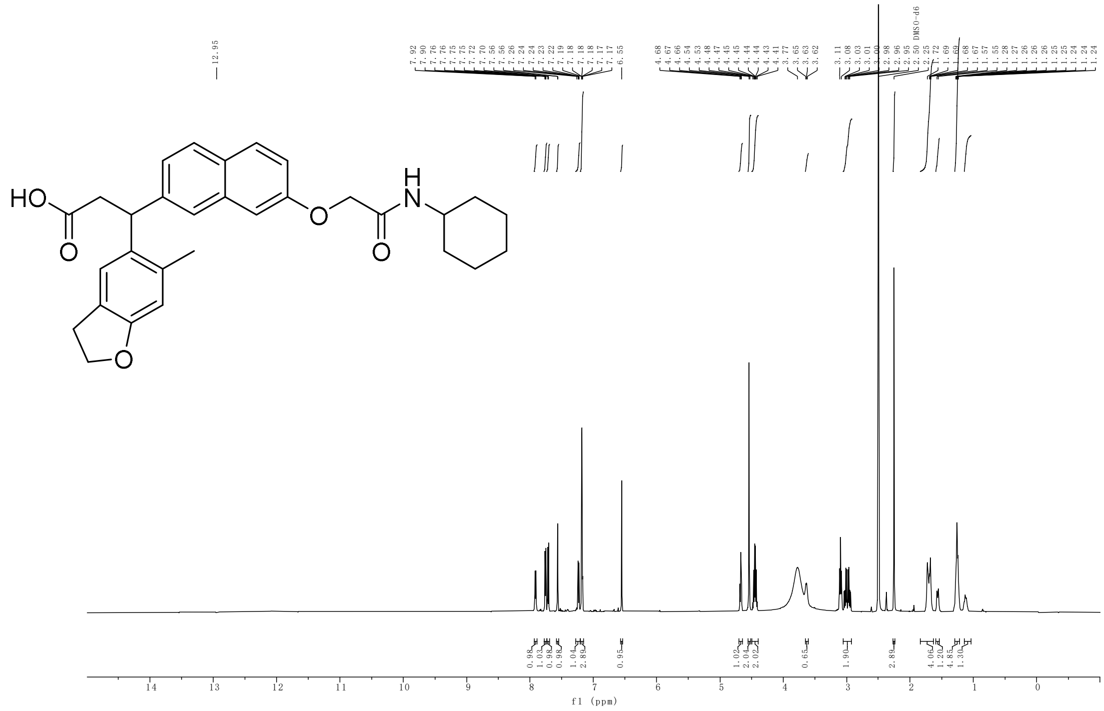

^{13}C NMR (151 MHz, DMSO-d_6) of compound **35**

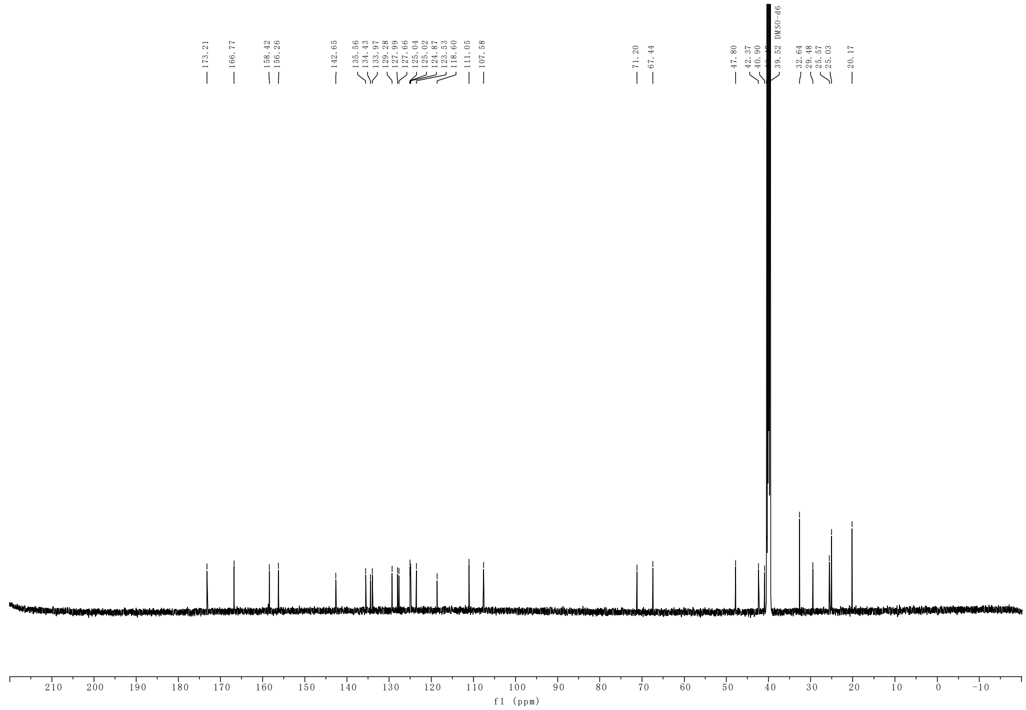

LC-MS Spectra (UV250) of compound **35**

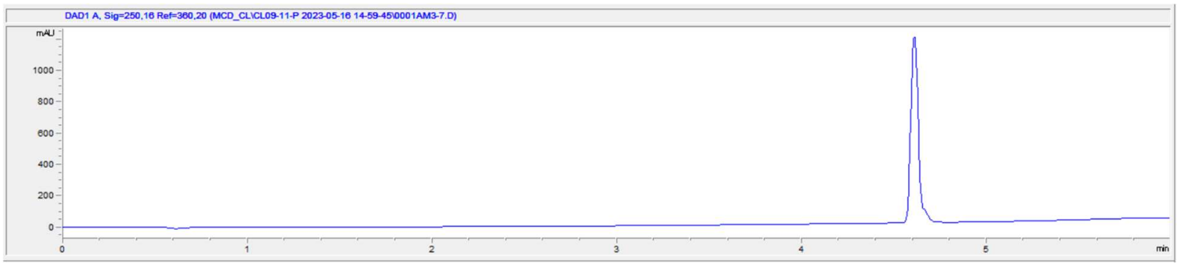

^{1}H NMR (600 MHz, DMSO-d_6) of compound **36**

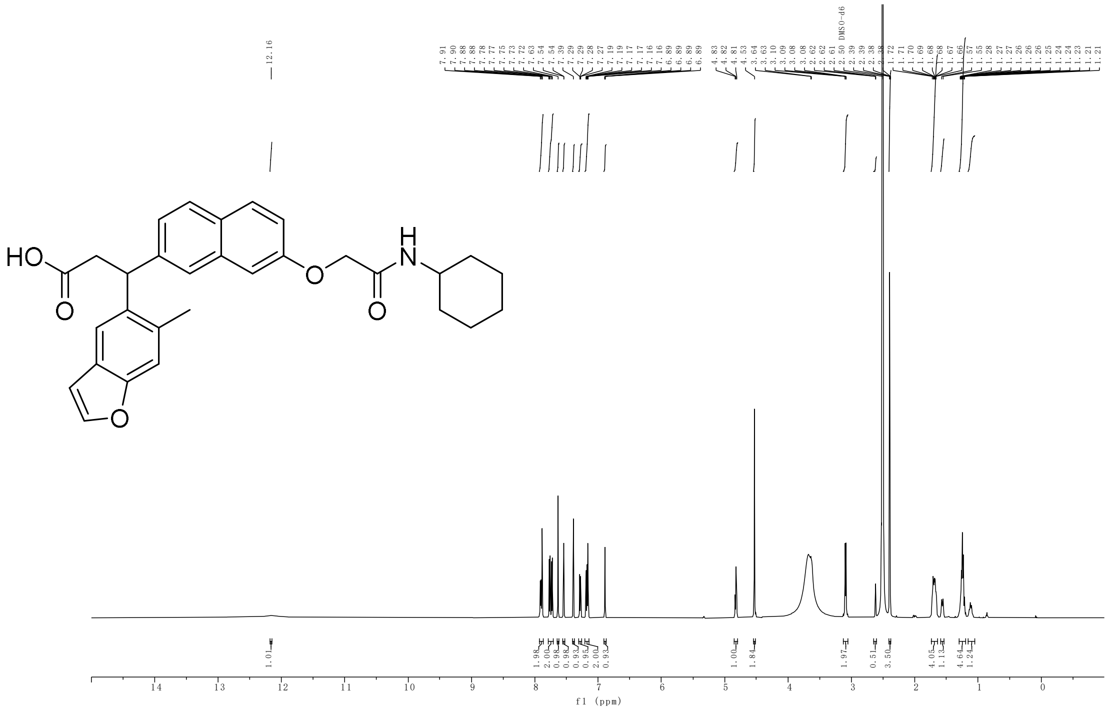

^{13}C NMR (151 MHz, DMSO-d_6) of compound **36**

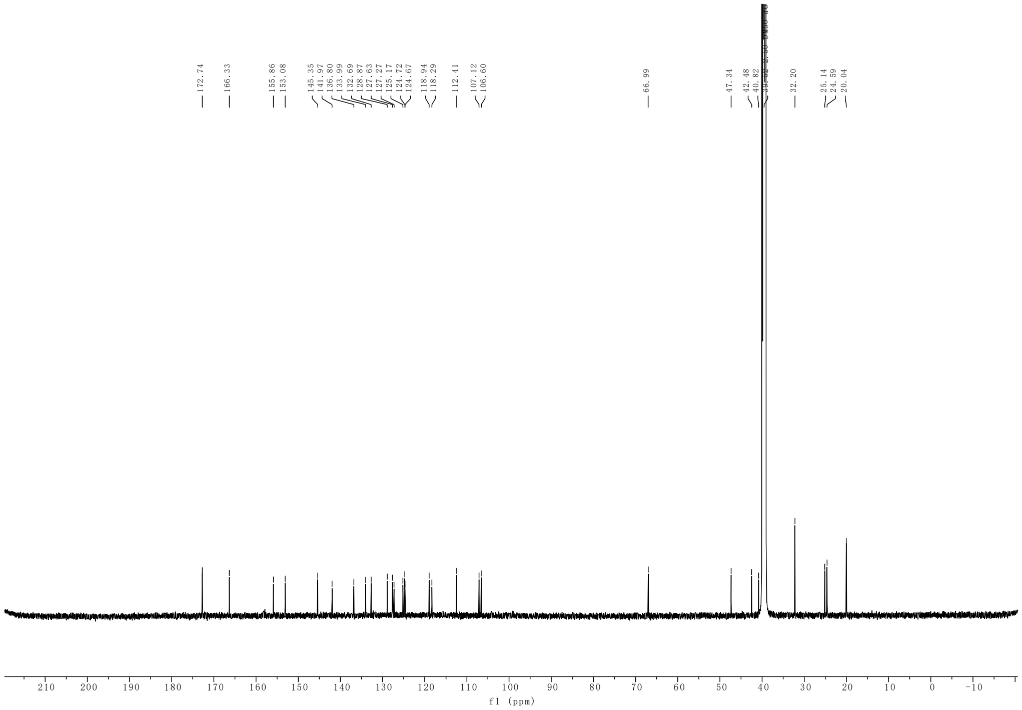

LC-MS Spectra (UV250) of compound **36**

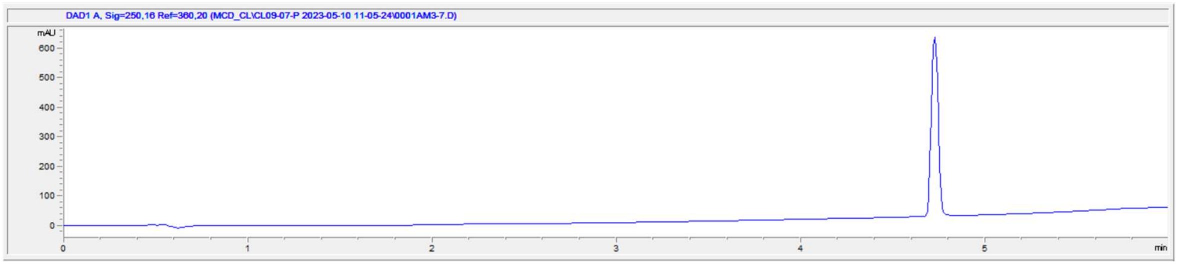

^{1}H NMR (600 MHz, DMSO-d_6) of compound **37**

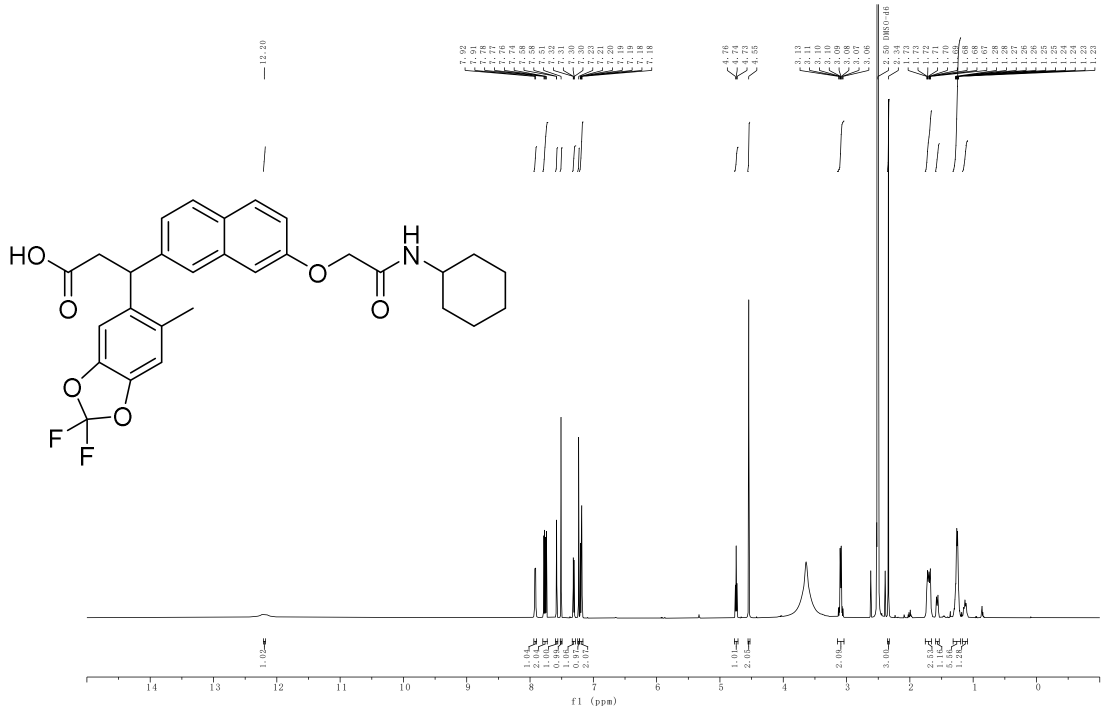

^{13}C NMR (151 MHz, DMSO-d_6) of compound **37**

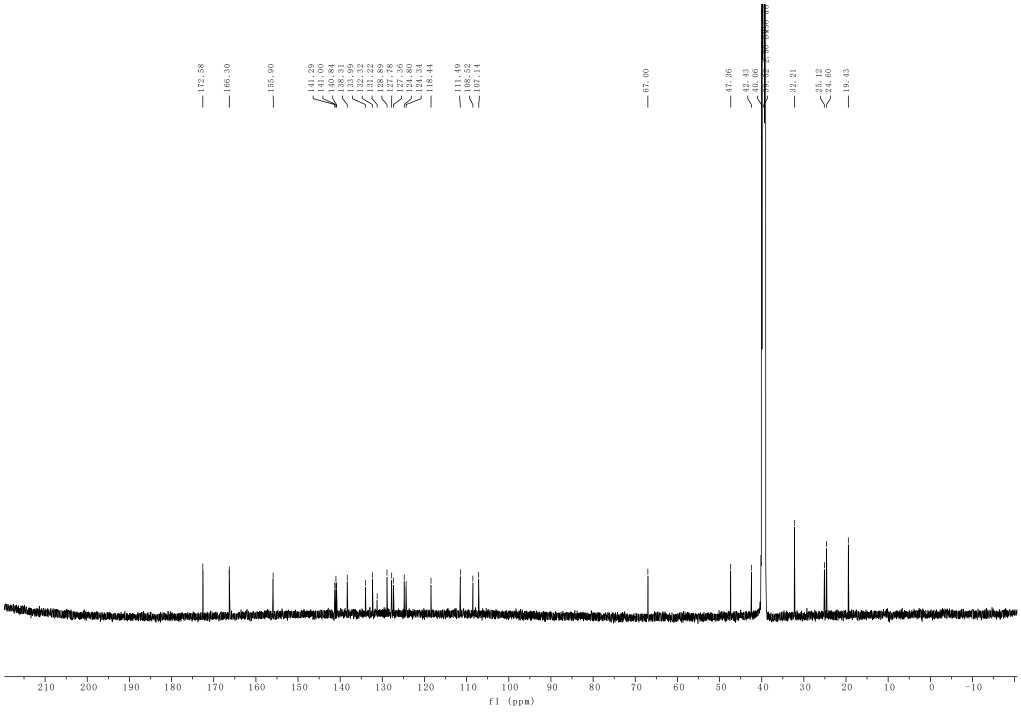

LC-MS Spectra (UV250) of compound **37**

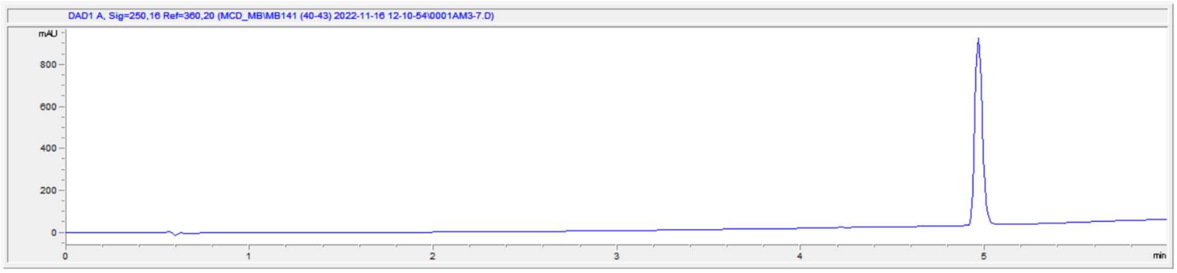

^{1}H NMR (600 MHz, DMSO-d_6) of compound **38**

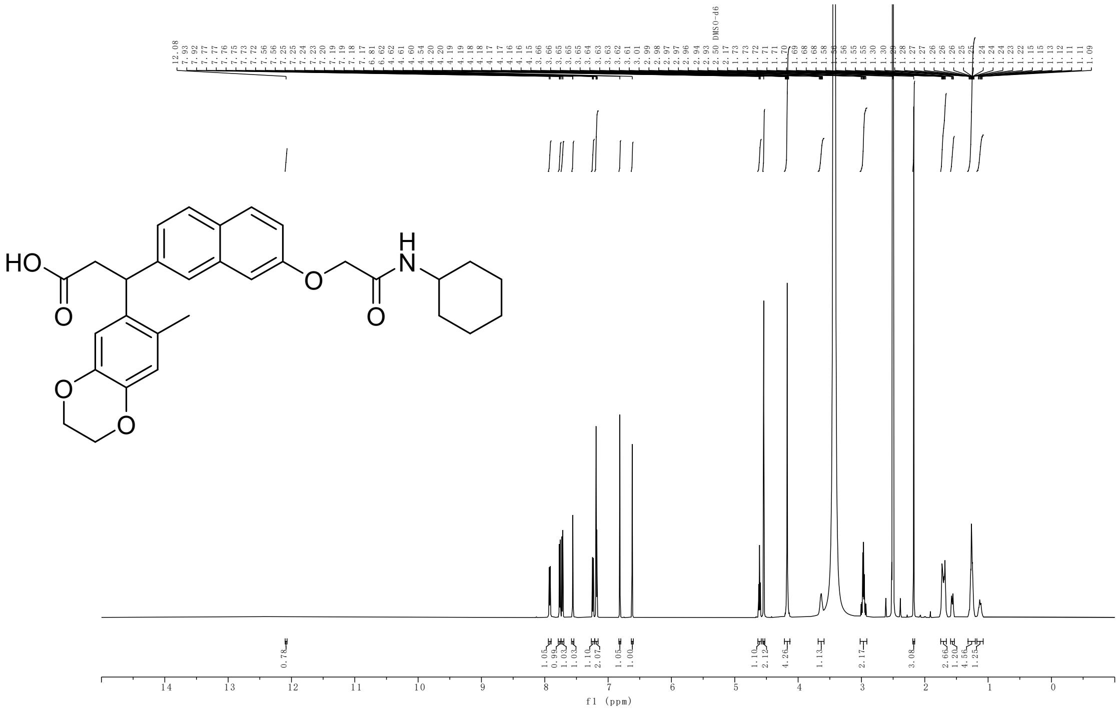

^{13}C NMR (151 MHz, DMSO-d_6) of compound **38**

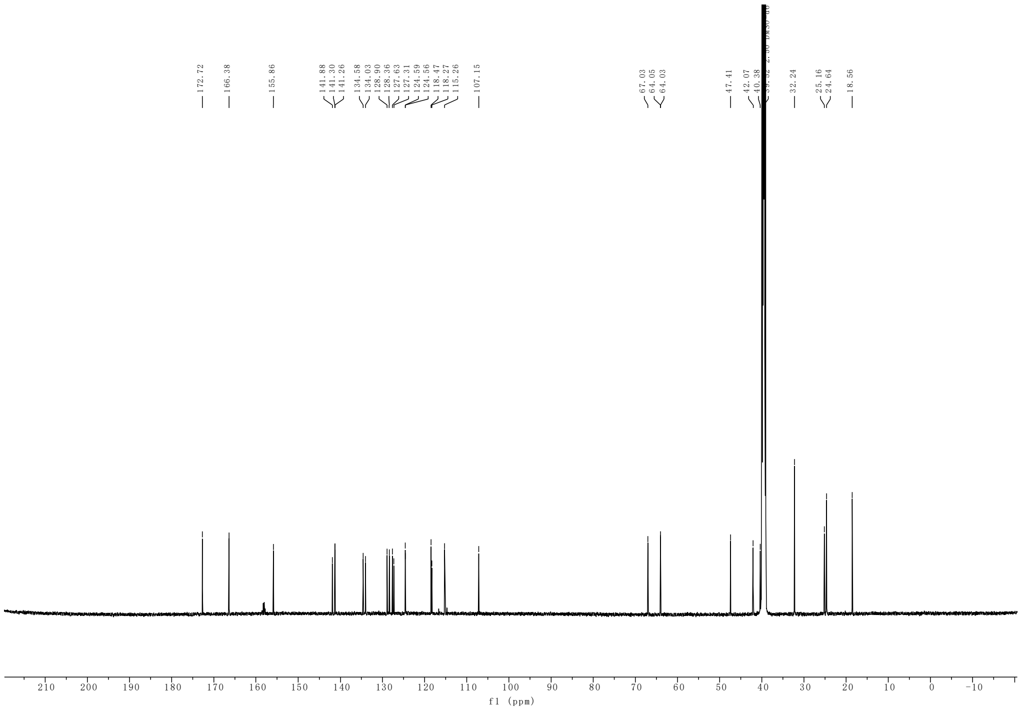

LC-MS Spectra (UV250) of compound **38**

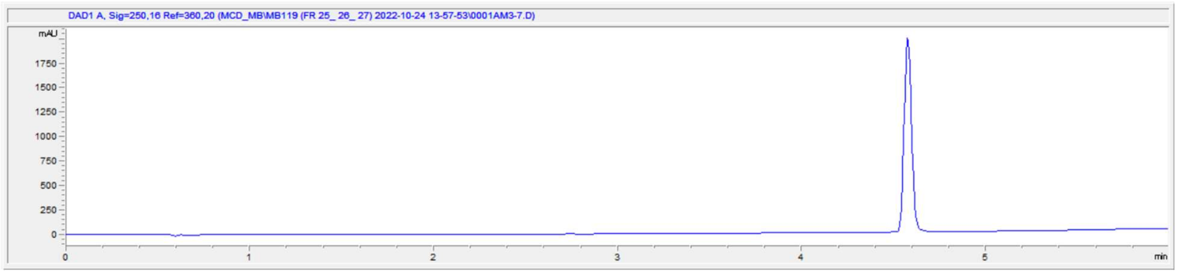

^{1}H NMR (600 MHz, DMSO-d_6) of compound **39**

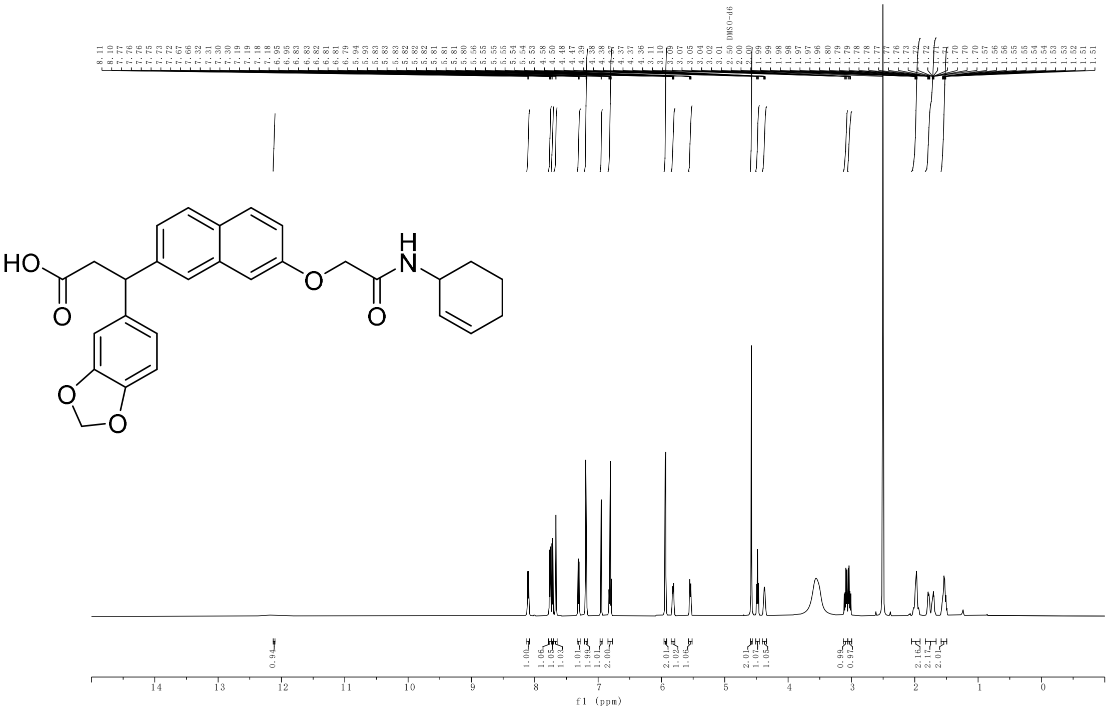

^{13}C NMR (151 MHz, DMSO-d_6) of compound **39**

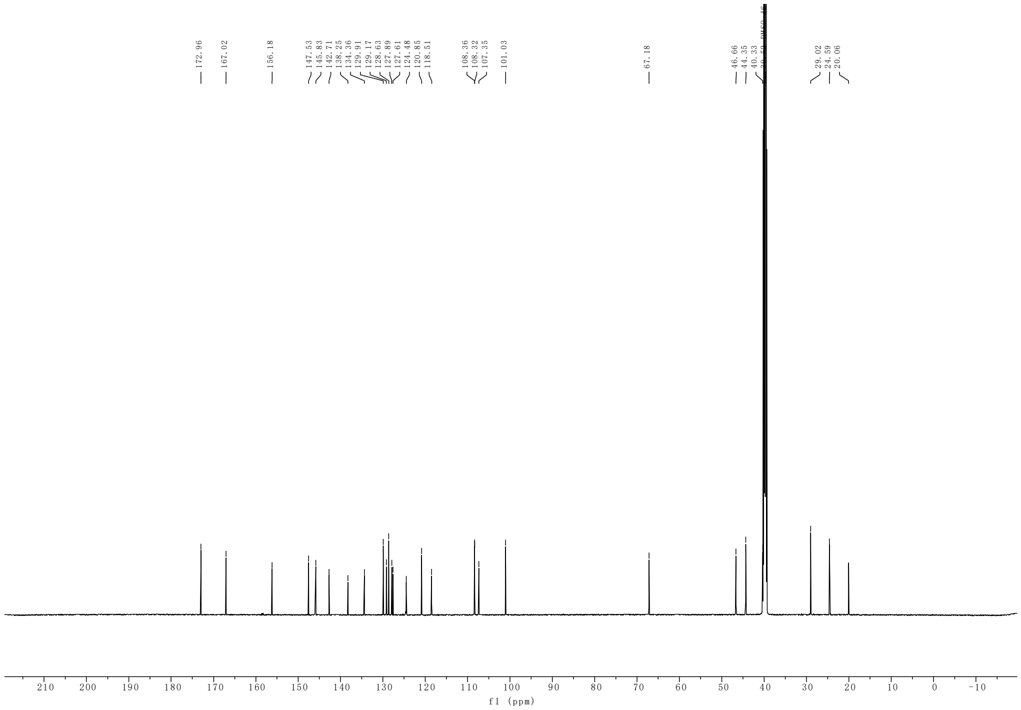

LC-MS Spectra (UV250) of compound **39**

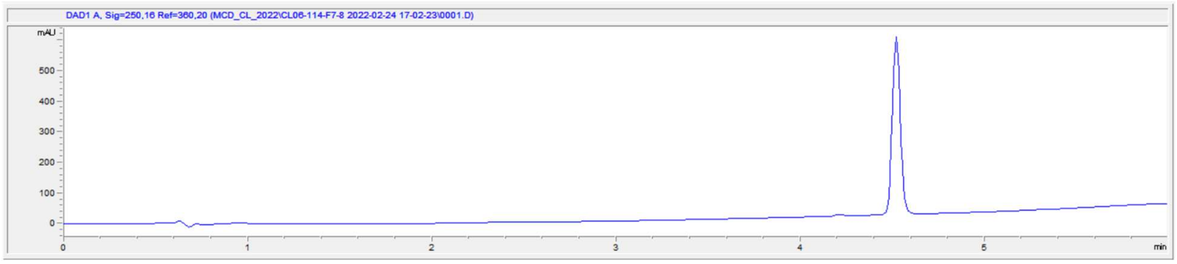

^{1}H NMR (600 MHz, DMSO-d_6) of compound **40**

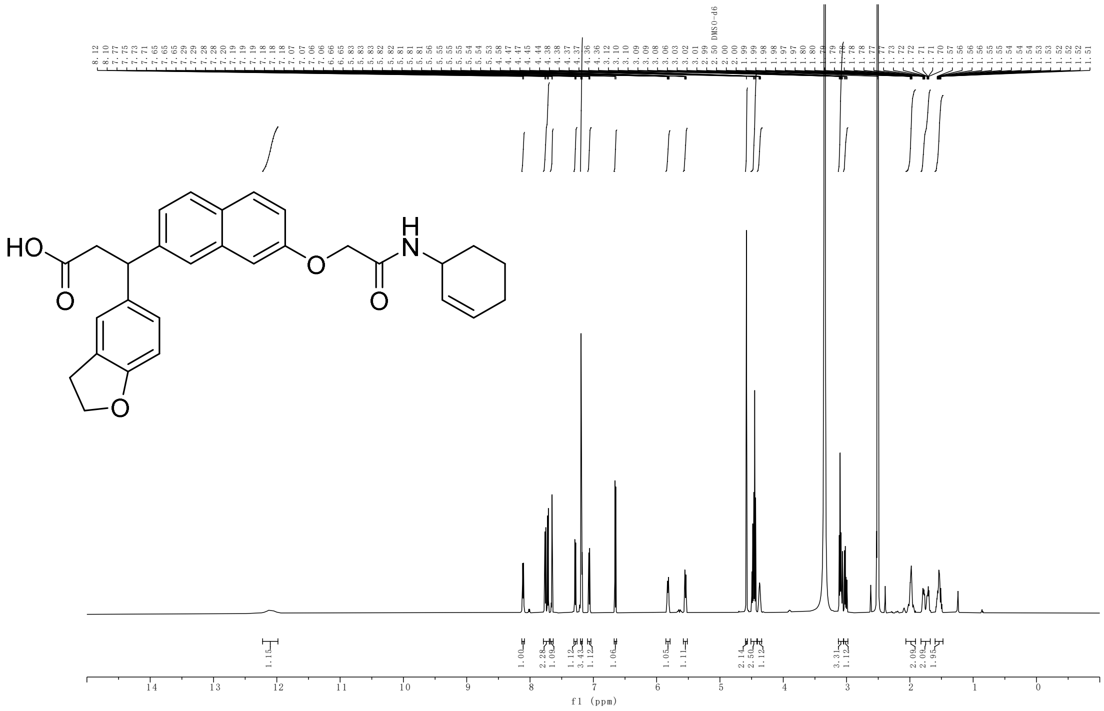

^{13}C NMR (151 MHz, DMSO-d_6) of compound **40**

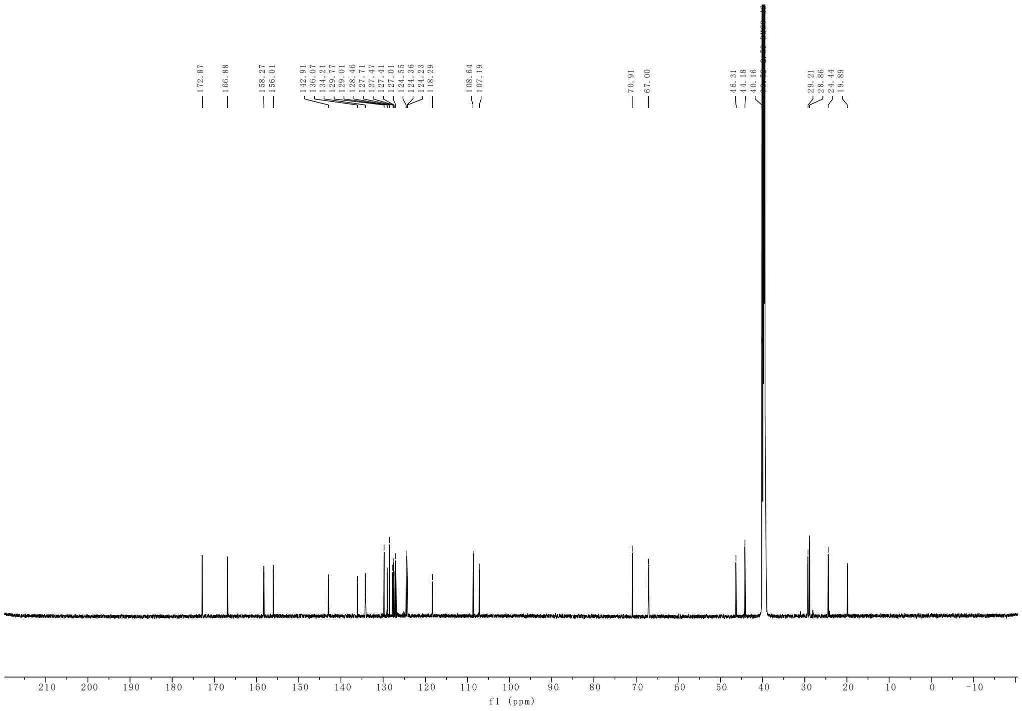

LC-MS Spectra (UV250) of compound **40**

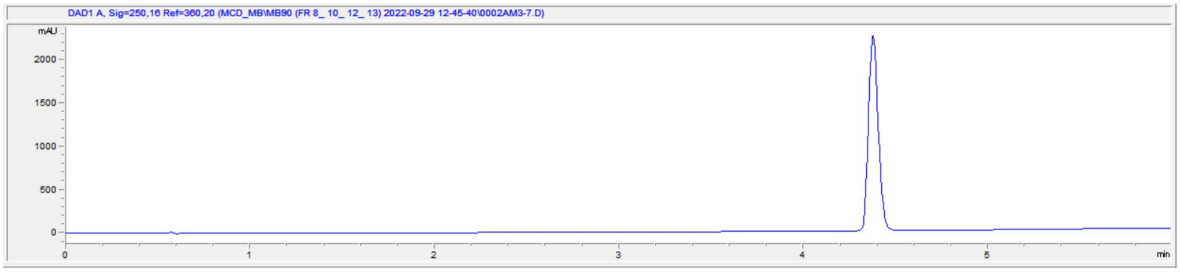

^{1}H NMR (600 MHz, DMSO-d_6) of compound **41**

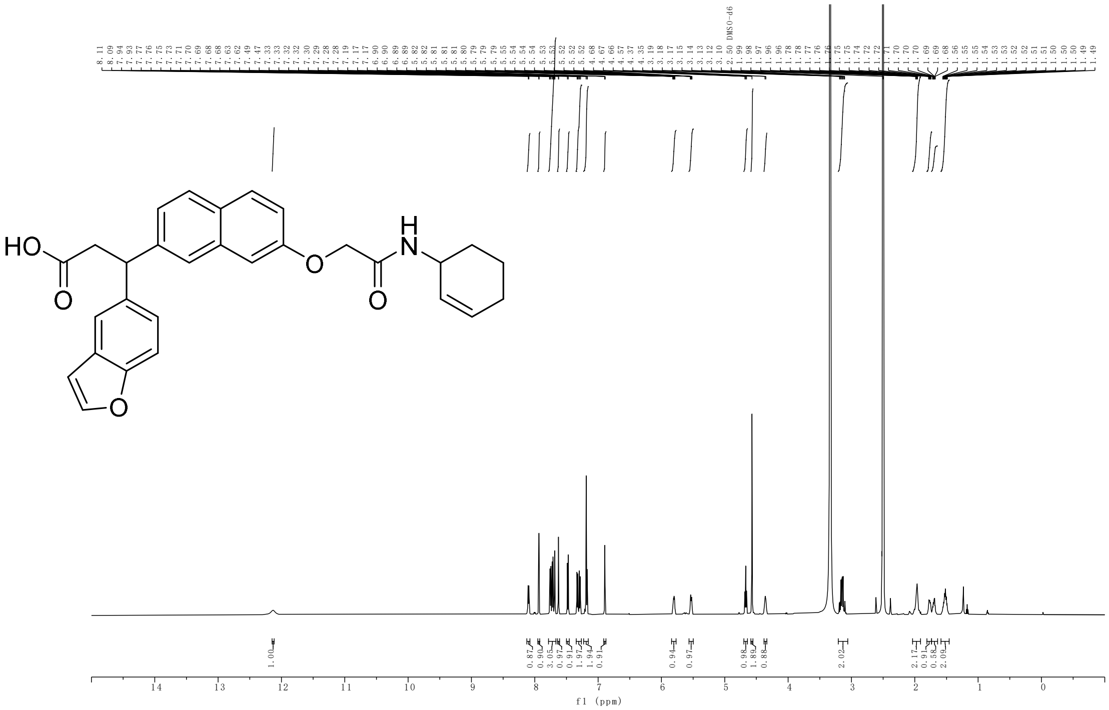

^{13}C NMR (151 MHz, DMSO-d_6) of compound **41**

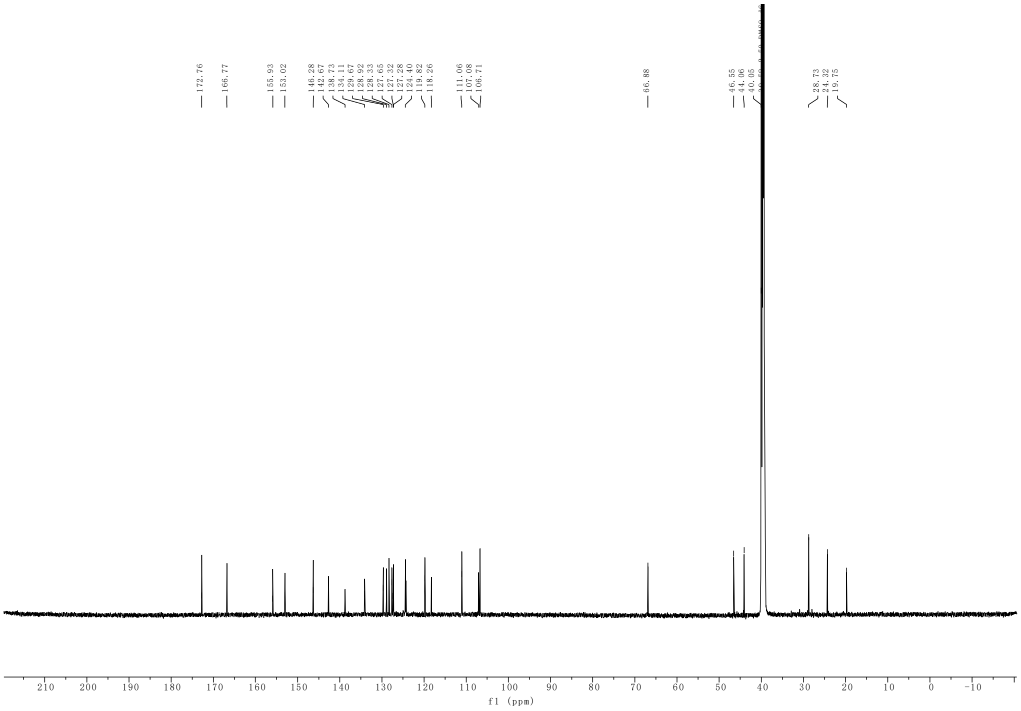

LC-MS Spectra (UV250) of compound **41**

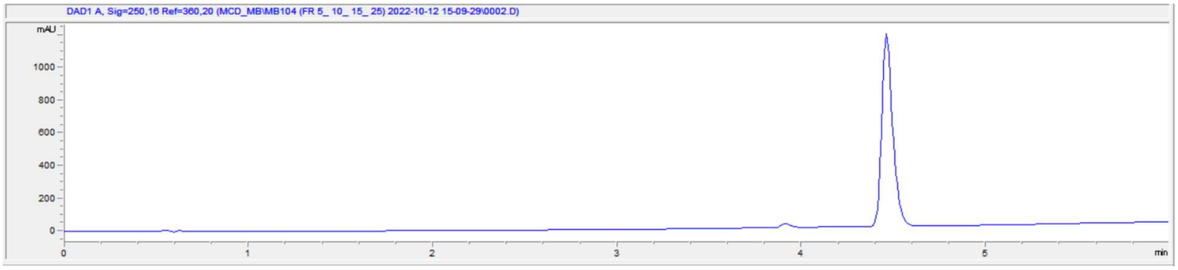

^{1}H NMR (600 MHz, DMSO-d_6) of compound **42**

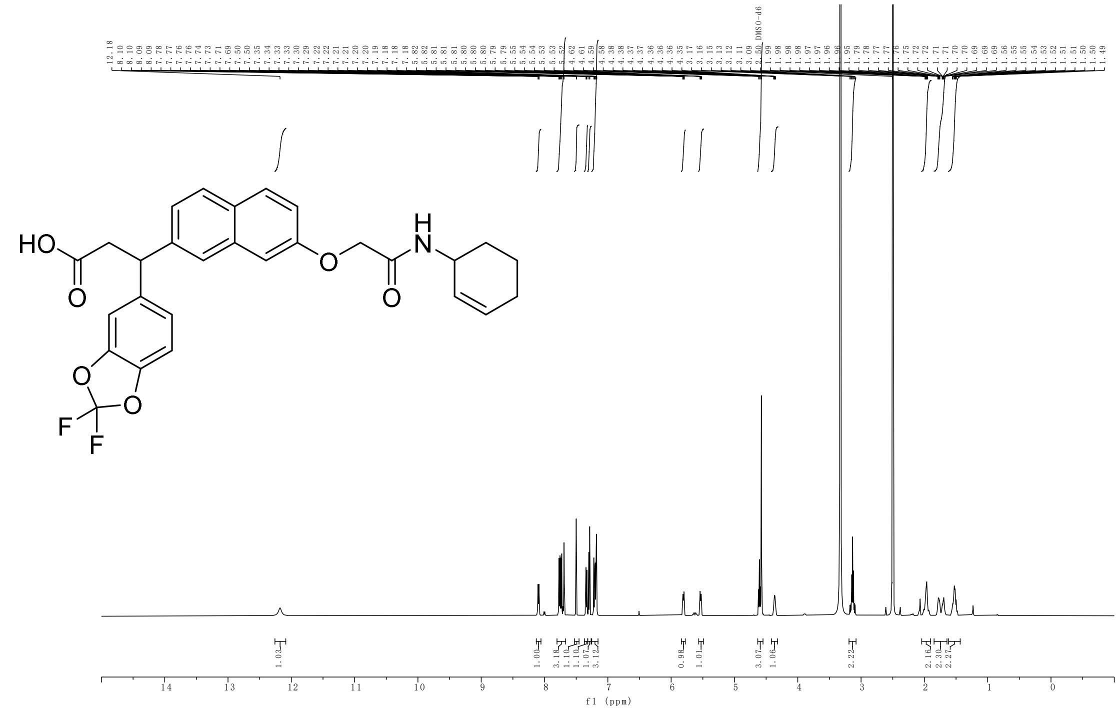

^{13}C NMR (151 MHz, DMSO-d_6) of compound **42**

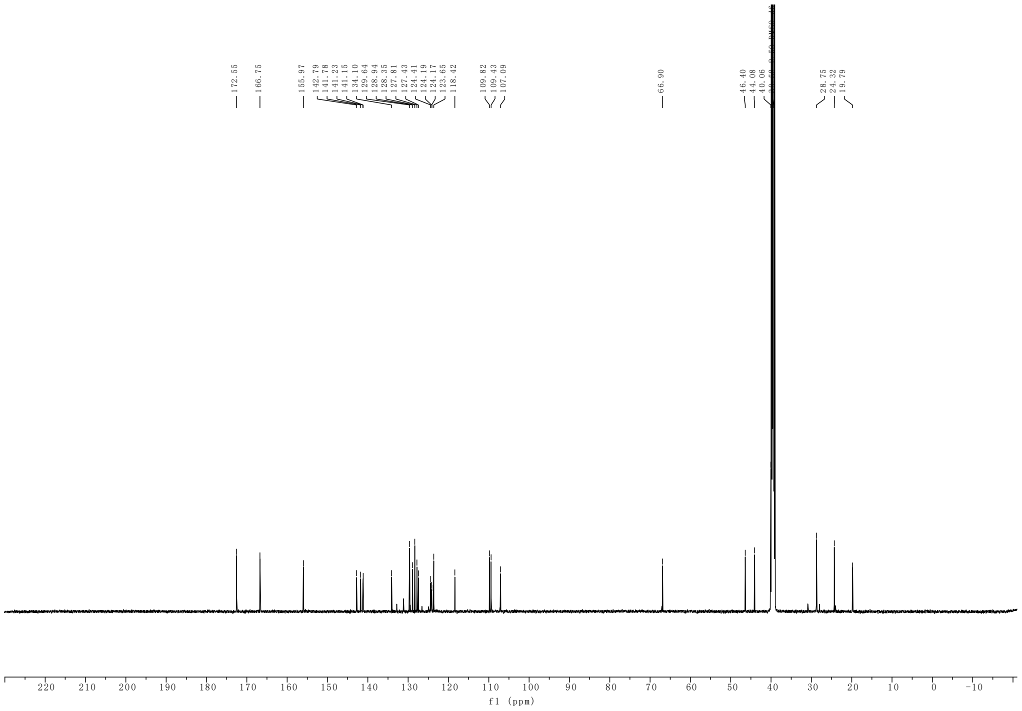

LC-MS Spectra (UV250) of compound **42**

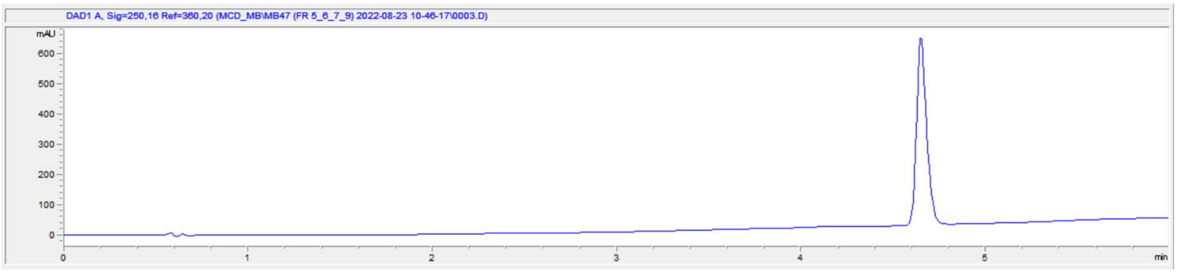

^{1}H NMR (600 MHz, DMSO-d_6) of compound **43**

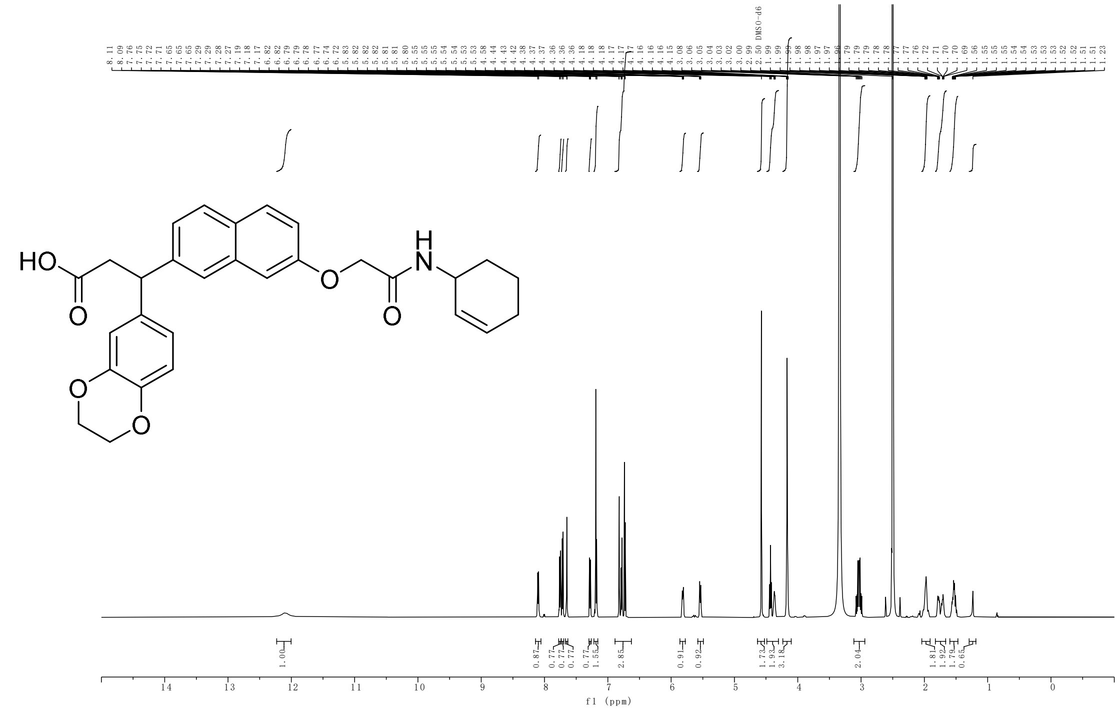

^{13}C NMR (151 MHz, DMSO-d_6) of compound **43**

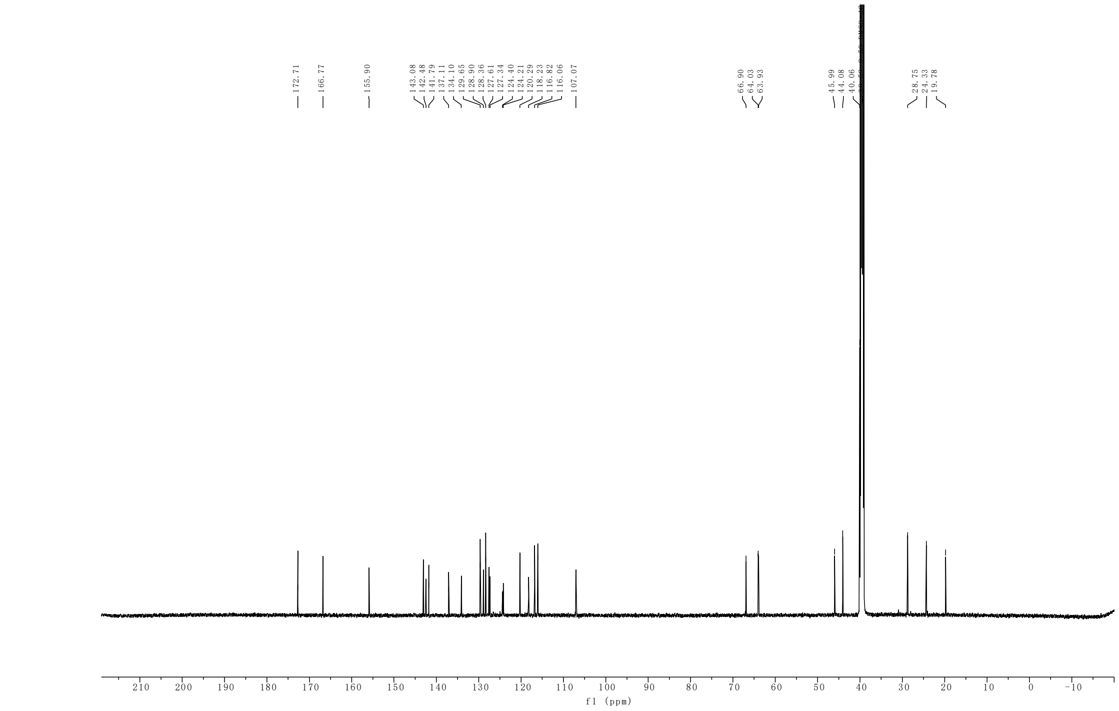

LC-MS Spectra (UV250) of compound **43**

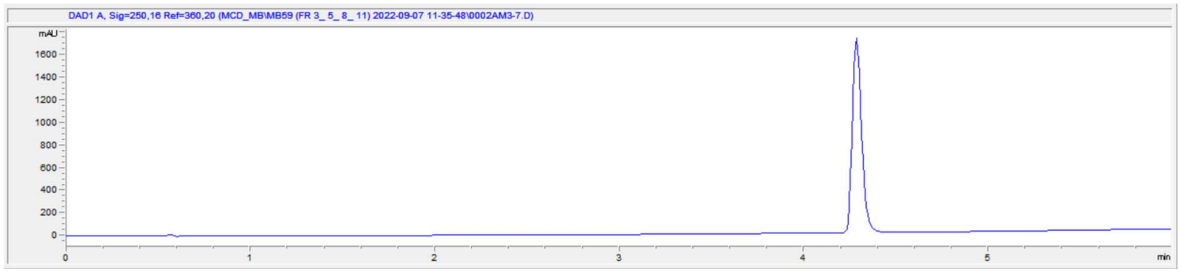

^{1}H NMR (600 MHz, DMSO-d_6) of compound **44**

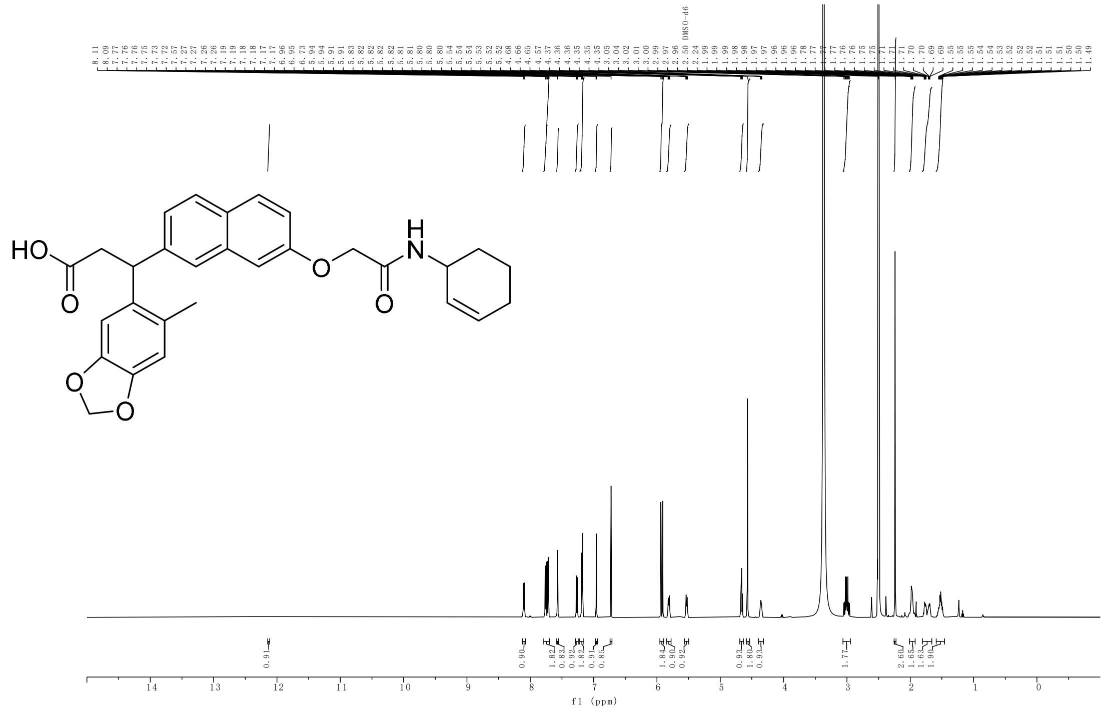

^{13}C NMR (151 MHz, DMSO-d_6) of compound **44**

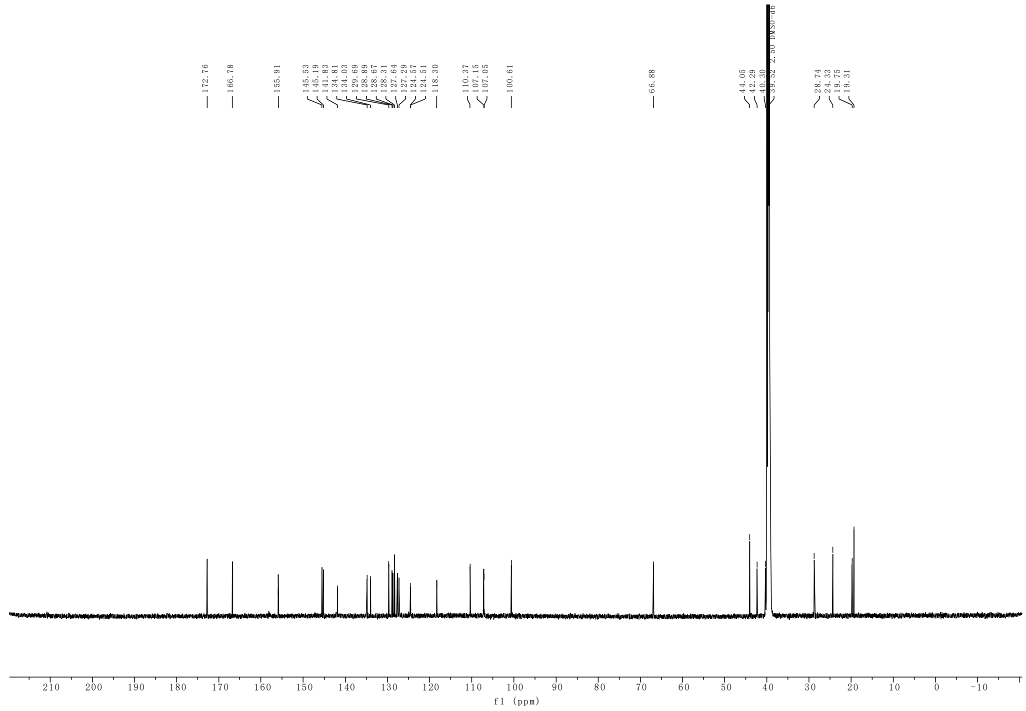

LC-MS Spectra (UV250) of compound **44**

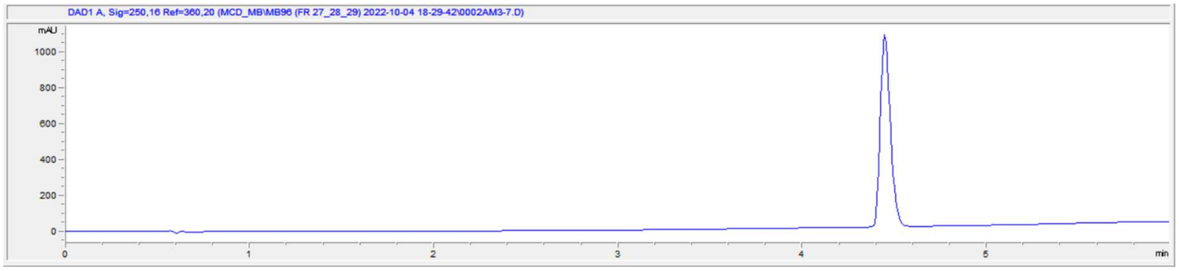

^{1}H NMR (600 MHz, DMSO-d_6) of compound **45**

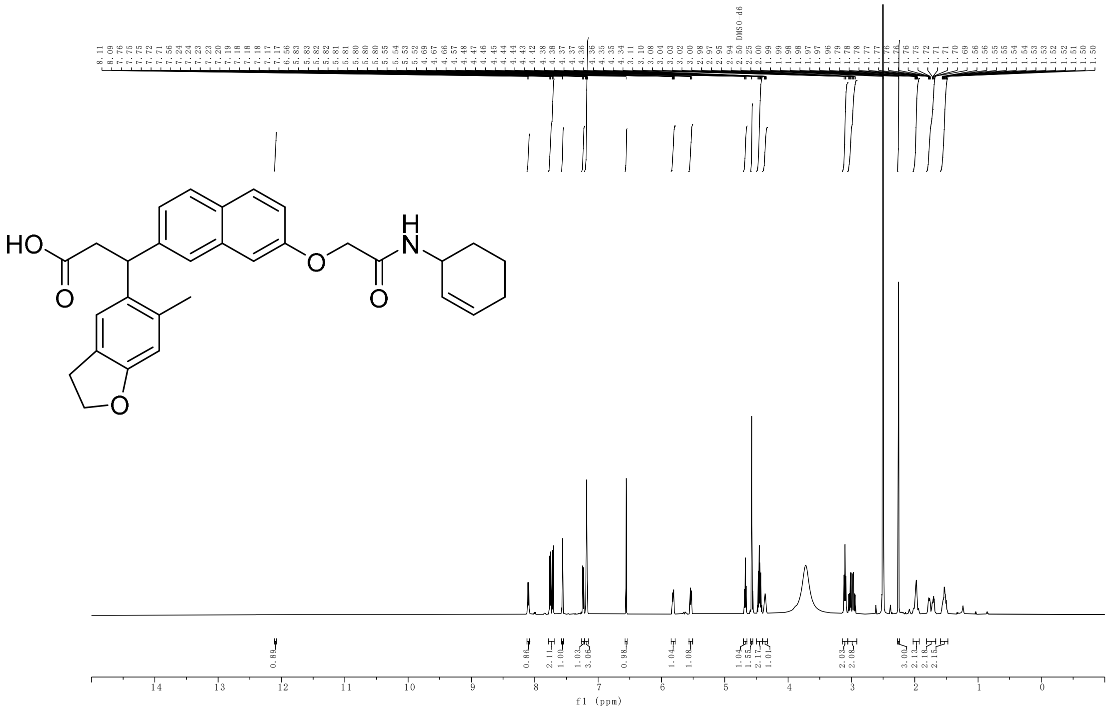

^{13}C NMR (151 MHz, DMSO-d_6) of compound **45**

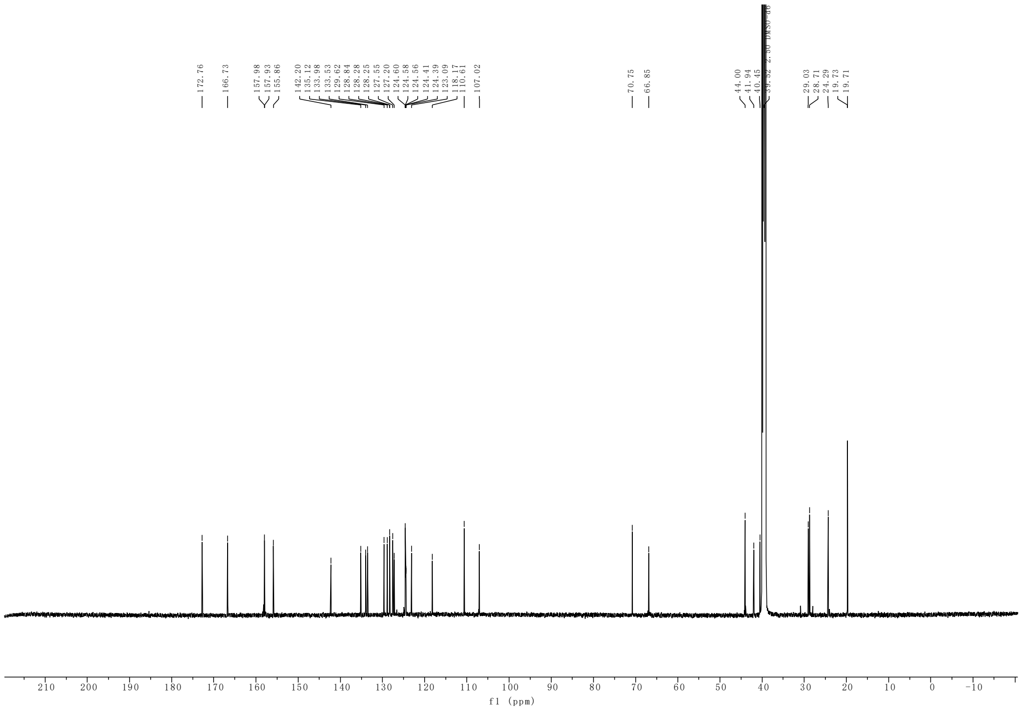

LC-MS Spectra (UV250) of compound **45**

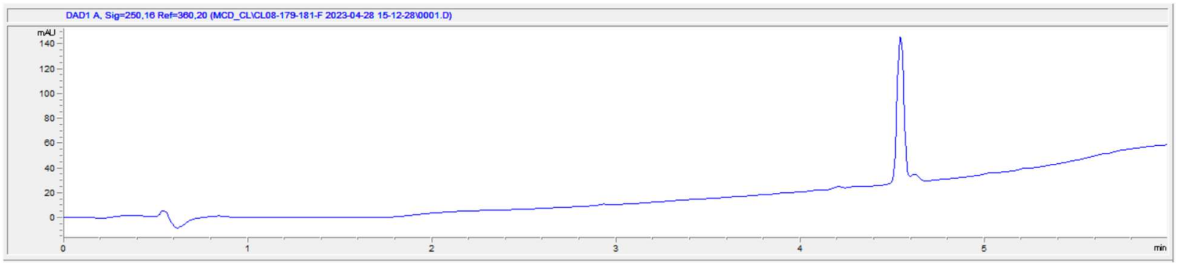

^{1}H NMR (600 MHz, DMSO-d_6) of compound **46**

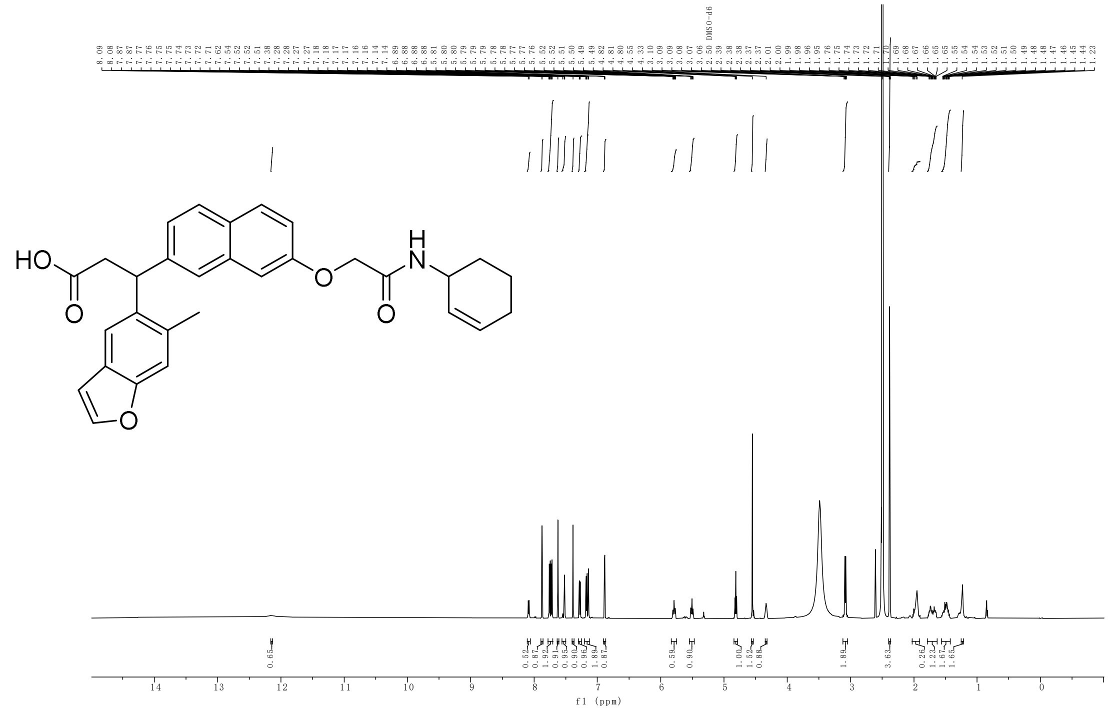

^{13}C NMR (151 MHz, DMSO-d_6) of compound **46**

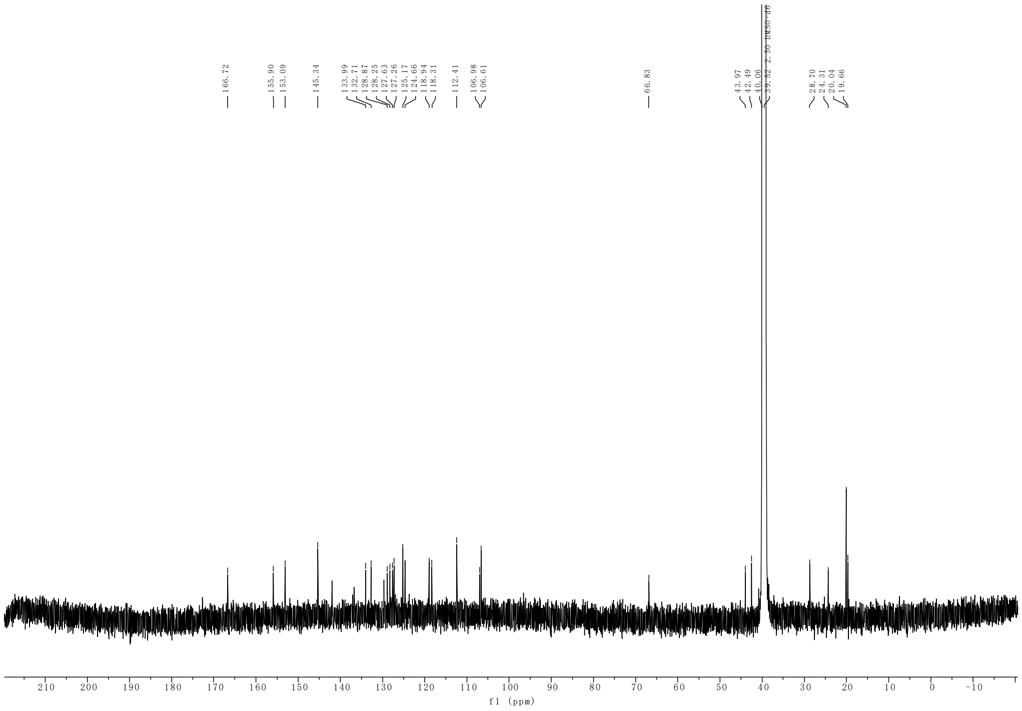

LC-MS Spectra (UV250) of compound **46**

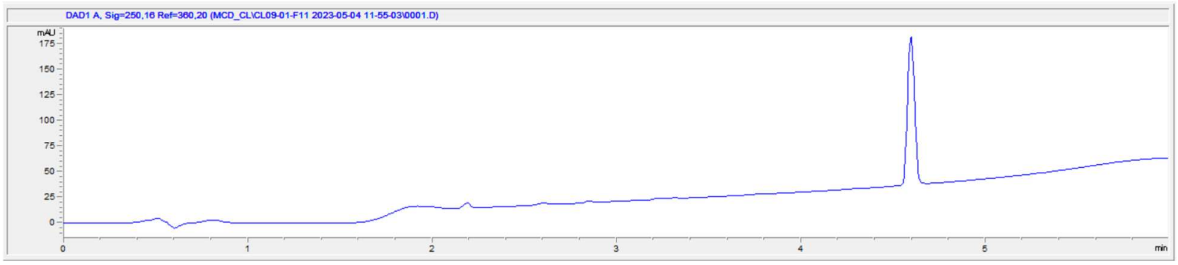

^{1}H NMR (600 MHz, DMSO-d_6) of compound **47**

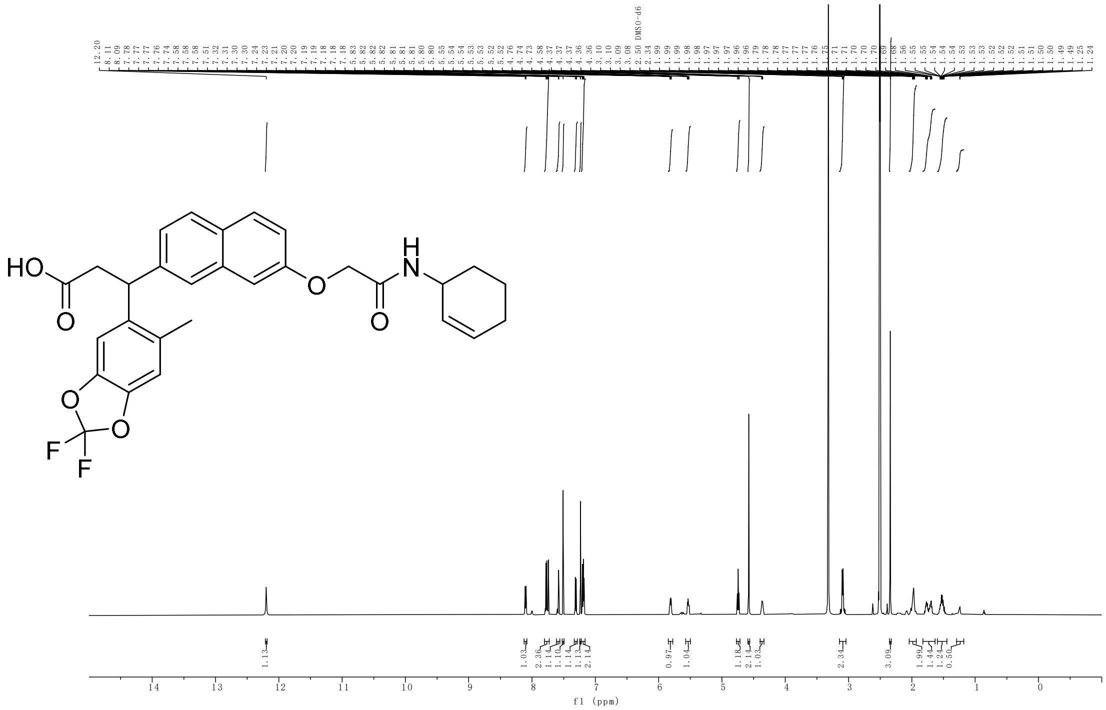

^{13}C NMR (151 MHz, DMSO-d_6) of compound **47**

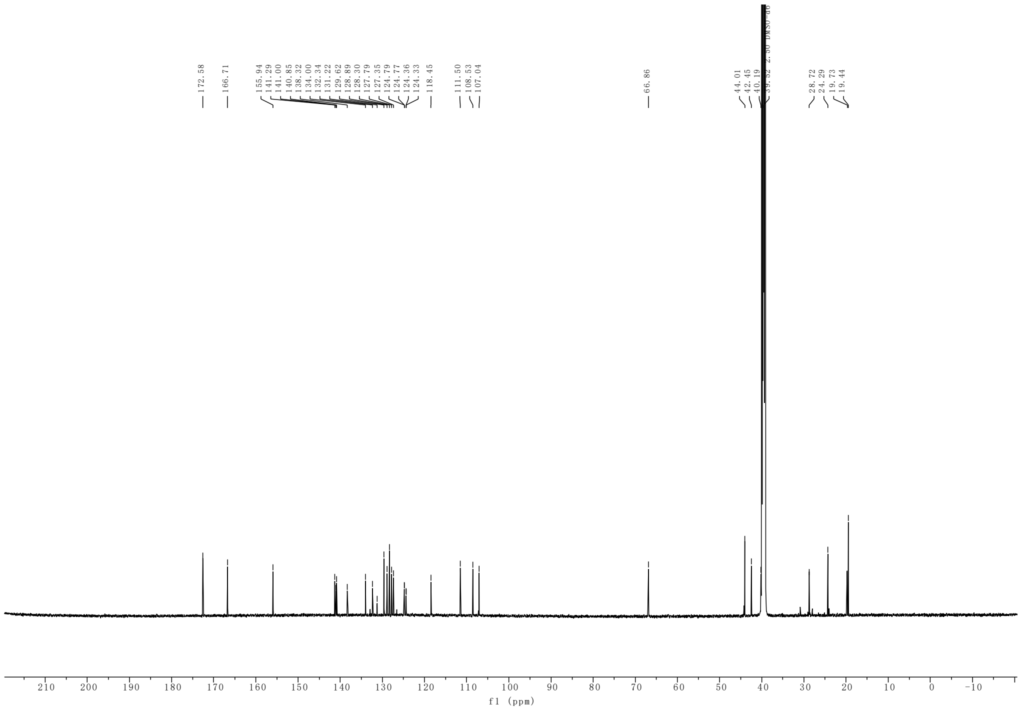

LC-MS Spectra (UV250) of compound **47**

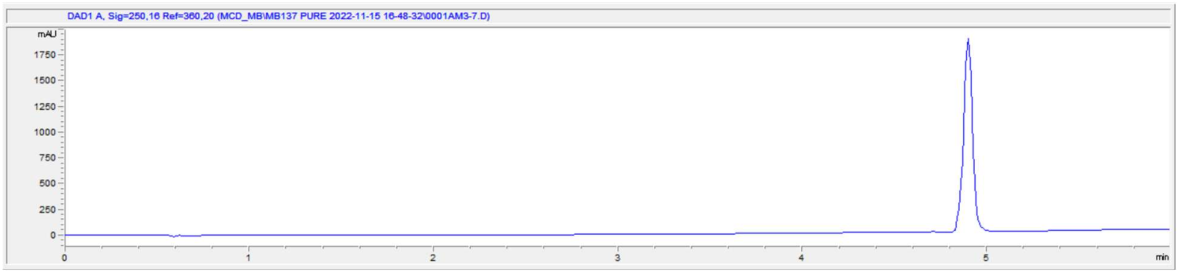

^{1}H NMR (600 MHz, DMSO-d_6) of compound **48**

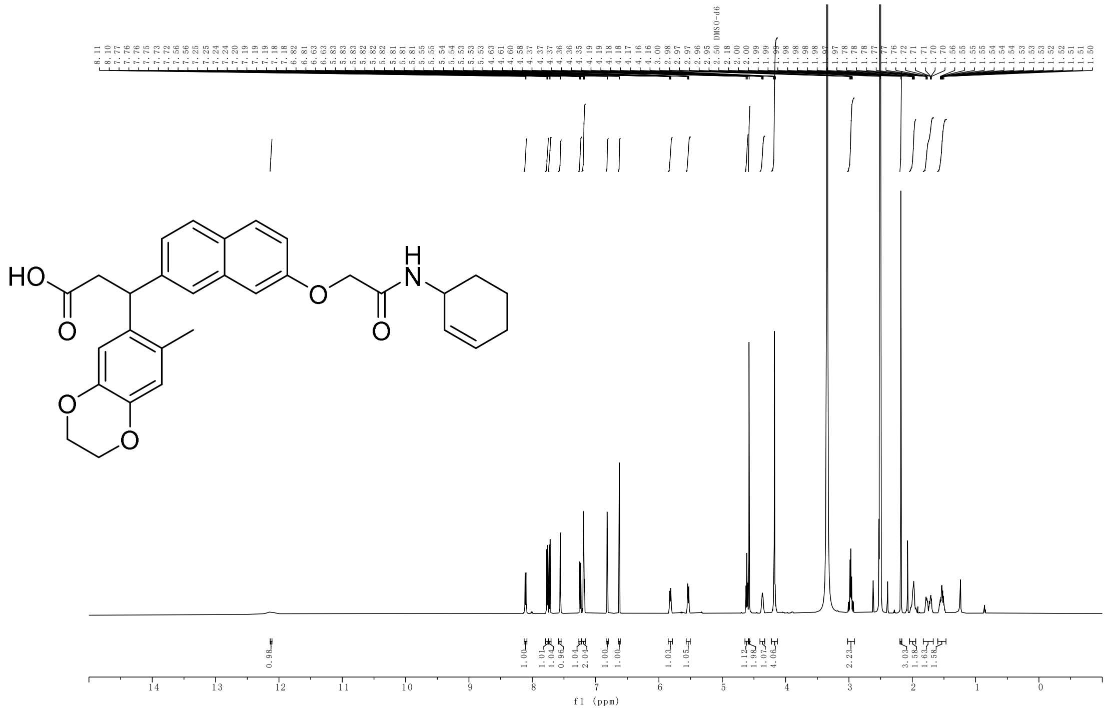

^{13}C NMR (151 MHz, DMSO-d_6) of compound **48**

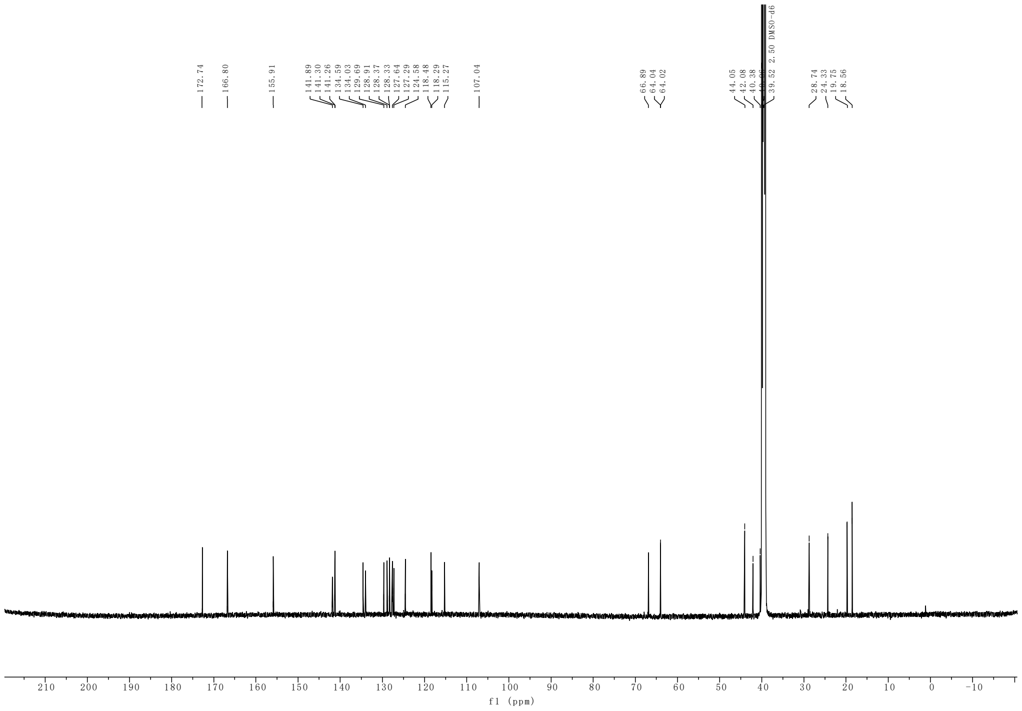

LC-MS Spectra (UV250) of compound **48**

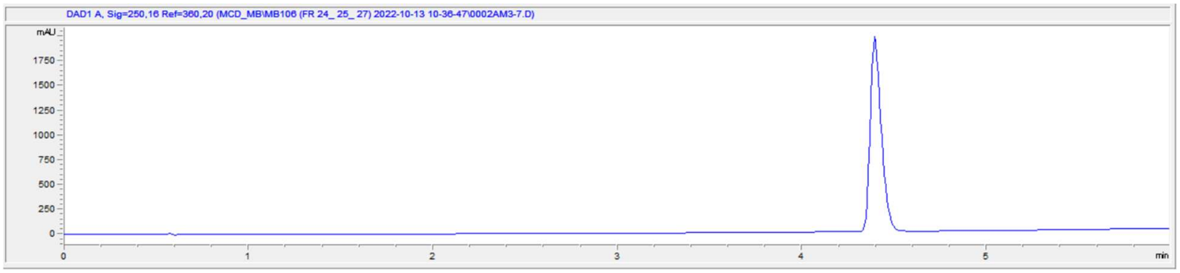

Supporting References

[1] K. T. Tran, J. S. Pallesen, S. M. Ø. Solbak, D. Narayanan, A. Baig, J. Zang, A. Aguayo-Orozco, R. M. C. Carmona, A. D. Garcia, A. Bach, *J. Med. Chem.* **2019**, *62*, 8028–8052.

[2] D. Norton, W. G. Bonnette, J. F. Callahan, M. G. Carr, C. M. Griffiths-Jones, T. D. Heightman, J. K. Kerns, H. Nie, S. J. Rich, C. Richardson, W. Rumsey, Y. Sanchez, M. L. Verdonk, H. M. G. Willems, W. E. Wixted, L. Wolfe, A. J.-A. Woolford, Z. Wu, T. G. Davies, *J. Med. Chem* **2021**, *64*, 15949–15972.

[3] J. S. Pallesen, D. Narayanan, K. T. Tran, S. M. Ø. Solbak, G. Marseglia, L. M. E. Sørensen, L. J. Høj, F. Munafò, R. M. C. Carmona, A. D. Garcia, H. L. Desu, R. Brambilla, T. N. Johansen, G. M. Popowicz, M. Sattler, M. Gajhede, A. Bach, *J. Med. Chem.* **2021**, *64*, 4623–4661.

[4] D. Narayanan, K. T. Tran, J. S. Pallesen, S. M. Ø. Solbak, Y. Qin, E. Mukminova, M. Luchini, K. O. Vasilyeva, D. Gonzalez Chichón, G. Goutsiou, C. Poulsen, N. Haapanen, G. M. Popowicz, M. Sattler, D. Olagnier, M. Gajhede, A. Bach, *J. Med. Chem.* **2022**, *65*, 14481–14526.

[5] Y. Qin, C. Poulsen, D. Narayanan, C. B. Chan, X. R. Chen, B. R. Montes, K. T. Tran, E. Mukminova, C. Y. Lin, M. Gajhede, A. N. Bullock, D. Olagnier, A. Bach, *J. Med. Chem.* **2024**, *67*, 18828−18864.

[6] D. Fearon, A. Powell, A. Douangamath, A. Dias, C. W. E. Tomlinson, B. H. Balcomb, J. C. Aschenbrenner, A. Aimon, I. A. Barker, F. Bertram, J. Brandão-Neto, P. A. Coe, P. Collins, L. E. Dunnett, M. Fairhead, R. J. Gildea, M. Golding, T. Gorrie-Stone, P. V. Hathaway, L. Koekemoer, T. Krojer, R. M. Lithgo, E. M. Maclean, P. G. Marples, H. Mikolajek, X. Ni, K. H. V. Nidamarthi, G. O'Donnell, R. Skyner, R. Talon, W. Thompson, G. Watt, C. F. Wild, M. A. Williams, M. Winokan, N. D. Wright, G. Winter, E. J. Shotton, F. von Delft, *Appl. Res.* **2025**, *4*, e202400192.

[7] T. Ursby, K. Åhnberg, R. Appio, O. Aurelius, A. Barczyk, A. Bartalesi, M. Bjelcic, F. Bolmsten, Y. Cerenius, R. B. Doak, M. Eguiraun, T. Eriksson, R. J. Friel, I. Gorgisyan, A. Gross, V. Haghighat, F. Hennies, E. Jagudin, B. Norsk Jensen, T. Jeppsson, M. Kloos, J. Lidon-Simon, G. M. A. de Lima, R. Lizatovic, M. Lundin, A. Milan-Otero, M. Milas, J. Nan, A. Nardella, A. Rosborg, A. Shilova, R. L. Shoeman, F. Siewert, P. Sondhauss, V. O. Talibov, H. Tarawneh, J. Thånell, M. Thunnissen, J. Unge, C. Ward, A. Gonzalez, U. Mueller, *J. Synchrotron Radiat.* **2020**, *27*, 1415−1429.

[8] O. B. Cox, T. Krojer, P. Collins, O. Monteiro, R. Talon, A. Bradley, O. Fedorov, J. Amin, B. D. Marsden, J. Spencer, F. von Delft, P. E. Brennan, *Chem. Sci.* **2016**, *7*, 2322–2330.

[9] W. Kabsch, *Acta Crystallogr. D Biol. Crystallogr.* **2010**, *66*, 125−132.

[10] A. J. McCoy, R. W. Grosse-Kunstleve, P. D. Adams, M. D. Winn, L. C. Storoni, R. J. Read, *J. Appl. Crystallogr.* **2007**, *40*, 658−674.

[11] F. Long, R. A. Nicholls, P. Emsley, S. Grazulis, A. Merkys, A. Vaitkus, G. N. Murshudov, *Acta Crystallogr. D Struct. Biol.* **2017**, *73*, 112−122.

[12] N. W. Moriarty, R. W. Grosse-Kunstleve, P. D. Adams, *Acta Crystallogr. D Biol. Crystallogr.* **2009**, *65*, 1074−1080.

[13] P. Emsley, B. Lohkamp, W. G. Scott, K. Cowtan, *Acta Crystallogr. D Biol. Crystallogr.* **2010**, *66*, 486–501.

[14] P. V. Afonine, R. W. Grosse-Kunstleve, N. Echols, J. J. Headd, N. W. Moriarty, M. Mustyakimov, T. C. Terwilliger, A. Urzhumtsev, P. H. Zwart, P. D. Adams, *Acta Crystallogr. D Biol. Crystallogr.* **2012**, *68*, 352−367.

[15] The PyMOL Molecular Graphics System, Version 2.5.5 Schrödinger, LLC.

[16] Schrödinger Release 2022-1: Maestro, Schrödinger, LLC, New York, NY, 2022.

[17] T. G. Davies, W. E. Wixted, J. E. Coyle, C. Griffiths-Jones, K. Hearn, R. McMenamin, D. Norton, S. J. Rich, C. Richardson, G. Saxty, H. M. G. Willems, A. J.-A. Woolford, J. E. Cottom, J.-P. Kou, J. G. Yonchuk, H. G. Feldser, Y. Sanchez, J. P. Foley, B. J. Bolognese, G. Logan, P. L. Podolin, H. Yan, J. F. Callahan, T. D. Heightman, J. K. Kerns, *J. Med. Chem.* **2016**, *59*, 3991−4006.

[18] Z. Nikolovska-Coleska, R. Wang, X. Fang, H. Pan, Y. Tomita, P. Li, P. P. Roller, K. Krajewski, N. G. Saito, J. A. Stuckey, S. Wang, *Anal. Biochem.* **2004**, *332*, 261−273.

[19] R. Adamson, Z. Chen, S. G. Bartual, R. Sethi, P. Canning, F. J. Sorrell, T. Krojer, F. von Delft, L. Toledo-Sherman, A. N. Bullock, *Zenodo*, **2019**, 10.5281/zenodo.3245339.

[20] J. Iegre, S. Krajcovicova, A. Gunnarsson, L. Wissler, H. Käck, A. Luchniak, S. Tångefjord, F. Narjes, D. R. Spring, *Chem. Sci.* **2023**, *14*, 10800−10805.

[21] E. Leary, E. T. Anderson, J. K. Keyes, T. R. Huskie, D. J. Blake, K. A. Miller, *Bioorg. Med. Chem.* **2023**, *78*, 117136.
